# Fluorescent Solvatochromic Probes for Long‐Term Imaging of Lipid Order in Living Cells

**DOI:** 10.1002/advs.202309721

**Published:** 2024-03-11

**Authors:** Takuya Tanaka, Atsushi Matsumoto, Andery S. Klymchenko, Eiji Tsurumaki, Junichi Ikenouchi, Gen‐ichi Konishi

**Affiliations:** ^1^ Department of Chemical Science and Engineering Tokyo Institute of Technology Tokyo 152‐8552 Japan; ^2^ Department of Biology Faculty of Sciences Kyushu University Fukuoka 819‐0395 Japan; ^3^ Laboratoire de Bioimagerie et Pathologies UMR 7021 CNRS Université de Strasbourg 74 route du Rhin Illkirch 67401 France; ^4^ Department of Chemistry Tokyo Institute of Technology Tokyo 152‐8552 Japan

**Keywords:** biological chemistry, biological techniques, biophysics, membrane organization, membrane probes, photostability, solvatochromic fluorescent dyes

## Abstract

High‐resolution spatio‐temporal monitoring of the cell membrane lipid order provides visual insights into the complex and sophisticated systems that control cellular physiological functions. Solvatochromic fluorescent probes are highly promising noninvasive visualization tools for identifying the ordering of the microenvironment of plasma membrane microdomains. However, conventional probes, although capable of structural analysis, lack the necessary long‐term photostability required for live imaging at the cellular level. Here, an ultra‐high‐light‐resistant solvatochromic fluorescence probe, 2‐*N*,*N*‐diethylamino‐7‐(4‐methoxycarbonylphenyl)‐9,9‐dimethylfluorene (FπCM) is reported, which enables live lipid order imaging of cell division. This probe and its derivatives exhibit sufficient fluorescence wavelengths, brightness, polarity responsiveness, low phototoxicity, and remarkable photostability under physiological conditions compared to conventional solvatochromic probes. Therefore, these probes have the potential to overcome the limitations of fluorescence microscopy, particularly those associated with photobleaching. FπCM probes can serve as valuable tools for elucidating mechanisms of cellular processes at the bio‐membrane level.

## Graphical abstract

Newly developed fluorescent solvatochromic dyes with excellent photostability, luminescence properties, and low phototoxicity that enable video observation under a microscope of the whole range of cell membrane physiological phenomena are reported. The dyes do not fade under continuous light irradiation for >1 h and can capture time‐resolved information on the order heterogeneity of membranes in living cells.

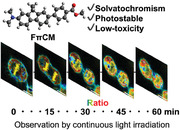

## 1 Introduction

Biological membranes not only separate cells and organelles from the surrounding environment but also govern various cellular functions, including cell morphogenesis, motility, material exchange, and signal transduction.^[^ ^1^ ^]^ Recent studies have highlighted the importance of lipid organization and membrane proteins for membrane function.^[^ ^2^ ^]^ The mechanisms underlying lipid functions have also received much attention in the context of clinical applications because defects in genes related to lipid biosynthesis can lead to inherited diseases^[^ ^3^ ^]^ and distinct alterations in lipid metabolism promote cancer cell growth and tumorigenesis.^[^ ^4^ ^]^ Membrane order and fluidity, influenced by lipid composition, significantly impact the structure and activity of membrane proteins.^[^ ^5^ ^]^ With the development of lipidomic analysis using shotgun mass spectrometry, it is now known that from microorganisms to mammals, cells robustly maintain membrane lipid composition at a suitable level.^[^ ^6^ ^]^ However, as it remains challenging to analyze local lipid composition in living cells, the establishment of novel tools that enable microscopic analysis of membrane fluidity is necessary to understand membrane structures that exert diverse functions in a precise manner.

The quantification of spatio‐temporal changes in lipids requires continuous observation using noninvasive fluorescent membrane probes. Solvatochromic dyes are among the most promising tools for visualizing the spatial distribution and biophysical properties of the liquid‐ordered (Lo) and liquid‐disordered (Ld) phases of the cell membrane because of their high sensitivity to local membrane properties, membrane permeability, and noninvasiveness.^[^ ^7^ ^]^ The standard noninvasive small solvatochromic fluorescent membrane probes, Prodan and Laurdan, were developed by Weber and Farris in 1979.^[^ ^8^ ^]^ Prodan and Laurdan can be easily introduced into the lipid layer but they are not suitable for continuous observation. They have the following disadvantages: cell damage due to UV excitation (350 nm), low absorbance, low quantum yield in low‐polarity regions (*Φ*_fl_ 0.02, *n*‐hexane), high concentration requirements, and low photostability.

In 2010, we designed the Prodan analog, fluorene (FR0), as a π‐system to extend the fluorescence wavelength and increase the absorption coefficient.^[^ ^9^ ^]^ Subsequently, in 2013, we also designed the Prodan analog, pyrene (PA), to maintain high quantum yields from nonpolar (*Φ*_fl_ 0.88 in *n*‐hexane) to polar (*Φ*_fl_ 0.85 in methanol) solvents.^[^ ^10^ ^]^ PA was successfully used to identify the distribution of cholesterol and sphingomyelin clusters in model membranes in 2016.^[^ ^11^ ^]^ However, aldehydes react with intracellular substrates or residues, leading to modifications of the chromophore and loss of luminescent properties.^[^ ^11^, ^12^, ^13^ ^]^ Therefore, a ketone analog of PA, PK, was developed and successfully applied for imaging lipid organization in cells and small animals.^[^ ^14^ ^]^ However, FR0, PA, and PK cannot completely suppress intersystem crossing (ISC) in low‐polarity solvents, which closely resemble the composition of cell membranes. ISC is a characteristic behavior of photoexcited triplet aromatic ketones.^[^ ^15^ ^]^ Consequently, this incomplete suppression of ISC leads to photobleaching owing to the photolysis of the dye.^[^ ^10^ ^]^ Additionally, intracellular reactions involving aldehydes and ketones have been reported, which also cause photobleaching. Moreover, the degradation products generated during these processes introduce spectral noise into the imaging data.

Generally, applications of solvatochromic cell membrane probes are limited to short‐term measurements.^[^ ^16^ ^]^ Shifting the probe's operating range to longer wavelengths is an attractive option to decrease phototoxicity, as seen with Nile Red dye.^[^ ^15^ ^]^ Nevertheless, even Nile Red probes present significant phototoxicity to specific cell membranes.^[^ ^13^ ^]^ Recent studies have explored exchangeable dyes based on Nile Red^[^ ^15^ ^]^ and other dyes^[^ ^17^ ^]^ to achieve long‐term measurements using strong excitation power of stimulated emission depletion (STED) microscopy.^[^ ^18^ ^]^ However, this approach requires a low affinity of the dye for bio‐membranes, which decreases the signal intensity in conventional microscopy.^[^ ^19^ ^]^ Therefore, a completely new molecular design of fluorophores is essential for continuous imaging of living cells under a microscope.

**Figure 1:**
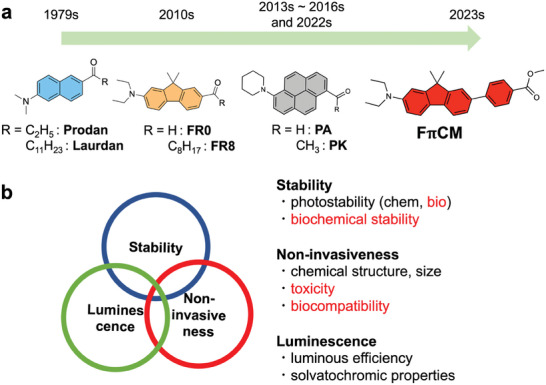
Development of solvatochromic membrane probes and molecular design concept. a) Historical development of solvatochromic membrane probes. b) Conceptual diagram of the molecular design. Elements in black are photochemically required, whereas elements in red are biologically required.

We recognized the importance of focusing on the physical attributes of dyes from a cell‐biological perspective. These attributes can be classified into three categories: luminescent properties, stability, and noninvasiveness (**Figure** **1**b). The realization of live imaging requires a limited and balanced molecular design, where the convergence of these three factors is mandated by the demands of both photochemistry and cell biology.

In this study, we introduce a solvatochromic fluorescent cell membrane probe that offers photostability and low toxicity, enabling long‐term (> 60 min) continuous observations. In the molecular design, we incorporated aromatic esters that do not react with cellular tissues, ensuring chemical stability and noninvasiveness. The photostability of aromatic esters can be explained by photophysical processes using energy diagrams such as those used for Prodan derivatives.^[^ ^10^, ^20^ ^]^ Moreover, we have explored strategies for suppressing and manipulating ISC and photodegradation by fine‐tuning their π‐systems. However, achieving a delicate equilibrium between molecular size and planarity is crucial, as it directly influences biocompatibility and invasiveness. We have examined several dyes, including conventional π‐systems and their conjugated extended forms, to develop probes suitable for bioimaging.

## 2 Results and Discussion

### 2.1 Photophysical Properties

**Figure 2:**
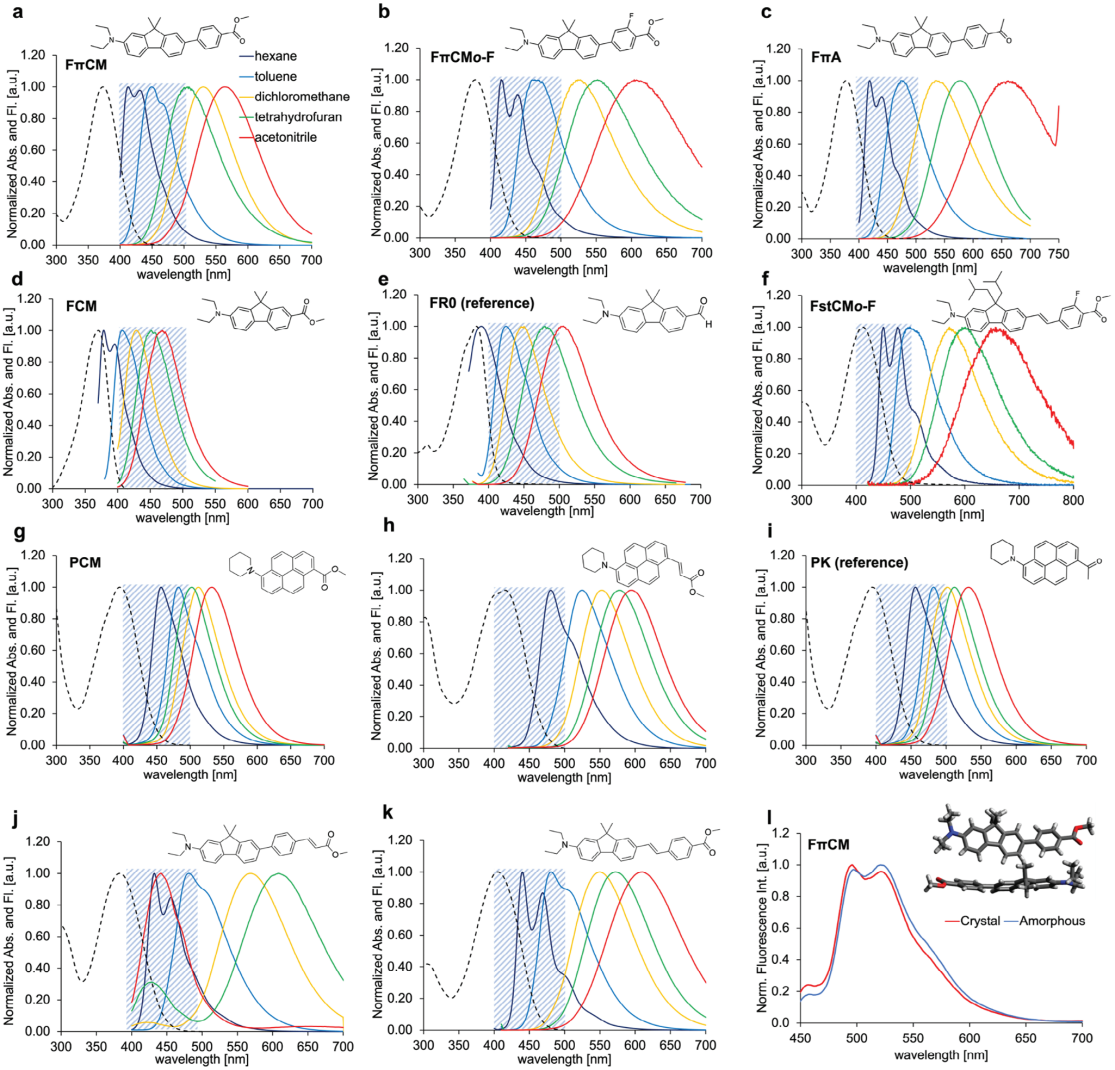
Spectroscopic properties. Chemical structure, absorption (dashed line), and fluorescence (solid line) spectra of a) FπCM, b) FπCM*o*‐F, c) FπA, d) FCM, e) FR0, f) FstCM*o*‐F, g) PCM, h) PCMst, i) PK, j) FπCMst and k) FstCM. The area of the blue mesh indicates the fluorescence range of Prodan. l) The fluorescence spectra in the solid crystal and amorphous states for FπCM and the single crystal packing structure of FπCM. The gray, white, red, and blue spheres represent C, H, O, and N, respectively.

We investigated the photophysical properties of the newly synthesized dyes as shown in **Figure** **2**a and **Table** **1**. In addition to the ester derivatives used in the Prodan (fluorene^[^ ^9^ ^]^ and pyrene^[^ ^10^ ^]^) and carbonyl derivatives, we considered a fluorene backbone with π‐conjugation extended in the long‐axis direction^[^ ^21^ ^]^ of the molecule to increase the dipole moment (*µ*). The photophysical properties of the ester derivatives were comparable to those of acetyl/formyl derivatives with the same π‐electron backbone, and by modifying it, strongly emitting dyes with various emission ranges were created. Among these, FCM, FπCM*o*‐F, PCM, PCMst, FπCMst, and FstCM exhibited excellent photophysical properties equal to or exceeding those of Prodan. FstCM*o*‐F emitted red light around 700 nm and holds promise as a cell membrane probe. However, not all dyes were suitable for cell imaging: FCM was excited by UV light, PCM, FπCM*o*‐F and FstCM*o*‐F had poor compatibility with cell membranes (Figure S138, Supporting Information). These dyes and FstCM had problems in the cell imaging conditions described below. The fluorine moiety in these compounds contributed to poor bio‐membrane compatibility.^[^ ^21^, ^22^ ^]^ Extensive trial‐and‐error studies have led us to conclude that FπCM is an effective membrane probe. The utility of FπCM as a probe is discussed below and the detailed photophysical properties of the other compounds are described in Section S3 (Supporting Information).

**Table 1:** Spectroscopic properties of FπCM, FπCM*o*‐F, FπA, FCM, FR0,^[^ ^9^ ^]^ FstCM*o*‐F, PCM, PCMst, PK,^[^ ^10^, ^28^ ^]^ FπCMst, FstCM and Prodan^[^ ^10^ ^]^ in various solvents and solid‐states.

		FπCM (*Δµ* = 17.2 D)^d)^				FπCM*o*‐F (*Δµ* = 17.2 D)^d)^				FπA (*Δµ* = 19.0 D)^d)^			
solvent	*ε*_r_ ^a)^	*Ε* [M^−1^ cm^−1^]	*λ*_abs_ [nm]	*λ*_fl_ [nm]	*Φ*_fl_ ^e)^ [–]	*ε* [M^−1^ cm^−1^]	*λ*_abs_ [nm]	*λ*_fl_ [nm]	*Φ*_fl_ ^e)^ [–]	*ε* [M^−1^ cm^−1^]	*λ*_abs_ [nm]	*λ*_fl_ [nm]	*Φ*_fl_ [–]
*n*‐Hexane	1.89	69600	364	431	0.88	65500	368	438	0.90	37200	369	440	0.85
Toluene	2.38	61400	372	450	0.83	57100	378	463	0.89	41500	378	477	0.93
THF	7.60	63200	373	504	0.87	69400	379	524	0.94	34200	377	536	0.98
DCM	9.08	61600	375	531	0.85	58100	379	552	0.84	37500	382	578	0.42
MeCN	37.5	63600	371	564	0.81	59700	376	604	0.77	41800	376	661	0.10
solid	–	–	–	496^b)^	0.48^b)^	–	–	505^b)^	0.54^b)^	–	–	492^b)^	0.03^b)^
				496^c)^	0.43^c)^			507^c)^	0.23^c)^				

		FCM (*Δµ* = 13.3 D)^d)^				FR0 (*Δµ* = 14.0 D)^d)^				FstCM*o*‐F (*Δµ* = 19.0 D)^d)^			
solvent	*ε*_r_ ^a)^	*Ε* [M^−1^ cm^−1^]	*λ*_abs_ [nm]	*λ*_fl_ [nm]	*Φ*_fl_ ^e)^ [–]	*ε* [M^−1^ cm^−1^]	*λ*_abs_ [nm]	*λ*_fl_ [nm]	*Φ*_fl_ ^e)^ [–]	*ε* [M^−1^ cm^−1^]	*λ*_abs_ [nm]	*λ*_fl_ [nm]	*Φ*_fl_ [–]
*n*‐Hexane	1.89	47600	368	379	0.92	42000	387	395	0.11	42300	405	450, 476	0.89
Toluene	2.38	39500	373	407	0.98	43000	395	434	0.98	43800	412	500	0.89
THF	7.60	37900	371	429	0.93	40000	393	462	0.85	39300	412	571	0.85
DCM	9.08	37900	370	452	0.84	40000	399	497	0.75	37500	427	600	0.83
MeCN	37.5	37800	370	469	0.96	35000	394	518	0.56	38300	409	671	0.45
Solid	–	–	–	385^b)^	0.12^b)^	–	–	–	0.02	–	–	522^b)^	0.09^b)^
												533^c)^	0.12^c)^

		PCM (*Δµ* = 9.30 D)^d)^				PCMst (*Δµ* = 14.5 D)^d)^				PK (*Δµ* = 9.40 D)^d)^			
Solvent	*ε*_r_ ^a)^	*ε* [M^−1^ cm^−1^]	*λ*_abs_ [nm]	*λ*_fl_ [nm]	*Φ*_fl_ ^e)^ [–]	*ε* [M^−1^ cm^−1^]	*λ*_abs_ [nm]	*λ*_fl_ [nm]	*Φ*_fl_ ^e)^ [–]	*ε* [M^−1^ cm^−1^]	*λ*_abs_ [nm]	*λ*_fl_ [nm]	*Φ*_fl_ ^e)^ [–]
*n*‐Hexane	1.89	19500	392	456	0.89	33200	400	480	0.75	17400	396	473	0.67
Toluene	2.38	19500	391	482	0.96	30300	417	525	0.98	18700	402	504	0.80
THF	7.60	19100	394	503	0.89	31100	415	553	0.97	17000	401	528	0.79
DCM	9.08	18100	398	512	0.92	29700	420	577	0.95	16700	407	538	0.83
MeCN	37.5	18200	394	536	0.77	30500	414	595	0.95	14000	400	549	0.76
Solid	–	–	–	522^b)^	0.09^b)^	–	–	522^b)^	0.72^b)^	–	–	–	0.01
				533^c)^	0.12^c)^								

		FπCMst (*Δµ* = 20.5 D)^d)^				FstCM (*Δµ* = 18.7 D)^d)^				Prodan^g)^ (*Δµ* = 7.0 D)^d)^			
Solvent	*ε*_r_ ^a)^	*ε* [M^−1^ cm^−1^]	*λ*_abs_ [nm]	*λ*_fl_ [nm]	*Φ*_fl_ ^e)^ [–]	*ε* [M^−1^ cm^−1^]	*λ*_abs_ [nm]	*λ*_fl_ [nm]	*Φ*_fl_ ^e)^ [–]	*ε* [M^−1^ cm^−1^]	*λ*_abs_ [nm]	*λ*_fl_ [nm]	*Φ*_fl_ [–]
*n*‐Hexane	1.89	52000	376	432, 456	0.79	67600	399	440, 469	0.72	18400^g)^	340	389	0.02
Toluene	2.38	45100	385	481	0.99	57700	407	502	0.94	–	347	416	0.56
THF	7.60	47100	383	568	0.99	59600	405	552	0.93	–	348	430	0.78
DCM	9.08	45700	386	608	0.24	58200	408	591	0.93	–	355	440	0.98
MeCN	37.5	49200	380	441	0.03	60400	402	626	0.89	–	350	455	0.80
Solid	–	–	–	500^b)^	0.71^b)^	–	–	525^b)^	0.70^b)^	–	–	–	–

Definitions: *λ*_abs_, maximum absorption wavelength; *λ*_fl_, maximum fluorescence wavelength; *Φ*_fl_, absolute fluorescence quantum yield; THF, tetrahydrofuran; DCM, dichloromethane; MeCN, acetonitrile; Δ*µ* = *µ*_e_‐µg.

^a)^ Dielectric constants;

^b)^ Amorphous Solids;

^c)^ Polycrystalline solid;

^d)^ Δ*µ* estimated by Lippert‐Mataga equation (Equation. S4, Supporting Information);

^e)^ Error ± 0.03;

^f)^ Prodan data were obtained from Reference [10];

^g)^ Prodan absorption coefficient from Reference [10].

FπCM exhibited a large absorption coefficient in toluene (*λ*_abs‐max_ 372 nm: *ε* = 57100 M^−1^cm^−1^; a common laser source 405 nm: *ε* = 26000 M^−1^cm^−1^). Absorption wavelengths (*λ*_abs_s) were nearly independent of changes in solvent polarity (*λ*_abs_ ≤ 7.0 nm), and there was no solvatochromism in the ground state. When diethylamine in FπCM was replaced with piperidine (FπpCM),^[^ ^23^ ^]^ a blue shift in *λ*_abs_ was observed, and when fluorine was introduced into the phenyl group of FπCM to compensate for the electron‐withdrawing property of the ester (FπCM*o*‐F),^[^ ^24^ ^]^ a red shift in wavelength was observed.^[^ ^25^ ^]^ The introduction of fluorine was also observed to have a property that contributes to the red shift of the fluorescence spectrum in medium to high polarity solvent. (Figure S69, Supporting Information) Computational results support this trend, with the broadening of the π conjugation due to the introduction of fluorine, the HOMO–LUMO energy gap and absorption spectra of FπCM and FπCM*o*‐F (Figure S70, Supporting Information). Thus, the introduction of substituents can tune the absorption and fluorescence wavelength.

FπCM maintained solvatochromic properties with a wide fluorescence range (431–564 nm; 5471 cm^−1^) and a high *Φ*_fl_ (≥ 0.81) over a wide polar range from *n*‐hexane (relative permittivity (*ε*_r_) = 1.89) to acetonitrile (37.5). The fluorescence range (431–564 nm; 5471 cm^−1^) was wider than that of Prodan (389–455 nm; 3729 cm^−1^) and PK (473–549 nm; 2927 cm^−1^). In FπCM, where the fluorescence spectrum shifts significantly from low to high polarity, high‐resolution color separation can be expected in ratiometric analysis, which separates the fluorescence intensity ratio at a certain wavelength.

The solvent dependence of FπCM fluorescence exhibited a characteristic property compared to the conventional dyes: The absorption and fluorescence spectra of FπCM demonstrated a linear dependence of the Stokes shift on orientation polarizability (Δ*f*), consistent with the Lippert‐Mataga equation (Equation 1):

(1) $$\Delta \nu =\frac{2{\left(\mu_{e}-\mu_{g}\right)}^{2}\Delta f}{\mathrm{hc}a^{3}}+\mathrm{const}.$$

where *µ*_e_ and *µg* are the dipole moments of the excited and ground states, respectively. *h* is the Planck constant, *c* is the light velocity, and *a* is the Onsager cavity radius. The fit of FπCM using Equation (1) (Figure S60, Supporting Information) gives Δ*µ* (*µ*_e_‐*µ_g_*) = 17.2 D, which is ≈3.2 D higher than that of the conventional FR0. This result is characteristic of solvatochromic dyes with an ICT mechanism, where dyes with a large Δ*µ* have a larger charge separation in the excited state, as explained by the energy gap law.^[^ ^26^ ^]^ In the high‐polarity region, the non‐radiative deactivation rate constant (*k*_nr_) is due to internal conversion compared to dyes with smaller charge separation. For example, the *k*_nr_ of FR0 (14.0 D) tends to be smaller, resulting in a significant decrease in *Φ*_fl_.^[^ ^26^, ^27^ ^]^ However, for FπCM, *k*_nr_ remained almost constant regardless of the polarity environment (Table S1, Supporting Information). In the case of the acetyl derivative FπA (16.7 D), the *k*_nr_ in acetonitrile follows the energy gap decrease, resulting in *Φ*_fl_ = 0.10. (Table 1). The stable and high *Φ*_fl_ value of FπCM is a major benefit of esters.

FπCM exhibited similar fluorescence spectra and a high *Φ*_fl_ of 0.45, regardless of the solid‐state order of the amorphous and crystalline phases (Figure 2i). Single crystal X‐ray structural analysis and quantum chemical calculations (Figures S90 and S91, Supporting Information, respectively) revealed that FπCM has a similar crystal packing structure to FπA, although the *Φ*_fl_ values were significantly different (FπCM: 0.43 vs FπA: 0.03). Other ester compounds also exhibited fluorescence properties in the solid state that are not present in the acetyl and formyl derivatives (Figure S82 and Sections S3 and S4, Supporting Information)

The fluorescence brightness (absorption coefficient *ε* × *Φ*_fl_) of FπCM achieved an excellent value of >21000, regardless of solvent polarity. Given the low polarity of the cell membrane, FπCM (22800) was more efficient than Prodan (370)^[^ ^10^ ^]^ and PK (11700)^[^ ^10^, ^28^ ^]^ in *n*‐hexane. This suggests that color discrimination imaging, suitable for analysis using lower concentrations of FπCM (i.e., lower toxicity), is feasible.

### 2.2 Photostability

**Figure 3:**
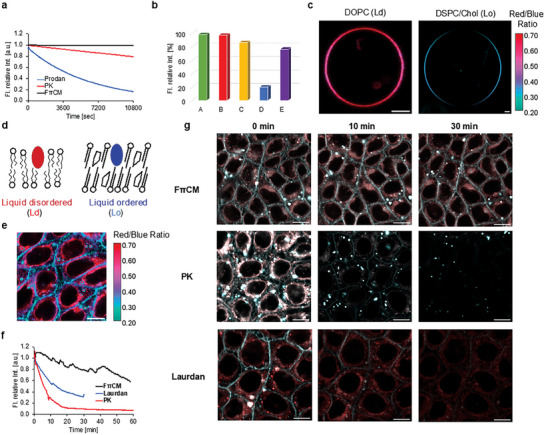
a) Photodegradation curves of FπCM, PK, and Prodan in toluene (1.0×10^−5^ M) under oxygen. The excitation wavelength (*λ*_ex_) was 375 nm. b) Comparison of fluorescence intensity ratio with the initial fluorescence intensity after 3 h of light irradiation in toluene (1.0×10^−5^ M) under oxygen. A: FπCM, B: FstCM*o*‐F, C: PK, D: Prodan, E: NR. NR data was obtained from ref. [28]. c) Confocal fluorescence images showing Ld and Lo phases in GUVs stained with FπCM. GUVs were composed of DOPC (Ld) and DSPC/Cho (Lo). The ratio of red (>500 nm) and blue (<500 nm) channels were displayed as color (right bar) and the mean signal intensity of both channels was displayed as brightness. Scale bars: 5 µm. d) Schematic image of Liquid‐disordered (Ld) and Liquid‐ordered (Lo) phases in the model membrane. Dyes exhibiting different wavelengths of fluorescence are also shown. e) Confocal image of EpH4 stained with FπCM. Red/Blue ratio and intensity are displayed as (c). f) Photodegradation curves of FπCM, PK, and Laurdan as a function of time in the cell membranes under continuous light irradiation. *λ*_ex_ = 405 nm. g) Time‐lapse images of fluorescent dyes in cell membranes under continuous light irradiation for FπCM, Laurdan, and PK. Cells were stained with a 10 µM probe for 5 min at 37 °C in 100 µL of Hanks’ balanced salt solution (HBSS). Subsequently, the solution was diluted with 1 mL of HBSS. *λ*_ex_ = 405 nm Laser, 10%. Scale bars: 5 µm in scale.

We evaluated the photostability of the solvatochromic dyes by measuring the photodegradation curves of continuous light irradiation in the presence of oxygen in a low‐polarity solvent (model) and under physiological conditions using live cells. Photostability varies greatly depending on the microscopic observation conditions, object of observation, and nature of the dye. Therefore, there is no standard evaluation method applicable to all dyes. To address this, we used toluene as a solvent for the measurements, given that it has similar polarity to cell membranes. The photodegradation curves of FπCM, PK, and Prodan are illustrated in **Figure** **3**a,b, while those of the other ester compounds are shown in Figures S78 and S79 (Supporting Information). All ester compounds maintained >94% of their initial fluorescence intensity after 3 h, showing excellent photostability compared to PK (86%), Prodan (20%), and NR (76%)^[^ ^18^, ^29^ ^]^ which are used for STED microscopy, where photostability is required. This result aligns with the energy diagram results (Figures S119–S121, Supporting Information), which indicate that ISC is unlikely to occur in the ester derivatives.

To assess the applicability of these ester derivatives for cellular labeling, we first compared these dyes (FπCM, PCM, FstCM, and FstCM*o*‐F) with distinct structural properties, by staining EpH4 live cells with each dye and observing them, under identical conditions. Among them, FπCM produced remarkably higher signal intensity than any other dyes (Figure S138, Supporting Information). The lower signal of PCM compared to FπCM suggests that fluorene chromophore is more suited than pyrene for cellular staining. For the fluorene derivatives, the order of the observed signal intensity was FπCM > FstCM > FstCM*o*‐F, suggesting that larger chemical structures may disturb the accessibility of dyes to cells, presumably due to low aqueous solubility. Therefore, we concluded that FπCM was the most suitable among this group of dyes for cell imaging, and it was employed in subsequent analysis.

**Figure 4:**
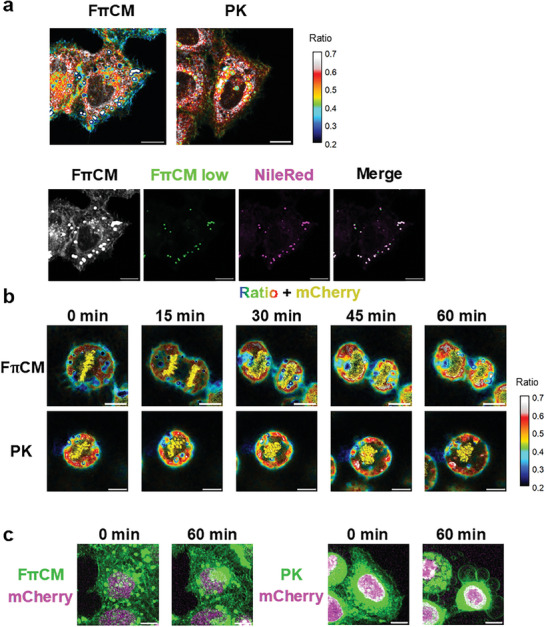
a) Upper panels, Live HeLa cells stained with 1 µM FπCM + 100 nm NileRed, or 1 µM PK. Red/Blue ratio and signal intensity are displayed as Figure 3c. Note that intracellular white (= high ratio) particles with blue (= low ratio) circular contours are attributed to lipid droplets. Lower panels, colocalization of bright particles of FπCM, and lipid droplet (NileRed). b,c) Simultaneous observation with solvatochromic dyes and a fluorescent protein in live cells: b) Comparison of the effect of FπCM and PK on the progression of HeLa cell division. Pseudo colors represent the intensity ratio of the red channel (500–700 nm) to the blue channel (400–500 nm), and brightness represents the average intensity of the two channels. Chromosomal morphology was simultaneously observed with mCherry‐conjugated H2B. Cells were incubated with 1 µM of the dyes for 10 min. 405 nm laser: 5%; 561 nm laser: 1%. Scale bars: 10 µm. Notably, we used a lower laser intensity than in the photostability observation in Figure 2. The intensity was manually adjusted to facilitate the integration of the ratiometric results. See Videos S1 and S2. c) Comparison of effects on interphase cell morphology. Signals from the blue channel of FπCM or PK (green) and mCherry signals are shown. Scale bars: 10 µm.

We next examined whether FπCM could detect membrane order in model membranes and living cells. As a model for biological membranes, giant unilamellar vesicles (GUVs) were stained with FπCM to assess their ability to distinguish between liquid‐ordered (Ld) and liquid‐disordered (Lo) phases, using confocal microscopy. Consistent with the spectroscopic properties in solvents with different polarities (Figure 2a), a high Red (> 500 nm)/Blue (< 500 nm) ratio was observed in Ld phase vesicles composed of DOPC, whereas a low ratio was observed in Lo phase vesicles of DSPC/Chol (Figures 3c and 3d). Next, we examined the membrane‐order heterogeneity within living cells. As shown in Figure 3e, FπCM revealed remarkable difference in order between cell‐surface plasma membrane rich in saturated lipids, sphingolipids, and cholesterol and inner organelles rich in unsaturated lipids^[^ ^5^ ^]^ in EpH4 mouse epithelial cells, L‐929 and NIH3T3 mouse fibroblasts, and human‐derived HeLa cells (**Figure** **4**a; Figure S137, Supporting Information), indicating that FπCM can detect membrane heterogeneity of cells irrespective of cell line and host species. These results demonstrate that FπCM can reveal membrane‐order heterogeneity as shown earlier for PK.^[^ ^14^ ^]^

We therefore compared the photostability of FπCM, PK, and Laurdan under physiological conditions in living cells. Figure 3f presents the photodegradation curves of FπCM, PK, and Laurdan during continuous light irradiation in EpH4 cells. Compared to the photodegradation curve in the toluene solvent, PK lost most of its luminescence at the beginning of the measurement. This may be attributed to the modification of the fluorescence unit resulting from the reaction with substrates in living cells, in addition to photodegradation. Laurdan and PK maintained only ≈30% and 10% of their initial fluorescence intensity, respectively, whereas FπCM maintained ≈80%. The exponential approximation curve derived from the decay curve in Figure 3f is shown in Figure S136 (Supporting Information), indicating that PK and Laurdan would be completely quenched in ≈30 min, whereas FπCM would persist for ≈5 h. Here, a relatively intense confocal laser light (10%) is used, suggesting that FπCM is resistant to the laser light intensity of various devices.

### 2.3 Bioimaging Application

We examined the advantage of this photostable membrane probe, FπCM, over PK for time‐course observations of live cells (Figure 4; Supplementary Videos S1 and S2, Supporting Information). Both FπCM and PK revealed membrane order heterogeneity of HeLa cells (Figure 4a), with essentially the same ratiometric images except slightly higher signal intensity in the plasma membrane region observed for FπCM. Again, the order of heterogeneity between the plasma membrane with higher order and the inner organelle with lower order was observed. Although notable ratio heterogeneity within intracellular regions was also observed, most of it may be attributed to signals from lipid droplets, the low polarity oil‐like organelle composed of neutral lipids, since brightest signals from FπCM co‐localized with well‐known lipid droplet marker Nile Red (Figure 4a).

We selected the progression of cell division as an indicator of the maintenance of physiological integrity in the cells. To visualize the morphology of the nuclei, mCherry‐conjugated histone H2B protein was transfected into HeLa cells, and images were acquired sequentially for each optical section at 405 nm excitation for FπCM/PK and 561 nm for mCherry. As shown in Figure 4b, both FπCM and PK detected membrane order heterogeneity, with low values of red/blue signals in the plasma membrane and high ratio values in inner membranes. The signals from the fluorescent protein mCherry, excited at 405 nm, did not affect the results of these ratiometric analyses, and the signals from FπCM excited at 561 nm were negligible. In terms of their effects on cell physiology, cell division progressed only during the observation with FπCM and not with PK. The progression of cell division in multiple cells within a single field of view (Video S1, Supporting Information) suggested that FπCM had almost no cytotoxicity under these observation conditions. We also compared the effects of time‐course observations on the morphology of the interphase cells (Figure 4c). No remarkable morphological changes occurred during FπCM observation, whereas large membrane blebs, similar to those observed as apoptosis progresses, were formed during PK observation (Video S2, Supporting Information).^[^ ^29^, ^30^ ^]^ In summary, FπCM produces lower toxicity than previously reported PK and therefore is more suitable for time‐course analysis of cellular processes with simultaneous observation with fluorescent proteins.

**Figure 5:**
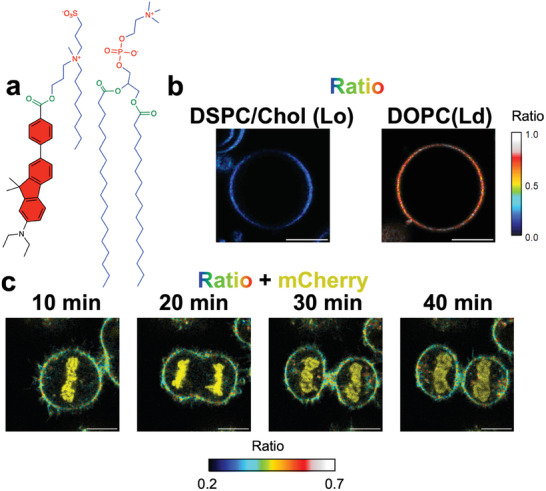
a) Structure of FπCM‐SO_3_. b) Ratiometric images of giant unilamellar vesicles (GUVs) of Lo (left, DSPC:cholesterol = 2:1) and Ld phases (right, DOPC) stained with FπCM‐SO_3_. Images were displayed in the same manner as a. 405 nm laser: 2.8% (left) and 1.5% (right). Scale bars: 10 µm. c) Ratiometric images of HeLa cells expressing histone 2B‐mCherry stained with FπCM‐SO_3_ displayed in the same manner as in Figure 4b. 405 nm laser: 1%; 561 nm laser: 1%. Scale bars: 10 µm. See Video S3 (Supporting Information).

As shown in Figure 4b, the difference in membrane order between different organelles (e.g., between the plasma membrane and endoplasmic reticulum) was much larger than the heterogeneity within a single continuous membrane (e.g., within the plasma membrane), making it difficult to analyze the spatiotemporal differences in order in a continuous membrane. One way to overcome this difficulty is organelle‐specific localization of dyes employing specific functional groups.^[^ ^30^, ^31^ ^]^ Therefore, we utilized a technique in which a linker consisting of a zwitterionic group and a long hydrophobic chain is coupled to selectively localize to the plasma membrane.^[^ ^17^, ^32^ ^]^ Compared to ketone‐based probes, modification methods for ester‐based FπCM are well described, and a high yield of the product FπCM‐SO_3_ was obtained (**Figure** **5**b; see also Section S2, Supporting Information). As shown in Figure 5b, the sensitivity to membrane order in GUVs was not disturbed by the amphiphilic anchor group. The effects of light polarization indicate that the fluorophore is orientated parallel to the fatty acid chains. When live cells were stained with FπCM‐SO_3_, signals from any inner membranes were remarkably reduced (Figure 5c) compared to the parental FπCM (Figure 4b). Although plasma membrane targeting tends to cause high toxicity,^[^ ^13^ ^]^ cell division was not affected by FπCM‐SO_3_ observation. These results indicate that the newly synthesized ester‐based probe FπCM is also applicable to organelle‐targeted observations with less toxicity.

## 3 Conclusion

In conclusion, we developed a practical lipid membrane probe that offers stability, luminescence properties, and low cytotoxicity, departing from the conventional solvatochromic dye design. A key strategy involved employing ester group donor‐π‐acceptor type ICT fluorophores. These ester groups possess weaker electron‐withdrawing properties than those of alkyl carbonyl groups. However, when we introduced the ester groups into the appropriate π‐electron framework, we achieved fluorescent solvatochromic properties comparable to alkyl carbonyl groups, and superior photostability. Additionally, FπCM exhibited high structural stability and can be stored at room temperature under an oxygen atmosphere, making it significantly easier to handle than carbonyl compounds, which are sensitive to oxygen. The combination of an appropriate π‐electron backbone and ester group holds potential for a wide range of organic fluorescent dyes, soft materials, and organic‐inorganic hybrid materials in the future, extending beyond solvatochromic dyes.

The biocompatibility, photostability, and low toxicity of FπCM allow for noninvasive, long‐term observations of lipid order heterogeneity in membranes of living cells, enabling the study of various biological phenomena. Given the established methods for localizing cell membrane probes to intracellular organelles,^[^ ^30^, ^31^ ^]^ the creation of organelle‐specific FπCM derivatives, such as FπCM‐SO_3_, is readily achievable. FπCM has the potential to revolutionize the application of solvatochromic probes because it can capture time‐resolved information of membrane order heterogeneity, which has not been obtainable from fragmented images with conventional probes or even with imaging mass spectroscopy. Investigating the correlation between membrane protein activation in response to extra/intracellular stimuli and spatiotemporal membrane fluidity transitions, an unexplored area, will shed light on the mechanisms underlying diverse membrane functions. Since live imaging with FπCM and organelle‐specific derivatives can easily be performed with conventional confocal microscopes, membrane order could become a standard, widely accessible information source for cell biologists, equivalent to membrane morphology.

## 4 Experimental Section

### Synthesis

All reagents were purchased from Tokyo Chemical Industries (TCI), Kanto Chemicals, and Sigma‐Aldrich. The details of the synthetic procedure are described in Supporting Information.

### Optical Measurements

UV–vis Absorption Spectroscopy spectra were recorded on a JASCO V‐670 UV–vis spectrophotometer. Absolute quantum yields were measured by a Hamamatsu Photonics Quantaurus QY apparatus. Fourier transform infrared (FT‐IR) spectra were recorded on a JASCO FT‐IR 469 plus spectrometer.

In photodegradation assays, a 0.1 µM solution of a given dye in a quartz micro‐cuvette (50 µL volume) was illuminated by 375, 360, and 405 nm light of Xenon lamp of a JASCO V‐670 UV–vis spectrophotometer (slits were open to 8 nm). During the time of illumination (10800 s), the illumination power density was ≈0.8 and 0.7 mW cm^−2^ for PK and FπCM and Laurdan, respectively, and the fluorescence at the maximum was recorded as a function of time.

### Cell Culture and Fluorescent Protein Expression

EpH4, HeLa, and HEK293T cells were cultured in Dulbecco's modified Eagle's medium (DMEM) supplemented with 10% fetal bovine serum. The coding sequence of human histone H2B and mCherry were cloned into the pLenti CMV Neo DEST vector (Plasmid #17392 from Addgene). HeLa cells stably expressing H2B‐mCherry were generated by lentiviral transduction followed by G418 (Wako Pure Chemical) selection.

### GUV Preparation

Phospholipids were purchased from Avanti Polar Lipids, and cholesterol was from Wako Pure Chemical. Giant unilamellar vesicles were prepared by gentle hydration as described previously (Zhao et al. 2007 BBA Biomembranes). Briefly, 250 nmol lipids containing 10% negatively charged PGs were dispensed into 13 mm screw cap test tubes, dried up with a rotary evaporator, and rehydrated with wet N_2_ gas at 60 °C. Hydrated lipid films were then swelled in 100 mM sucrose solution at 60 °C and gently cooled down to 25 °C for 10 h. GUVs were harvested into 100 mM glucose solution and observed in a simple chamber made of a coverslip and a slide with a silicon sheet spacer.

### Staining, Confocal Microscopy, and Image Analysis

Stock solutions of solvatochromic dyes were prepared in DMSO and used as 500–1000 times dilution. Cells cultured in glass‐bottom dishes were stained by incubation with the indicated concentration of solvatochromic dyes in Leibovitz's L‐15 medium supplemented with 10% FBS for 10 min at 37 °C. GUVs were stained in 100 mM glucose solution for 10 min at room temperature. All observation was performed with a confocal microscope (Carl Zeiss LSM900) equipped with Plan‐APO (63×/1.40 NA, oil immersion) objective. Images were acquired using Carl Zeiss Zen 3.4 software and analyzed using ImageJ/Fiji software. Dyes were excited with a 405 nm laser, and short (blue, 400‐x nm) and long (red, x‐750 nm) signals were separated with variable dichroic mirrors and simultaneously detected with two photomultipliers. Separation wavelengths “x” above were 465 nm for Laurdan, 560 nm for PK, and 500 nm for FπCM and FπCM‐SO_3_. For ratiometric image production, raw images were blurred with median filter of 3 pixels radius. For simultaneous observation of mCherry and solvatochromic dyes, images with 405 and 561 nm excitation were sequentially acquired for every optical section.

### Theoretical Calculations

DFT calculations were carried out with the Gaussian 09 and 16 program. The structures were optimized at the ωB97XD/6‐31G(d,p), ωB97XD/6‐311+G(d,p), and B3LYP/6‐311+G(d,p) level of theory. The frequency analysis was carried out for each optimized structure, giving no imaginary wavenumber. The excited states were optimized at the same calculation level as the ground state structure optimization. The calculated spectra and selected data are shown in Section S5 (Supporting Information) Solvent effects were included through the Integral Equation Formalism (IEF) version of the Polarizable Continuum Model (PCM) or solute electron density model (SMD). Relaxed excited state PESs have been obtained assuming a complete relaxation of the solvent polarization.

### Single Crystal X‐Ray Diffraction

Diffraction data were collected on a Rigaku R‐AXIS RAPID diffractometer using multi‐layer mirror monochromated CuKa radiation (*λ* = 1.54187 Å). The structure was solved by the direct method (SHELXT 2018/2) and refined by the full‐matrix least squares method (SHELXL 2018/3). Non‐hydrogen atoms were refined anisotropically. FπCM: CCDC2303310, FπCMo‐F: CCDC2303308, FπA: CCDC2303309, PCM: CCDC2303312, FstCMo‐F: CCDC2303311 contain the supplementary crystallographic data for this paper. These data can be obtained free of charge from The Cambridge Crystallographic Data Centre via www.ccdc.cam.ac.uk/data_request/cif.

## Conflict of Interest

T.T. and G.K. are inventors on a patent application (JP 2023‐083070) submitted by Tokyo Institute of Technology that covers the compounds and the synthetic methods included in this paper.

## Supporting information

Supporting Information

Supplemental Video 1

Supplemental Video 2

Supplemental Video 3

## Data Availability

The data that support the findings of this study are available from the corresponding author upon reasonable request.
