# Supplementary material for: Fluorescent Solvatochromic Probes for Long‐Term Imaging of Lipid Order in Living Cells
Source: Adv Sci (Weinh). 2024 Mar 11;11(17):2309721. doi: 10.1002/advs.202309721 (PMC11077641; doi:10.1002/advs.202309721)
Supplement: Supplementary file 1 — Supporting Information [file ADVS-11-2309721-s002.pdf]

ADVANCED SCIENCE
Open Access

Supporting Information

for *Adv. Sci.*, DOI 10.1002/advs.202309721

Fluorescent Solvatochromic Probes for Long-Term Imaging of Lipid Order in Living Cells

Takuya Tanaka, Atsushi Matsumoto, Andery S. Klymchenko, Eiji Tsurumaki, Junichi Ikenouchi and Gen-ichi Konishi**

Supporting Information

Fluorescent solvatochromic probes for long-term imaging of lipid order in living cells

Takuya Tanaka,[1] Atsushi Matsumoto,[2] Andery S. Klymchenko,[3] Eiji Tsurumaki,[4] Junichi Ikenouchi,*[2] Gen-ichi Konishi*[1]

[1] T. Tanaka, Prof. G. Konishi
Department of Chemical Science and Engineering
Tokyo Institute of Technology, Tokyo. 152-8552, Japan
E-mail: konishi.g.aa@m.titech.ac.jp

[2] Dr. A. Matsumoto, Prof. J. Ikenouchi
Department of Biology, Faculty of Sciences
Kyushu University, Fukuoka 819-0395, Japan
E-mail: ikenouchi.junichi.033@m.kyushu-u.ac.jp

[3] Dr. A. Klymchenko
Laboratoire de Bioimagerie et Pathologies, UMR 7021 CNRS
Université de Strasbourg, 74 route du Rhin, 67401 Illkirch, France

[4] Prof. E. Tsurumaki
Department of Chemistry
Tokyo Institute of Technology, Tokyo 152-8552, Japan

Abstract: High-resolution spatio-temporal monitoring of the cell membrane lipid order provides visual insights into the complex and sophisticated systems that control cellular physiological functions. Solvatochromic fluorescent probes are highly promising noninvasive visualization tools for identifying the ordering of the microenvironment of plasma membrane microdomains. However, conventional probes, although capable of structural analysis, lack the necessary long-term photostability required for live imaging at the cellular level. Here, we report an ultra-high-light-resistant solvatochromic fluorescence probe, 2-*N*,*N*-diethylamino-7-(4-methoxycarbonylphenyl)-9,9-dimethylfluorene (FπCM), which enables live lipid order imaging of cell division. This probe and its derivatives exhibit sufficient fluorescence wavelengths, brightness, polarity responsiveness, low phototoxicity, and remarkable photostability under physiological conditions compared to conventional solvatochromic probes. Therefore, these probes have the potential to overcome the limitations of fluorescence microscopy, particularly those associated with photobleaching. FπCM probes can serve as valuable tools for elucidating mechanisms of cellular processes at the bio-membrane level.

Table of contents.

S1. General Methods and Materials
S2. Synthesis and Characterization
S3. Optical properties
  S3-1. Photophysical properties
  S3-2. Photostability
  S3-4. Concentration dependence
  S3-5. Photophysical properties in solid-state and PMMA matrix
  S3-6. Lippert-Mataga plot analysis
S4. Crystallographic information
S5. Theoretical calculations
  S5-1. Optimization structure and Energy diagram
  S5-2. Summary of theoretical calculations for monomer
  S5-3. Intramolecular interaction energy and transitions probabilities for dimers
S6. Biological optical imaging
S7. Cartesian coordinates information
S8. References

S1. General Methods and Materials

Characterization

^{1}H NMR and ^{13}C NMR spectra were recorded on 500 MHz (125 MHz for ^{13}C NMR) BRUKER spectrometer or 100 MHz JEOL 400 spectrometers and $CDCl_3$ ($\delta(^{1}H)$ = 7.26 ppm, δ(13C) = 77.16 ppm) and DMSO ($\delta(^{1}H)$ = 2.50 ppm, $\delta(^{13}C)$ = 39.52 ppm) were used as solvent using tetramethyl silane (TMS) as the internal standard. The date for ^{1}H NMR was reported as follows: chemical shift (δ ppm), multiplicity (s = singlet, d = doublet, t = triplet, q = quartet, quin = quintet, sex = sextet m = multiple), integration, coupling constant (Hz). ^{13}C NMR spectra were reported as chemical shifts in ppm and multiplicity where appropriate. Column chromatography was carried out with Kanto Chemical silica gel 60N (40–50 mesh). TLC was carried out with Merck Silica Gel 60 F254 (0.2 mm) plate. Fourier transform infrared (FT-IR) spectra were recorded on a JASCO FT-IR 469 plus spectrometer with a universal Zn-Se ATR (attenuated total reflection) accessory in the 600-4000 cm^{-1}. Melting points (m.p.) were recorded on a Yanaco micro melting point apparatus. The mass spectrometry (MS) was performed on JEOL JMS700 mass spectrometer.

Optical measurements

Ultraviolet-Visible Absorption (UV-Vis) spectra were recorded on a JASCO V-670 UV-Vis spectrophotometer. Fluorescence spectra were recorded on a JASCO FP-6500 spectrofluorometer. The wavelengths obtained by the fluorescence spectrometer were converted to wavenumber using the equation $I = \lambda^2\ I(\lambda)$[4]. Absolute quantum yields were measured by a Hamamatsu Photonics Quantaurus QY apparatus. All photophysical measurements performed in solution were carried out using dilute solutions with optical densities (ODs) around 0.10 at the maximum absorption wavelength in 1 cm path-length quartz cells at room temperature (298 K). Fluorescence lifetimes were measured on a time correlated single photon counting system (HORIBA Fluoro Cube). The excitation light sources were LED pulse Lamps (Nano LED, 379 or 401 nm). In all samples, the time-to-amplitude converter ranges ware 50 ns, and the counts amounts were 10,000. Reptation rates were 1 MHz.

Cell Culture and fluorescent protein expression

EpH4, HeLa and HEK293T cells were cultured in Dulbecco's modified Eagle's medium (DMEM) supplemented with 10% fetal bovine serum. The coding sequence of human histone H2B and mCherry were cloned into the pLenti CMV Neo DEST vector (Plasmid #17392 from Addgene). HeLa cells stably expressing H2B-mCherry were generated by lentiviral transduction followed by G418 (Wako Pure Chemical) selection.

GUV preparation

Phospholipids were purchased from Avanti Polar Lipids, and cholesterol was from Wako Pure Chemical. Giant unilamellar vesicles were prepared by gentle hydration as described previously (Zhao et al. 2007 BBA Biomembranes). Briefly, 250 nmol lipids containing 10% negatively charged PGs were dispensed into 13 mm screw cap test tubes, dried up with a rotary evaporator, and rehydrated with wet N_2 gas at 60°C. Hydrated lipid films were then swelled in 100 mM sucrose solution at 60°C and gently cooled down to 25°C for 10 h. GUVs were harvested into 100 mM glucose solution and observed in a simple chamber made of a coverslip and a slide with silicon sheet spacer. Lipid composition

Staining, confocal microscopy and image analysis

Stock solutions of solvatochromic dyes were prepared in DMSO and used as 500-1000 times dilution. Cells cultured in glass-bottom dishes were stained by incubation with indicated concentration of solvatochromic dyes in Leibovitz's L-15 medium supplemented with 10% FBS for 10 min at 37°C. GUVs were stained in 100 mM glucose solution for 10 min at room temperature. All observation was performed with a confocal microscope (Carl Zeiss LSM900) equipped with Plan-APO (63×/1.40 NA, oil immersion) objective. Images were acquired using Carl Zeiss Zen 3.4 software and analyzed using ImageJ/Fiji software. Dyes were excited with a 405 nm laser, and short (blue, 400-x nm) and long (red, x-750 nm) signals were separated with variable dichroic mirror and simultaneously detected with two photomultipliers. Separation wavelengths 'x' above were 465 nm for Laurdan, 560 nm for PK and 500 nm for FπCM and FπCM-SO_3. For ratiometric images production, raw images were blurred with median filter of 3 pixels radius. For simultaneous observation of mCherry and solvatochromic dyes, images with 405 nm and 561 nm excitation were sequentially acquired for every optical section.

Theoretical calculations

DFT calculations were carried out with Gaussian 09 and 16 program.[S15,S16] The structures were optimized and the frequency analysis was carried out for each optimized structure, giving no imaginary wavenumber. The excited states were calculated by the time-dependent (TD)-DFT method (50-50, Nstate = 6). The calculated spectra and selected data are shown in Section S5 and S7. Solvent effects were included through the Integral Equation Formalism (IEF) version of the Polarizable Continuum Model (PCM). Relaxed excited state PESs have been obtained assuming a complete relaxation of the solvent polarization.

Single crystal X-ray diffraction

Diffraction data were collected on a Rigaku R-AXIS RAPID diffractometer using multi-layer mirror monochromated CuKa radiation (λ = 1.54187 Å). The structure was solved by the direct method (SHELXT 2018/2) and refined by the full-matrix least squares method (SHELXL 2018/3). Non-hydrogen atoms were refined anisotropically.

FπCM: CCDC2303310, FπCMo-F: CCDC2303308, FπA: CCDC2303309, PCM: CCDC2303312, FstCMo-F: CCDC2303311

Materials

Unless otherwise noted, all reagents and chemicals were as received without further purification. 2,7-dibromo-9,9-dimethylfluorene, Potassium *tert*-butoxide (KO^{t}Bu), (±)-2,2'-Bis(diphenylphosphino)-1,1'-binaphthyl (±)BINAP, Palladium(II) Acetate ($Pd(OAc)_2$, Piperidine, *p*-toluenesulfonic acid (*p*-TsOH), Methyl triphenyl phosphonium bromide, Methyl 4-bromo-2-fluorobenzoate, Triethylamine, 3-bromo-1-propanol, Thionyl chloride, *N*-octylamino-*N*-methylamine, 1,3-propanesultone, methyl 4-bromobenzoate, Bis(pinacolate)diborone, Potassium acetate and acrylic acid were obtained from TCI (Tokyo, Japan). Diethylamine, Sodium hydroxide, and spectrpgrade *n*-hexane, toluene, dichloromethane, THF, acetonitrile were purchased from Wako Pure Chem (Tokyo, Japan). Magunesium Sulfate Anhydrous ($MgSO_4$), [1,3-Bis(2,6-diisopropylphenyl)imidazol-2-ylidene](3-chloropyridyl)palladium(II) dichloride (Pd-PEPPSI-IPr), $Pd(dppf)Cl_2CH_2Cl_2$ and tri(*o*-tolyl)phosphine were obtained from Sigma-Aldrich Japan (Tokyo, Japan). Dehydrated THF, 4-(methoxycarbonyl)phenylboronic acid, Potassium phosphate hydrate, Tetrakis(triphenylphosphine)palladium (0) ($Pd(PPh_3)_4$, 3-fluoro-4-(methoxycarbonyl)phenylboronic acid, 4-acetylphenylboronic acid, 2.6M *n*-butyl lithium,

hydrochloric acid, Ammonium chloride (NH_4Cl) and spectrograde pure water were purchased from Kanto Chem (Tokyo, Japan).

S2. Synthesis and Characterization

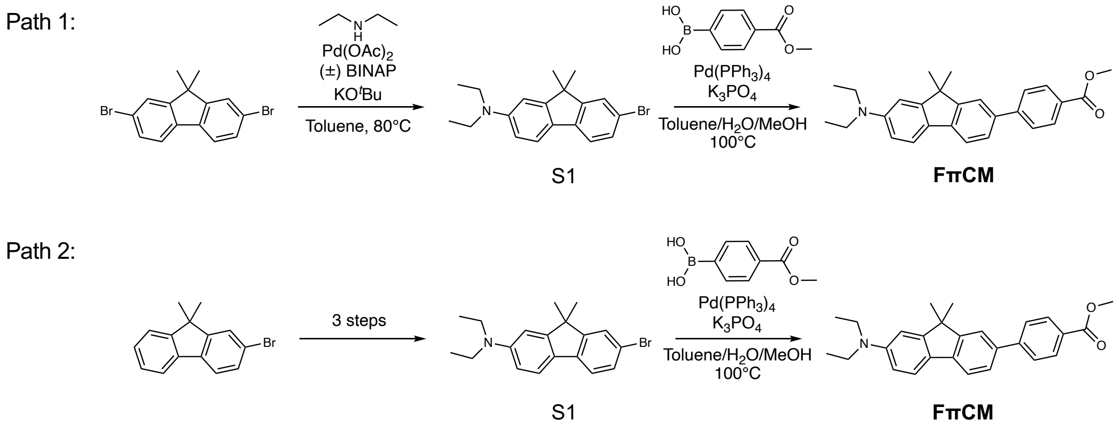

Figure S1. Synthetic rout to **FπCM**.

The starting material S1 in Path 2 was synthesized adapting published protocols.[S1]

7-bromo-2-*N,N*-diethylamino-9,9-dimethylfluorene (S1). A mixture of 2,7-dibromo-9,9-dimethylfluorene (1.0 eq, 5.0 mmol, 1.76 g), KO^tBu (1.5 eq, 0.84 g, 7.50 mmol), diethyllamine (1.0 eq, 0.37 mL, 5.0 mmol), (±)BINAP (10 mol%, 0.31 g, 0.50 mmol) and $Pd(OAc)_2$ (5.0 mol%, 0.056 g, 0.25 mmol) was dissolved in dehydrated toluene (30 mL) and stirred for 1h at 100°C. After cooling to room temperature, the reaction mixture was quenched with water, extracted with dichloromethane, and washed with water and brine. The combined organic layers were dried with $MgSO_4$, filtered, and then evaporated under reduced pressure to give a residue. Purification by silica gel column chromatography (eluent: hexane/ethyl acetate = 5/1 (v/v)) yielded **S1** as a colorless solid (1.03 g, 60% yield). **^{1}H NMR** (500 MHz, $CDCl_3$) δ7.50 (d, *J* = 8.2 Hz, 1H, Ar**H**), 7.44 (s, 1H, Ar**H**), 7.39 (d, *J* = 7.9 Hz, 1H, Ar**H**), 7.36 (d, *J* = 8.2 Hz, 1H, Ar**H**), 6.67 (s, 1H, Ar**H**), 6.66-6.64 (m, 1H, Ar**H**), 3.41 (q, *J* = 7.0 Hz, 4H, NC**H**$_2$), 1.44 (s, 6H, C**H**$_3$), 1.20 (t, *J* = 7.0 Hz, 6H, C**H**$_3$) ppm. (**Figure S2**)

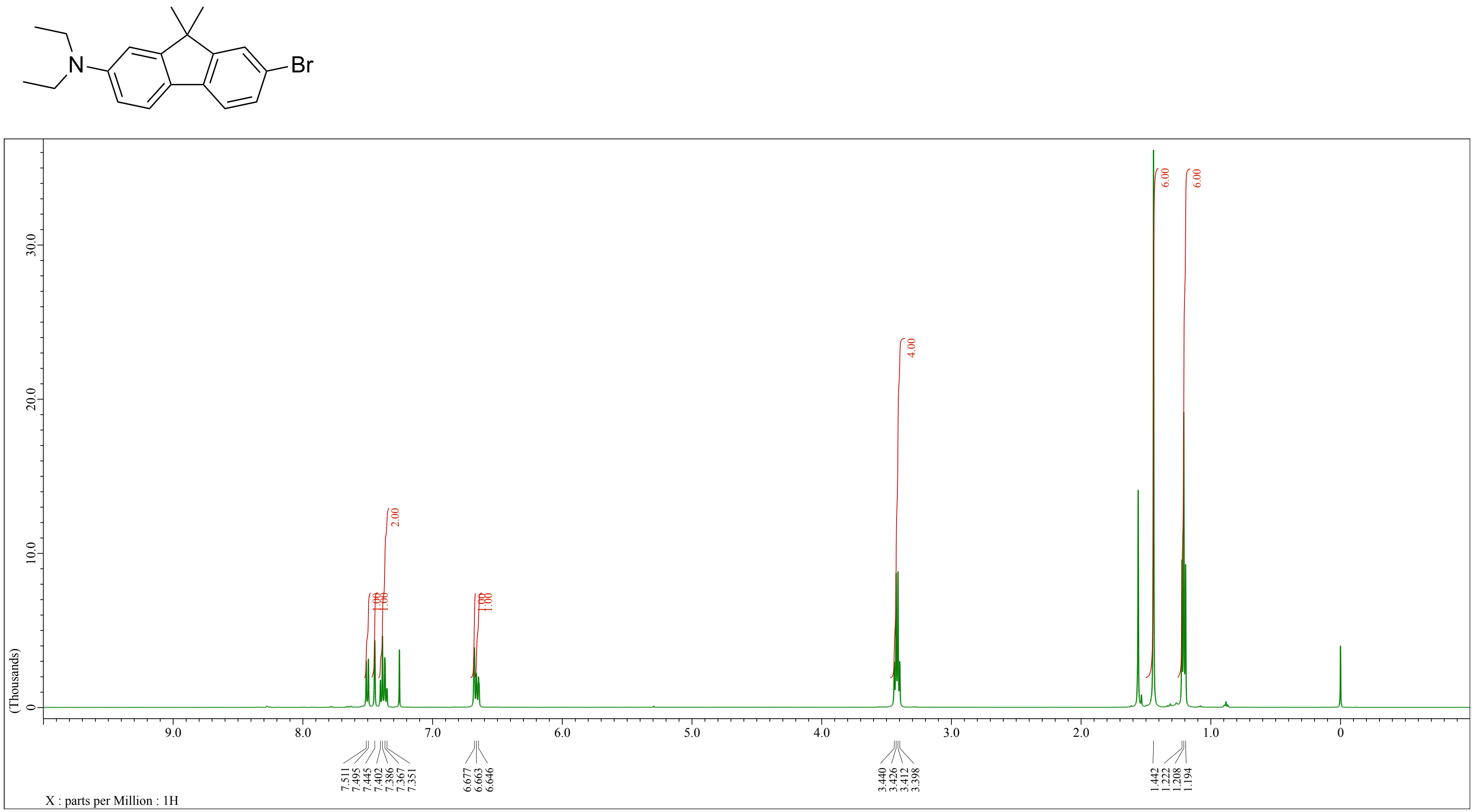

Figure S2. ^{1}H NMR (500 MHz, $CDCl_3$) spectrum of S1

methyl 4-(7-(diethylamino)-9,9-dimethyl-*9H*-fluoren-2-yl) benzoate (FπCM). A mixture of **S1** (1.0 eq, 3.0 mmol, 1.03 g), 4-(methoxycarbonyl)phenylboronic acid (1.5 eq, 4.5 mmol, 0.81 g), potassium phosphate hydrate (3.0 eq, 9.0 mmol, 1.90 g) and $Pd(PPh_3)_4$ (5.0 mol%, 0.15 mmol, 0.17 g) was dissolved in 5/1/2 (v/v/v) solution of toluene, high purity water and MeOH under argon atmosphere. The reaction mixture was heated to 100°C and stirred for 6 hours, and then cooled to room temperature. The reaction mixture was quenched with water. The organic products were extracted with dichloromethane, and the organic layer was washed with water and brine. The combined organic layers were dried with $MgSO_4$, filtered, and then evaporated under reduced pressure to give a residue. Purification by silica gel column chromatography (eluent: hexane/ethyl acetate = 5/1 (v/v)) and recrystallization from hexane/dichloromethane (5/1) yielded **FπCM** as a yellow solid (0.93 g, 78% yield). **^{1}H NMR** (500 MHz, $CDCl_3$) δ8.10 (d, *J* = 8.2 Hz, 2H, Ar**H**), 7.72 (d, *J* = 8.2 Hz, 2H, Ar**H**), 7.63-7.54 (m, 4H, Ar**H**), 6.73 (d, *J* = 1.5 Hz, 1H, Ar**H**), 6.69 (d, *J* = 8.5 Hz, 1H, Ar**H**), 3.94 (s, 3H, OC**H**$_3$), 3.44 (q, *J* = 7.0 Hz, 4H, NC**H**$_2$), 1.52 (s, 6H, C**H**$_3$), 1.23 (t, *J* = 7.0 Hz, 6H, C**H**$_3$) ppm. (**Figures S3 and S4**) **^{13}C NMR** (100 MHz, $CDCl_3$) δ 167.3, 156.0, 153.6, 148.2, 146.5, 140.5, 136.6, 130.2, 128.3, 126.9, 126.5, 126.3, 121.3, 121.2, 118.8, 110.9, 105.8, 52.2, 46.9, 44.8, 27.7, 12.8 ppm. (**Figure S5**) **HRMS** (EI+) m/z Calcd. For $C_{27}H_{29}NO_2$ $[M]^+$:399.2198 Found: 399.2200 (**Figure S6**) **FT-IR** (**Figure S7**) **m.p.**: 164.1-164.3°C.

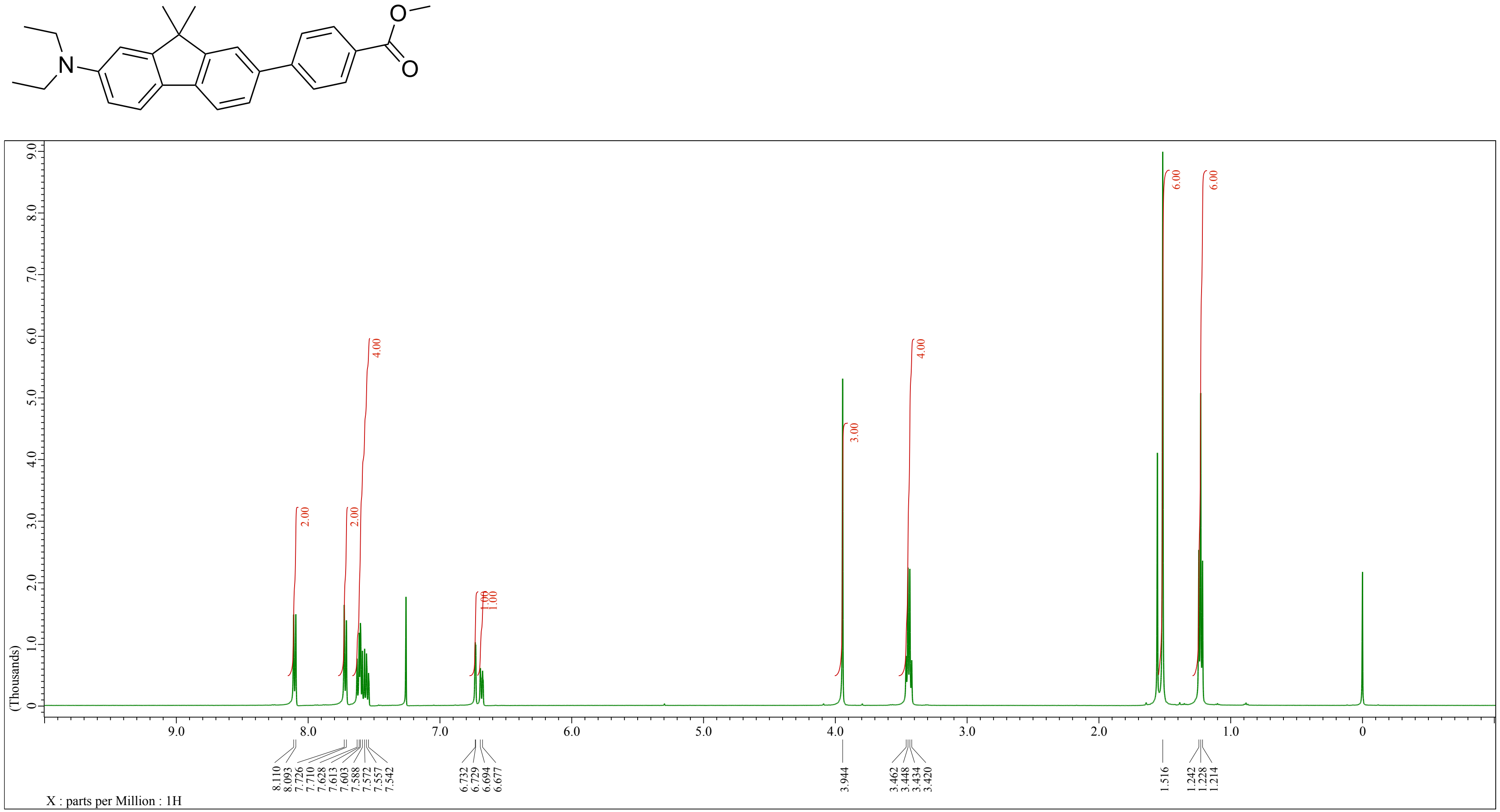

Figure S3. ^{1}H NMR (500 MHz, $CDCl_3$) spectrum of **FπCM**.

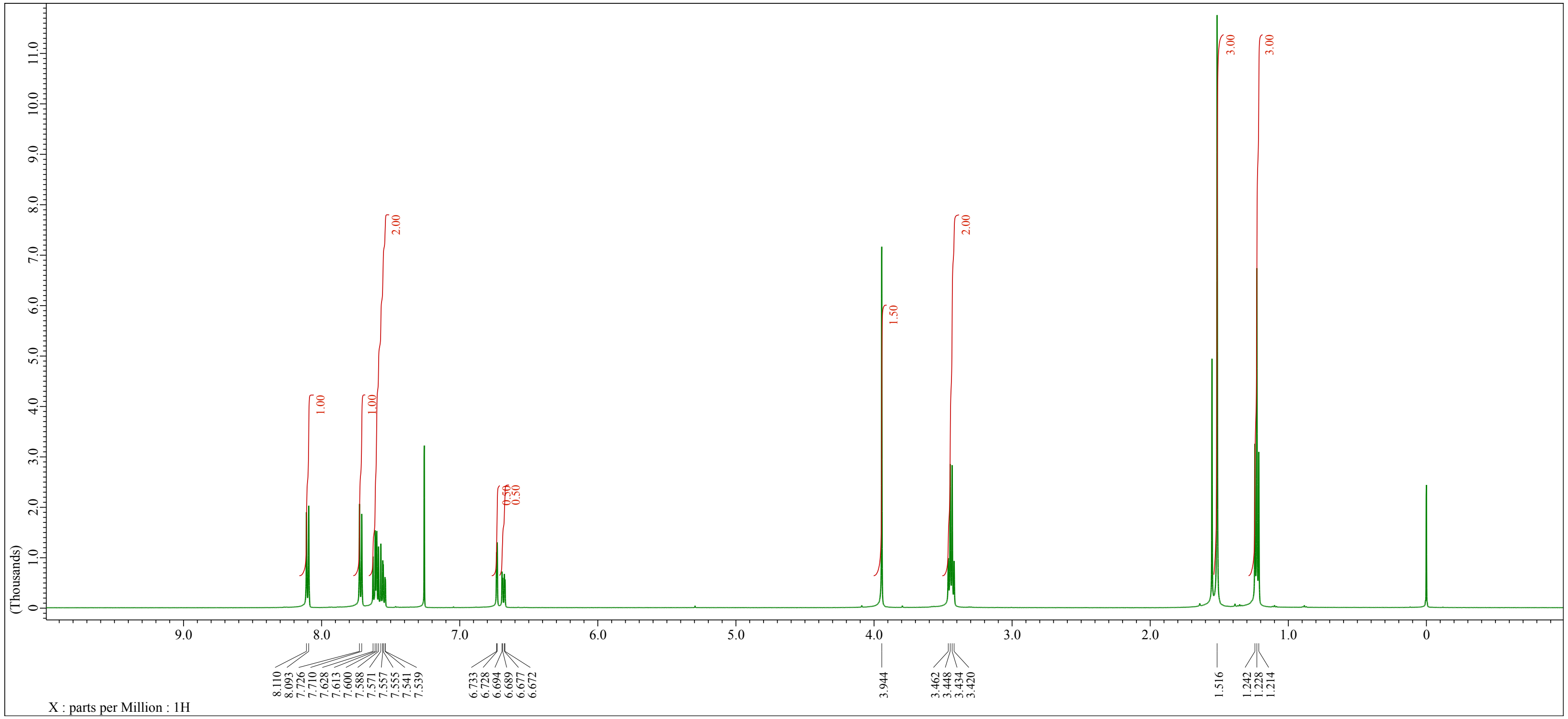

Figure S4. ^{1}H NMR (500 MHz, $CDCl_3$) spectrum of **FπCM** after 12 months in storage at room temperature under oxygen atmosphere from purification.

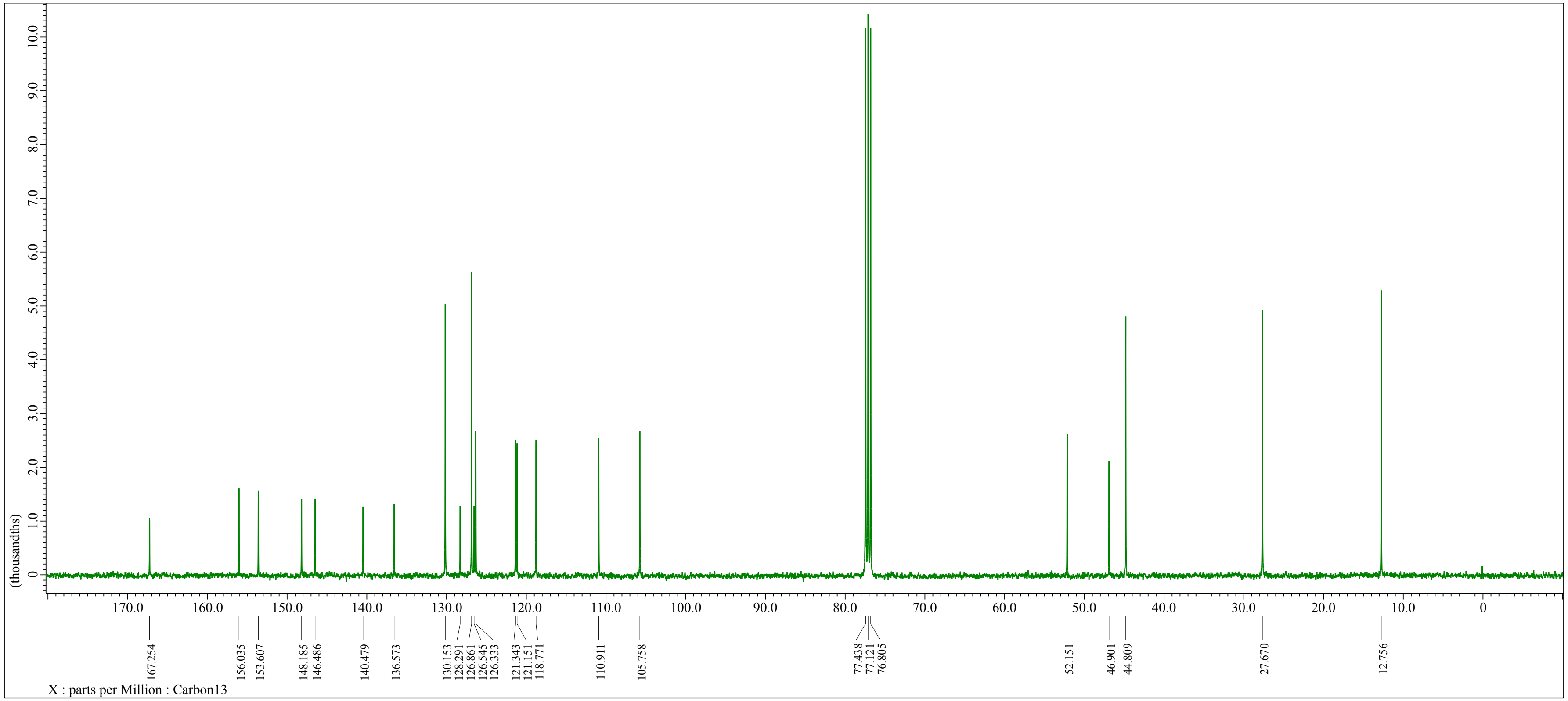

Figure S5. ^{13}C NMR (100 MHz, $CDCl_3$) spectrum of **FπCM**.

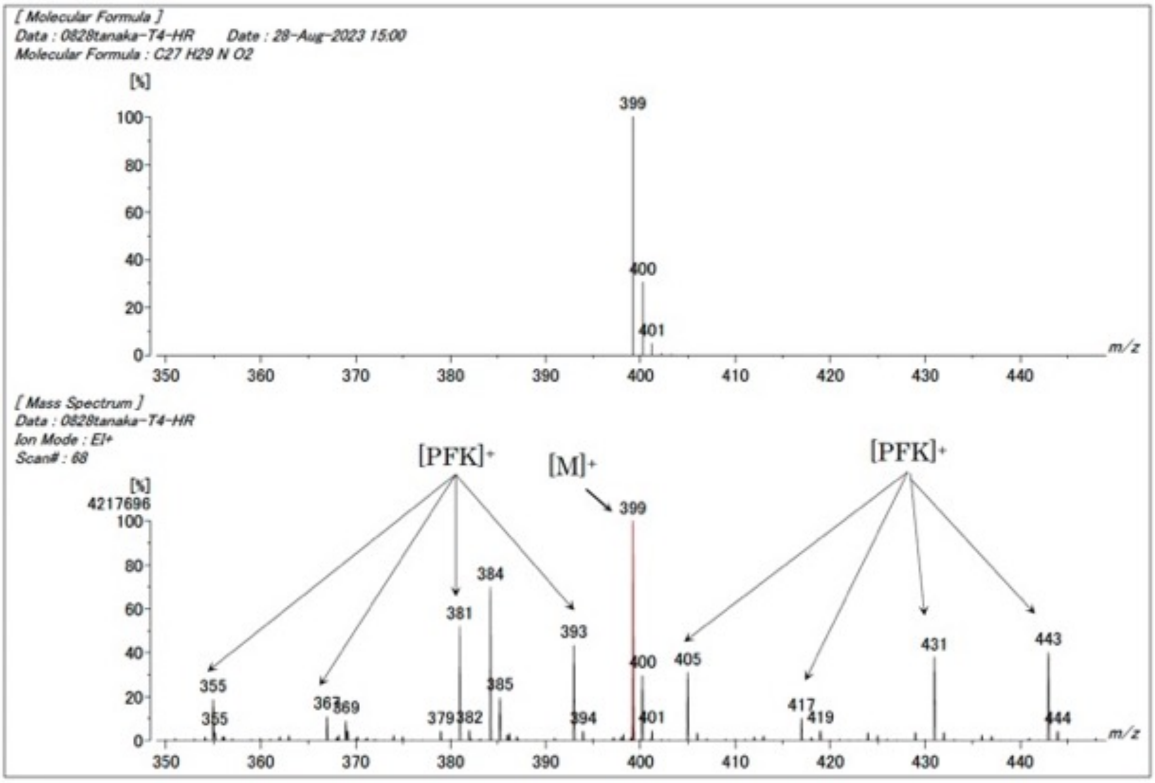

	Observed m/z	Int%	Err[ppm / mmu]	U.S.	Composition
1	399.2200	100.00	+3.8 / +1.5	14.5	C25 H27 N4 O
2			+0.4 / +0.2	14.0	C27 H29 N O2 ←
3			-4.2 / -1.7	6.5	C14 H27 N10 O4
4			+2.5 / +1.0	2.0	C11 H29 N9 O7
5			-0.8 / -0.3	1.5	C13 H31 N6 O8
6			-4.2 / -1.7	1.0	C15 H33 N3 O9

Figure S6. MS spectrum of **FπCM**.

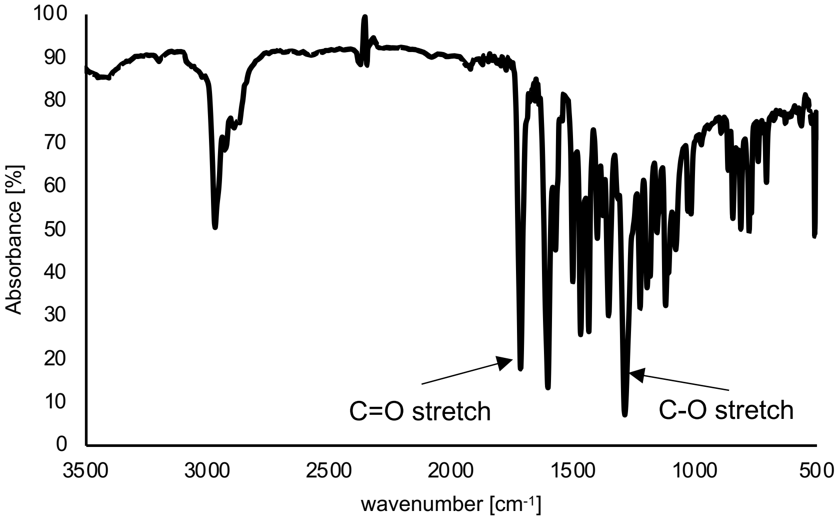

Figure S7. FT-IR spectrum of **FπCM**.

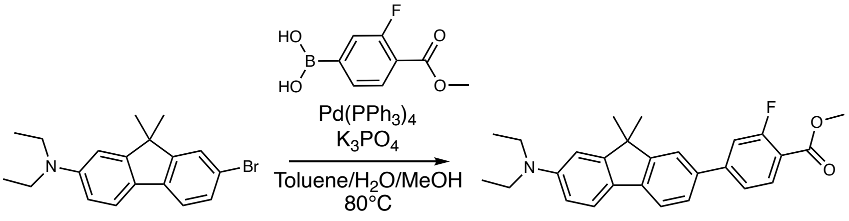

S1 **FπCM*o*-F**

Figure S8. Synthetic rout to **FπCM*o*-F**.

methyl 4-(7-(diethylamino)-9,9-dimethyl-9*H*-fluoren-2-yl)-2-fluorobenzoate (FπCM*o*-F). A mixture of **S1** (1.0 eq, 5.0 mmol, 1.72 g), 3-Fluoro-4-(methoxycarbonyl)phenylboronic Acid (1.5 eq, 7.5 mmol, 1.48 g), potassium phosphate hydrate (3.0 eq, 15 mmol, 3.36 g) and $Pd(PPh_3)_4$ (5.0 mol%, 0.25 mmol, 0.20 g) was dissolved in 5/1/2 (v/v/v) solution of toluene, high purity water and MeOH under argon atmosphere. The reaction mixture was heated to 80°C and stirred for 6 hours, and then cooled to room temperature. The reaction mixture was quenched with water. The organic products were extracted with dichloromethane, and the organic layer was washed with water and brine. The combined organic layers were dried with $MgSO_4$, filtered, and then evaporated under reduced pressure to give a residue. Purification by silica gel column chromatography (eluent: hexane/ethyl acetate = 5/1 (v/v)) and recrystallization from hexane/dichloromethane (5/1) yielded **FπCM*o*-F** as a green solid (1.46 g, 70% yield). **^{1}H NMR** (500 MHz, $CDCl_3$) δ8.00 (t, J = 7.8 Hz, 1H, Ar**H**), 7.62 (d, J = 7.6 Hz, 1H, Ar**H**), 7.58 (d, J = 7.9 Hz, 2H, Ar**H**), 7.53 (d, J = 7.9 Hz, 1H, Ar**H**), 7.49 (d, J = 8.2 Hz, 1H, Ar**H**), 7.42 (d, J = 12.5 Hz, 1H, Ar**H**), 6.72 (s, 1H, Ar**H**), 6.68 (d, J = 8.2 Hz, 1H, Ar**H**), 3.96 (s, 3H, OC**H**$_3$), 3.44 (q, J = 7.0 Hz, 4H, NC**H**$_2$), 1.51 (s, 6H, C**H**$_3$), 1.23 (t, J = 7.0 Hz, 6H, C**H**$_3$) ppm. (**Figure S9**) **^{13}C NMR** (100 MHz, CDCl3) δ 165.1, 163.8, 161.2, 156.1, 153.7, 148.8, 148.7, 148.3, 141.1, 135.1, 132.6, 126.3, 122.3, 122.2, 121.5, 121.0, 118.8, 116.3, 116.2, 115.0, 114.8, 110.9, 105.7, 52.3, 46.9, 44.8, 27.6, 12.7 ppm. (**Figure S10**) The number of C atoms of ^{13}C NMR for **FstCMo-F** (13C-NMR data) is more than the theoretical data, because F atoms can cause splits of C atoms in ^{13}C NMR spectrum.[S2] **HRMS** (EI+) m/z Calcd. For $C_{27}H_{28}FNO_2$ $[M]^+$: 417.2104, Found: 417.2105. (**Figure S11**) **FT-IR** (**Figure S12**) **m.p.**: 128.6-129.8°C

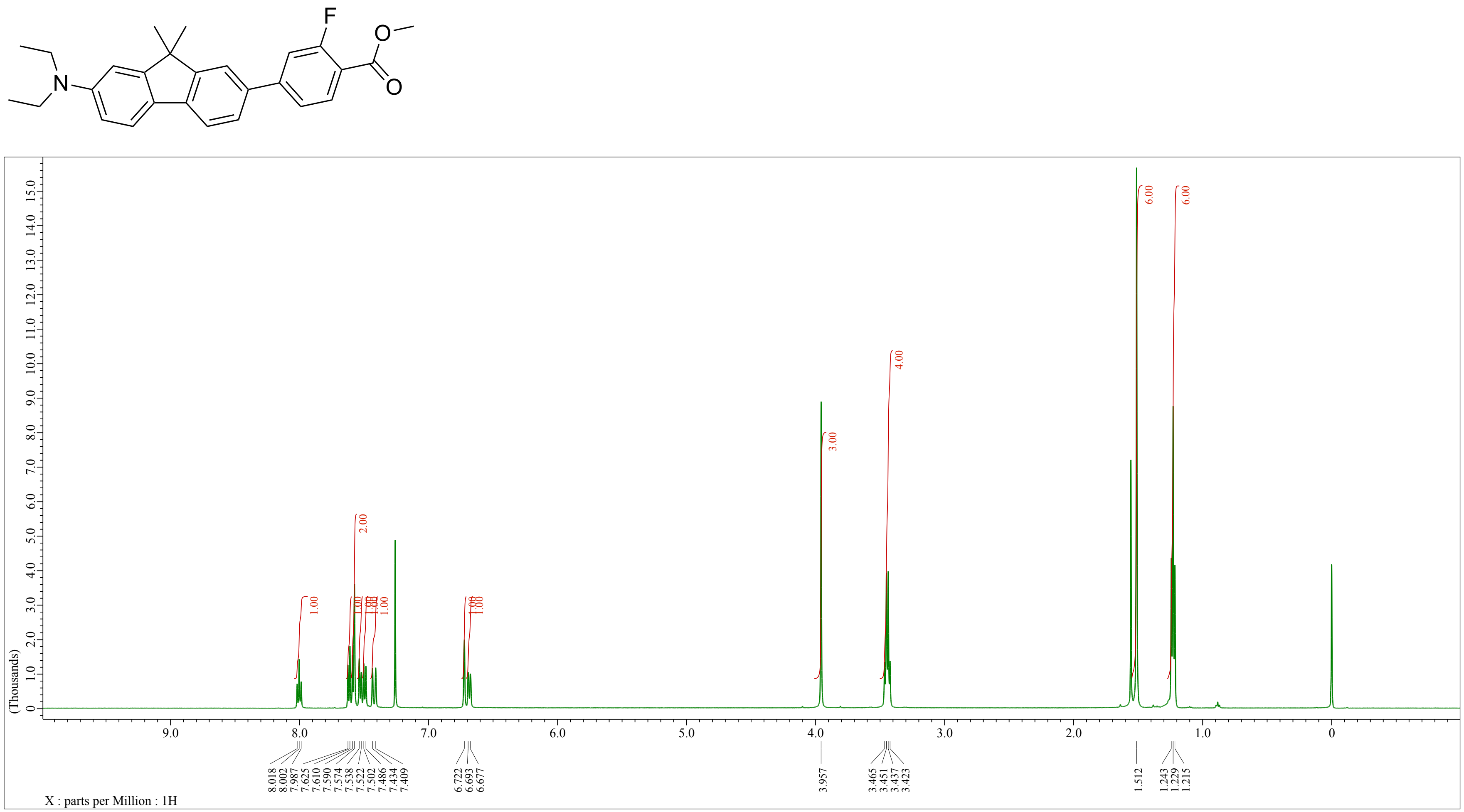

Figure S9. ^{1}H NMR (500 MHz, $CDCl_3$) spectrum of **FπCM*o*-F**.

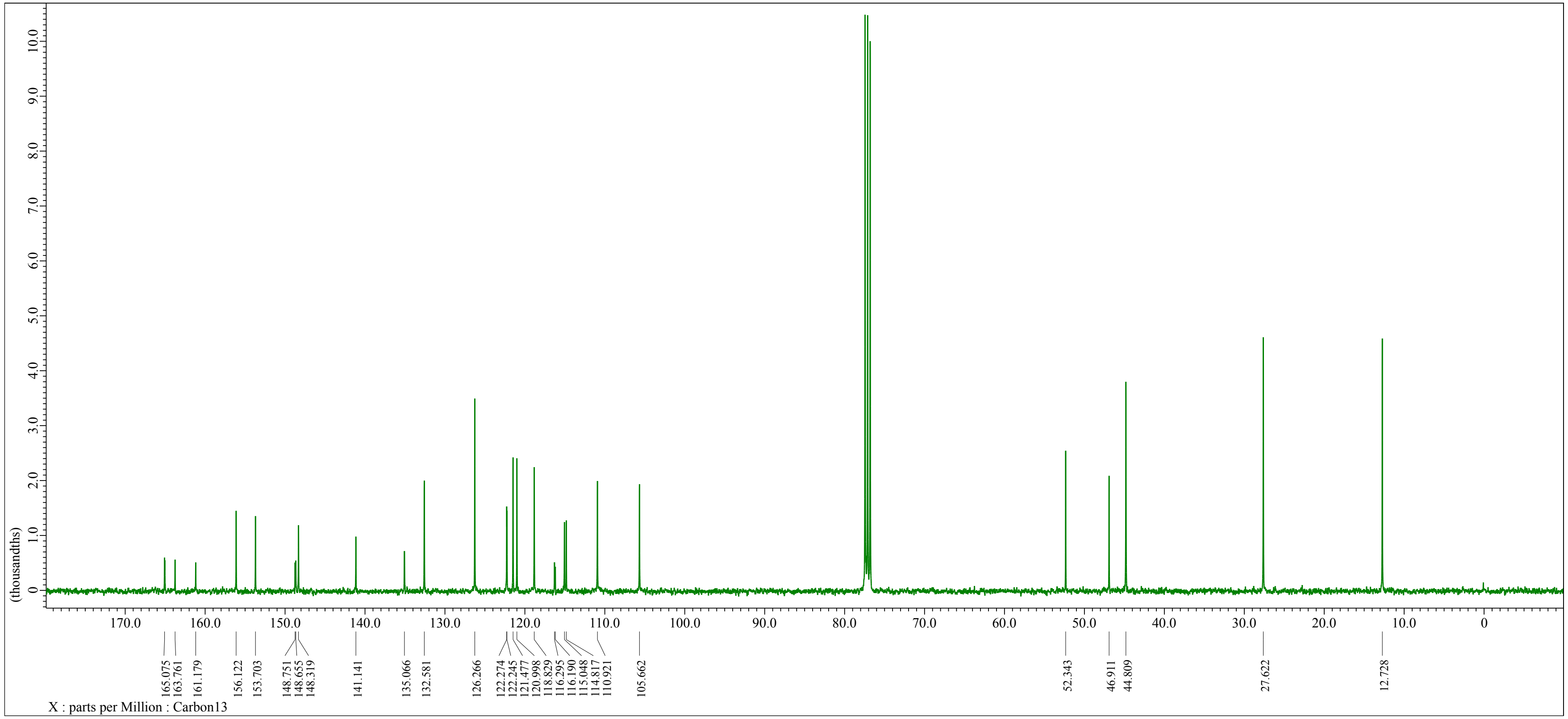

Figure S10. ^{13}C NMR (100 MHz, $CDCl_3$) spectrum of **FπCM*o*-F**.

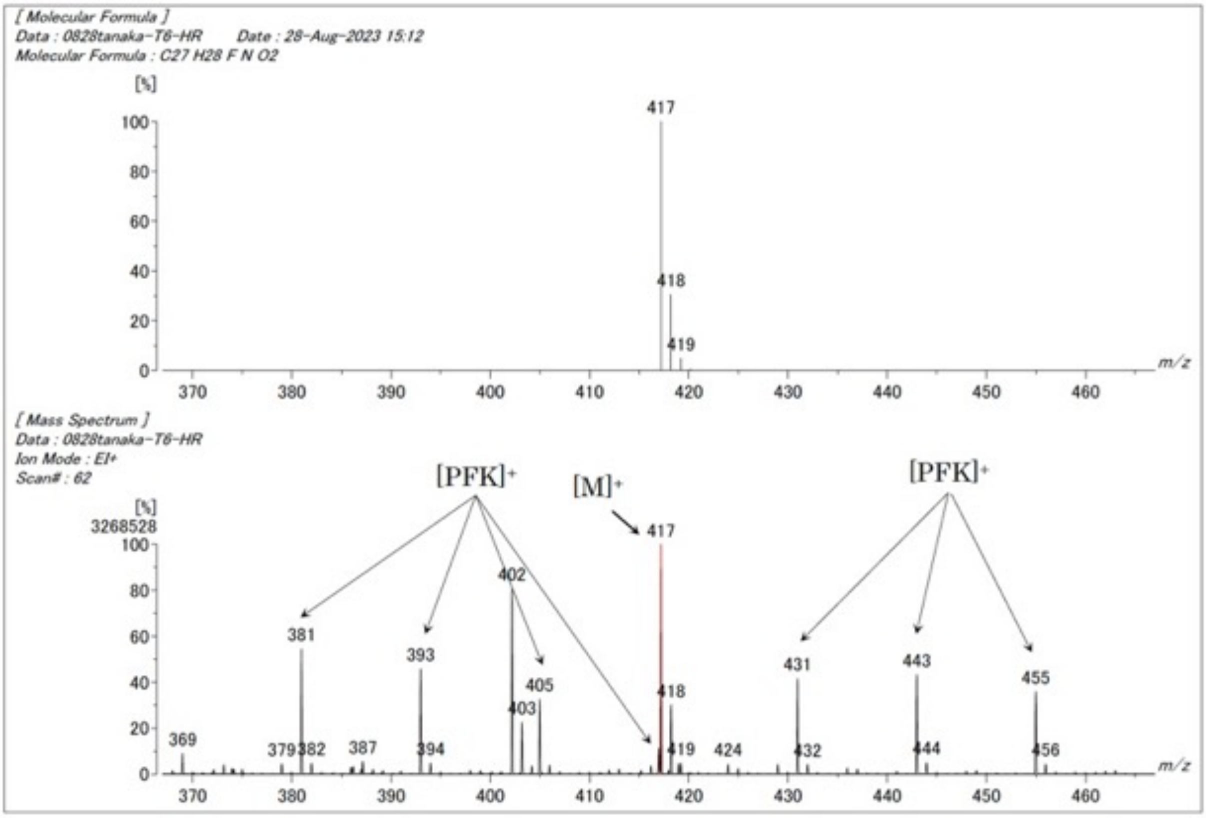

Observed m/z	Int%	Err[ppm / mmu]	U.S.	Composition
1 417.2105	100.00	+3.4 / +1.4	14.5	C25 H26 F N4 O
2		+0.2 / +0.1	14.0	C27 H28 F N O2 ←
3		-4.2 / -1.8	6.5	C14 H26 F N10 O4
4		+2.2 / +0.9	2.0	C11 H28 F N9 O7
5		-1.0 / -0.4	1.5	C13 H30 F N6 O8
6		-4.2 / -1.8	1.0	C15 H32 F N3 O9

Figure S11. MS spectrum of **FπCM*o*-F**.

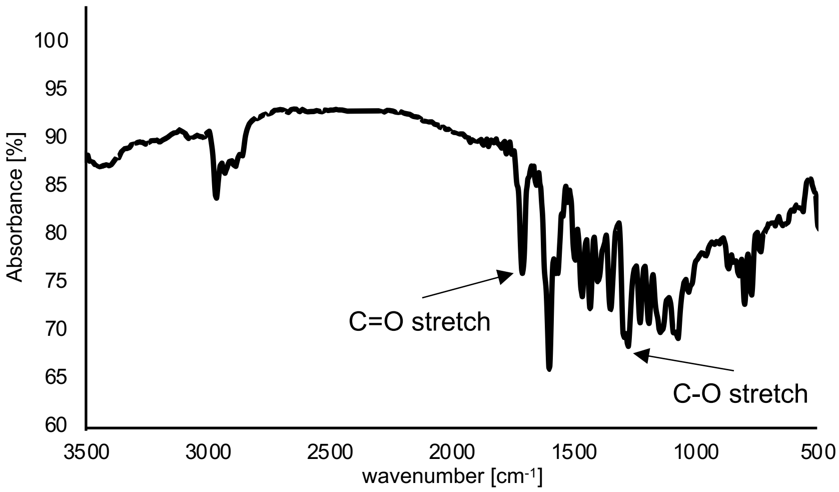

Figure S12. FT-IR spectrum of **FπCM*o*-F**.

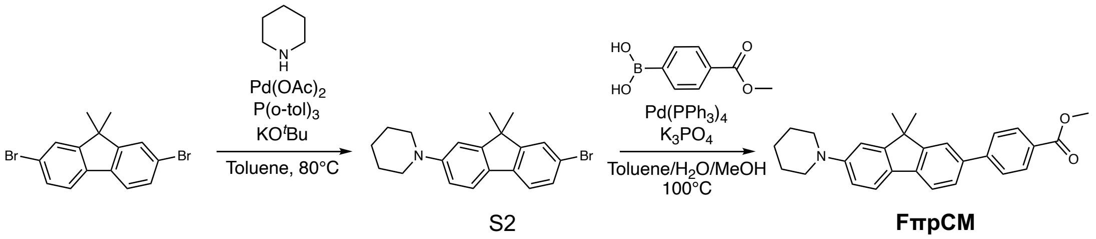

Figure S13. Synthetic rout to **FπpCM**.

7-bromo-2-*piperidyl*-9,9-dimethylfluorene (S2). A mixture of 2,7-dibromo-9,9-dimethylfluorene (1.0 eq, 5.0 mmol, 1.76 g), KO^{t}Bu (1.5 eq, 0.84 g, 7.50 mmol), piperidine (1.0 eq, 0.43 mL, 5.0 mmol), (±)BINAP (10 mol%, 0.31 g, 0.50 mmol) and $Pd(OAc)_2$ (5.0 mol%, 0.056 g, 0.25 mmol) was dissolved in dehydrated toluene (30 mL) and stirred for 4h at 100°C. After cooling to room temperature, the reaction mixture was quenched with water, extracted with dichloromethane, and washed with water and brine. The combined organic layers were dried with $MgSO_4$, filtered, and then evaporated under reduced pressure to give a residue. Purification by silica gel column chromatography (eluent: hexane/ethyl acetate = 5/1 (v/v)) yielded **S2** as a colorless solid (0.85 g, 48% yield). **^{1}H NMR** (500 MHz, $CDCl_3$) δ 7.54 (d, J = 8.2 Hz, 1H, Ar**H**), 7.48 (s, 1H, Ar**H**), 7.44 (d, J = 7.9 Hz, 1H, Ar**H**), 7.39 (d, J = 7.9 Hz, 1H, Ar**H**), 6.98 (s, 1H, Ar**H**), 6.91 (d, J = 7.6 Hz, 1H, Ar**H**), 3.22 (t, J = 5.2 Hz, 4H, NC**H**$_2$), 1.75 (s, 4H, C**H**$_2$), 1.61 (t, J = 5.8 Hz, 2H, C**H**$_2$), 1.44 (s, 6H, C**H**$_3$) ppm. (**Figure S14)**

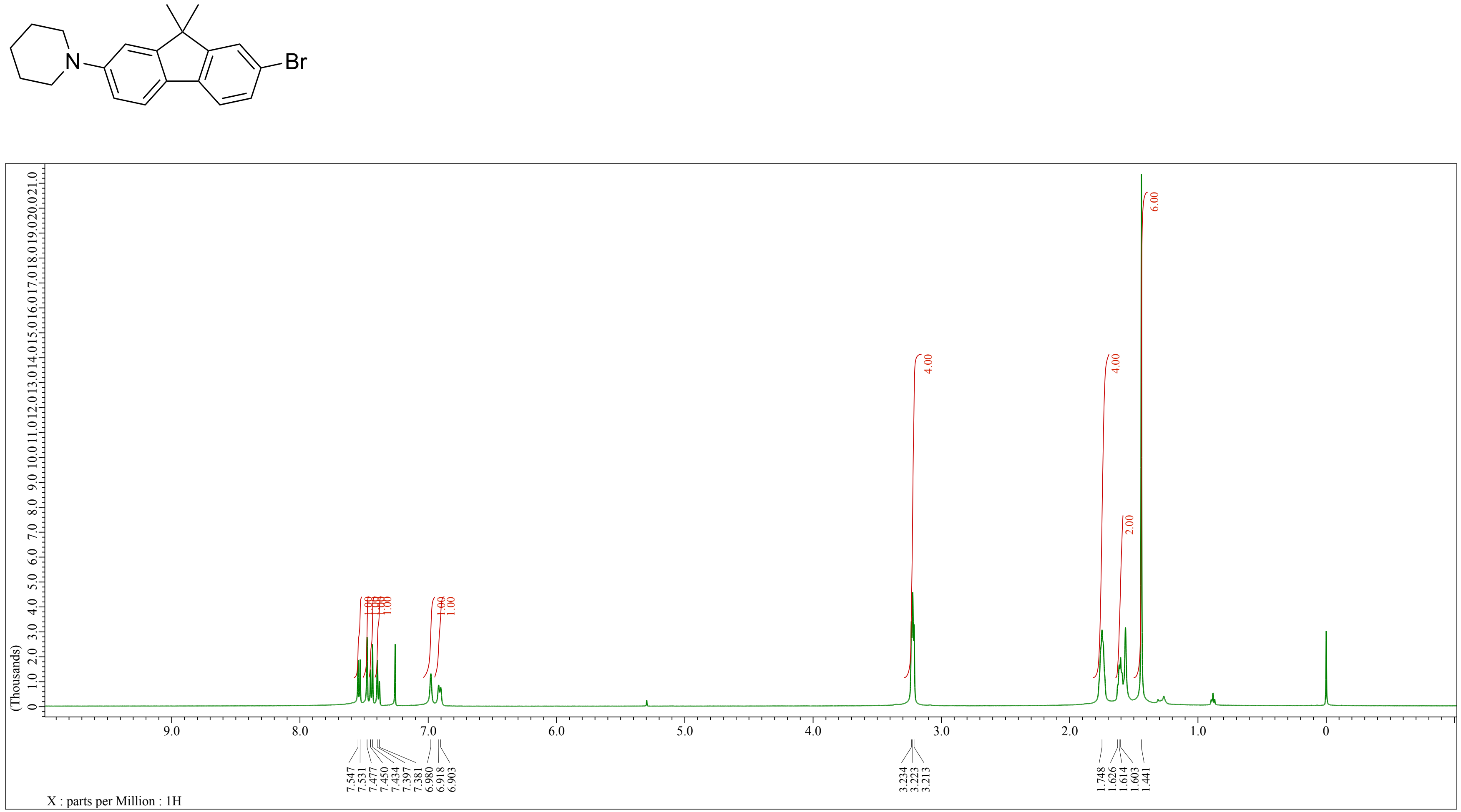

Figure S14. ^{1}H NMR (500 MHz, $CDCl_3$) spectrum of **S2**.

methyl 4-(9,9-dimethyl-7-(piperidin-1-yl)-*9H*-fluoren-2-yl)benzoate (FπpCM). A mixture of **S2** (1.0 eq, 2.40 mmol, 0.85 g), 4-(methoxycarbonyl)phenylboronic acid (1.5 eq, 3.60 mmol, 0.65 g), potassium phosphate hydrate (3.0 eq, 7.2 mmol, 1.76 g) and $Pd(PPh_3)_4$ (5.0 mol%, 0.12 mmol, 0.14 g) was dissolved in 5/1/2 (v/v/v) solution of toluene, high purity water and MeOH under argon atmosphere. The reaction mixture was heated to 100°C and stirred for 6 hours, and then cooled to room temperature. The reaction mixture was quenched with water. The organic products were extracted with dichloromethane, and the organic layer was washed with water and brine. The combined organic layers were dried with $MgSO_4$, filtered, and then evaporated under reduced pressure to give a residue. Purification by silica gel column chromatography (eluent: hexane/ethyl acetate = 5/1 (v/v)) and recrystallization from hexane/dichloromethane (5/1) yielded **FπpCM** as a yellow solid (0.69 g, 70% yield). **^{1}H NMR** (500 MHz, $CDCl_3$) δ8.11 (d, *J* = 7.9 Hz, 2H, Ar**H**), 7.72 (d, *J* = 8.2 Hz, 2H, Ar**H**), 7.67 (d, *J* = 7.9 Hz, 1H, Ar**H**), 7.61 (d, *J* = 7.6 Hz, 2H, Ar**H**), 7.56 (d, *J* = 7.9 Hz, 1H, Ar**H**), 7.02 (s, 1H, Ar**H**), 6.94 (d, *J* = 8.2 Hz, 1H, Ar**H**), 3.95 (s, 3H, OC**H**$_3$), 3.25 (t, *J* = 5.3 Hz, 4H, NC**H**$_2$), 1.78-1.74 (m, 4H, C**H**$_2$), 1.64-1.59 (m, 2H, C**H**$_2$), 1.51 (s, 6H, C**H**$_3$) ppm. (**Figure S15**) **^{13}C NMR** (100 MHz, $CDCl_3$) δ 167.2, 155.4, 154.1, 152.6, 146.4, 140.0, 137.4, 130.2, 128.5, 127.0, 126.4, 121.3, 120.9, 119.4, 115.5, 110.9, 52.2, 51.1, 47.0, 27.6, 26.1, 24.4 ppm. (**Figure S16**) **HRMS** (EI+) m/z Calcd. For $C_{28}H_{29}NO_2$ $[M]^+$: 411.2198, Found: 411.2198. (**Figure S17**) **FT-IR** (**Figure S18**) **m.p.**: 176.7-177.4°C.

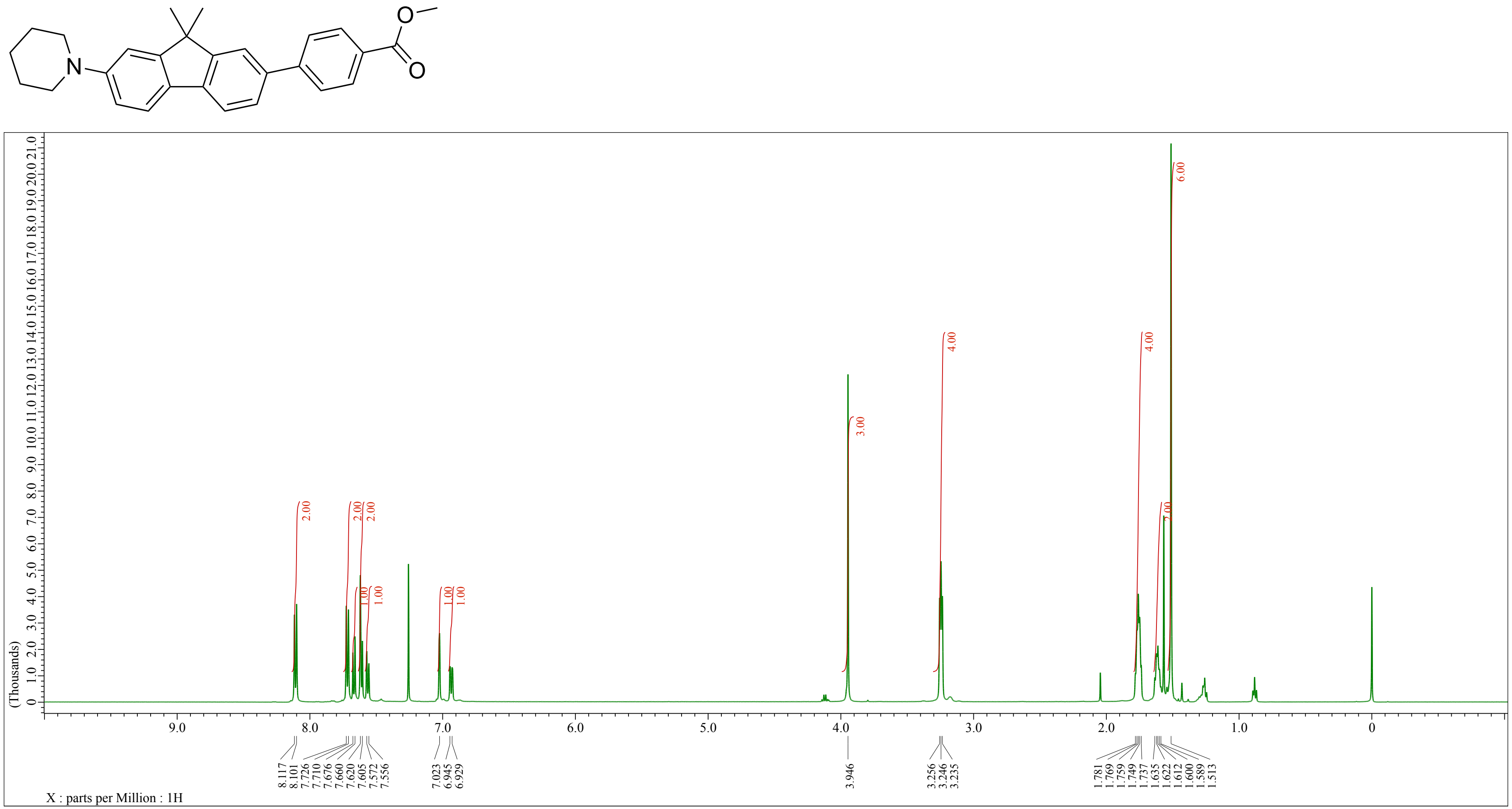

Figure S15. ^{1}H NMR (500 MHz, $CDCl_3$) spectrum of **FπpCM**.

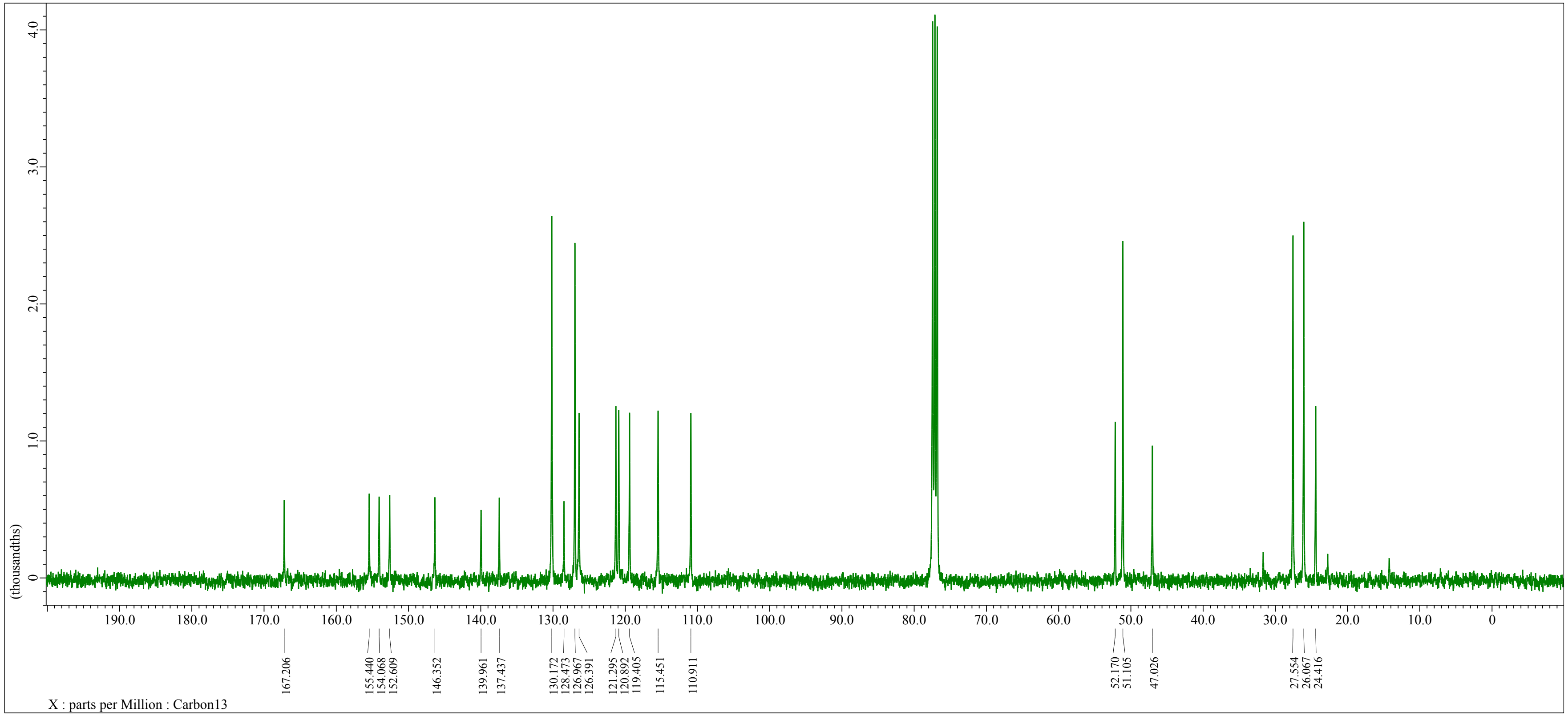

Figure S16. ^{13}C NMR (100 MHz, $CDCl_3$) spectrum of **FπpCM**.

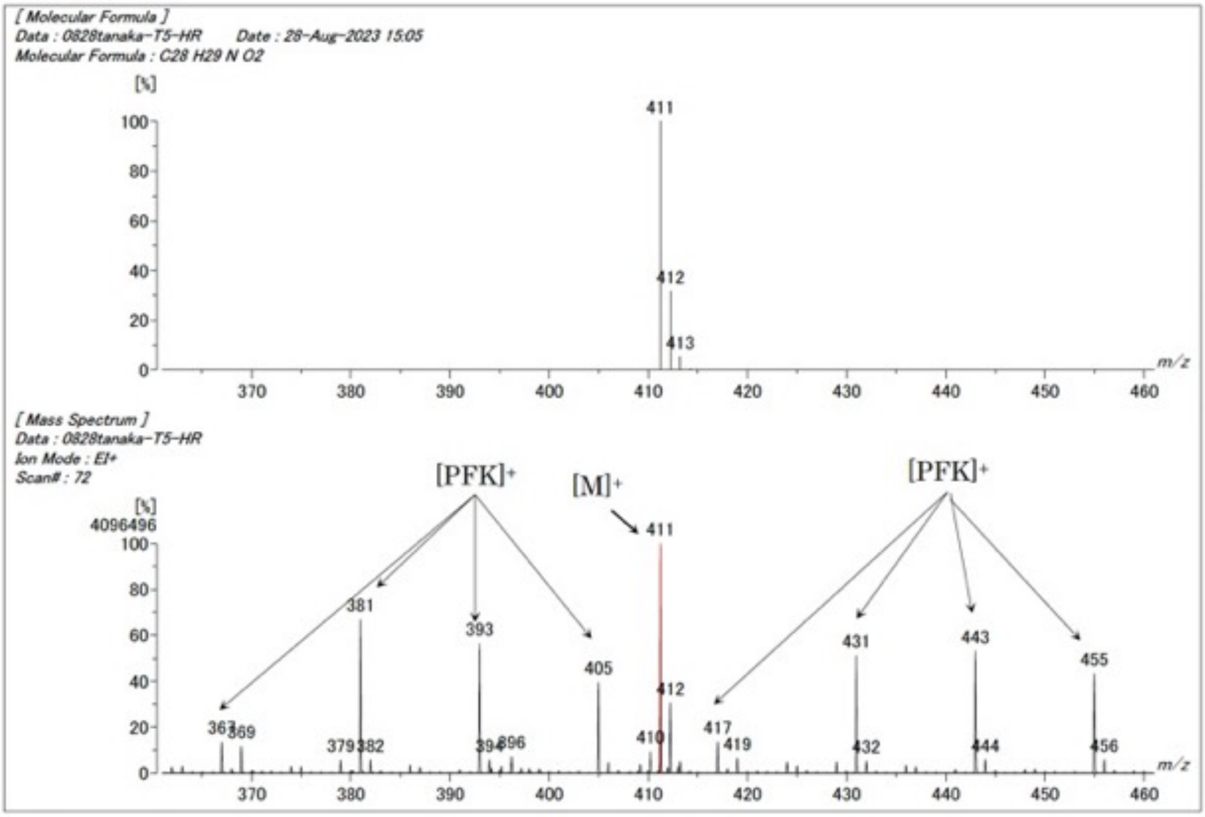

Observed m/z	Int%	Err[ppm / mmu]	U.S.	Composition
1 411.2197	100.00	+3.0 / +1.2	15.5	C26 H27 N4 O
2		-0.3 / -0.1	15.0	C28 H29 N O2 ←
3		-4.8 / -2.0	7.5	C15 H27 N10 O4
4		+1.7 / +0.7	3.0	C12 H29 N9 O7
5		-1.5 / -0.6	2.5	C14 H31 N6 O8
6		-4.8 / -2.0	2.0	C16 H33 N3 O9

Figure S17. MS spectrum of **FπpCM**.

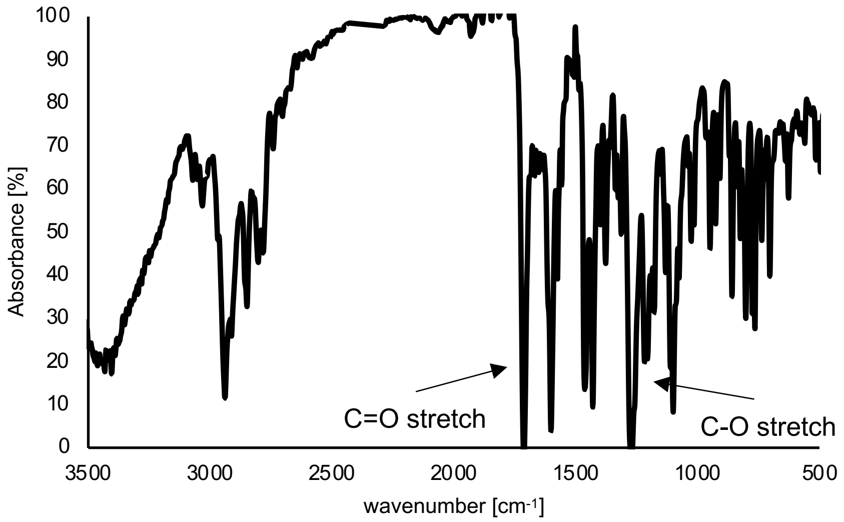

Figure S18. FT-IR spectrum of **FπpCM**.

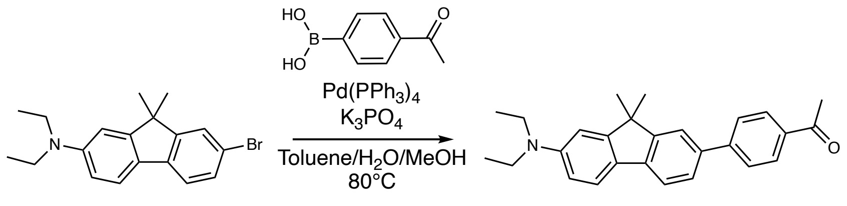

Figure S19. Synthetic rout to **FπA**.

1-(4-(7-(diethylamino)-9,9-dimethyl-*9H*-fluoren-2-yl)phenyl)ethan-1-one (FπA). A mixture of **S1** (1.0 eq, 3.0 mmol, 1.03 g), 4-acetylphenylboronic acid (1.5 eq, 4.5 mmol, 0.74 g), potassium phosphate hydrate (3.0 eq, 9.0 mmol, 1.90 g) and $Pd(PPh_3)_4$ (5.0 mol%, 0.15 mmol, 0.17 g) was dissolved in 5/1/2 (v/v/v) solution of toluene, high purity water and MeOH under argon atmosphere. The reaction mixture was heated to 100°C and stirred for 6 hours, and then cooled to room temperature. The reaction mixture was quenched with water. The organic products were extracted with dichloromethane, and the organic layer was washed with water and brine. The combined organic layers were dried with $MgSO_4$, filtered, and then evaporated under reduced pressure to give a residue. Purification by silica gel column chromatography (eluent: hexane/ethyl acetate = 5/1 (v/v)) and recrystallization from hexane/dichloromethane (5/1) yielded **FπA** as an orange solid (0.96 g, 83% yield). **^{1}H NMR** (500 MHz, $CDCl_3$) δ8.03 (d, J = 8.2 Hz, 2H, Ar**H**), 7.74 (d, J = 8.2 Hz, 2H, Ar**H**), 7.63-7.54 (m, 4H, Ar**H**), 6.73 (s, 1H, Ar**H**), 6.69 (d, J = 8.2 Hz, 1H, Ar**H**), 3.44 (q, J = 7.0 Hz, 4H, NC**H**$_2$), 2.64 (s, 3H, COC**H**$_3$), 1.52 (s, 6H, C**H**$_3$), 1.23 (t, J = 7.0 Hz, 6H, C**H**$_3$) ppm. (**Figure S20**) **^{13}C NMR** (100 MHz, $CDCl_3$) δ 197.9, 156.1, 153.6, 148.2, 146.7, 140.6, 136.4, 135.4, 129.0, 127.0, 126.5, 126.3, 121.4, 121.2, 118.8, 110.9, 105.7, 46.9, 44.8, 27.7, 26.7, 12.7 ppm. (**Figure S21**) **HRMS** (EI+) m/z Calcd. For $C_{27}H_{29}NO$ $[M]^+$:383.2249, Found: 383.2257. (**Figure S22**) **FT-IR** (**Figure S23**) **m.p.**: 199.8-201.0°C.

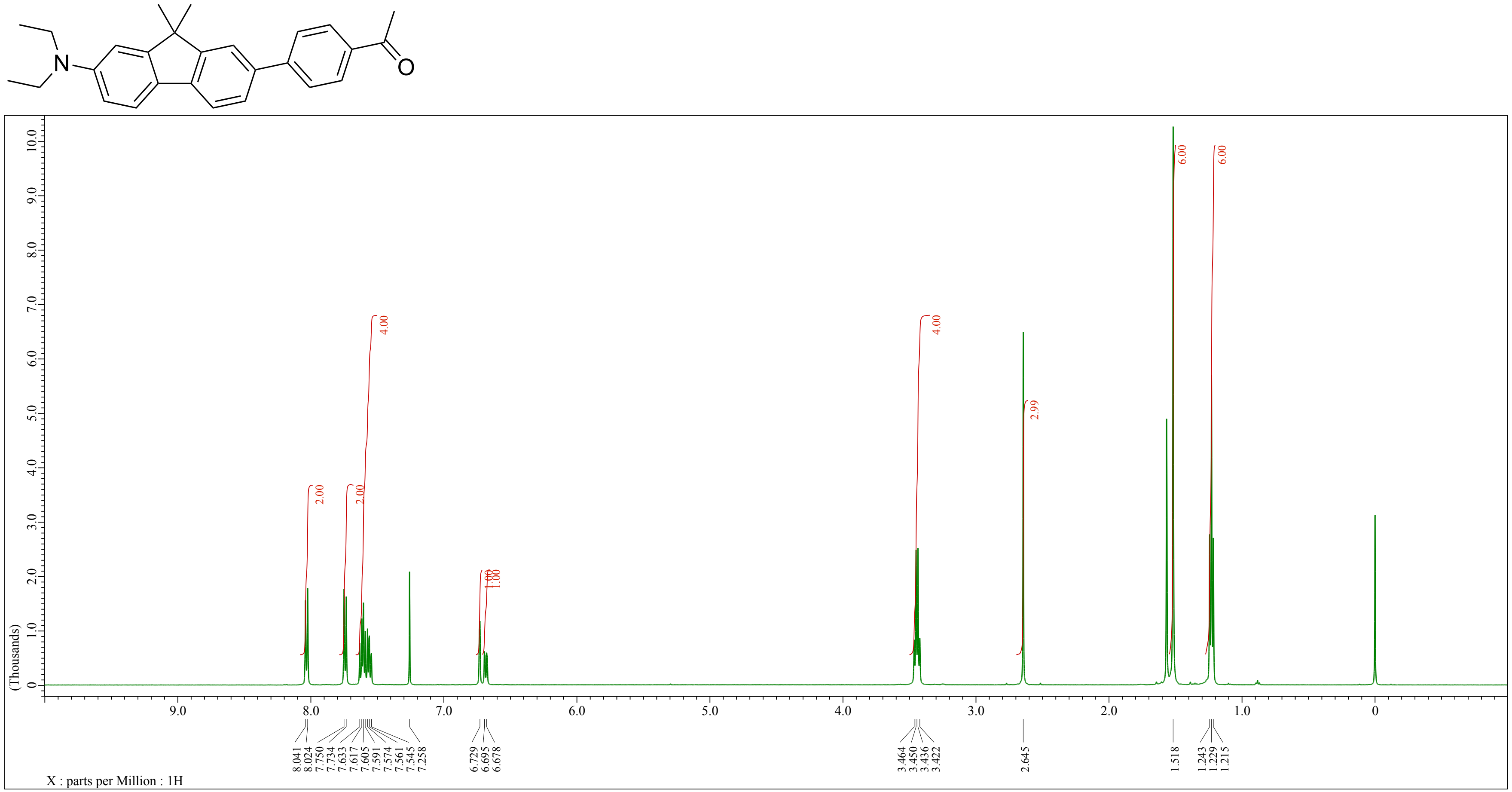

Figure S20. ^{1}H NMR (500 MHz, $CDCl_3$) spectrum of **FπA**.

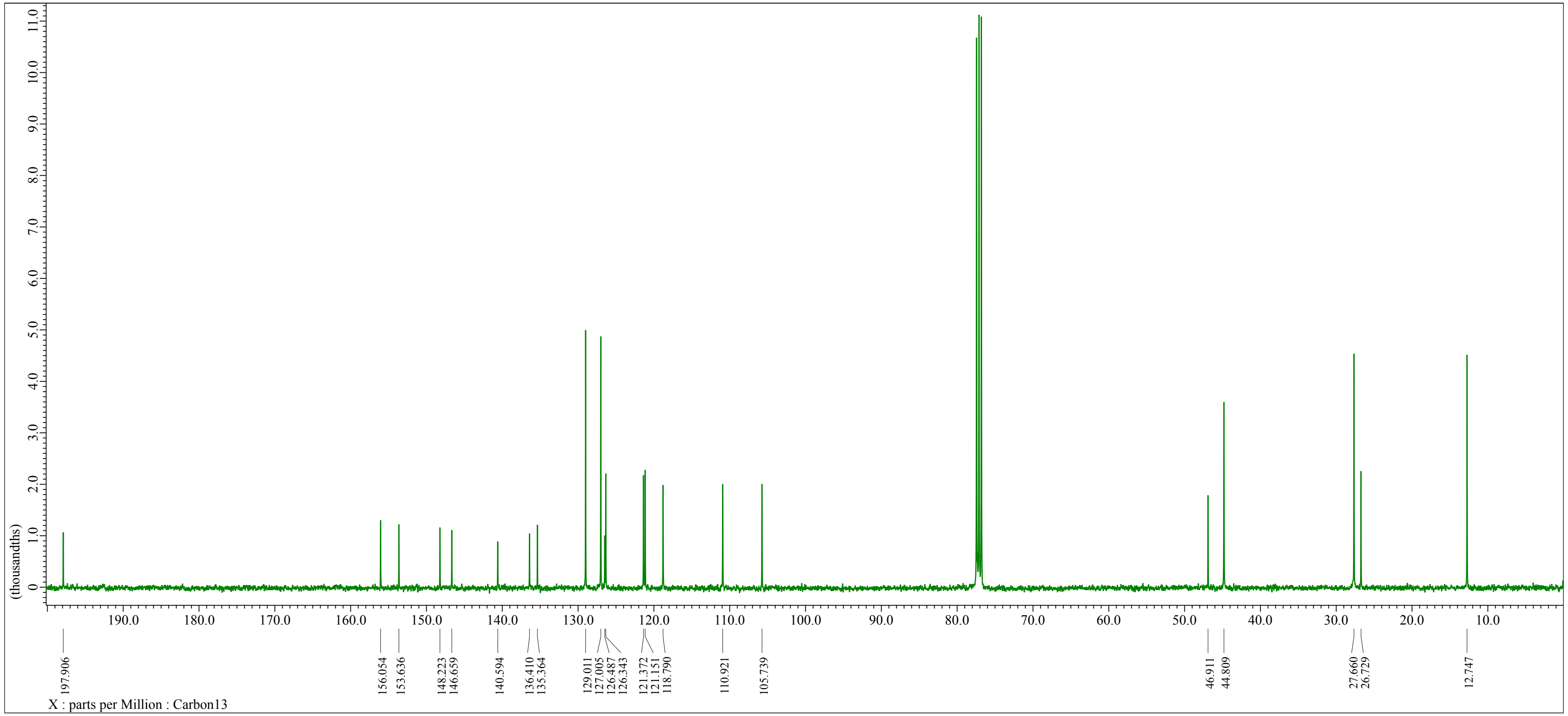

Figure S21. ^{13}C NMR (100 MHz, $CDCl_3$) spectrum of **FπA**.

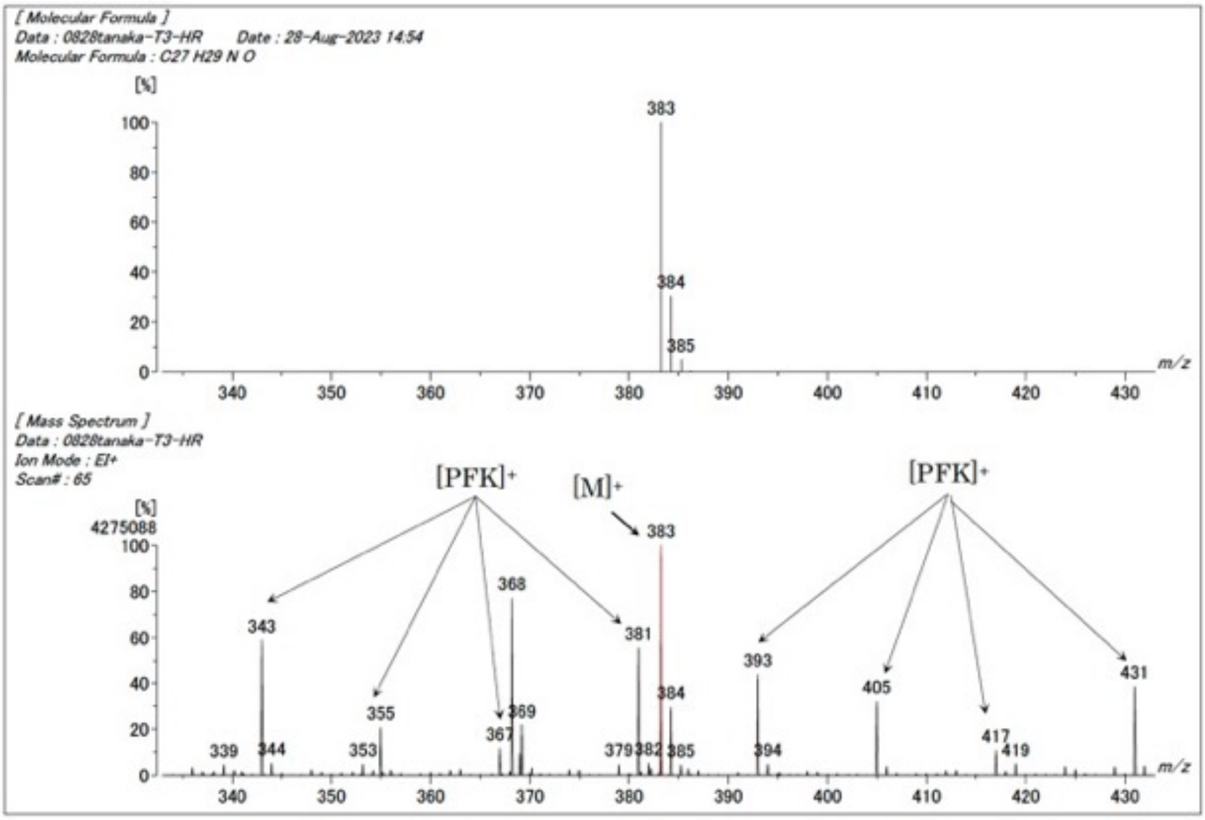

Observed m/z	Int%	Err[ppm / mmu]	U.S.	Composition
1 383.2257	100.00	+2.0 / +0.8	14.0	C27 H29 N O
2		-2.8 / -1.1	6.5	C14 H27 N10 O3
3		+4.2 / +1.6	2.0	C11 H29 N9 O6
4		+0.7 / +0.3	1.5	C13 H31 N6 O7
5		-2.8 / -1.1	1.0	C15 H33 N3 O8

Figure S22. MS spectrum of **FπA**.

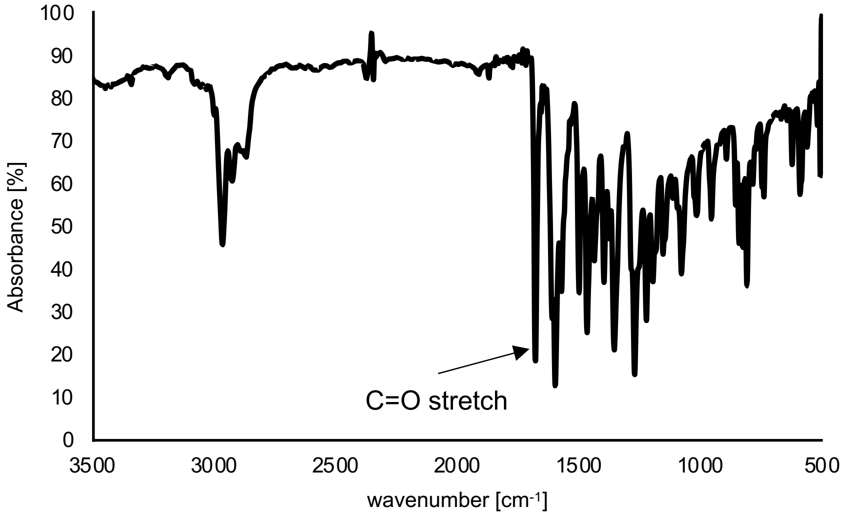

Figure S23. FT-IR spectrum of **FπA**.

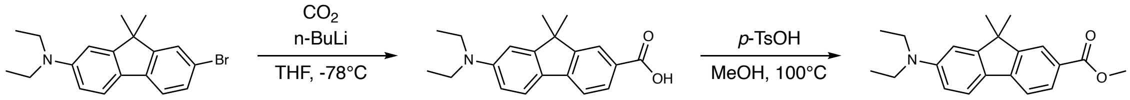

S1 S3 **FCM**

Figure S**24**. Synthetic rout to **FCM**.

7-(diethylamino)-9,9-dimethyl-*9H*-fluorene-2-carboxylic acid (S3). **S1** (1.0 eq, 5.0 mmol, 1.72 g) was dissolved in dehydrated tetrahydrofuran (0.33 M, 15 mL) under argon atmosphere. The reaction mixture was cooled to -78°C, and 2.6 M n-butyl lithium (1.5 eq, 7.5 mmol, 2.88 mL) was added dropwise. The reaction mixture was stirred for 30 min at -78°C and then, heated up to r.t. The reaction mixture was added to dry ice and stirred for 2 hours. After a while, reaction mixture was treated with 20% hydrochloric acid, filtered, and washed with water. The precipitation **S3** was dried in a vacuum. The Product was used for next reaction without purification (1.23 g, 80% yield). **^{1}H NMR** (500 MHz, $CDCl_3$) δ8.05 (d, J = 10.4 Hz, 2H, Ar**H**), 7.59 (dd, J = 10.5, 8.4 Hz, 2H, Ar**H**), 6.70-6.68 (m, 2H, Ar**H**), 3.45 (q, J = 7.1 Hz, 4H, NC**H**$_2$), 1.49 (s, 6H, C**H**$_3$), 1.23 (t, J = 7.2 Hz, 6H, C**H**$_3$) ppm. **(Figure S25**)

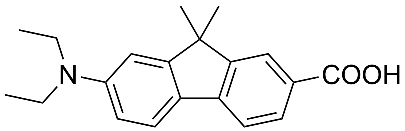

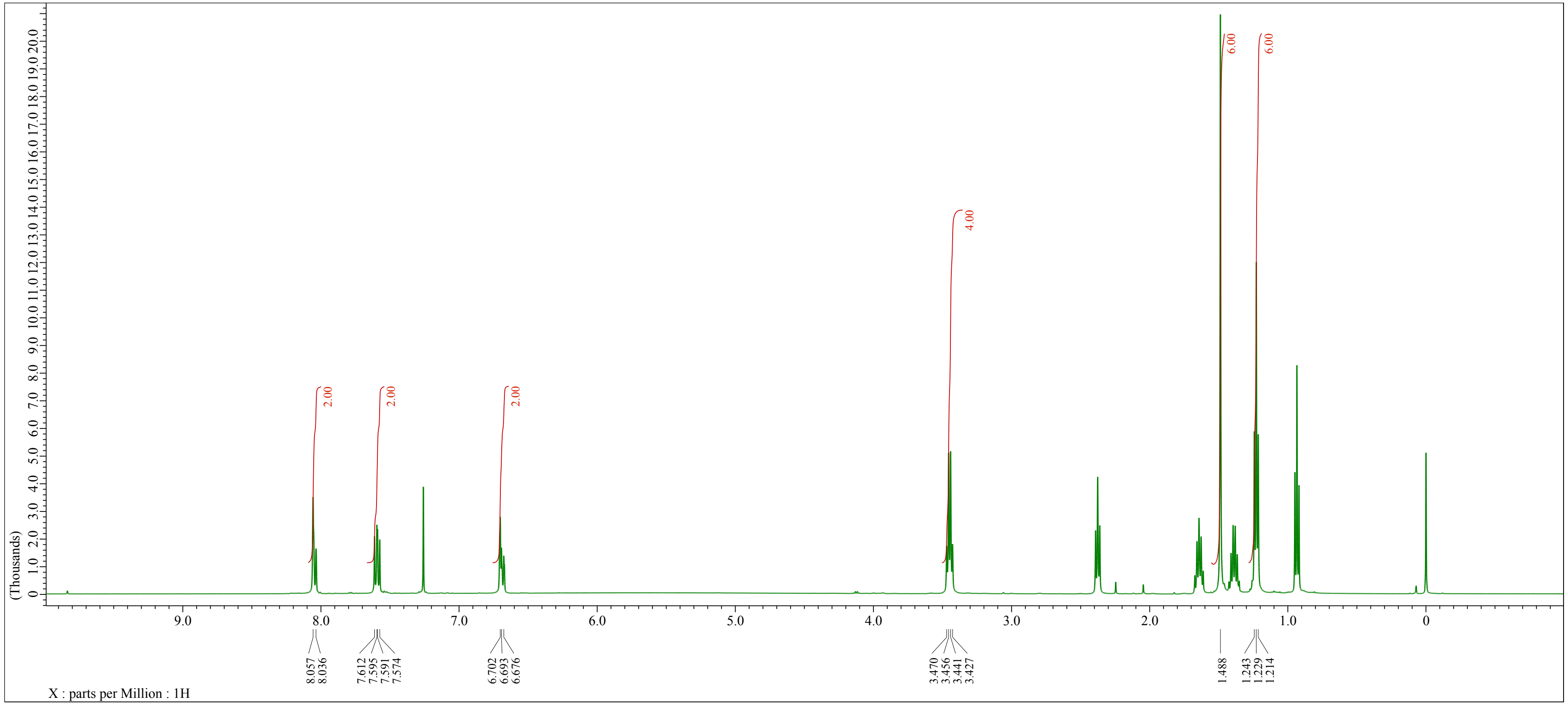

Figure S25. ^{1}H NMR (500 MHz, $CDCl_3$) spectrum of **S3**.

methyl 7-(diethylamino)-9,9-dimethyl-9H-fluorene-2-carboxylate (FCM). A mixture of **S3** (1.0 eq, 4.0 mmol, 1.23 g), *p*-TsOH, and MeOH (30 mL) was refluxed for 18 hours. After cooling to r.t, the reaction mixture was extracted with dichloromethane, washed with water and brine, and combined organic layers were dried with magnesium sulfate, filtered, and then evaporated under reduced pressure to give residue. Purification by silica gel column chromatography (eluent: hexane/ethyl acetate = 3/1 (v/v)) and recrystallization from hexane/dichloromethane (5/1) yielded **FCM** as a yellowish solid (0.86 g, 67% yield). **^{1}H NMR** (500 MHz, $CDCl_3$) δ8.01 (s, 1H, Ar**H**), 7.98 (d, *J* = 7.9 Hz, 1H, Ar**H**), 7.57 (dd, *J* = 14.0, 8.2 Hz, 2H, Ar**H**), 6.70 (s, 1H, Ar**H**), 6.67 (d, *J* = 8.5 Hz, 1H, Ar**H**), 3.92 (s, 3H, OC**H**$_3$), 3.44 (q, *J* = 7.0 Hz, 4H, NC**H**$_2$), 1.48 (s, 6H, C**H**$_3$), 1.22 (t, *J* = 7.0 Hz, 6H, C**H**$_3$) ppm. (**Figure S26**) **^{13}C NMR** (100 MHz, $CDCl_3$) δ 167.8, 156.9, 152.6, 148.8, 145.1, 129.2, 126.2, 125.8, 123.6, 122.1, 117.8, 110.9, 105.4, 51.9, 46.8, 44.8, 27.4, 12.7 ppm. (**Figure S27**) **HRMS** (EI+) m/z Calcd. For $C_{21}H_{25}NO_2$ $[M]^+$:323.1885, Found: 323.1880 (**Figure S27**) **FT-IR** (**Figure S28**) **m.p.**: 99.0-102.8°C.

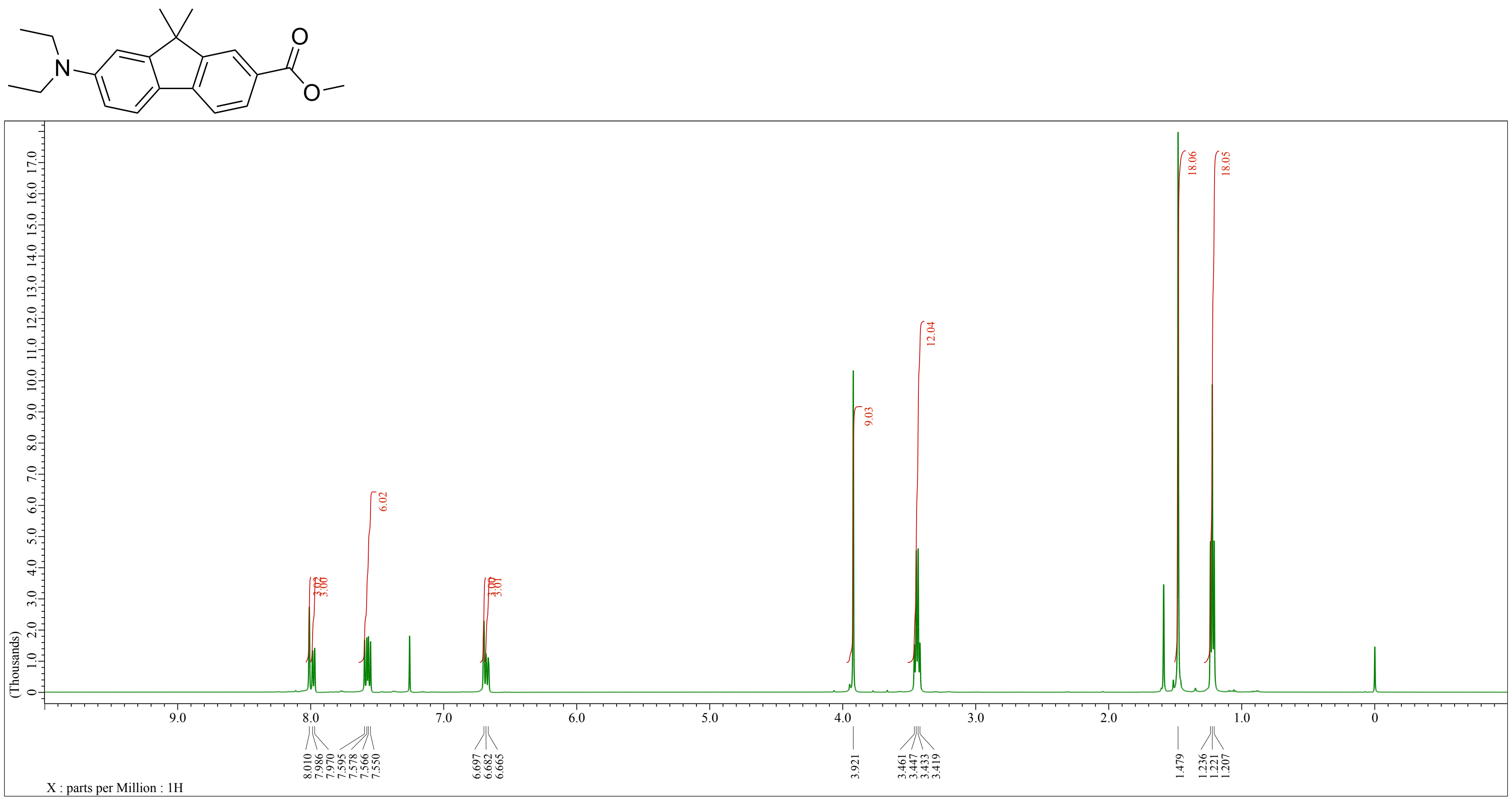

Figure S26. ^{1}H NMR (500 MHz, $CDCl_3$) spectrum of **FCM**.

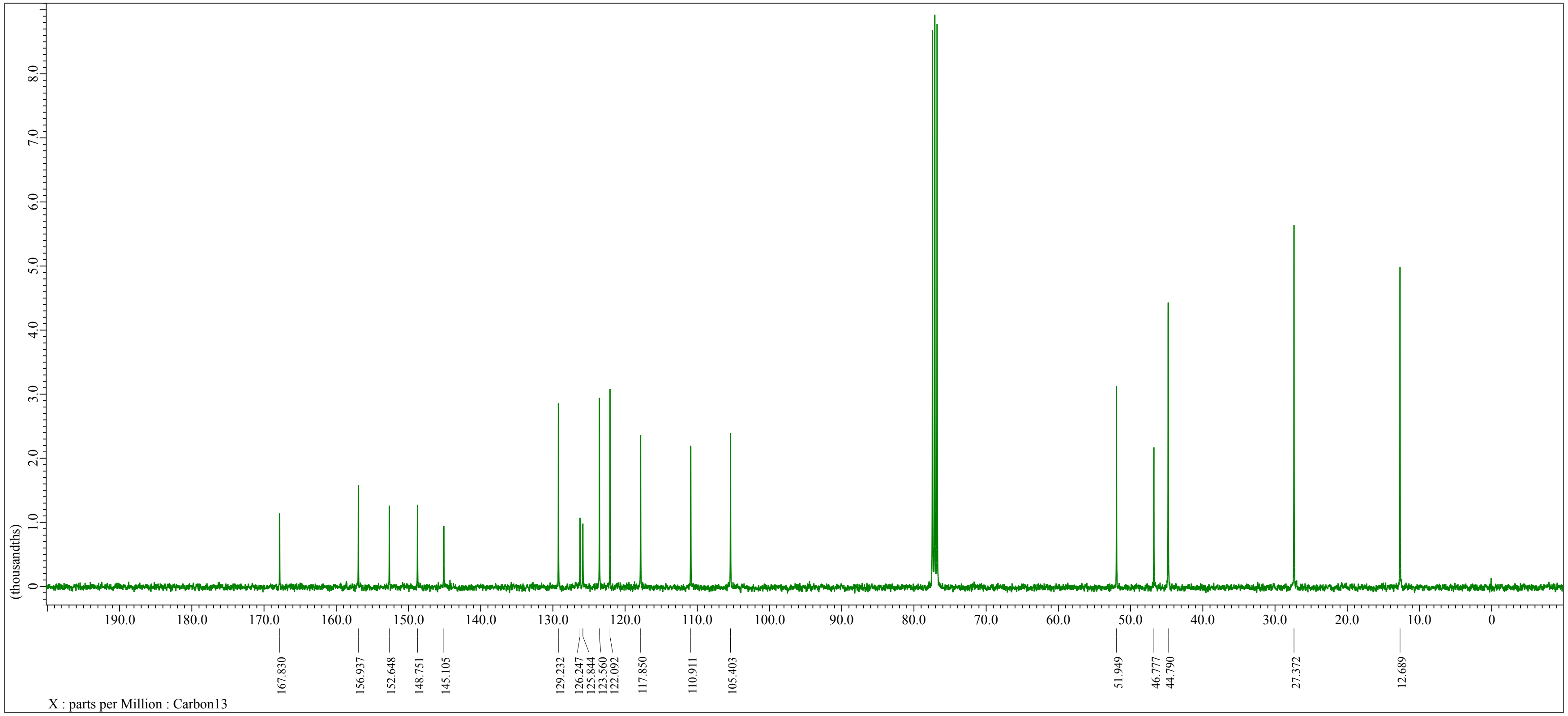

Figure S27. ^{13}C NMR (100 MHz, $CDCl_3$) spectrum of **FCM**.

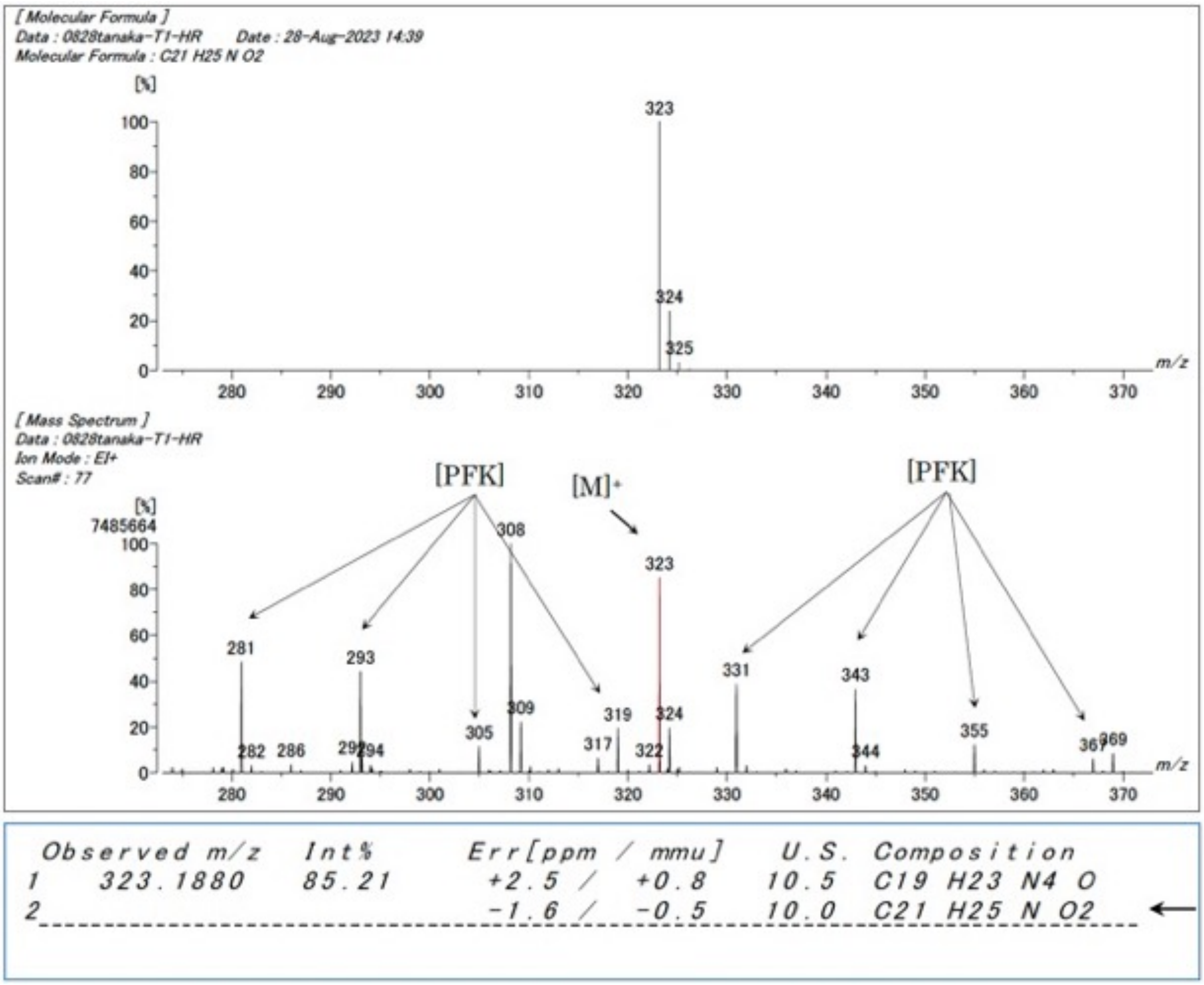

Figure S28. MS spectrum of **FCM**.

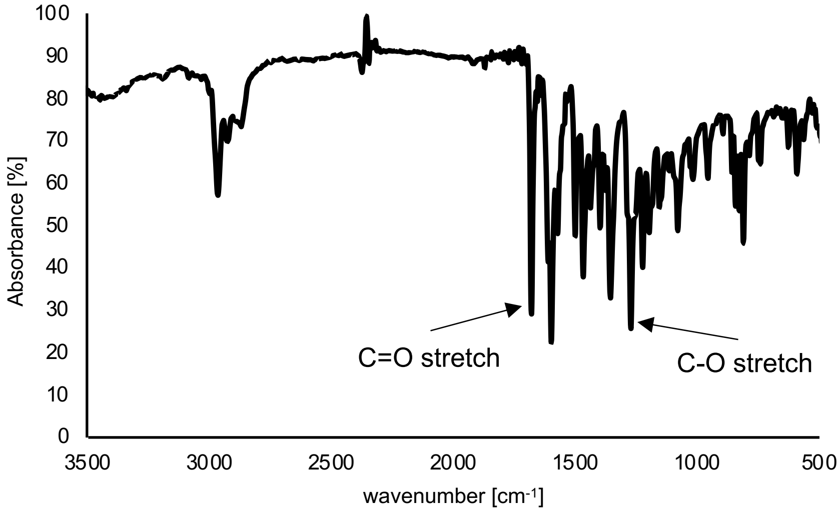

Figure S29. FT-IR spectrum of **FCM**.

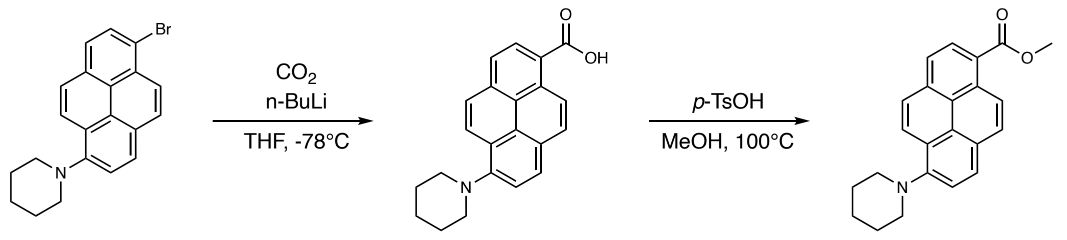

Figure S30. Synthetic rout to **PCM**.

The starting material S3 was synthesized adapting published protocols.[S3]

6-(piperidin-1-yl)pyrene-1-carboxylic acid (S4). **S3** (1.0 eq, 3.0 mmol, 1.09 g) was dissolved in dehydrated tetrahydrofuran (0.15 M, 20 mL) under argon atmosphere. The reaction mixture was cooled to -78°C, and 2.6 M *n*-butyl lithium (1.5 eq, 4.50 mmol, 1.73 mL) was added dropwise. The reaction mixture was stirred for 30 min at -78°C and then, heated up to r.t. The reaction mixture was added to dry ice and stirred for 2 hours. After a while, reaction mixture was treated with 20% hydrochloric acid, filtered, and washed with water. The precipitation **S4** was dried in a vacuum. The progress of the reaction was confirmed by TLC and the Product was used for next reaction without purification.

methyl 6-(piperidin-1-yl)pyrene-1-carboxylate (PCM). A mixture of **S4**, *p*-TsOH (30 mg), and MeOH (30 mL) was refluxed for 18 hours. After cooling to r.t, the reaction mixture was extracted with dichloromethane, washed with water and brine, and combined organic layers were dried with $MgSO_4$, filtered, and then evaporated under reduced pressure to give residue. Purification by silica gel column chromatography (eluent: hexane/ethyl acetate = 3/1 (v/v)) and recrystallization from hexane/dichloromethane (5/1) yielded **PCM** as a yellowish solid (0.52 g, 50% yield). **^{1}H NMR** (500 MHz, $CDCl_3$) δ9.11 (d, J = 9.5 Hz, 1H, Ar**H**), 8.58 (d, J = 8.2 Hz, 1H, Ar**H**), 8.53 (d, J = 9.2 Hz, 1H, Ar**H**), 8.18 (d, J = 8.2 Hz, 1H, Ar**H**), 8.14-8.08 (m, 2H, Ar**H**), 8.05 (d, J = 9.2 Hz, 1H, Ar**H**), 7.76 (d, J = 8.2 Hz, 1H, Ar**H**), 4.08 (s, 3H, OC**H**$_3$), 3.22 (s, 4H, NC**H**$_2$), 1.96-1.91 (m, 4H, C**H**$_2$), 1.72 (s, 2H, C**H**$_2$) ppm. (**Figure S31**) **^{13}C NMR** (100 MHz, $CDCl_3$) δ 168.7, 150.6, 134.7, 131.9, 129.6, 128.6, 126.9, 126.3, 126.1, 125.7, 125.6, 124.8, 123.5, 123.0, 122.7, 117.6, 55.3, 52.3, 26.8, 24.6 ppm. (**Figure S32**) **HRMS** (EI+) m/z Calcd. For $C_{23}H_{21}NO_2$ $[M]^+$: 343.1572, Found: 343.1567 (**Figure S33**) **FT-IR** (**Figure S34**) **m.p.**: 160.8-162.0°C.

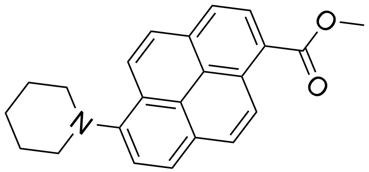

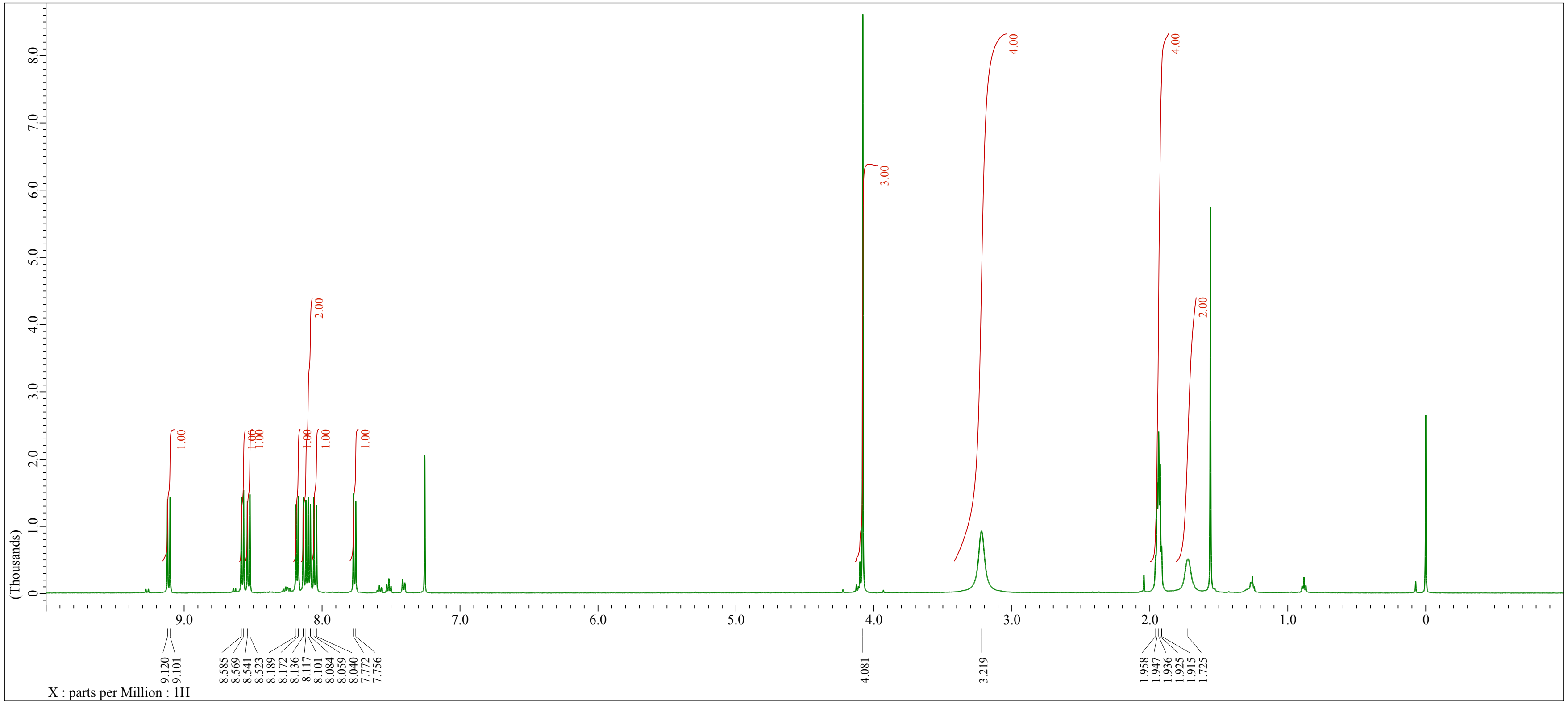

Figure S31. ^{1}H NMR (500 MHz, $CDCl_3$) spectrum of **PCM**.

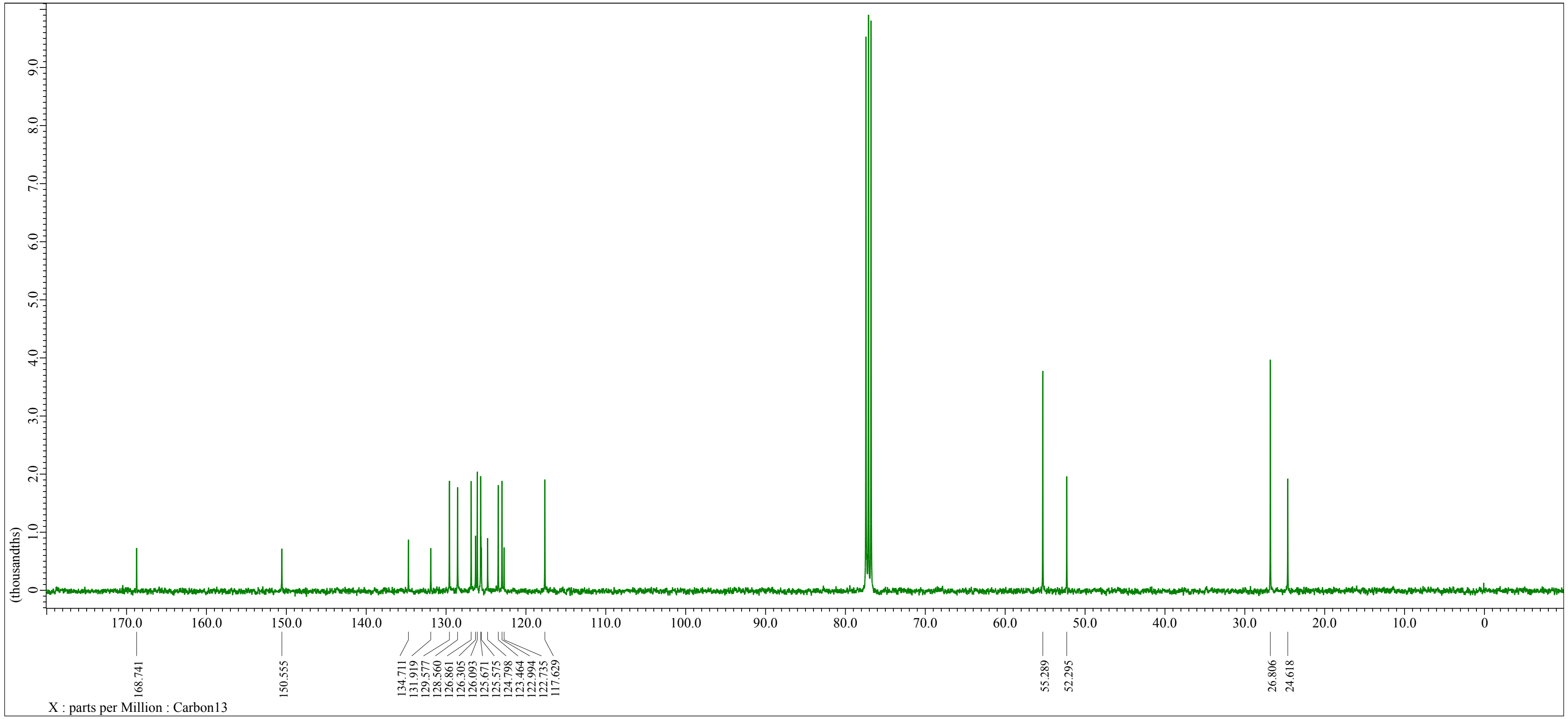

Figure S32. ^{13}C NMR (100 MHz, $CDCl_3$) spectrum of **PCM**.

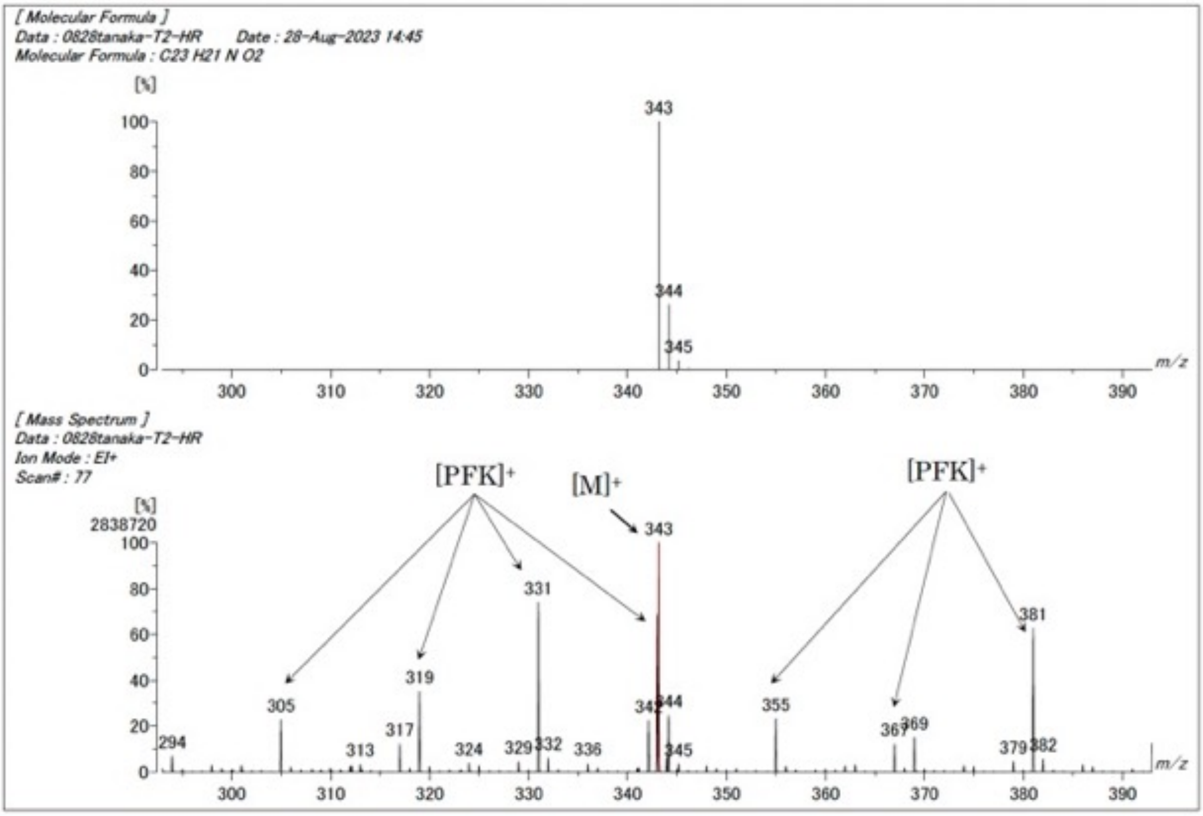

	Observed m/z	Int%	Err[ppm / mmu]	U.S.	Composition
1	343.1567	100.00	+2.4 / +0.8	14.5	C21 H19 N4 O
2			-1.5 / -0.5	14.0	C23 H21 N O2 ←
3			+0.9 / +0.3	2.0	C7 H21 N9 O7
4			-3.0 / -1.0	1.5	C9 H23 N6 O8

Figure S33. MS spectrum of **PCM**.

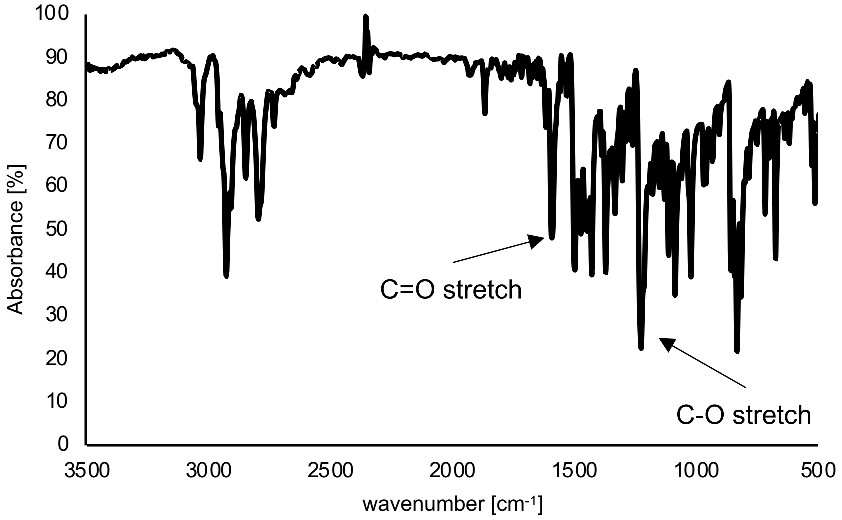

Figure S34. FT-IR spectrum of **PCM**.

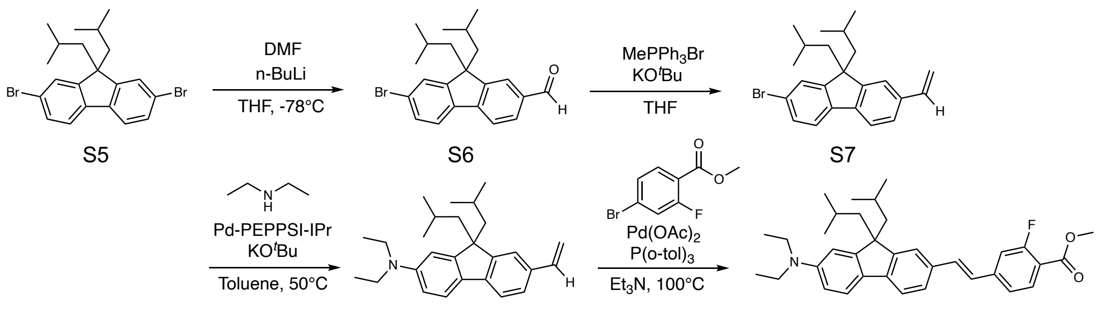

Figure S35. Synthetic rout to **FstCM*o*-F**.

7-bromo-9,9-diisobutyl-9*H*-fluorene-2-carbaldehyde (S6). S5 (1.0 eq, 5.00 mmol, 2.18 g) was dissolved in dehydrated tetrahydrofuran (0.20 M, 25 mL) under argon atmosphere. The reaction mixture was cooled to -78°C, and 2.6 M *n*-butyl lithium (1.5 eq, 7.5 mmol, 2.88 mL) was added dropwise. The reaction mixture was stirred for 30 min at -78°C and then, heated up to r.t. *N,N*-dimethylformamide (DMF) (1.10 eq, 5.50 mmol, 0.40 mL) was added to the reaction mixture. After 2 hours, the reaction mixture was extracted with dichloromethane, washed with water and brine, and combined organic layers were dried with magnesium sulfate, filtered, and then evaporated under reduced pressure to give residue. Purification by silica gel column chromatography (eluent: hexane/ethyl acetate = 3/1 (v/v)) yielded **S6** as a colorless solid (1.23 g, 64% yield). **^{1}H NMR** (500 MHz, $CDCl_3$) δ10.06 (s, 1H, C**H**O), 7.88 (t, *J* = 7.8 Hz, 2H, Ar**H**), 7.82 (d, *J* = 7.9 Hz, 1H, Ar**H**), 7.65 (d, *J* = 7.9 Hz, 1H, Ar**H**), 7.55 (s, 1H, Ar**H**), 7.52 (d, *J* = 7.9 Hz, 1H, Ar**H**), 1.94 (m, 4H, C$\mathbf{H}_2$), 0.87-0.79 (m, 2H, C**H**), 0.36 (dd, *J* = 24.1, 6.4 Hz, 12H, C$\mathbf{H}_3$) ppm. (**Figure S36**)

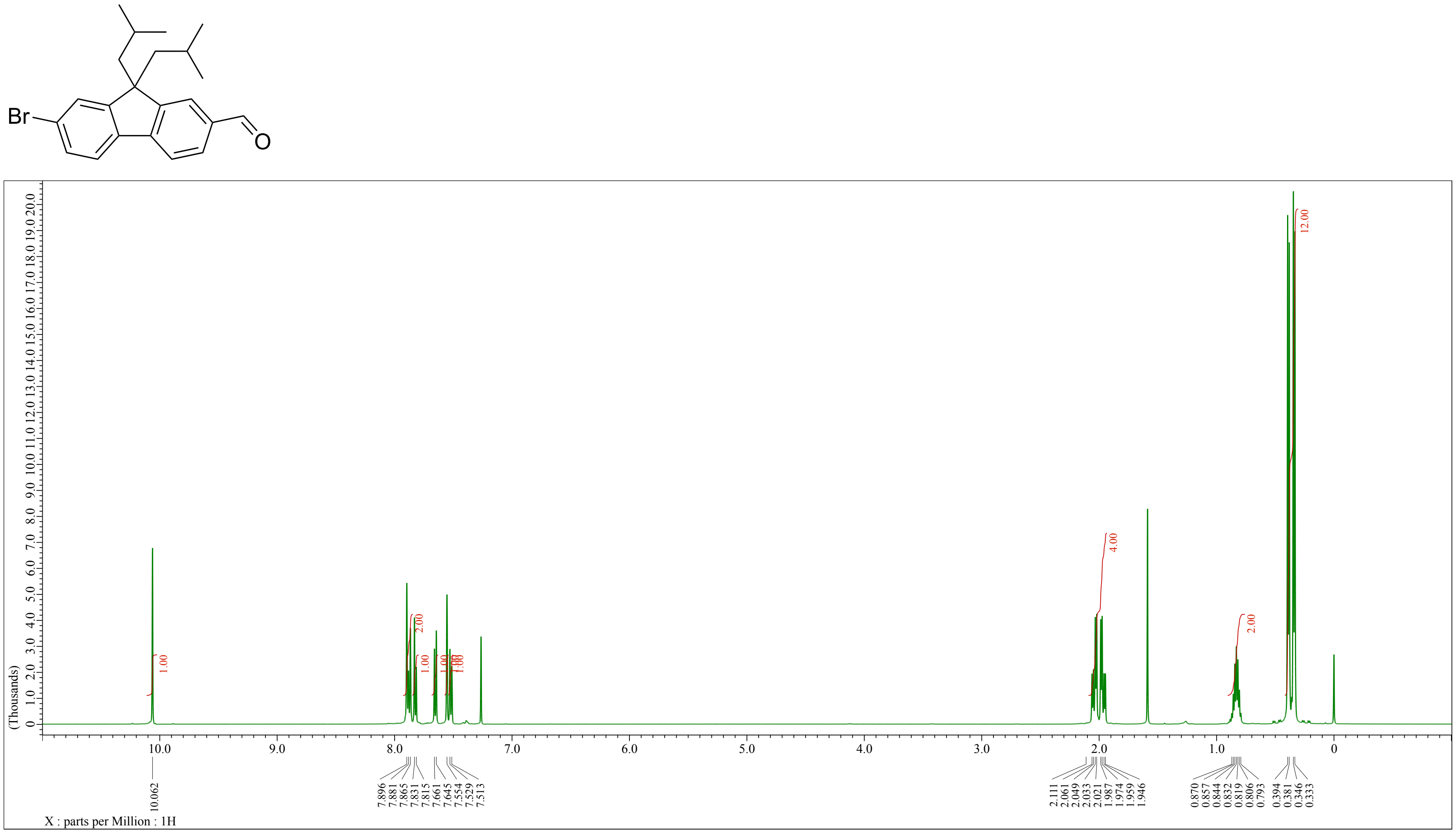

Figure S36. ^1H NMR (500 MHz, $CDCl_3$) spectrum of **S6**.

2-bromo-9,9-diisobutyl-7-vinyl-9*H*-fluorene (S7). Methyl triphenyl phosphonium bromide (1.2 eq, 1.37 g, 3.84 mmol) was dissolved in THF (30 mL) under an argon atmosphere. The solution was cooled to 0 °C, and then KO^{t}Bu (1.5 eq, 0.537 g, 4.80 mmol) was added. Following stirring for 10 minutes, **S6** (1.0 eq, 1.23 g, 3.20 mmol) was added and then warmed to room temperature and stirred for 2 hours. The reaction mixture was quenched with sat. NH_4Cl aq. and organic products were extracted with dichloromethane. The organic layer was washed with water and brine. The combined organic layers were dried with $MgSO_4$, filtered, and then evaporated under reduced pressure to give a residue. Purification by column chromatography on silica (hexane/dichloromethane = 3:1 (v/v)) gave **S7** as a colorless oil. (0.98 g, 80% yield); **^{1}H NMR** (500 MHz, $CDCl_3$) δ7.62 (d, *J* = 7.9 Hz, 1H, Ar**H**), 7.54 (d, *J* = 8.2 Hz, 1H, Ar**H**), 7.47 (s, 1H, Ar**H**), 7.45 (d, *J* = 7.9 Hz, 1H, Ar**H**), 7.40 (d, *J* = 7.9 Hz, 1H, Ar**H**), 7.37 (s, 1H, Ar**H**), 6.79 (dd, J = 17.4, 10.7 Hz, 1H, C**H**=CH_2), 5.79 (d, *J* = 17.4 Hz, 1H, CH=C**H**$_2$), 5.27 (d, *J* = 10.7 Hz, 1H, CH=C**H**$_2$), 1.94 (m, 4H, C**H**$_2$), 0.89-0.82 (m, 2H, C**H**), 0.37 (q, *J* = 7.1 Hz, 12H, C**H**$_3$) ppm. (**Figure S37**)

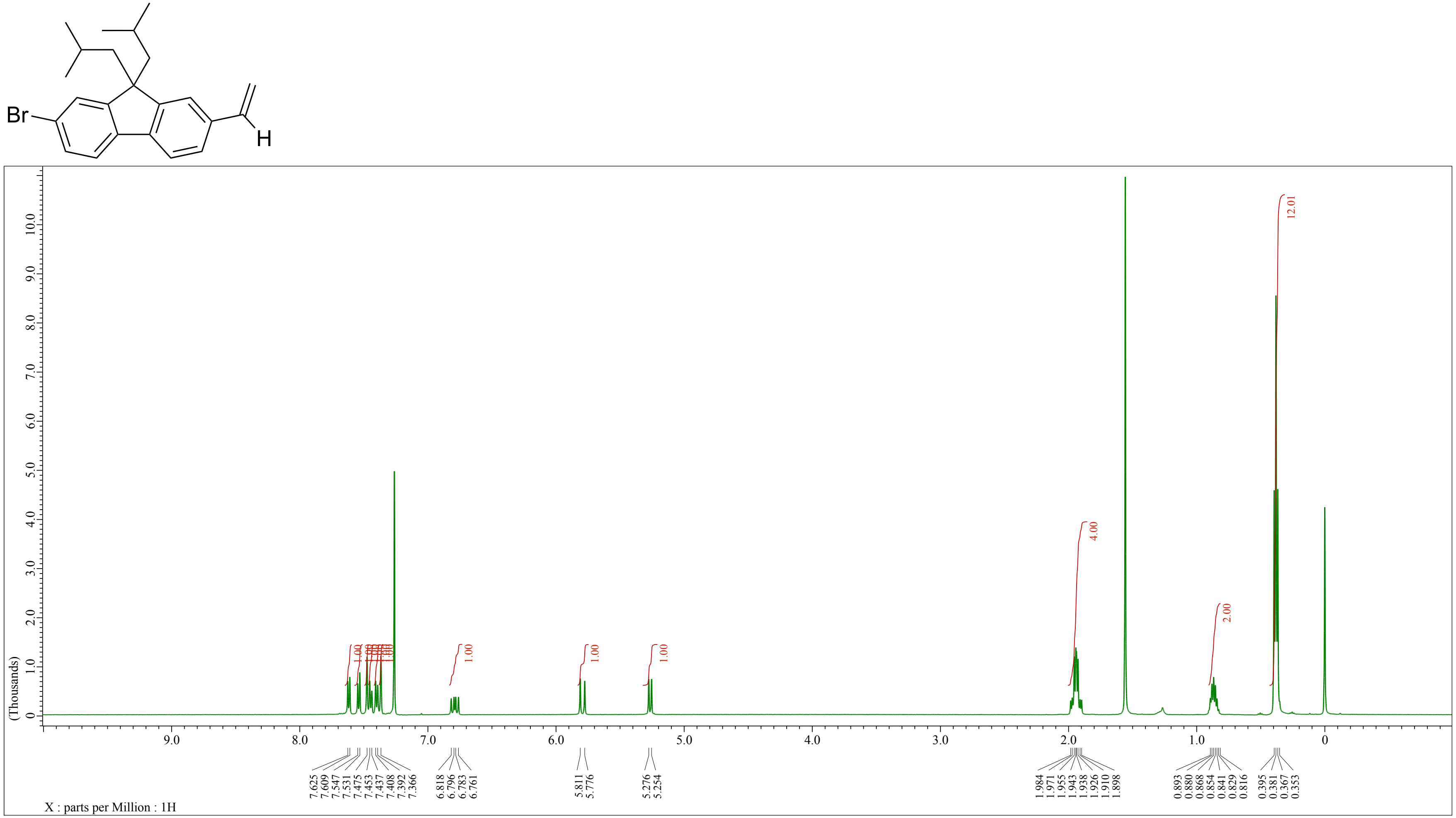

Figure S37. ^{1}H NMR (500 MHz, $CDCl_3$) spectrum of **S7**.

***N*,*N*-diethyl-9,9-diisobutyl-7-vinyl-9*H*-fluoren-2-amine (S8).** A mixture of **S7** (1.0 eq, 0.98 g, 2.56 mmol), KOtBu (1.5 eq, 0.430 g, 3.84 mmol), Diethylamine (2.0 eq, 0.532 mL, 5.10 mmol), and Pd-PEPPSI-IPr (5.0 mol%, 0.09 g, 0.13 mmol) was dissolved in dehydrated Toluene (20 mL) and stirred for 12h at 50°C. After cooling to r.t, the reaction mixture was quenched with water, extracted with dichloromethane, and washed with water and brine. The combined organic layers were dried with $MgSO_4$, filtered, and then evaporated under reduced pressure to give a residue. Purification by column chromatography on silica (hexane/ethyl acetate = 5:1 (v/v)) gave **S8** as a yellowish oil. (0.78 g, 81% yield); **^{1}H NMR** (500 MHz, $CDCl_3$) δ7.48 (dd, *J* = 14.2, 8.1 Hz, 2H, Ar**H**), 7.33 (s, 1H, Ar**H**), 6.77 (dd, *J* = 17.5, 10.8 Hz, 1H, C**H**=CH_2), 6.68-6.64 (m, 2H, Ar**H**), 5.72 (d, *J* = 17.7 Hz, 1H, CH=C**H_2**), 5.16 (d, *J* = 11.0 Hz, 1H, CH=C**H_2**), 3.41 (q, *J* = 6.9 Hz, 4H, NC**H_2**), 1.90 (m, 4H, C**H_2**), 1.19 (t, *J* = 7.0 Hz, 6H, C**H_3**), 0.93 (q, *J* = 6.2 Hz, 2H, C**H**), 0.41 (dd, *J* = 16.9, 6.6 Hz, 12H, C**H_3**) ppm. (**Figure S38**)

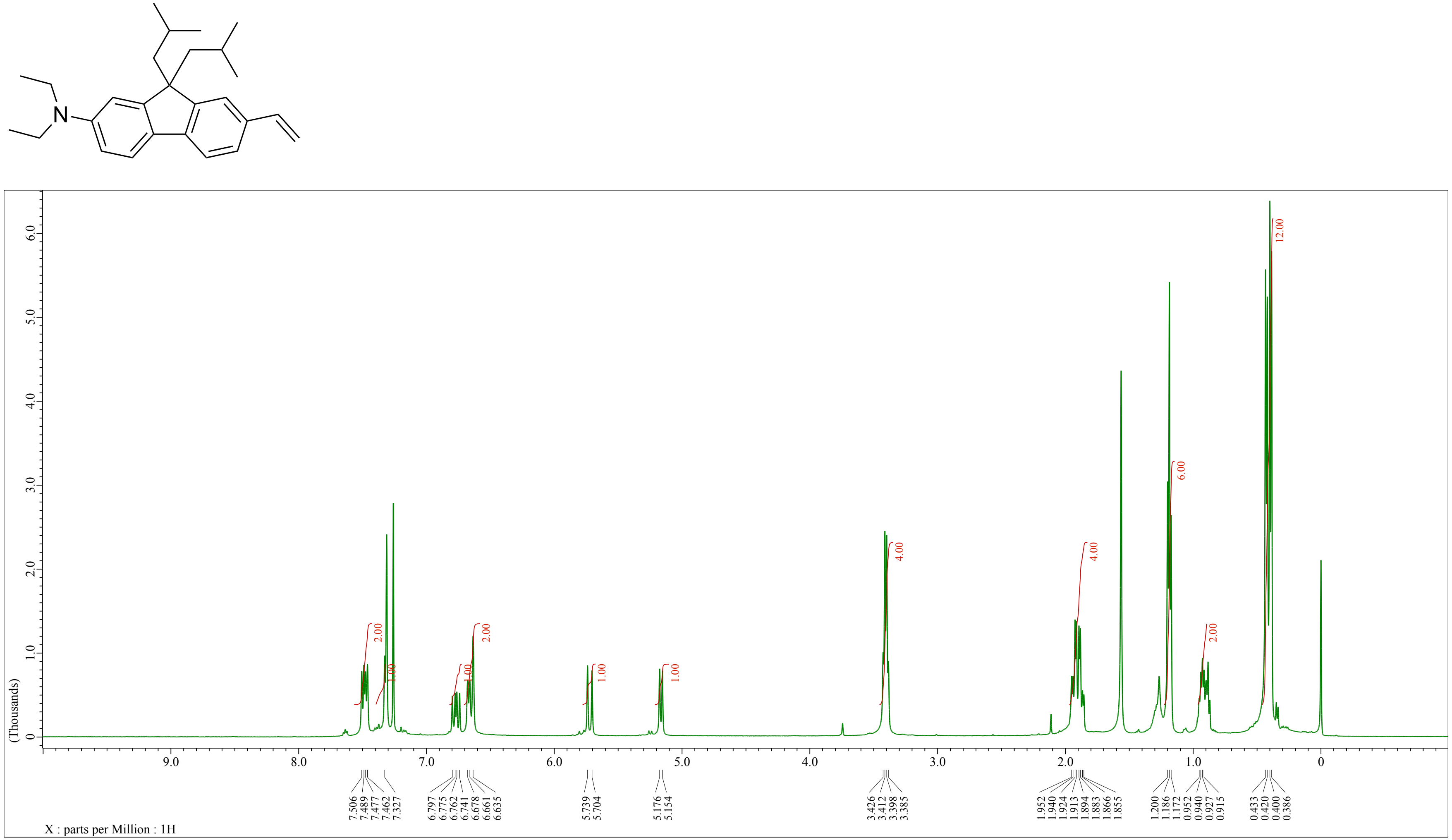

Figure S38. ^{1}H NMR (500 MHz, $CDCl_3$) spectrum of **S8**.

methyl (*E*)-4-(2-(7-(diethylamino)-9,9-diisobutyl-9*H*-fluoren-2-yl)vinyl)-2-fluorobenzoate (FstCMo-F). A mixture of **S8** (1.0 eq, 0.78 g, 2.07 mmol), tri(*o*-tolyl)phosphine (10 mol%, 0.06 g, 0.207 mmol), palladium(II) acetate (5.0 mol%, 0.02 g, 0.103 mmol), and methyl 4-bromo-2-fluorobenzoate (1.5 eq, 0.723 g, 3.11 mmol) was dissolved in triethylamine (5 mL) in the pressure-resistant tube and stirred for 12h at 100°C. After cooling to r.t, the reaction mixture was quenched with water, extracted with dichloromethane, and washed with water and brine. The combined organic layers were dried with $MgSO_4$, filtered, and then evaporated under reduced pressure to give a residue. Purification by column chromatography on silica (hexane/ethyl acetate = 5:1 (v/v)) and recrystallization with hexane/dichloromethane (3/1 (v/v)) gave **FstCM*o*-F** as a yellow solid. (0.20 g, 21% yield); **^{1}H NMR** (500 MHz, $CDCl_3$) δ7.92 (t, *J* = 7.9 Hz, 1H, Ar**H**), 7.52 (d, *J* = 8.2 Hz, 2H, Ar**H**), 7.45-7.43 (m, 2H, Ar**H**), 7.34 (d, *J* = 7.9 Hz, 1H, C**H**=C**H**), 7.29 (s, 1H, Ar**H**), 7.04 (d, *J* = 16.2 Hz, 1H, Ar**H**), 6.68 (d, *J* = 8.2 Hz, 1H, C**H**=C**H**), 6.63 (s, 1H, Ar**H**), 3.94 (s, 3H, OC**H**$_3$), 3.42 (q, *J* = 7.0 Hz, 4H, NC**H**$_2$), 1.94 (m, 4H, C**H**$_2$), 1.20 (t, *J* = 7.0 Hz, 6H, C**H**$_3$), 0.97-0.92 (m, 2H, C**H**), 0.43 (dd, *J* = 20.1, 6.7 Hz, 12H, C**H**$_3$) ppm. (**Figure S39**) **^{13}C NMR** (100 MHz, $CDCl_3$) δ 164.9, 163.8, 161.2, 152.9, 150.4, 147.9, 145.0, 144.9, 143.4, 133.8, 132.5, 129.2, 126.3, 124.0, 121.9, 121.8, 120.9, 118.4, 116.3, 114.0, 113.8, 111.4, 107.5, 54.6, 52.3, 50.8, 45.0, 31.7, 24.8, 24.7, 24.7, 22.7, 14.2, 12.7 ppm. (**Figure S40**) The number of C atoms of ^{13}C NMR for **FstCMo-F** (^{13}C-NMR data) is more than the theoretical data, because F atoms can cause splits of C atoms in ^{13}C NMR spectrum.[S2] **HRMS** (EI+) m/z Calcd. For $C_{35}H_{42}NO_2$ $[M]^+$: 527.3200, Found: 527.3209 (**Figure S41**) **FT-IR** (**Figure S42**) **m.p.**: 129.3-131.6°C.

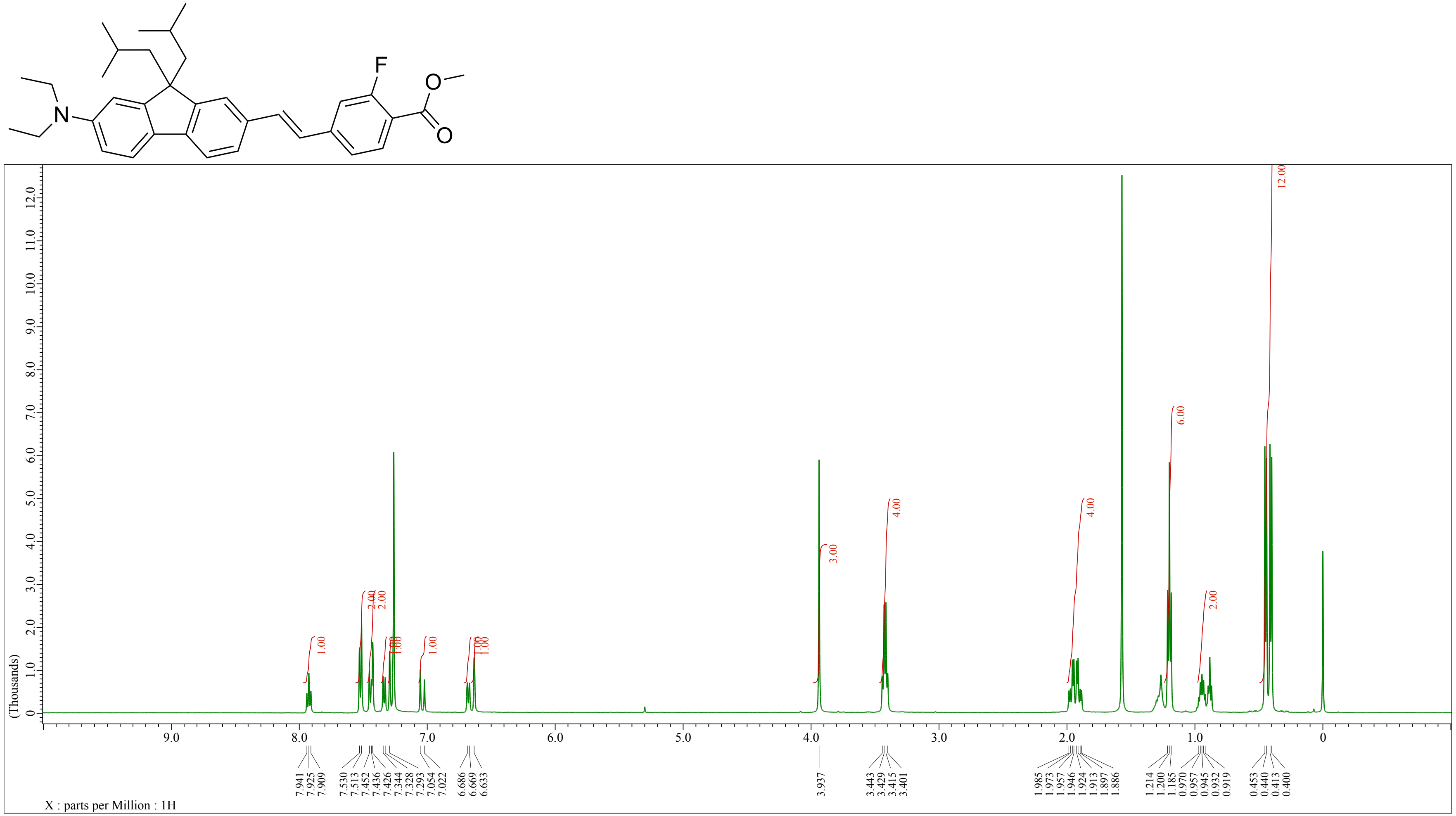

Figure S39. ^{1}H NMR (500 MHz, $CDCl_3$) spectrum of **FstCM*o*-F**.

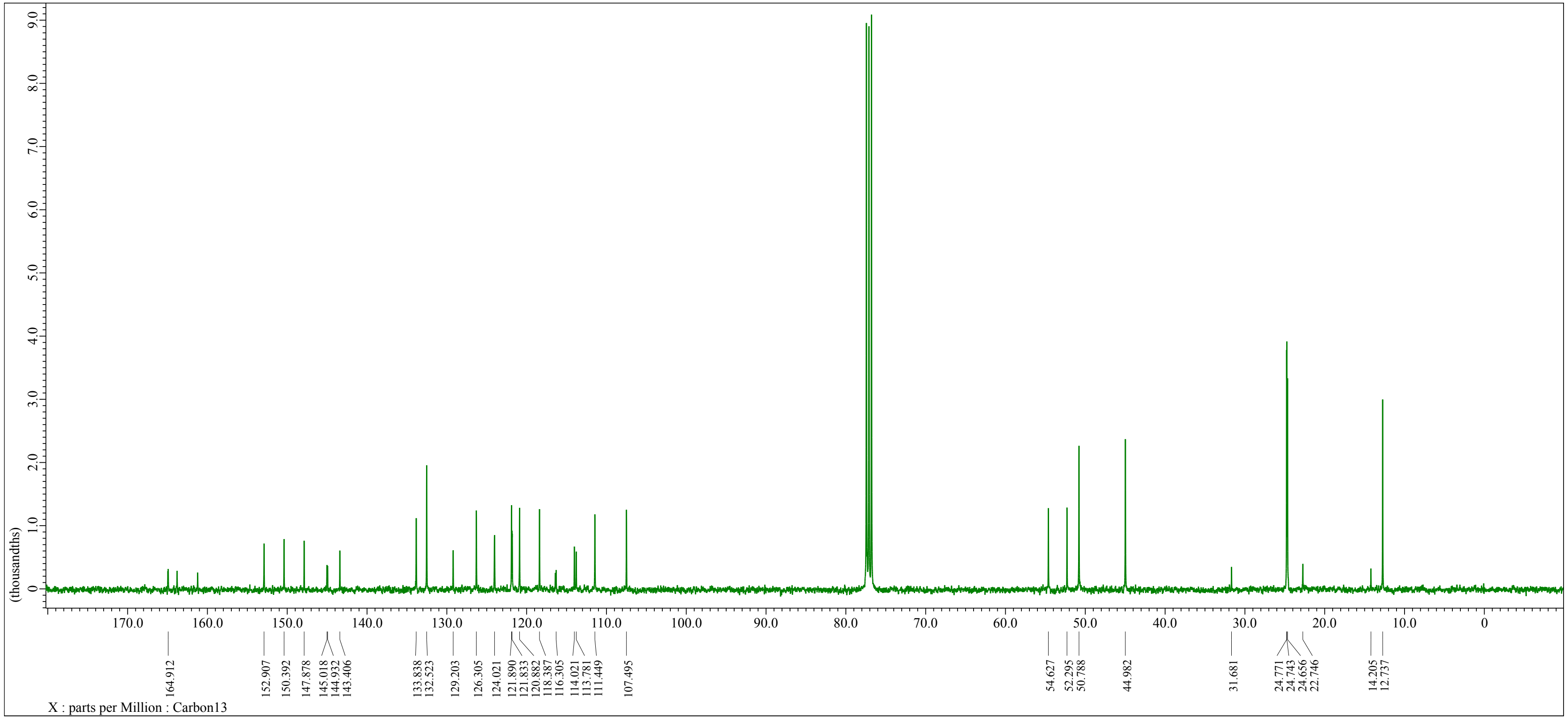

Figure S40. ^{13}C NMR (100 MHz, $CDCl_3$) spectrum of **FstCM*o*-F**.

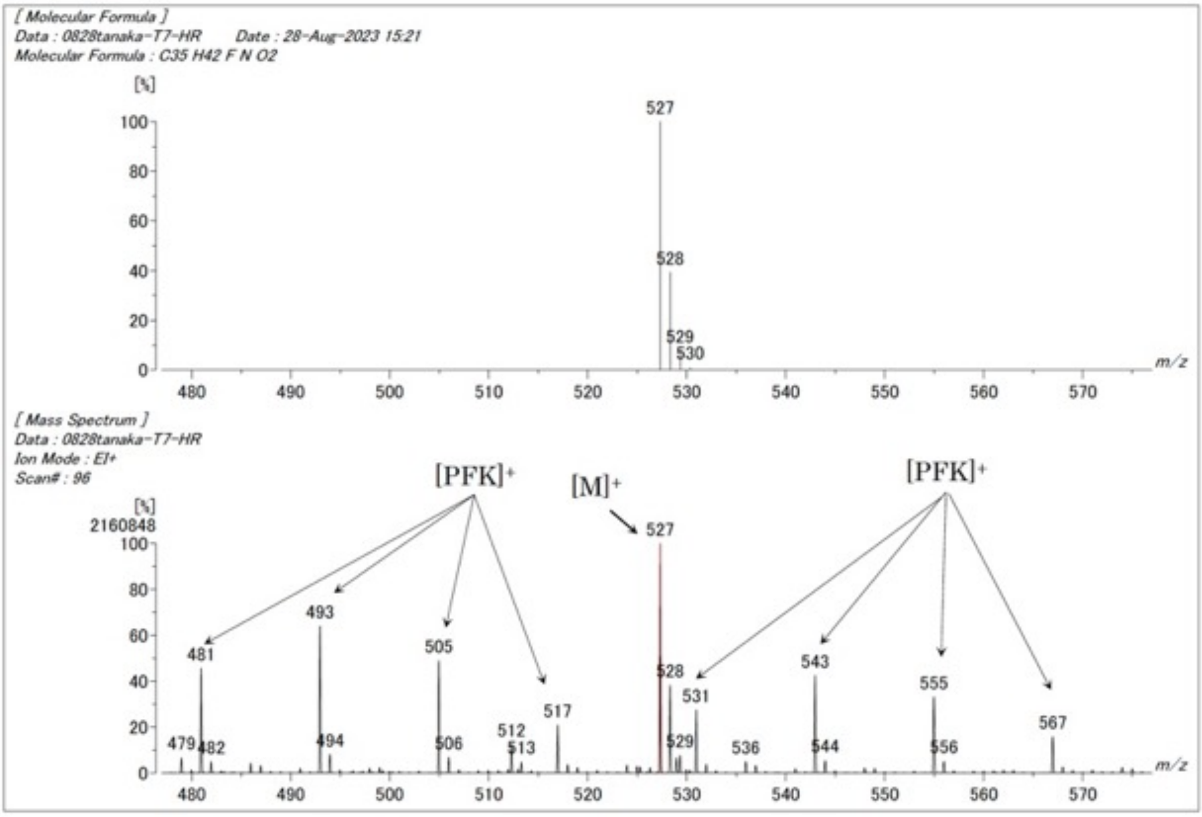

Observed m/z	Int%	Err[ppm / mmu]	U.S.	Composition
1 527.3209	100.00	+1.8 / +0.9	15.0	C35 H42 F N O2
2		-1.7 / -0.9	7.5	C22 H40 F N10 O4
3		+3.4 / +1.8	3.0	C19 H42 F N9 O7
4		+0.8 / +0.4	2.5	C21 H44 F N6 O8
5		-1.7 / -0.9	2.0	C23 H46 F N3 O9

Figure S41. MS spectrum of **FstCM*o*-F**.

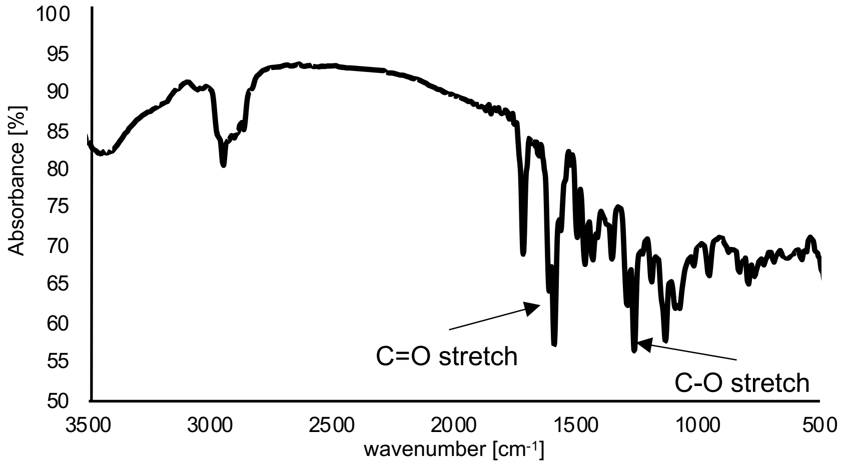

Figure S42. FT-IR spectrum of **FstCM*o*-F**.

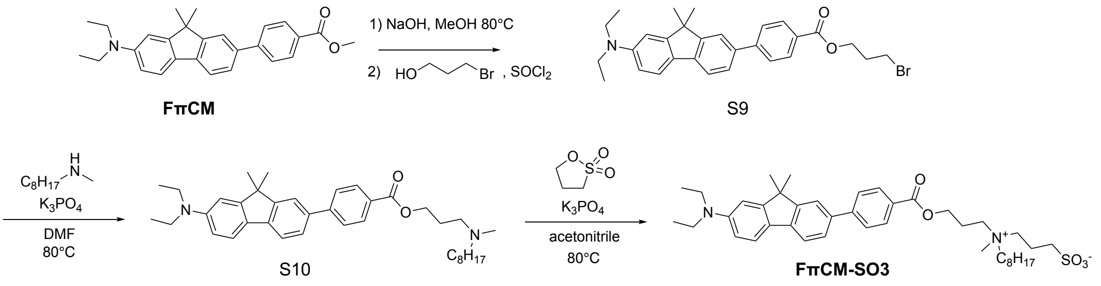

Figure S43. Synthetic rout to **FπCM-SO$_3$**.

4-(7-(diethylamino)-9,9-dimethyl-9*H*-fluoren-2-yl)benzoic acid (S9). 1) A mixture of **FπCM** (1.0 eq, 3.0 mmol, 1.20 g) and Sodium hydroxide (10 wt%, 5.0 g) was dissolved in 100 mL of ethanol (EtOH) under argon atmosphere. The reaction mixture was heated to 80°C and stirred for 12 hours, and then cooled to room temperature. The organic products were extracted with dichloromethane, and the organic layer was washed with water and brine. The combined organic layers were dried with $MgSO_4$, filtered, and then evaporated under reduced pressure to give a residue. The progress of the reaction was confirmed by TLC and the Product was used for the next reaction without purification. 2) A mixture of the product mentioned above was dissolved in 15 mL of 3-bromo-1-propanol and the mixture was cooled to 0°C and 3.0 mL of Thionyl chloride was added dropwise. After this operation, the reaction mixture was heated to 80°C and stirred for 16 h. The reaction mixture was quenched with water, and organic products were extracted with dichloromethane. The organic layer was washed with water and brine. The combined organic layers were dried with $MgSO_4$, filtered, and then evaporated under reduced pressure to give a residue. The obtained product contained a large amount of 3-bromo-1-propanol remained and was not able to be analyzed by NMR. The progress of the reaction was confirmed by TLC, and this residue was used without purification because it does not affect the next reaction. (**S9**)

3-(methyl(octyl)amino)propyl 4-(7-(diethylamino)-9,9-dimethyl-9*H*-fluoren-2-yl)benzoate (S10). A mixture of **S9**, *N*-octylamino-*N*-methylamine (6.97 mmol, 1.0 mL), and potassium phosphate (10 mmol, 2.24 g) was dissolved in DMF under argon atmosphere. The reaction mixture was heated to 100°C and stirred for 12 hours, and then cooled to room temperature. The reaction mixture was quenched with water. The organic products were extracted with dichloromethane, and the organic layer was washed with water and brine. The combined organic layers were dried with magnesium sulfate, filtered, and then evaporated under reduced pressure to give a residue. Purification by silica gel column chromatography

(eluent: hexane/ethyl acetate = 3/1 (v/v)) yielded **S10** containing 3-bromo-1-propanol (59.4 mol%) as a yellowish oil (Estimated yield: 0.40 g, 23.3% yield from the starting material: **FπCM**). **^{1}H NMR** (500 MHz, $CDCl_3$)δ 8.05 (d, J = 7.6 Hz, 2H, Ar**H**), 7.71 (d, J = 7.9 Hz, 2H, Ar**H**), 7.59-7.50 (m, 4H, Ar**H**), 6.72-6.66 (m, 2H, Ar**H**), 4.51 (t, J = 6.9 Hz, 2H, Ar**H**) 3.80 (s, 2H, $C\mathbf{H_2}$), 3.58-3.49 (m, 2H, $C\mathbf{H_2}$), 3.43 (t, J = 6.7 Hz, 4H, $NC\mathbf{H_2}$), 3.21 (s, 3H, $C\mathbf{H_3}$), 3.00 (m, 2H, $C\mathbf{H_2}$), 2.31 (m, 4H, $C\mathbf{H_2}$), 1.50 (s, 6H, $C\mathbf{H_3}$), 1.22 (t, J = 6.9 Hz, 14H, $C\mathbf{H_2}$ and $NC\mathbf{H_2}C\mathbf{H_3}$), 0.82 (t, J = 6.6 Hz, 3H, $C\mathbf{H_3}$) (**Figure S44**)

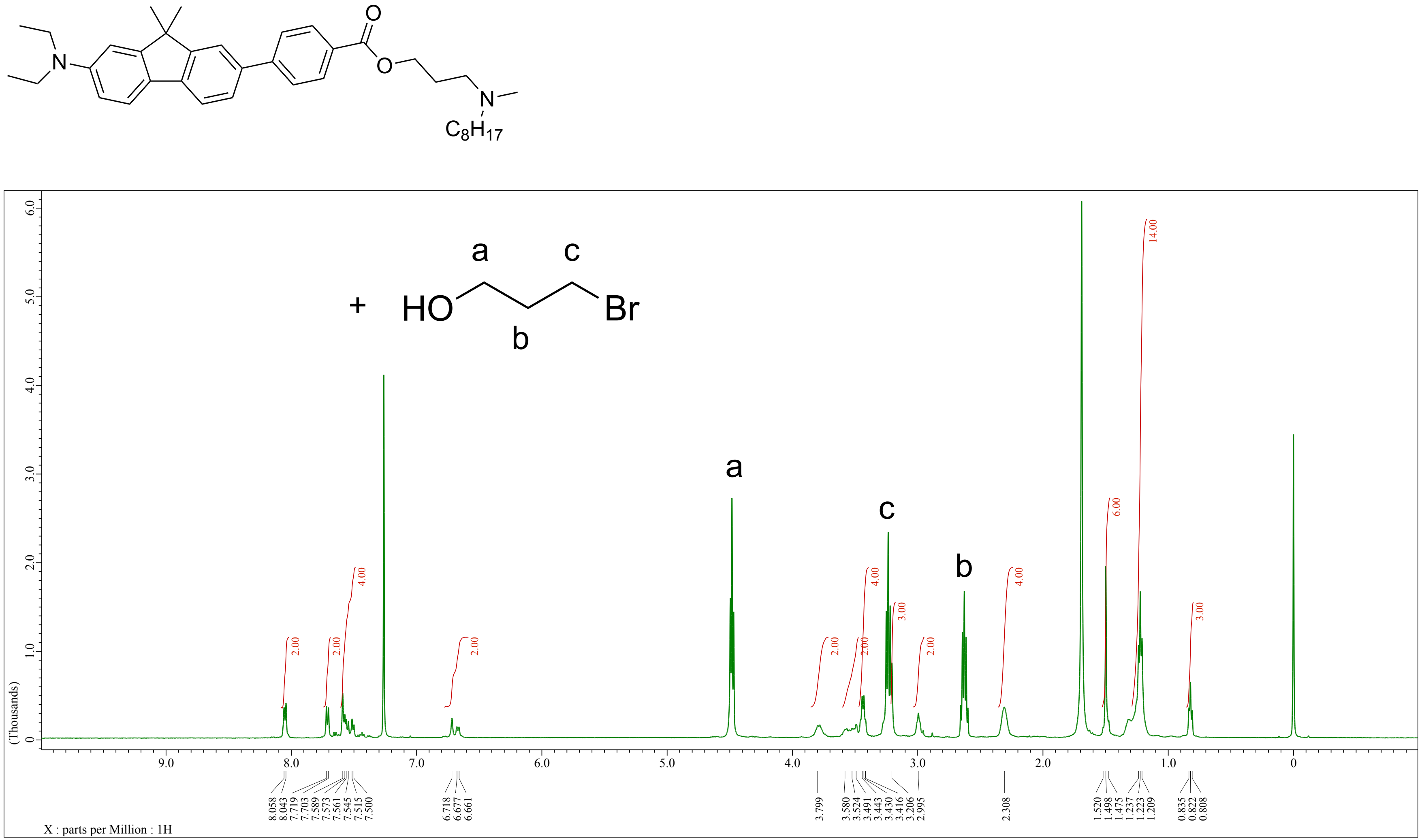

Figure S44. ¹H NMR (500 MHz, $CDCl_3$) spectrum of **S10**.

3-((3-((4-(7-(diethylamino)-9,9-dimethyl-9H-fluoren-2 yl)benzoyl)oxy)propyl)(methyl)(octyl)ammonio)propane-1-sulfonate (FπCM-SO_3). A mixture of **S10** (1.0 eq, 0.70 mmol, 0.40 g), 1,3-propanesultone (4.30 eq, 3.0 mmol, 0.366 g), and potassium phosphate (12.9 eq, 9.0 mmol, 2.01 g) was dissolved in acetonitrile under argon atmosphere. The reaction mixture was heated to 80°C and stirred for 12 hours, and then cooled to room temperature. The reaction mixture was quenched with water. The organic products were extracted with dichloromethane, and the organic layer was washed with water and brine. The combined organic layers were dried with magnesium sulfate, filtered, and then evaporated under reduced pressure to give a residue. Purification by silica gel column chromatography (eluent: chloroform/MeOH = 6/1 (v/v)) yielded **FπCM-ammonium salt** as a yellow solid (0.16 g, 33.0% yield). **^{1}H NMR** (500 MHz, $CDCl_3$) δ 8.06 (d, J = 8.2 Hz, 2H, Ar**H**), 7.71 (d, J = 8.5 Hz, 2H, Ar**H**), 7.67-7.59 (m, 4H, Ar**H**), 7.54-7.36 (m, 2H, Ar**H**), 4.50 (m, 2H, COOC**H**$_2$), 3.82-3.79 (m, 2H, C**H**$_2$), 3.59-3.45 (m, 4H, N(C**H**$_2$)), 3.21 (s, 3H, N^+C**H**$_3$), 3.02-2.98 (m, 2H, C**H**$_2$), 2.31 (m, 4H, C**H**$_2$), 1.81 (m, 6H, N^+C**H**$_2$), 1.70 (m, 2H, C**H**$_2$), 1.51 (s, 6H, C**H**$_3$), 1.23 (m, 14H, NCH_2C**H**$_3$+C**H**$_3$), 0.82 (t, J = 6.9 Hz, 3H, C**H**$_3$) (**Figure S45**) **^{13}C NMR** was not able to detect clearly due to solubility issue. **HRMS** (FAB+) m/z Calcd. For $C_{41}H_{59}N_2O_5S$ $[M]^+$: 691.4145, Found: 691.4135. (**Figure S46**) **FT-IR** (**Figure S47**) **m.p.**: 93.4-95.2°C.

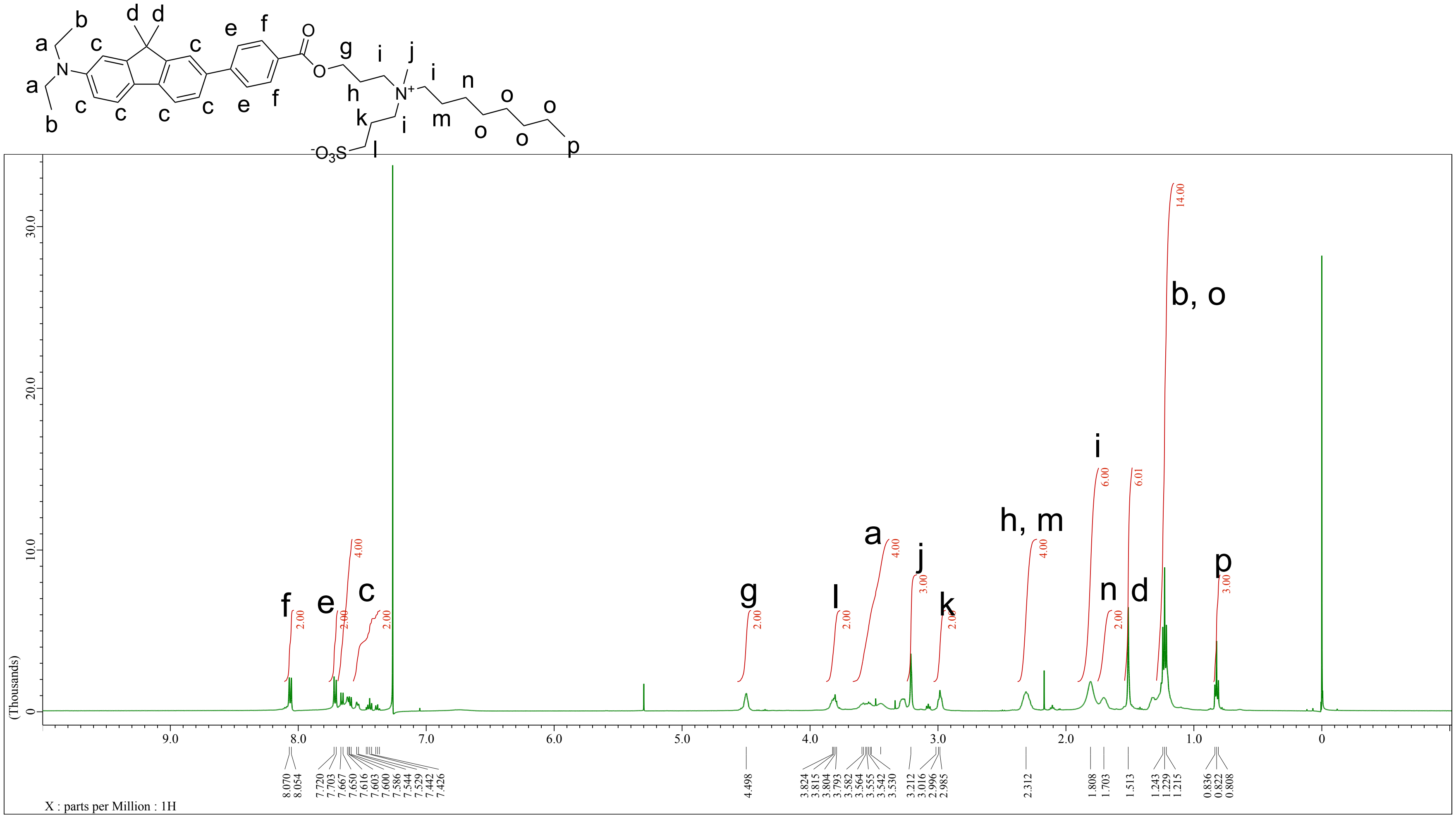

Figure S45. ^{1}H NMR (500 MHz, DMSO-d6) spectrum of **FπCM-SO$_3$**.

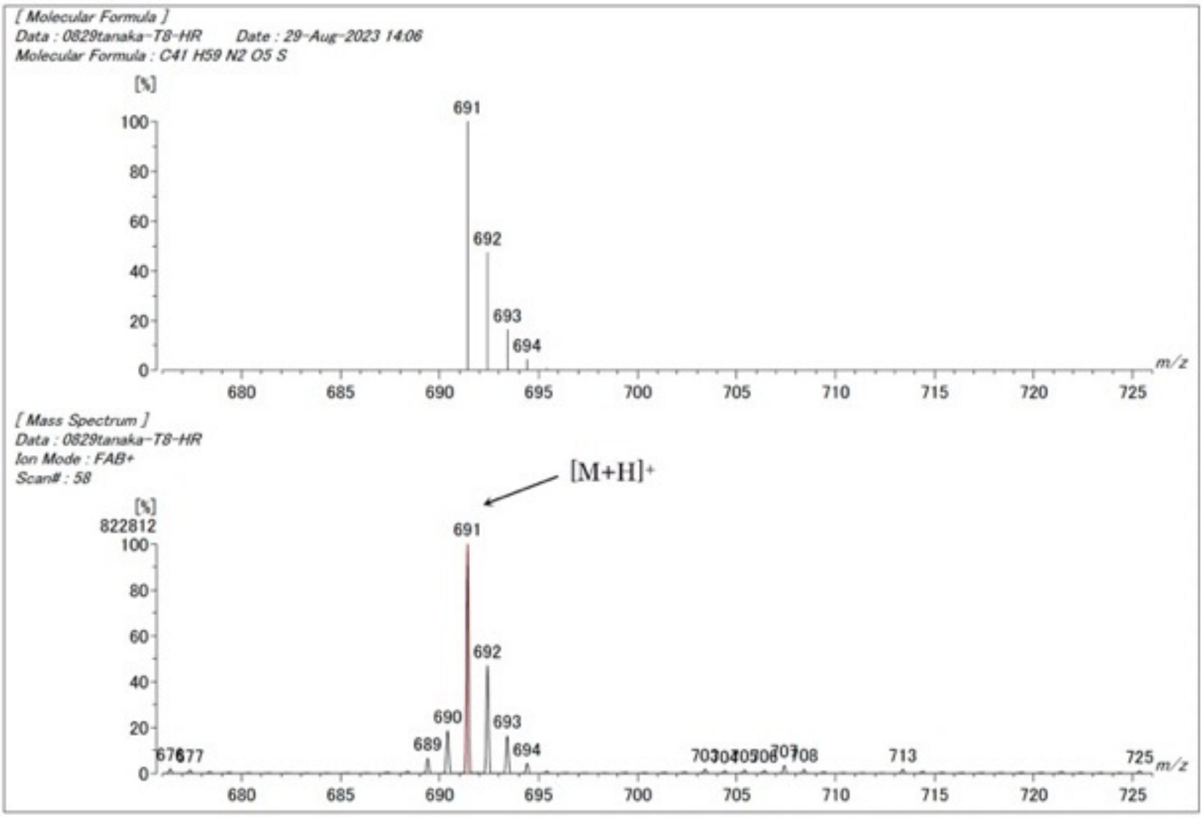

Observed m/z	Int%	Err[ppm / mmu]	U.S.	Composition
1 691.4135	100.00	-1.4 / -1.0	20.0	C40 H53 N9 S
2		+2.5 / +1.7	15.5	C37 H55 N8 O3 S
3		+0.5 / +0.4	15.0	C39 H57 N5 O4 S
4		-1.4 / -1.0	14.5	C41 H59 N2 O5 S
5		+2.5 / +1.7	10.0	C38 H61 N O8 S
6		-0.2 / -0.1	2.5	C25 H59 N10 O10 S

Figure S46. MS spectrum of **FπCM-SO$_3$**.

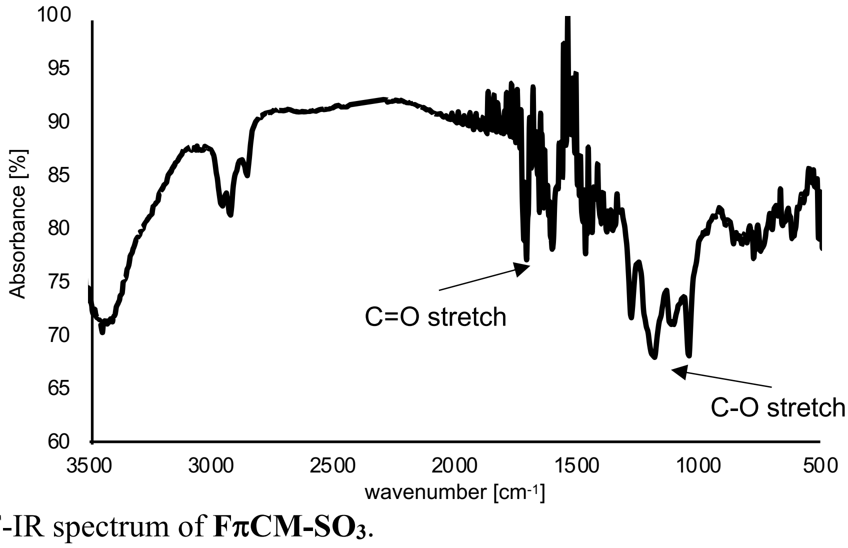

Figure S47. FT-IR spectrum of **FπCM-SO$_3$**.

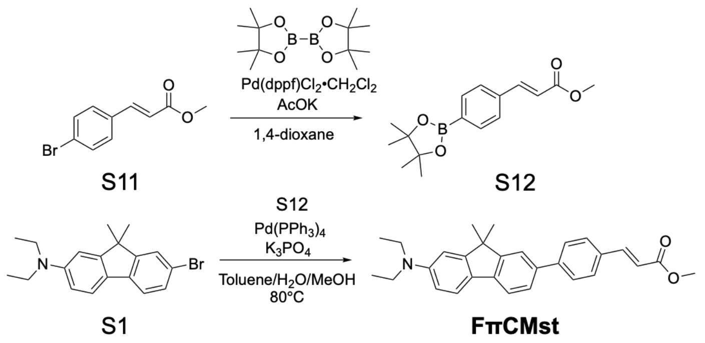

Figure S48. Synthetic rout to **FπCMst**.

methyl (*E*)-3-(4-(4,4,5,5-tetramethyl-1,3,2-dioxaborolan-2-yl)phenyl)acrylate (**S12**).

A mixture of methyl (*E*)-3-(4-bromophenyl)acrylate (**S11**) (1.0 eq, 5.0 mmol, 1.20 g), bis(pinacolate)diborone (1.1 eq, 5.5 mmol, 1.40 g), Pd(dppf)Cl_2•CH_2Cl_2 (3.0 mol%, 0.15 mmol, 0.12 g) and potassium acetate (3.0 eq, 15 mmol, 1.47 g) was dissolved in 15 mL of 1,4-dioxane under argon atmosphere. The reaction mixture was heated to 80°C and stirred for 16 hours, and then cooled to room temperature. The reaction mixture was quenched with water. The organic products were extracted with dichloromethane, and the organic layer was washed with water and brine. The combined organic layers were dried with $MgSO_4$, filtered, and then evaporated under reduced pressure to give a residue. Purification by silica gel column chromatography (eluent: hexane/ethyl acetate = 6/1 (v/v)) yielded **S12** as a colorless solid (1.41 g, 98.0% yield). **^{1}H NMR** (500 MHz, $CDCl_3$) δ 7.82 (d, J = 8.2 Hz, 2H, Ar**H**), 7.73 (d, J = 15.8 Hz, 1H, C**H**=C**H**), 7.52 (d, J = 8.2 Hz, 2H, Ar**H**), 6.49 (d, J = 15.9 Hz, 1H, C**H**=C**H**), 3.81 (s, 3H, OC**H**$_3$), 1.35 (s, 12H, C**H**$_3$) ppm. (**Figure S49**)

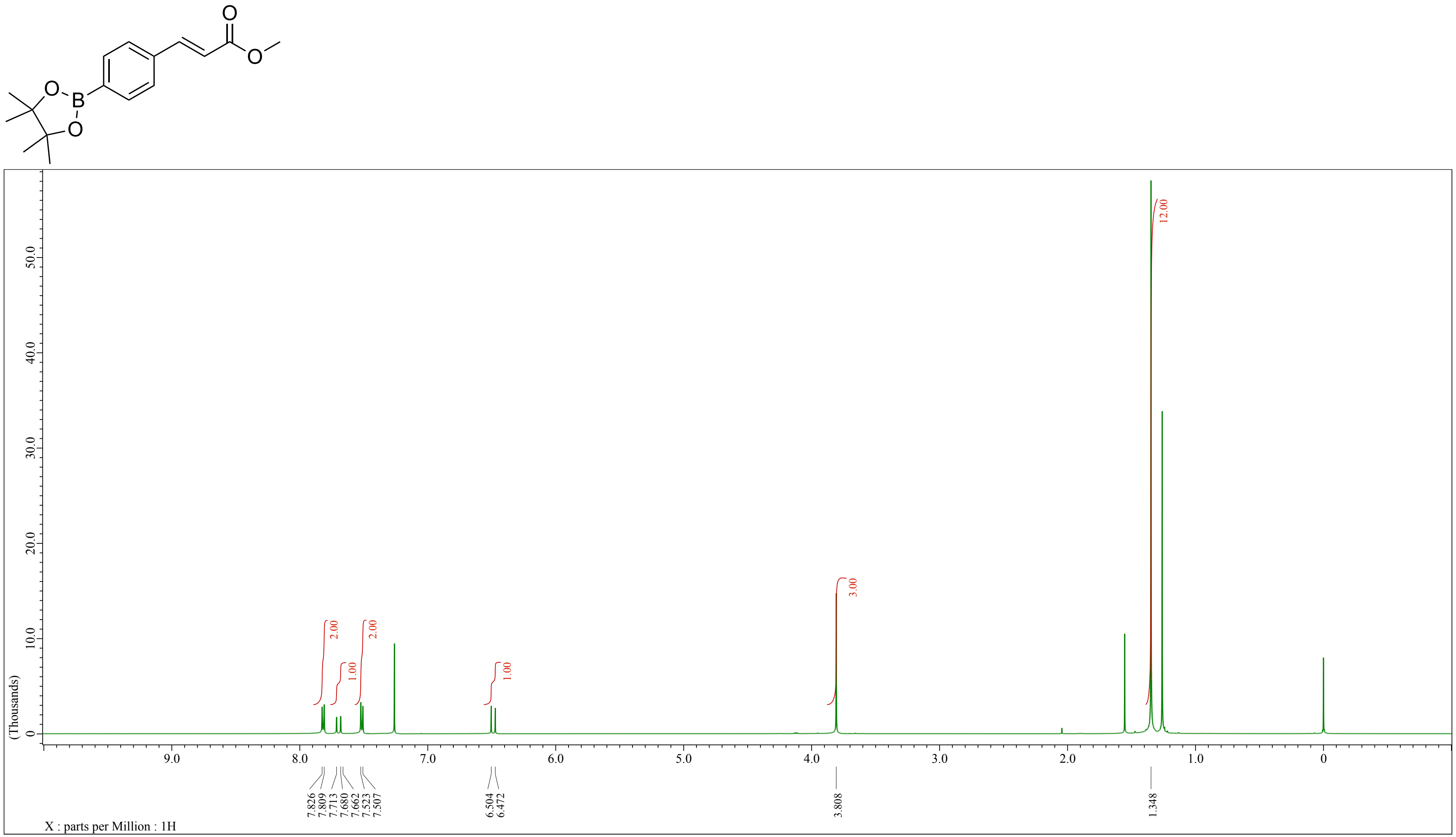

Figure S49. ^{1}H NMR (500 MHz, $CDCl_3$) spectrum of **S12**.

methyl (*E*)-3-(4-(7-(diethylamino)-9,9-dimethyl-9*H*-fluoren-2-yl)phenyl)acrylate (FπCMst). A mixture of **S1** (1.0 eq, 1.50 mmol, 0.52 g), **S12** (1.5 eq, 2.3 mmol, 0.66 g), $Pd(PPh_3)_4$ (5.0 mol%, 0.075 mmol, 0.09 g) and potassium phosphate (3.0 eq, 4.5 mmol, 1.0 g) was dissolved in 5/1/2 (v/v/v) solution of toluene, high purity water and MeOH under argon atmosphere. The reaction mixture was heated to 100°C and stirred for 12 hours, and then cooled to room temperature. The reaction mixture was quenched with water. The organic products were extracted with dichloromethane, and the organic layer was washed with water and brine. The combined organic layers were dried with $MgSO_4$, filtered, and then evaporated under reduced pressure to give a residue. Purification by silica gel column chromatography (eluent: hexane/ethyl acetate = 5/1 (v/v)) and recrystallization from hexane/dichloromethane (5/1) yielded **FπCMst** as a greenish solid (0.48 g, 75% yield). **^{1}H NMR** (500 MHz, $CDCl_3$) δ 7.74 (d, J = 15.9 Hz, 1H, C**H**=C**H**), 7.68 (dd, J = 6.6, 1.7 Hz, 2H, Ar**H**), 7.62-7.57 (m, 5H, Ar**H**), 7.53 (dd, J = 7.8, 1.7 Hz, 1H, Ar**H**), 6.73 (d, J = 2.4 Hz, 1H, Ar**H**), 6.68 (dd, J = 8.5, 2.4 Hz, 1H, Ar**H**), 6.48 (d, J = 16.2 Hz, 1H, C**H**=C**H**), 3.82 (s, 3H, OC**H**$_3$), 3.46-3.42 (m, 4H, NC**H**$_2$), 1.51 (s, 6H, C**H**$_3$), 1.24-1.21 (m, 6H, C**H**$_3$) ppm. (**Figure S50**) **^{13}C NMR** (100 MHz, $CDCl_3$) δ 167.7, 156.0, 153.6, 148.1, 144.7, 144.0, 140.2, 136.8, 132.8, 128.7, 127.4, 127.2, 126.7, 126.1, 121.3, 120.9, 118.8, 117.2, 110.9, 105.8, 51.8, 46.9, 44.8, 27.7, 12.8 ppm. (**Figure S51**) **HRMS** (EI+) m/z Calcd. For $C_{29}H_{31}NO_2$ $[M]^+$: 425.2355, Found: 425.2355 (**Figure S52**) **FT-IR** (**Figure S53**) **m.p.**: 138.5-139.1°C.

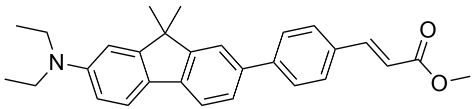

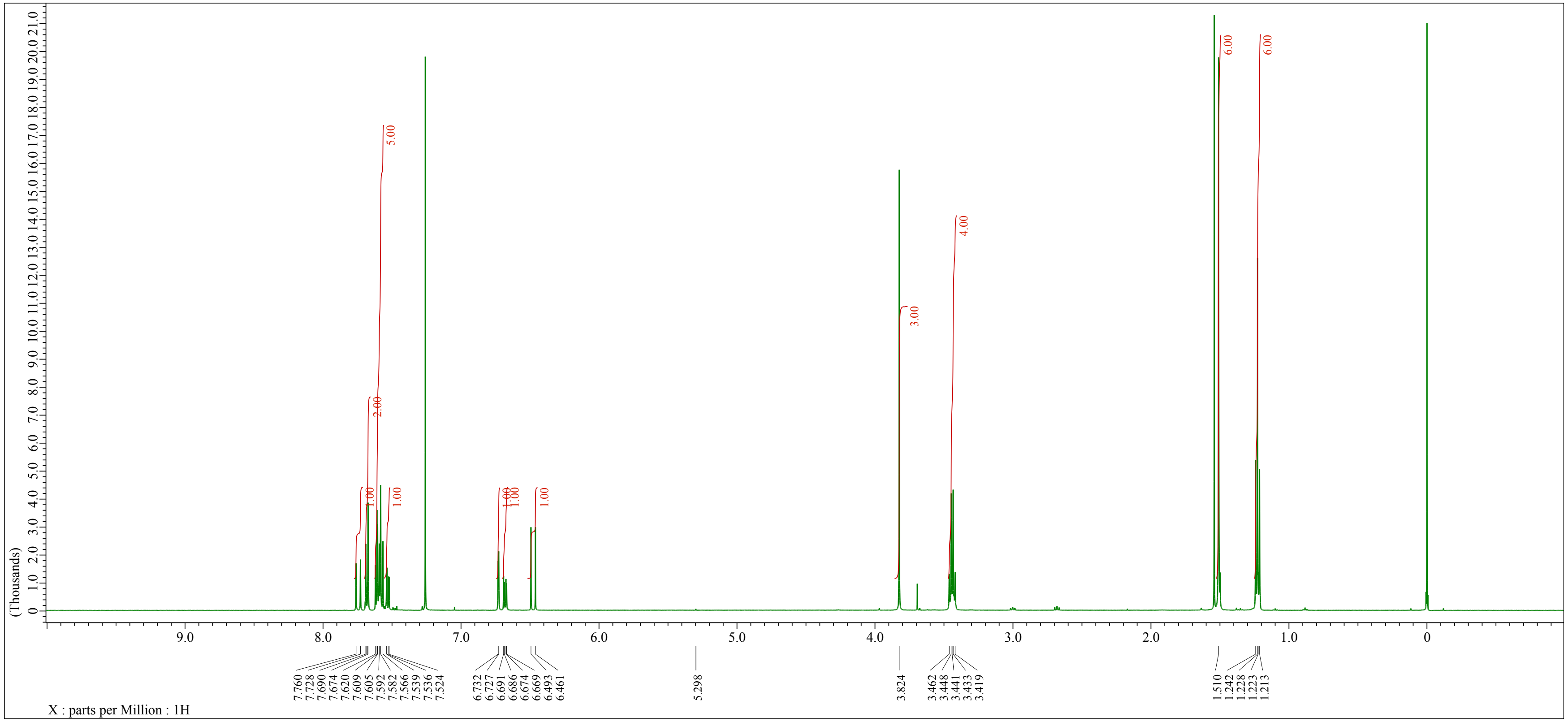

Figure S50. ^{1}H NMR (500 MHz, $CDCl_3$) spectrum of **FπCMst**.

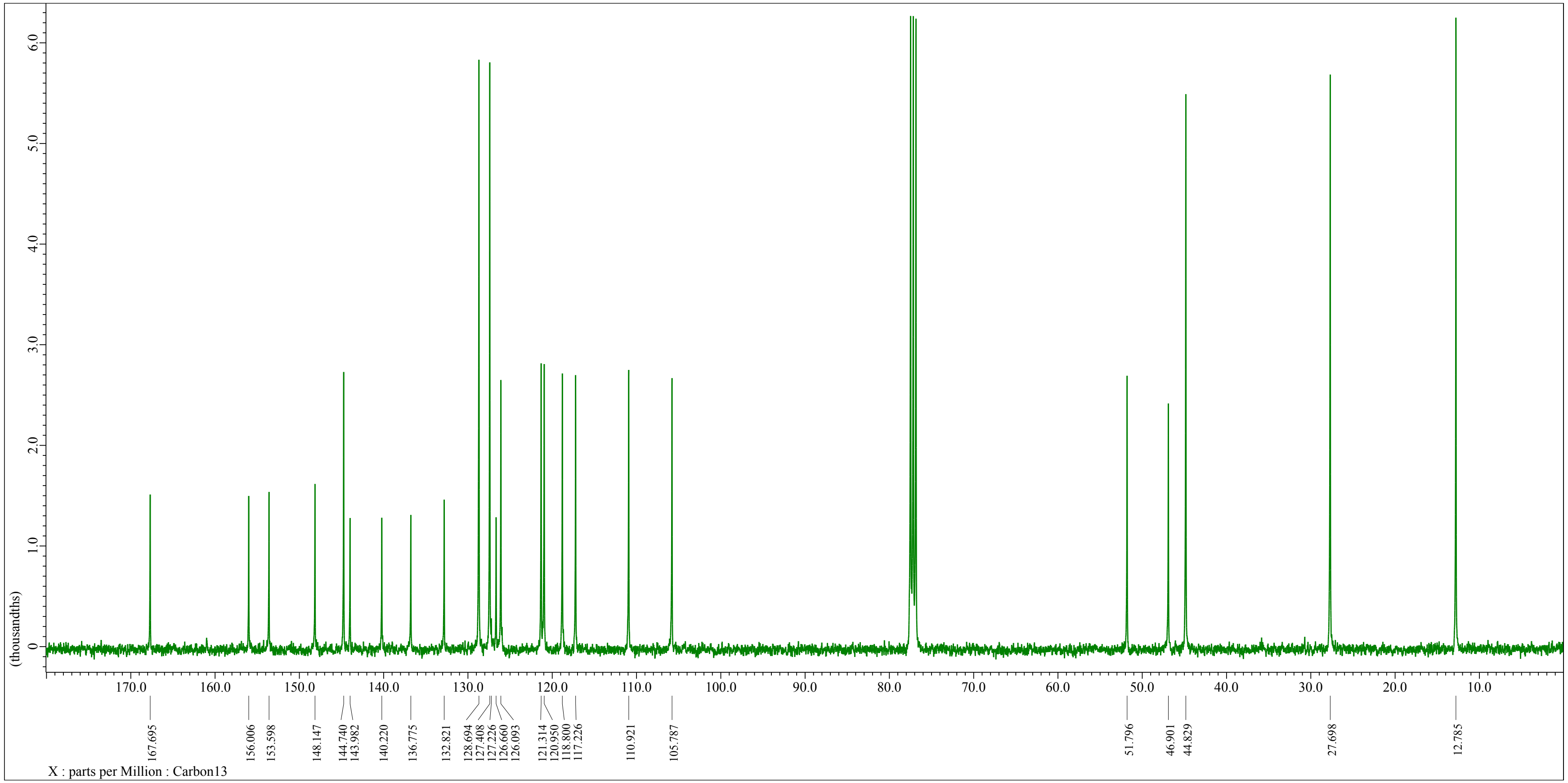

Figure S51. ^{13}C NMR (100 MHz, $CDCl_3$) spectrum of **FπCMst**.

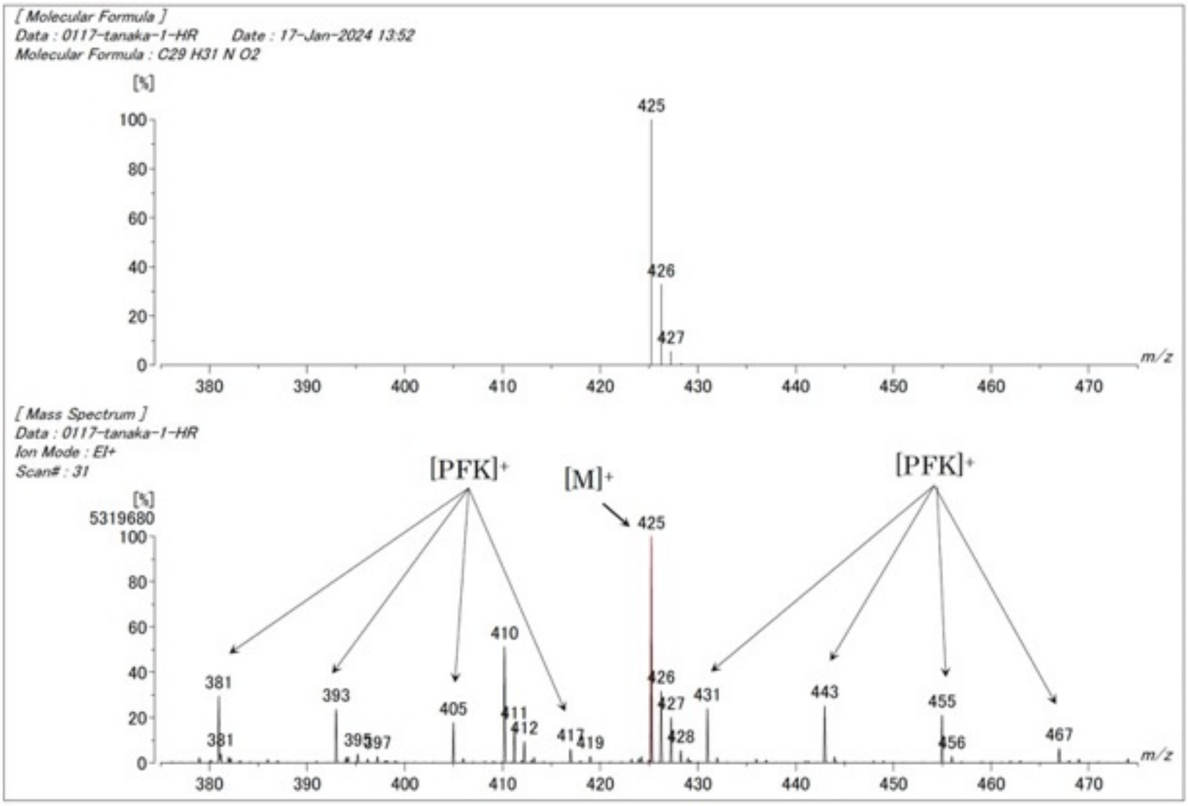

	Observed m/z	Int%	Err[ppm / mmu]	U.S.	Composition
1	425.2359	100.00	+4.1 / +1.8	15.5	C27 H29 N4 O
2			+1.0 / +0.4	15.0	C29 H31 N O2
3			-3.4 / -1.4	7.5	C16 H29 N10 O4
4			+3.0 / +1.3	3.0	C13 H31 N9 O7
5			-0.2 / -0.1	2.5	C15 H33 N6 O8
6			-3.4 / -1.4	2.0	C17 H35 N3 O9

Figure S52. MS spectrum of **FπCMst**.

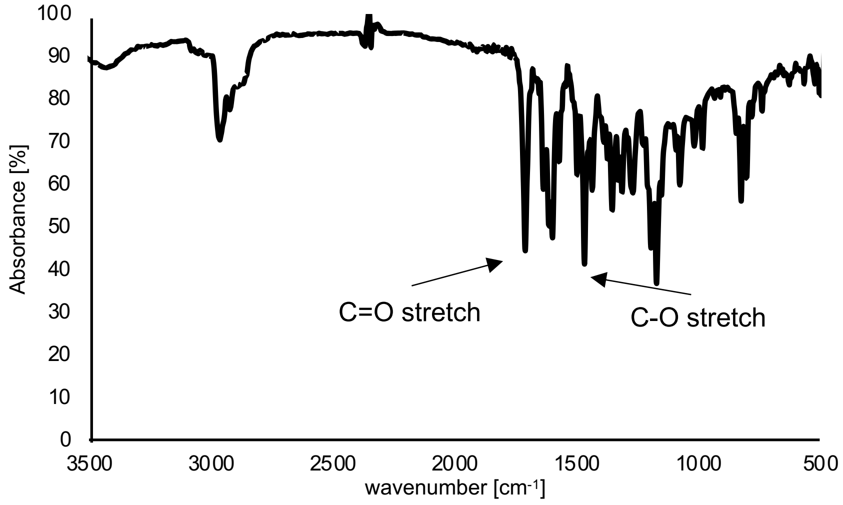

Figure S53. FT-IR spectrum of **FπCMst**.

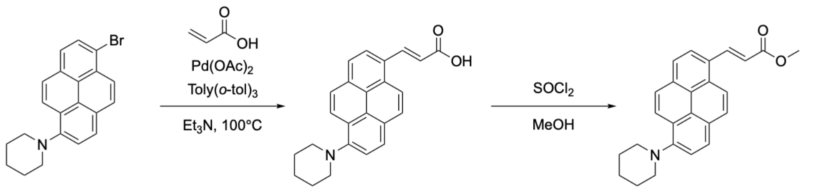

Figure S54. Synthetic rout to **PCMst**.

(*E*)-3-(6-(piperidin-1-yl)pyren-1-yl)acrylic acid (**S13**). A mixture of **S3** (1.0 eq, 1.5 mmol, 0.55 g), acrylic acid (1.2 eq, 1.8 mmol, 0.13 g), palladium(II) acetate (5.0 mol%, 0.075 mmol, 0.020 g) and tri(*o*-tolyl)phosphine (10 mol%, 0.15 mmol, 0.045 g) was dissolved in triethylamine (5 mL) and under argon atmosphere in the pressure-resistant tube and stirred for 12h at 100°C. After cooling to r.t, the reaction mixture was quenched with water, extracted with dichloromethane, and washed with water and brine. The combined organic layers were dried with $MgSO_4$, filtered, and then evaporated under reduced pressure to give a residue. The progress of the reaction was confirmed by TLC and the Product was used for next reaction without purification.

methyl (*E*)-3-(6-(piperidin-1-yl)pyren-1-yl)acrylate (**PCMst**). **S13** was dissolved in MeOH and stirred at 0°C. 5 mL of thionyl chloride was added dropwise to the reaction mixture, stirred for 30 min at room temperature. The reaction mixture was heated to 100°C and stirred for 24 hours, and then cooled to room temperature. The organic products were extracted with dichloromethane, and the organic layer was washed with water and brine. The combined organic layers were dried with $MgSO_4$, filtered, and then evaporated under reduced pressure to give a residue. Purification by silica gel column chromatography (eluent: hexane/ethyl acetate = 3/1 (v/v)) and recrystallization from hexane/dichloromethane (5/1) yielded **PCMst** as an orange solid (0.37 g). **^{1}H NMR** (500 MHz, $CDCl_3$) δ 8.82 (d, J = 15.6 Hz, 1H, C**H**=C**H**), 8.46 (d, J = 9.2 Hz, 1H, Ar**H**), 8.33 (d, J = 9.5 Hz, 1H, Ar**H**), 8.23 (d, J = 8.2 Hz, 1H, Ar**H**), 8.14 (d, J = 8.2 Hz, 1H, Ar**H**), 8.08 (dd, J = 8.7, 5.3 Hz, 2H, Ar**H**), 8.03 (d, J = 9.2 Hz, 1H, Ar**H**), 7.74 (d, J = 8.2 Hz, 1H, Ar**H**), 6.69 (d, J = 15.9 Hz, 1H, C**H**=C**H**), 3.89 (s, 3H, COOC**H**$_3$), 3.21 (s, 4H, NC**H**$_2$), 1.96-1.91 (m, 4H, C**H**$_2$), 1.72 (s, 2H, C**H**$_2$) ppm. (**Figure S55**) **^{13}C NMR** (100 MHz, $CDCl_3$) δ 167.8, 150.2, 141.9, 133.1, 130.4, 128.7, 127.4, 126.6, 126.4, 126.3, 126.0, 125.6, 125.1, 124.6, 124.4, 124.2, 120.5, 119.2, 117.5, 55.2, 51.9, 26.8, 24.6 ppm. (**Figure S56**) **HRMS** (EI+) m/z Calcd. For $C_{25}H_{23}NO_2$ $[M]^+$: 369.1729, Found: 369.1729 (**Figure S57**) **FT-IR** (**Figure S58**) **m.p.**: 161.0-162.4°C.

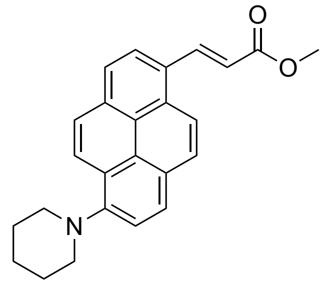

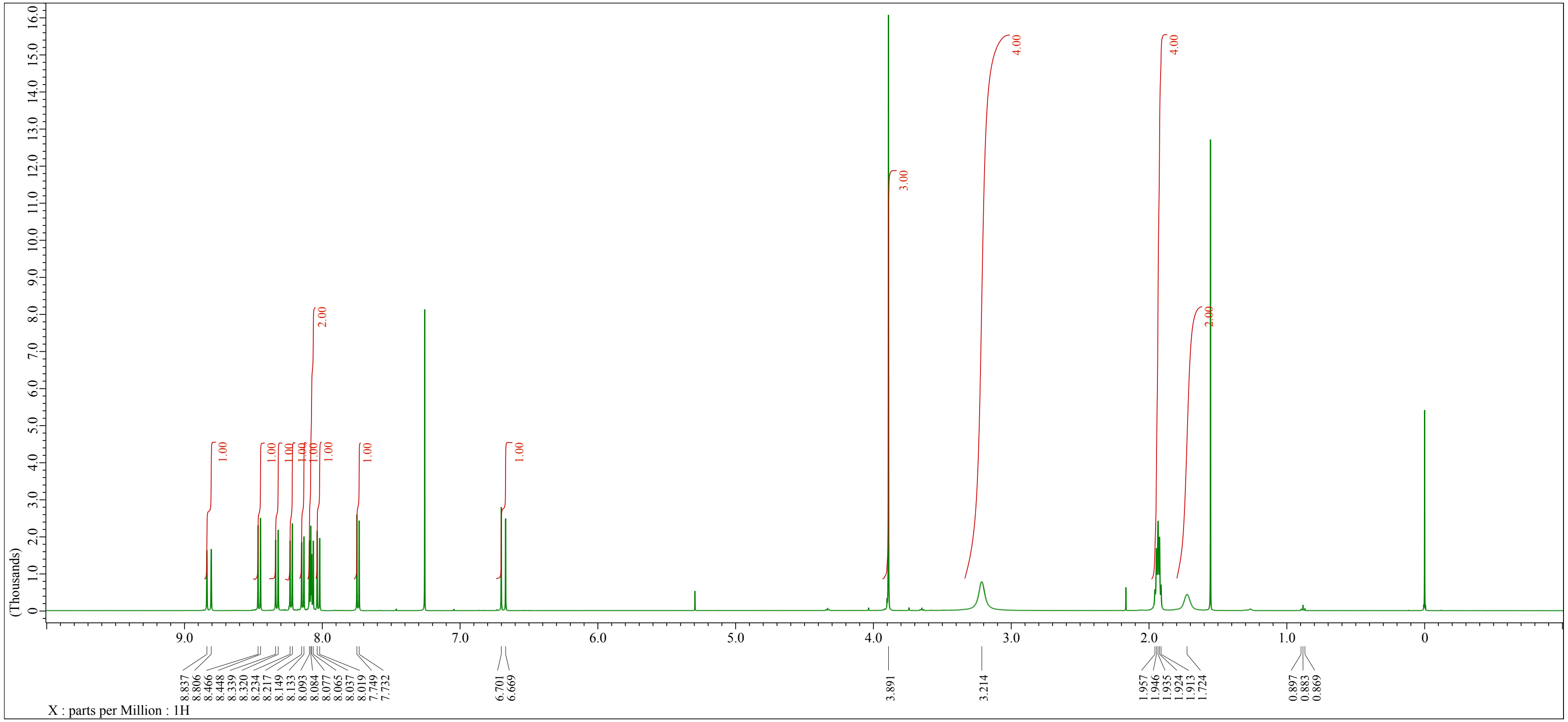

Figure S55. ^{1}H NMR (500 MHz, $CDCl_3$) spectrum of **PCMst**.

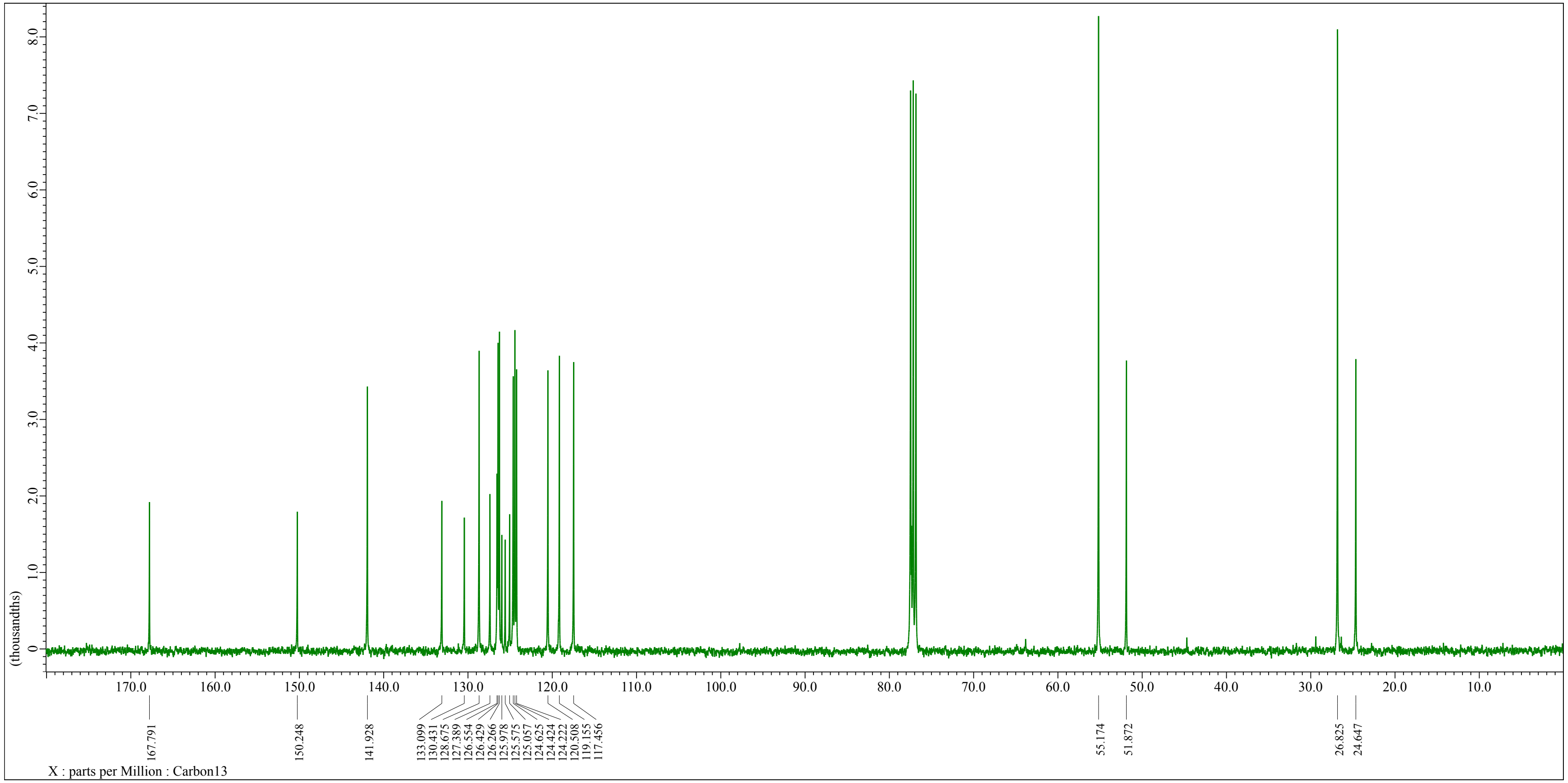

Figure S56. ^{13}C NMR (100 MHz, $CDCl_3$) spectrum of **PCMst**.

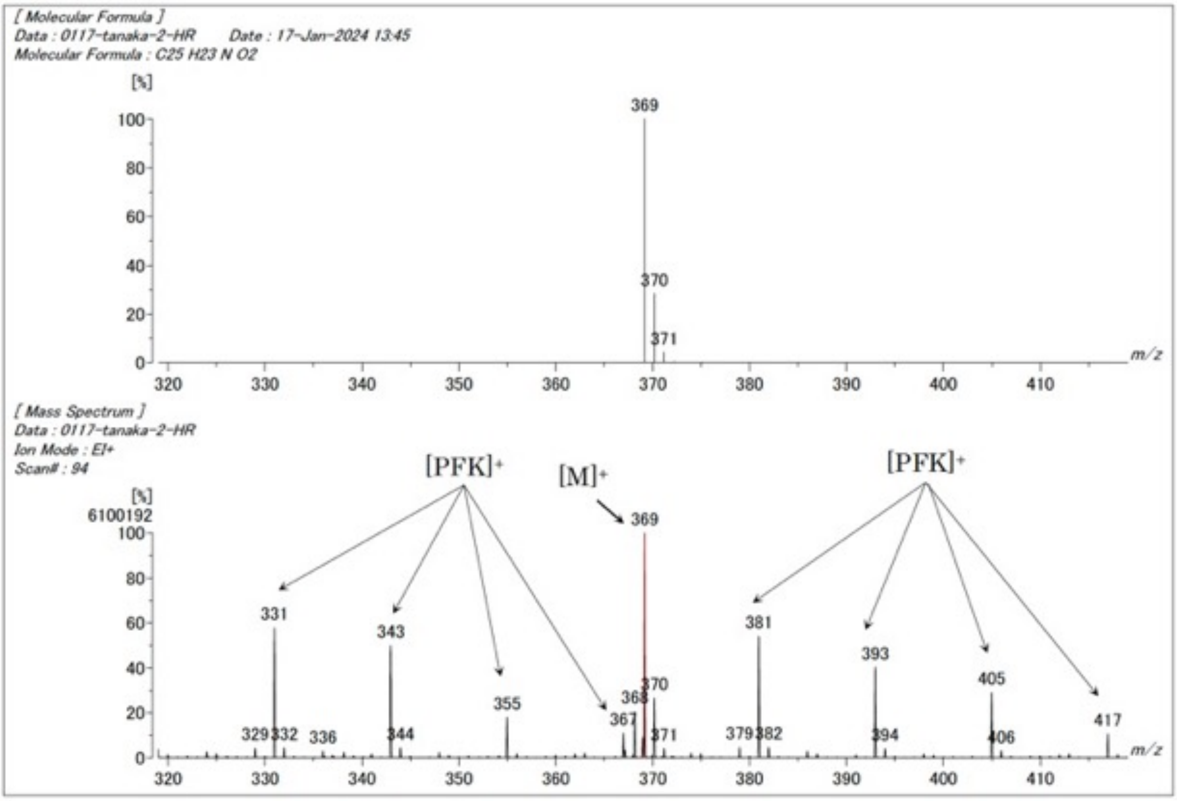

Observed m/z	Int%	Err[ppm / mmu]	U.S.	Composition
1 369.1726	100.00	+2.9 / +1.1	15.5	C23 H21 N4 O
2		-0.8 / -0.3	15.0	C25 H23 N O2 ←
3		+1.5 / +0.6	3.0	C9 H23 N9 O7
4		-2.1 / -0.8	2.5	C11 H25 N6 O8

Figure S57. MS spectrum of **PCMst**.

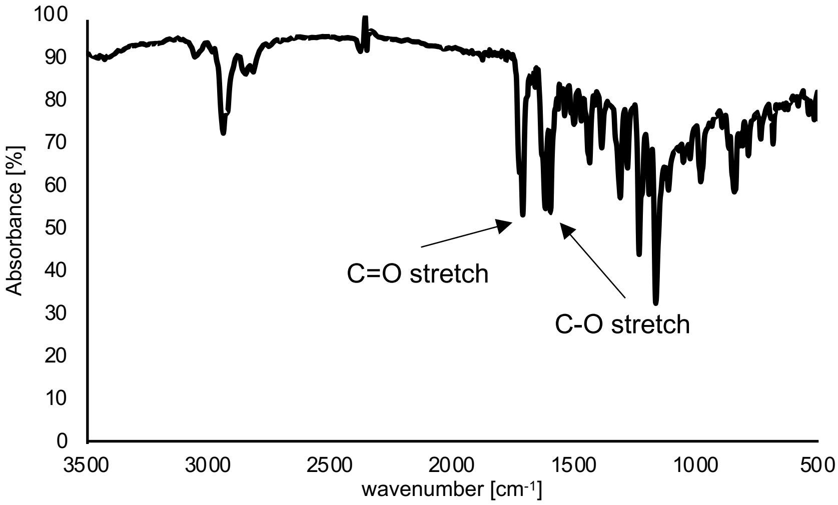

Figure S58. FT-IR spectrum of **PCMst**.

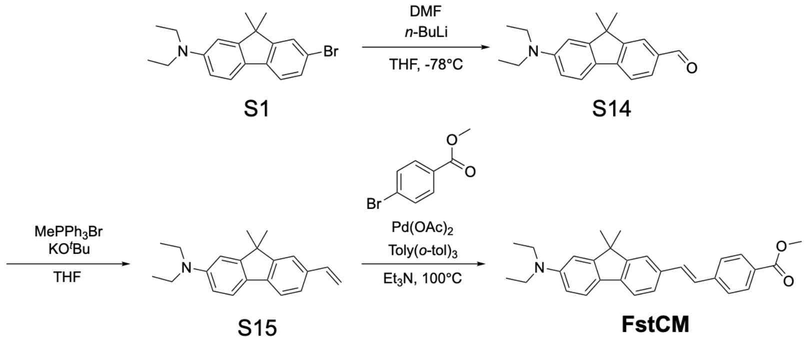

Figure S59. Synthetic rout to **FstCM**.

7-(diethylamino)-9,9-dimethyl-9*H*-fluorene-2-carbaldehyde (S14). **S1** (1.0 eq, 5.0 mmol, 1.72 g) was dissolved in 20 mL of dehydrated tetrahydrofuran under argon atmosphere. The reaction mixture was cooled to -78°C, and 2.6 M *n*-butyl lithium (1.5 eq, 7.5 mmol, 2.9 mL) was added dropwise. The reaction mixture was stirred for 30 min at -78°C and then, heated up to r.t. *N,N*-dimethylformamide (DMF) (1.10 eq, 5.50 mmol, 0.40 mL) was added to the reaction mixture. After 2 hours, the reaction mixture was extracted with dichloromethane, washed with water and brine, and combined organic layers were dried with magnesium sulfate, filtered, and then evaporated under reduced pressure to give residue. Purification by silica gel column chromatography (eluent: hexane/ethyl acetate = 3/1 (v/v)) yielded **S14** as a yellow solid (1.21 g, 83% yield). **^{1}H NMR** (500 MHz, $CDCl_3$) δ 9.97 (s, 1H,), 7.87 (d, J = 0.9 Hz, 1H, Ar**H**), 7.77 (dd, J = 7.9, 1.5 Hz, 1H, Ar**H**), 7.64-7.60 (m, 2H, Ar**H**), 6.69-6.68 (m, 2H, Ar**H**), 3.45 (q, J = 7.1 Hz, 4H, NC**H**$_2$), 1.49 (s, 6H, C**H**$_3$), 1.23 (t, J = 7.2 Hz, 6H, C**H**$_3$) ppm. (**Figure S60**)

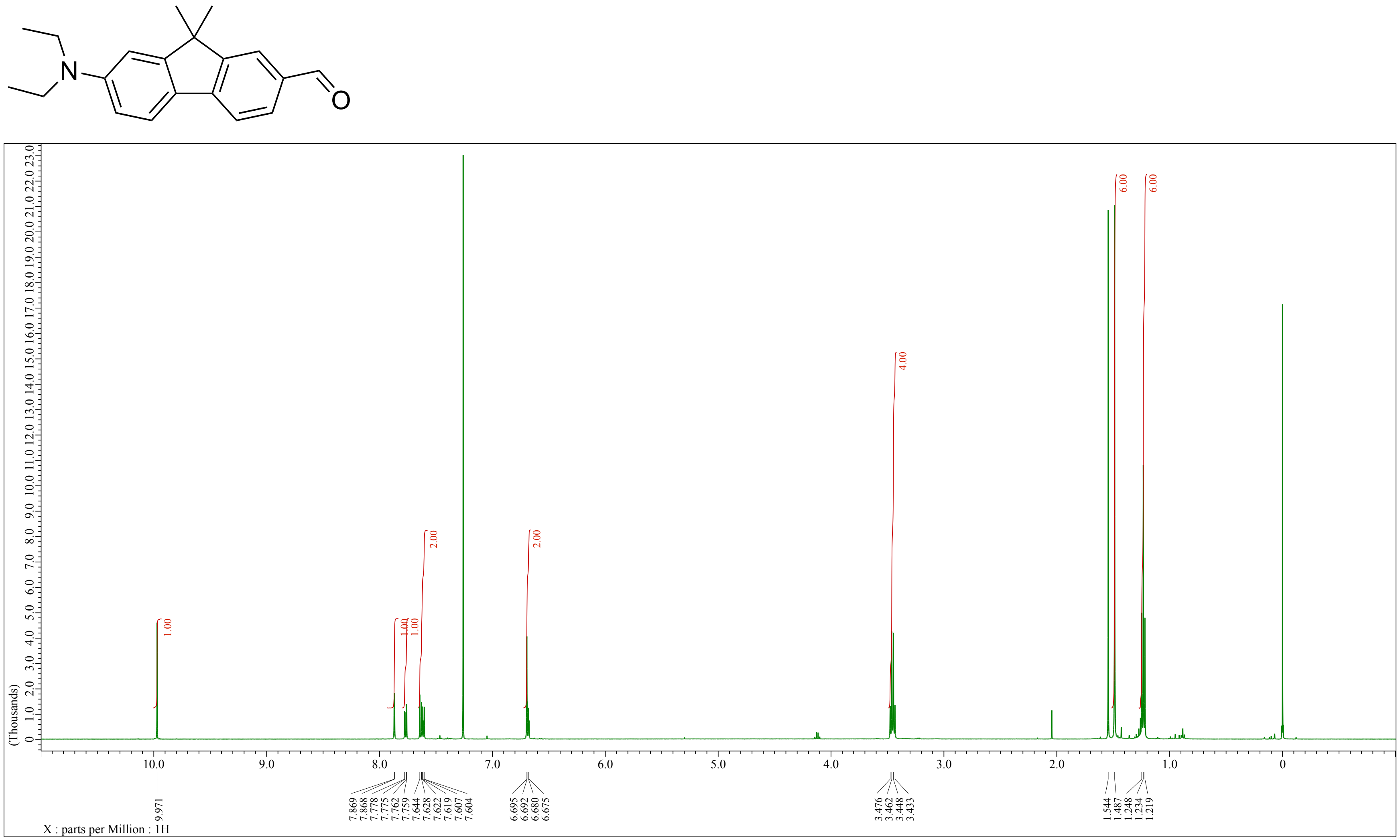

Figure S60. ^{1}H NMR (500 MHz, $CDCl_3$) spectrum of **S14**.

***N*,*N*-diethyl-9,9-dimethyl-7-vinyl-9*H*-fluoren-2-amine** (**S15**). Methyl triphenyl phosphonium bromide (1.2 eq, 5.0 mmol, 1.78 g) was dissolved in THF (20 mL) under an argon atmosphere. The solution was cooled to 0 °C, and then KO^{t}Bu (1.5 eq, 6.22 mmol, 0.70 g) was added. Following stirring for 10 minutes, **S14** (1.0 eq, 4.20 mmol, 1.21 g) in 5 mL of dehydrated tetrahydrofuran was added and then warmed to room temperature and stirred for 2 hours. The reaction mixture was quenched with sat. NH_4Cl aq. and organic products were extracted with dichloromethane. The organic layer was washed with water and brine. The combined organic layers were dried with $MgSO_4$, filtered, and then evaporated under reduced pressure to give a residue. Purification by column chromatography on silica (hexane/dichloromethane = 3:1 (v/v)) gave **S15** as a yellowish oil. (0.97 g, 80% yield); **^{1}H NMR** (500 MHz, $CDCl_3$) δ 7.51 (dd, J = 18.2, 8.1 Hz, 2H, Ar**H**), 7.41 (s, 1H, Ar**H**), 6.77 (dd, J = 17.5, 10.8 Hz, 1H, C**H**=C**H**$_2$), 6.71 (s, 1H, Ar**H**), 6.66 (dd, J = 8.5, 2.4 Hz, 1H, Ar**H**), 5.74 (d, J = 16.8 Hz, 1H, C**H**=C**H**$_2$), 5.18 (d, J = 10.1 Hz, 1H, Ar**H**), 3.42 (q, J = 7.0 Hz, 4H, NC**H**$_2$), 1.47 (s, 6H, C**H**$_3$), 1.21 (t, J = 7.0 Hz, 6H, CH_3) ppm. (**Figure S61**)

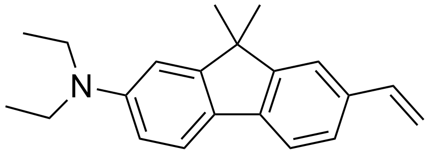

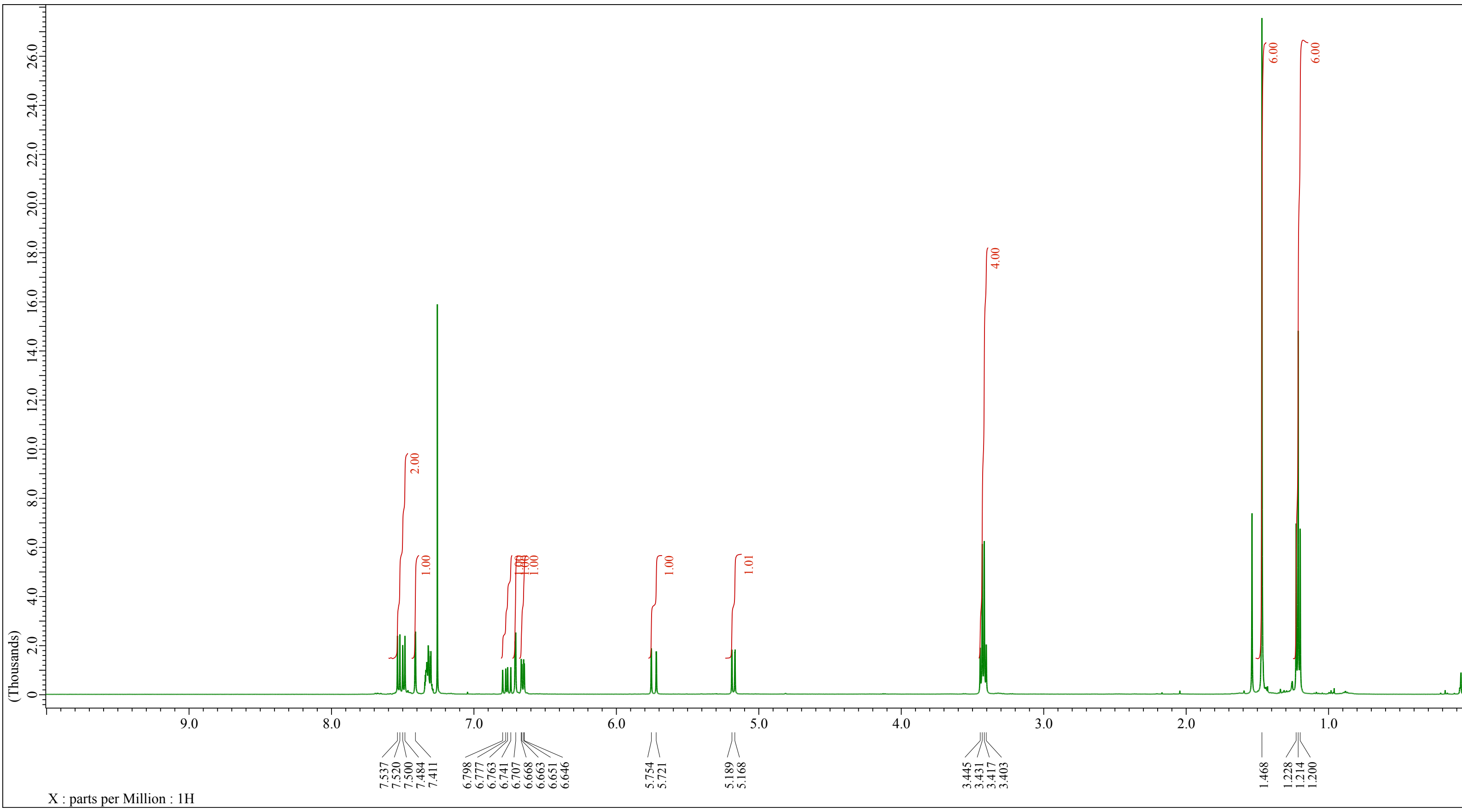

Figure S61. ^{1}H NMR (500 MHz, $CDCl_3$) spectrum of **S15**.

methyl (*E*)-4-(2-(7-(diethylamino)-9,9-dimethyl-9*H*-fluoren-2-yl)vinyl)benzoate (**FstCM**). A mixture of **S15** (1.0 eq, 1.50 mmol, 0.44 g), Methyl 4-boromobenzoate (1.0 eq, 1.50 mmol, 0.32 g), palladium(II) acetate (5.0 mol%, 0.075 mmol, 0.020 g) and tri(*o*-tolyl)phosphine (10 mol%, 0.15 mmol, 0.045 g) was dissolved in triethylamine (5 mL) and under argon atmosphere in the pressure-resistant tube and stirred for 12h at 100°C. After cooling to r.t, the reaction mixture was quenched with water, extracted with dichloromethane, and washed with water and brine. The combined organic layers were dried with $MgSO_4$, filtered, and then evaporated under reduced pressure to give a residue. Purification by silica gel column chromatography (eluent: hexane/ethyl acetate = 5/1 (v/v)) and recrystallization from hexane/dichloromethane (5/1) yielded **FπCMst** as a greenish solid (0.51 g, 80% yield). **^{1}H NMR** (500 MHz, $CDCl_3$) δ 8.02 (d, J = 8.5 Hz, 2H, Ar**H**), 7.58-7.53 (m, 5H, Ar**H**), 7.43 (dd, J = 8.1, 1.4 Hz, 1H, Ar**H**), 7.29 (d, J = 16.2 Hz, 1H, C**H**=C**H**), 7.13 (d, J = 16.2 Hz, 1H, C**H**=C**H**), 6.72 (d, J = 2.4 Hz, 1H, Ar**H**),

6.67 (dd, *J* = 8.5, 2.4 Hz, 1H, Ar**H**), 3.90 (s, 3H, COOC**H**$_3$), 3.44 (q, *J* = 7.0 Hz, 4H, NC**H**$_2$), 1.50 (s, 6H, C**H**$_3$), 1.22 (t, *J* = 7.0 Hz, 6H, C**H**$_3$) ppm. (**Figure S62**) **^{13}C NMR** (100 MHz, $CDCl_3$) δ 167.1, 156.1, 153.4, 148.2, 142.5, 140.8, 133.6, 132.3, 130.2, 128.5, 126.8, 126.6, 126.2, 125.6, 121.3, 120.4, 118.7, 110.9, 105.8, 52.1, 46.7, 44.8, 27.7, 12.8 ppm. (**Figure S63**) **HRMS** (EI+) m/z Calcd. For $C_{29}H_{31}NO_2$ $[M]^+$: 425.2355, Found: 425.2355. (**Figure S64**) **FT-IR** (**Figure S65**) **m.p.**: 161.9-162.4°C.

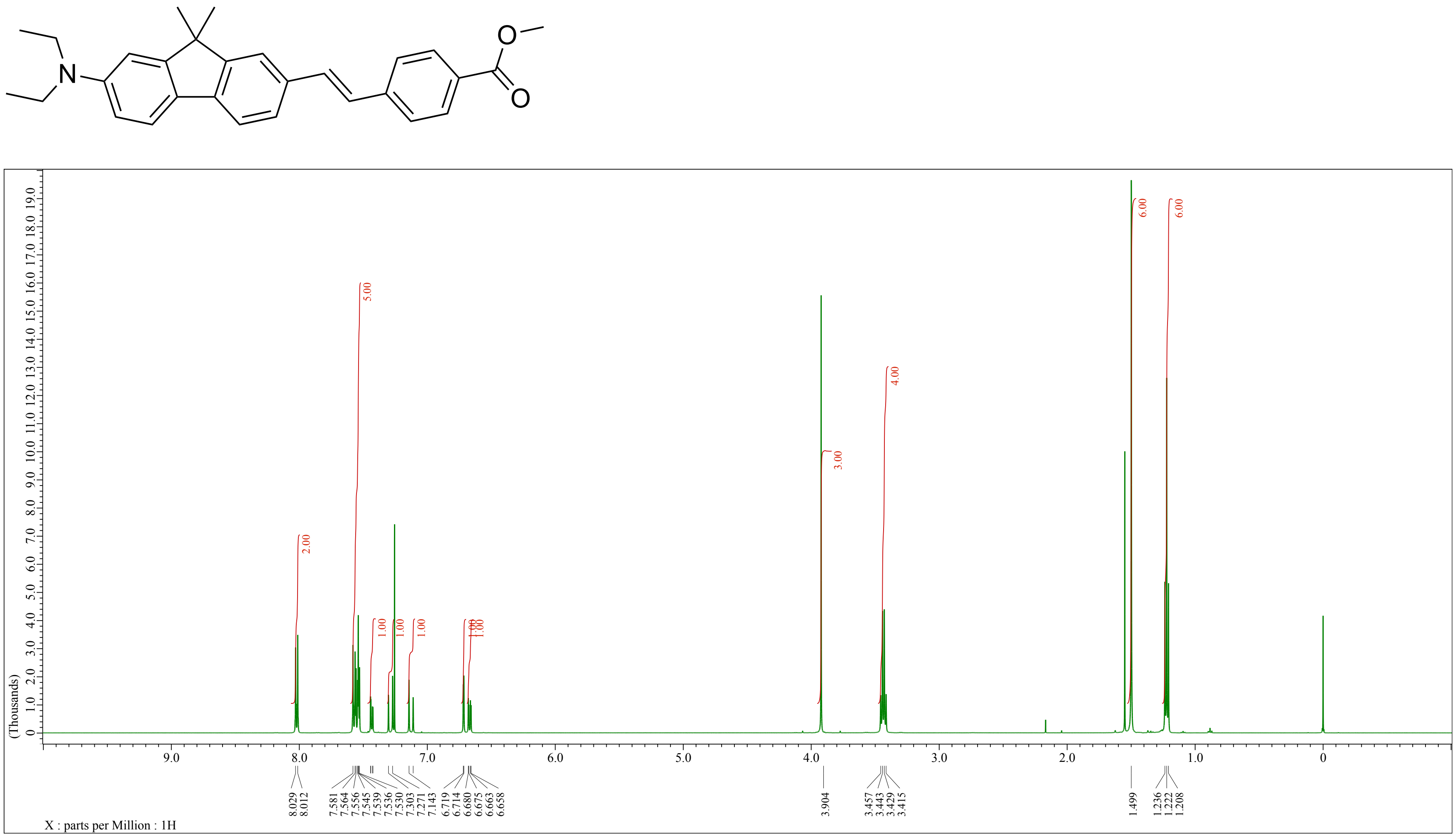

Figure S62. ^{1}H NMR (500 MHz, $CDCl_3$) spectrum of **FstCM**.

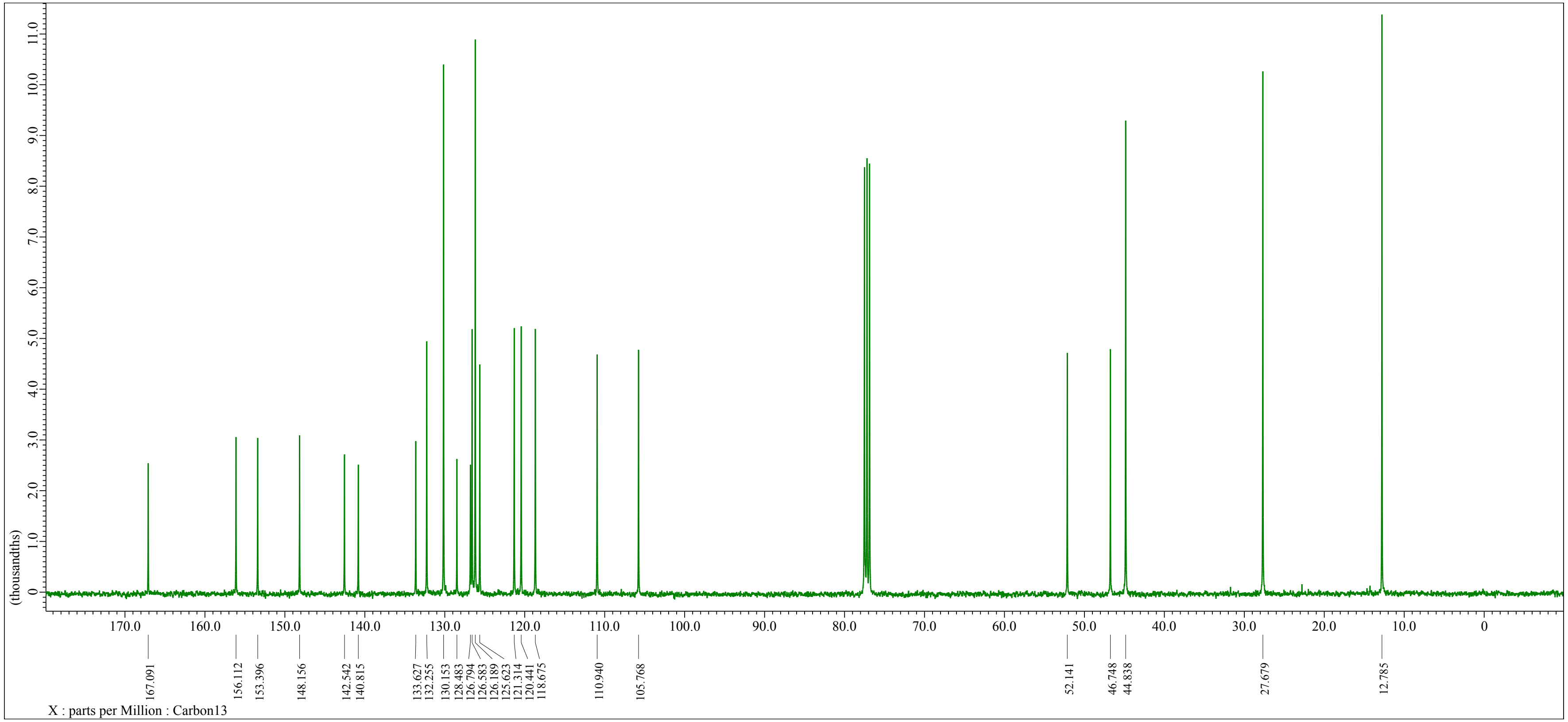

Figure S63. ^{13}C NMR (100 MHz, $CDCl_3$) spectrum of **FstCM**.

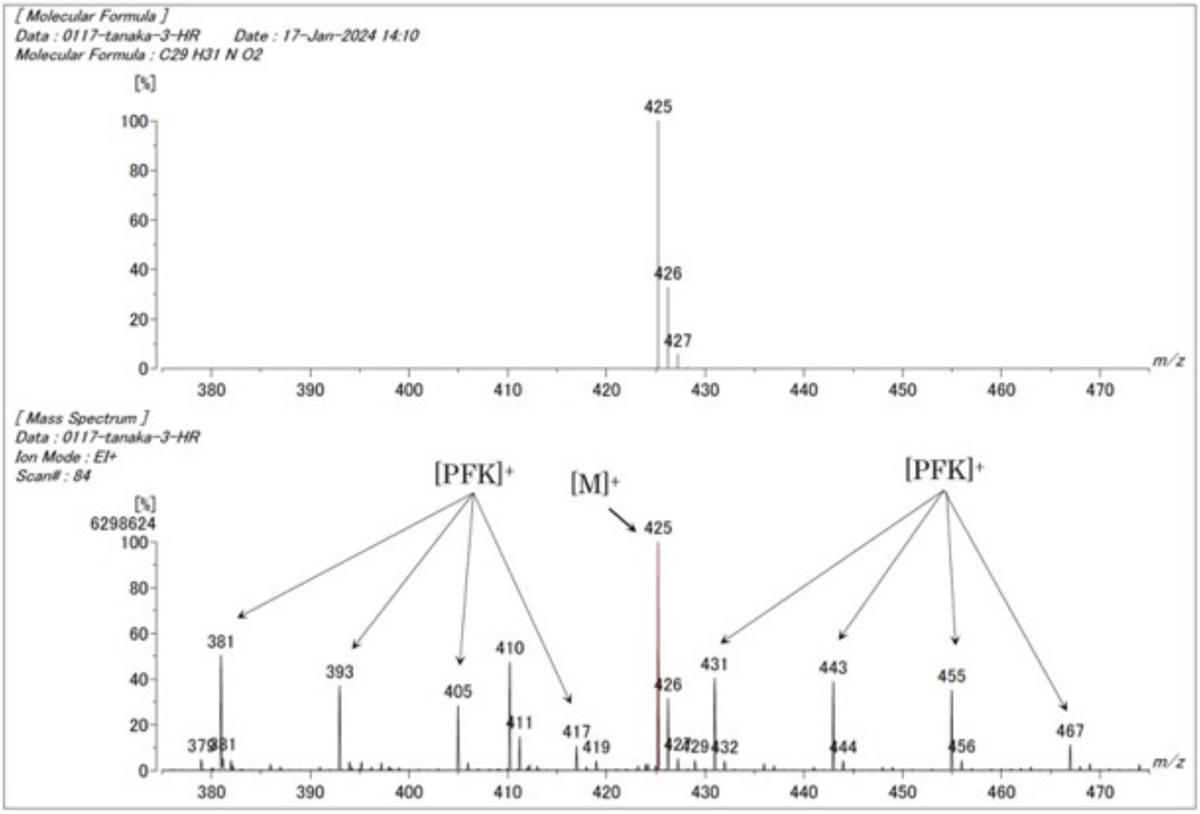

Observed m/z	Int%	Err[ppm / mmu]	U.S.	Composition
1 425.2361	100.00	+4.6 / +2.0	15.5	C27 H29 N4 O
2		+1.5 / +0.6	15.0	C29 H31 N O2
3		-2.9 / -1.2	7.5	C16 H29 N10 O4
4		+3.4 / +1.5	3.0	C13 H31 N9 O7
5		+0.3 / +0.1	2.5	C15 H33 N6 O8
6		-2.9 / -1.2	2.0	C17 H35 N3 O9

Figure S64. MS spectrum of **FstCM**.

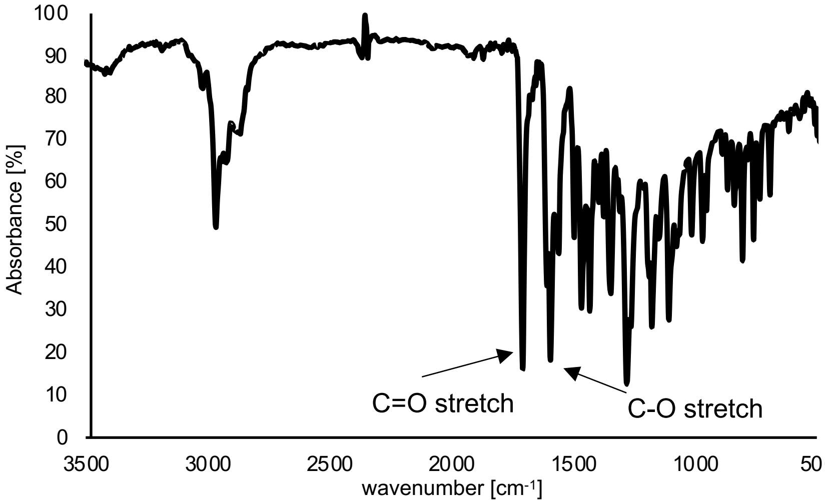

Figure S65. FT-IR spectrum of **FstCM**.

S3. Optical properties

S3-1. Photophysical properties

methyl 4-(7-(diethylamino)-9,9-dimethyl-*9H*-fluoren-2-yl) benzoate (FπCM).

Table S1. Optical properties of **FπCM**. Maximum absorption wavelength (λ_{abs}), maximum fluorescence wavelength (λ_{fl}), fluorescence quantum yields (λ_{fl}), fluorescence lifetime (τ), radiative deactivation rate constant (k_r) and non-radiative deactivation rate constant (k_{nr}).

solvent	$E_T(30)^b$	ε [M^{-1} cm^{-1}]	λ_{abs} [nm]	λ_{fl} [nm]	Φ_{fl} [-]	τ/χ^{2e} [ns]/[-]	k_r [10^8 s^{-1}]	k_{nr} [10^8 s^{-1}]
n-hexane	31.0	69600	364	431	0.88	1.6/1.04	5.5	0.75
toluene	33.9	61400	372	450	0.83	1.6/1.10	5.2	1.1
THF	37.4	63200	373	504	0.87	2.3/1.13	3.8	0.57
dichloromethane	40.7	61600	375	531	0.85	2.8/1.11	3.0	0.54
acetonitrile	45.6	63600	371	564	0.81	2.7/1.11	3.0	0.70
solid	-	-	-	496^c	0.48^c	-	-	-
		-	-	496^d	0.43^d	-	-	-

a. Difinitions. λ_{abs} = maximum absorption wavelength, λ_{fl} = maximum fluorescence wavelength, λ_{fl} = absolute fluorescence quantum yield, τ = fluorescence lifetime, k_r = radiative deactivation rate constant, k_{nr} = non-radiative deactivation rate constant, THF = tetrahydrofuran b. These data were obtained from ref. S4. c. amorphous solid. d. crystal solid e. CHI SQUARE.

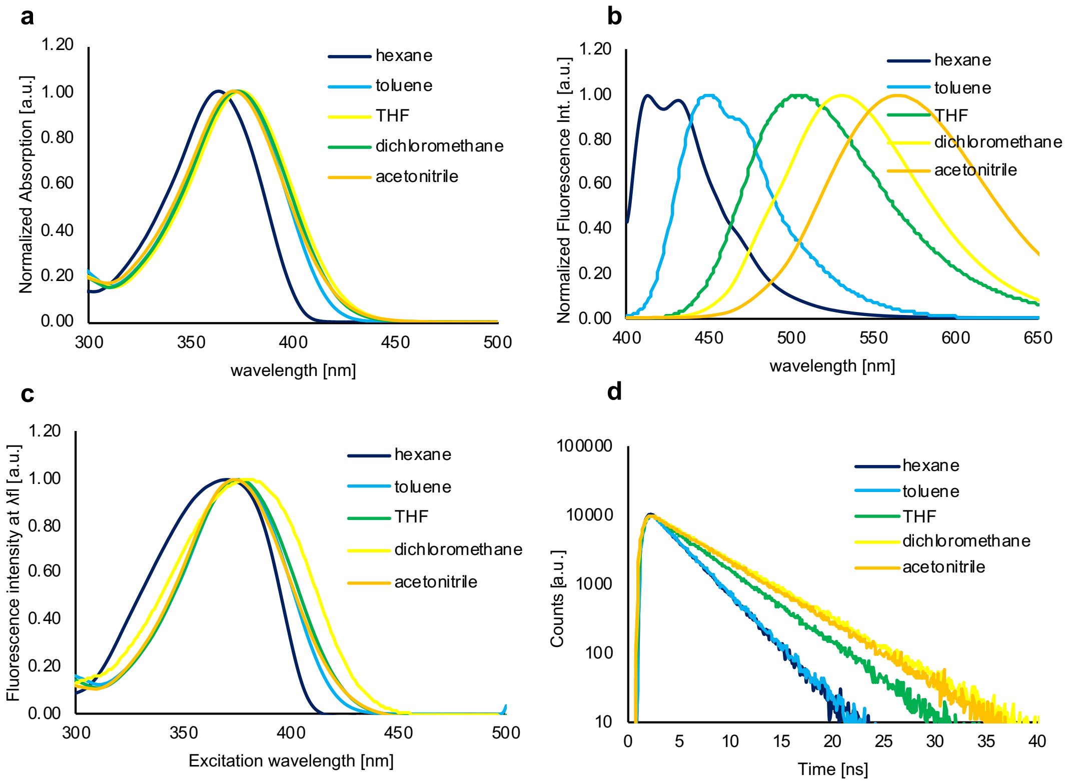

Figure S66. Optical measurement spectra of **FπCM**. **a**, absorption spectra. **b**, fluorescence spectra. The excitation wavelength was maximum absorption wavelength in each solvent. **c**, excitation spectra at maximum fluorescence wavelength (λ_{fl}). **d**, fluorescence lifetime decay profile (λ_{ex} = 379 nm).

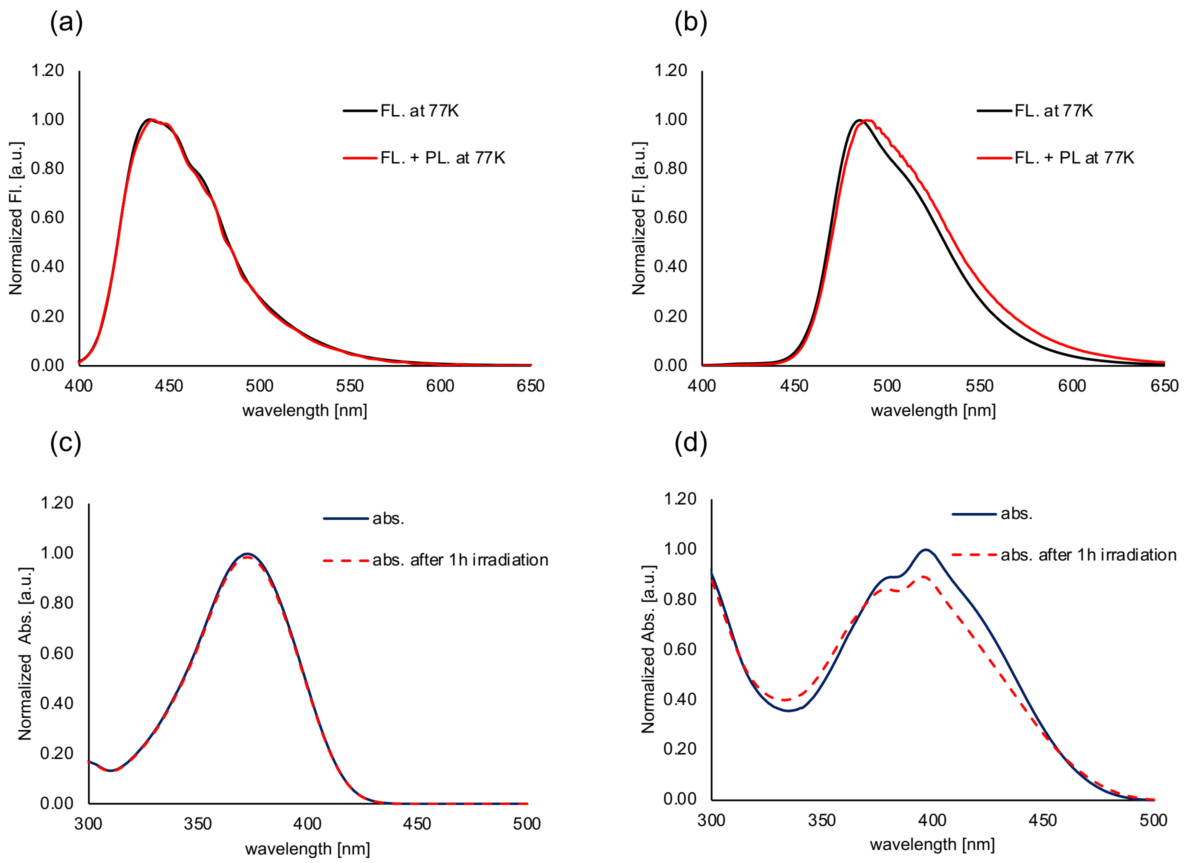

Figure S67. The fluorescence and phosphorescence measurement in EPA solvent (diethyl ether/isopentane/ethanol volume fraction 5:5:2): **a**, for FπCM at 77K. **b**, for PK at 77K. The absorption spectra in toluene of fresh samples and samples irradiated with a merry-go-round-type photoirradiation apparatus (Iris-MG, CELL System Co.) equipped with a 405 nm-centered LED light source with 32 mW output power for 1 h. output power for 1 h: **c**, FπCM. **d**, PK.

methyl 4-(9,9-dimethyl-7-(piperidin-1-yl)-*9H*-fluoren-2-yl)benzoate (FπpCM).

When the diethylamino group was changed to a piperidyl group (**FπpCM**), it showed λ_{abs} around 350 nm, which is about 20 nm blue-shifted compared to **FπCM**. Burger et al. reported that changing the electron-donating group in a push-pull type fluorene dye from a dialkylamino group to a cyclic amino group causes and that as the alkyl chain of the dialkylamino group is lengthened, the electron-donating property increases and the wavelength becomes longer in accordance with the increase in dielectric effect.[S5] This behavior is also applicable to π-conjugated extended fluorene, where the absorption maxima can be tuned by the structure of the amino group.

The solid-state fluorescence quantum yield λ_{fl} was about 20% lower than that of **FπpCM**; in the case of **FπpCM**, the piperidyl group is more planar than the diethylamino group, which

is likely to consume more energy through intermolecular electronic interactions, resulting in lower luminescence.

Table S2. Optical properties of **FπpCM**.

solvent	$E_T(30)^b$	ε [M^{-1} cm^{-1}]	λ_{abs} [nm]	λ_{fl} [nm]	Φ_{fl} [-]
n-hexane	31.0	52900	349	430	0.87
toluene	33.9	47400	357	458	0.88
THF	37.4	49800	356	515	0.91
dichloromethane	40.7	47600	354	547	0.91
acetonitrile	45.6	49200	354	570	0.84
solid	-	-	-	495^c	0.23^c

a. Difinitions. λ_{abs} = maximum absorption wavelength, λ_{fl} = maximum fluorescence wavelength, λ_{fl} = absolute fluorescence quantum yield, THF = tetrahydrofuran b. These data were obtained from ref. S4. c. amorphous solid.

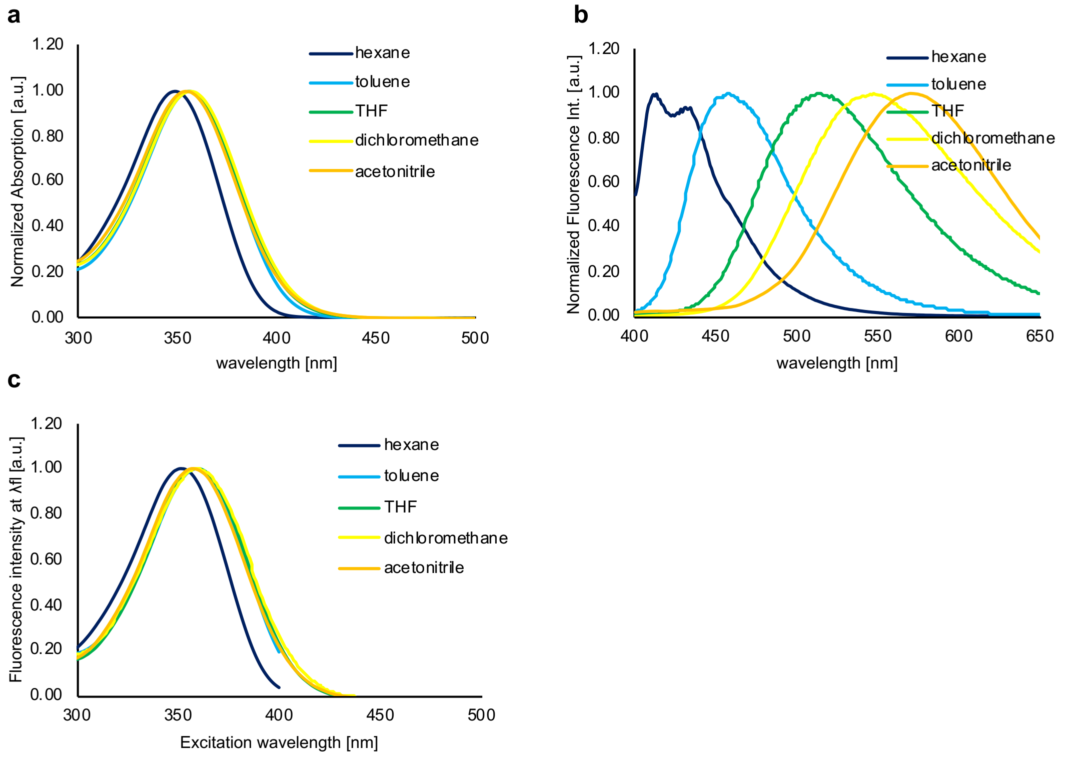

Figure S68. Optical measurement spectra of **FπpCM**. **a**, absorption spectra. **b**, fluorescence spectra. The excitation wavelength was maximum absorption wavelength in each solvent. **c**, excitation spectra at maximum fluorescence wavelength (λ_{fl}).

methyl 4-(7-(diethylamino)-9,9-dimethyl-9H-fluoren-2-yl)-2-fluorobenzoate (FπCM*o*-F)

FπCM*o*-F is a derivative of **FπCM**, the main compound in this paper, in which an electron-withdrawing fluorine is introduced at the ortho position of the methoxycarbonyl group. Fluorine has been reported as a substituent that increases electron-withdrawing ability. The λ_{abs}s of **FπCM*o*-F** were blue-shifted by 4~6 nm longer wavelength than that of **FπCM**, confirming that fluorine is more electron-withdrawing than hydrogen in the electronic ground state. In comparison with the absorption spectrum, the fluorescence spectrum corresponding to the electronically excited state shows almost the same λ_{fl} as that of **FπCM** for *n*-hexane and toluene, which are solvents with low polarity (dielectric constant), and a shift of about 20 nm longer than that of **FπCM** for medium polarity solvents such as THF and dichloromethane, and a shift of about 40 nm longer for high polarity solvents such as acetonitrile. This result suggests that the fluorine substituent undergoes stepwise stabilization as the polarity of the solvent increases, resulting in a longer wavelength shift of λ_{fl}.

Table S3. Optical properties of **FπCM*o*-F**.

solvent	$E_T(30)^b$	ε [M^{-1} cm^{-1}]	λ_{abs} [nm]	λ_{fl} [nm]	Φ_{fl} [-]	τ/χ^{2e} [ns]/[-]	k_r [10^8 s^{-1}]	k_{nr} [10^8 s^{-1}]
n-hexane	31.0	65500	368	438	0.90	1.6/1.25	5.6	0.63
toluene	33.9	57100	378	463	0.89	1.8/1.11	4.9	0.60
THF	37.4	69400	379	524	0.94	2.4/1.04	3.9	0.25
dichloromethane	40.7	58100	379	552	0.84	2.9/1.04	2.9	0.55
acetonitrile	45.6	59700	376	604	0.77	3.3/1.10	2.3	0.70
solid	-	-	-	505^c	0.54^c	-	-	-
		-	-	507^d	0.23^d	-	-	-

a. Difinitions. λ_{abs} = maximum absorption wavelength, λ_{fl} = maximum fluorescence wavelength, λ_{fl} = absolute fluorescence quantum yield, τ = fluorescence lifetime, k_r = radiative deactivation rate constant, k_{nr} = non-radiative deactivation rate constant, THF = tetrahydrofuran b. These data were obtained from ref. S4. c. amorphous solid. d. crystal solid. e. CHI SQUARE.

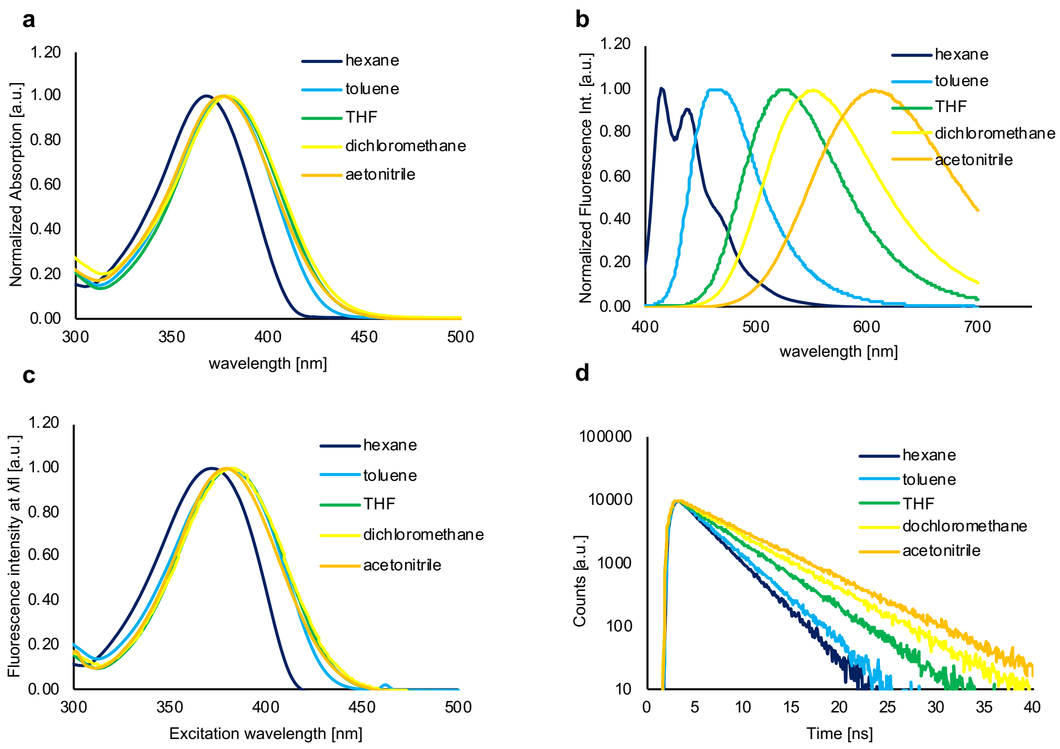

Figure S69. Optical measurement spectra of **FπCM*o*-F**. **a**, absorption spectra. **b**, fluorescence spectra. The excitation wavelength was maximum absorption wavelength in each solvent. **c**, excitation spectra at maximum fluorescence wavelength (λ_{fl}). **d**, fluorescence lifetime decay profile (λ_{ex} = 379 nm).

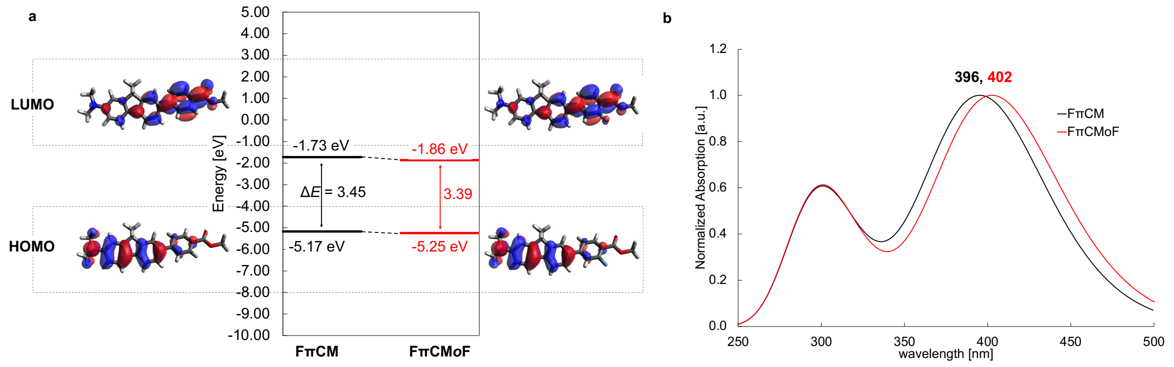

Figure S70. **a**, Optimized structure and HOMO-LUMO energies in the ground states of FπCM and FπCMo-F calculated by a DFT method. **b**, Absorption spectra of FπCM and FπCMo-F calculated from single-point energy calculations. All calculations were performed at the B3LYP/6-311G+(d,p) level of theory.

1-(4-(7-(diethylamino)-9,9-dimethyl-*9H*-fluoren-2-yl)phenyl)ethan-1-one (FπA).

We have investigated the photophysical properties of **FπA** an acetyl derivative of **FπCM**. The λ_{abs} of **FπA** was slightly longer wavelength shifted than that of **FπCM**, suggesting that the electron-withdrawing property of the substituent has little effect on the λ_{abs}. Compared to the reference compounds **FR0** and **FR8**[S1].the λ_{abs} was shifted about 25 nm shorter. In the ground-state stable structure obtained from single crystal X-ray structure analysis and structure-optimized from quantum chemical calculations, a dihedral angle of about 35° was formed between the phenyl group and the fluorene backbone. It is considered that the effective absorption wavelength is reduced at least sterically.

FπA showed the same solvatochromic properties as **FπCM**. Its λ_{fl} recorded a longer wavelength than that of **FπCM**, confirming that the acetyl group has greater electron-withdrawing properties than the ester group in the excited state. The fluorescence quantum yields (λ_{fl}) also showed fluorescence properties characteristic of compounds, which also have large transition dipole moments ($\Delta\mu$) (**Table S4**). Dye with a large $\Delta\mu$ generally inactivates fluorescence in highly polar solvents due to the energy gap law[S6], and **FπA** also exhibited this behavior, with λ_{fl} = 0.10 in acetonitrile. This result is not expected for **FπA** as a membrane probe, since it cannot stably emit in all polarity ranges.

Table S4. Optical properties of **FπA**.

solvent	$E_T(30)^b$	ε [M^{-1} cm^{-1}]	λ_{abs} [nm]	λ_{fl} [nm]	Φ_{fl} [-]	τ/χ^{2e} [ns]/[-]	k_r [10^8 s^{-1}]	k_{nr} [10^8 s^{-1}]
n-hexane	31.0	37200	369	440	0.85	1.4/1.05	6.1	1.1
toluene	33.9	41500	378	477	0.93	1.8/1.11	5.3	0.40
THF	37.4	34200	377	536	0.98	2.7/1.18	3.6	0.070
dichloromethane	40.7	37500	382	578	0.42	1.6/1.16	2.6	3.6
acetonitrile	45.6	41800	376	661	0.10	1.6/1.16	0.60	5.6
solid	-	-	-	492^c	0.03^c	-	-	-
		-	-	492^d	0.03^d	-	-	-

a. Difinitions. λ_{abs} = maximum absorption wavelength, λ_{fl} = maximum fluorescence wavelength, λ_{fl} = absolute fluorescence quantum yield, τ = fluorescence lifetime, k_r = radiative deactivation rate constant, k_{nr} = non-radiative deactivation rate constant, THF = tetrahydrofuran b. These data were obtained from ref. S4. c. amorphous solid. d. crystal solid. e. CHI SQUARE.

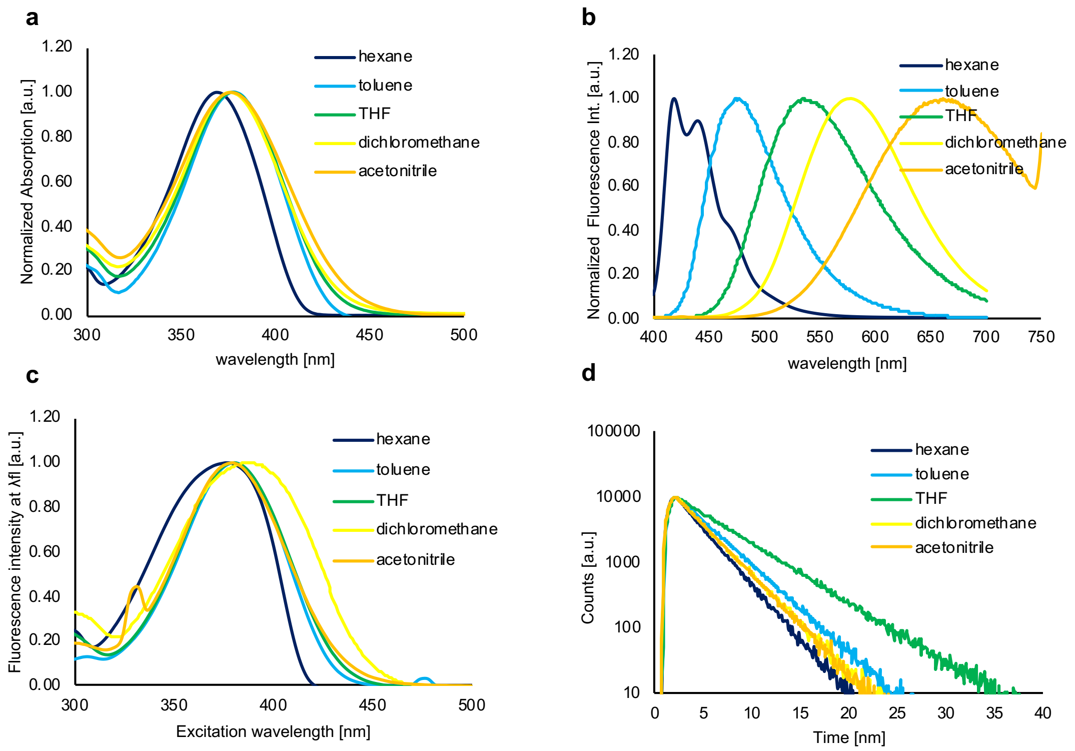

Figure S71. Optical measurement spectra of **FπA**. **a**, absorption spectra. **b**, fluorescence spectra. The excitation wavelength was maximum absorption wavelength in each solvent. **c**, excitation spectra at maximum fluorescence wavelength (λ_{fl}). **d**, fluorescence lifetime decay profile (λ_{ex} = 379 nm).

methyl 7-(diethylamino)-9,9-dimethyl-9H-fluorene-2-carboxylate (FCM).

We investigated **FCM** of the ester derivatives of the fluorene membrane probes **FR0** and **FR8**. The λ_{abs} of **FCM** were blue shifted by about 30 nm compared to those of **FR0** and **FR8**. Furthermore, the FWHM absorption spectrum of **FCM** is relatively smaller than that of **FπCM**, etc., and absorbs almost no light at the wavelength of common laser light (405 nm). However, λ_{fl} = 469 nm for the highly polar acetonitrile solvent was shorter wavelength than **FR0** (518 nm) and **FR8** (498 nm), covering only the blue to light blue range. On the other hand, formyl and acetyl groups hardly emit light in *n*-hexane with a λ_{fl} of about 10%, while **FCM** recorded a λ_{fl} of 92%. This result suggests that the ester group is more luminescent than other carbonyl substituents and that there is little intersystem crossing over to the excited triplet state in low-polarity solvents.

Table S5. Optical properties of **FCM**.

solvent	$E_T(30)^b$	ε [M^{-1} cm^{-1}]	λ_{abs} [nm]	λ_{fl} [nm]	Φ_{fl} [-]	τ/χ^{2d} [ns]/[-]	k_r [10^8 s^{-1}]	k_{nr} [10^8 s^{-1}]
n-hexane	31.0	47600	368	379	0.92	1.6//1.01	5.8	0.50
toluene	33.9	39500	373	407	0.98	1.6/1.03	6.1	0.10
THF	37.4	37900	371	429	0.93	1.9/0.89	4.9	0.37
dichloromethane	40.7	37900	370	452	0.84	2.0/1.07	4.2	0.80
acetonitrile	45.6	37800	370	469	0.96	2.5/1.00	3.8	0.16
solid	-	- -	- -	456^c	0.12^c	- -	- -	- -

a. Difinitions. λ_{abs} = maximum absorption wavelength, λ_{fl} = maximum fluorescence wavelength, λ_{fl} = absolute fluorescence quantum yield, τ = fluorescence lifetime, k_r = radiative deactivation rate constant, k_{nr} = non-radiative deactivation rate constant, THF = tetrahydrofuran b. These data were obtained from ref. S4. c. amorphous solid. d. CHI SQUARE.

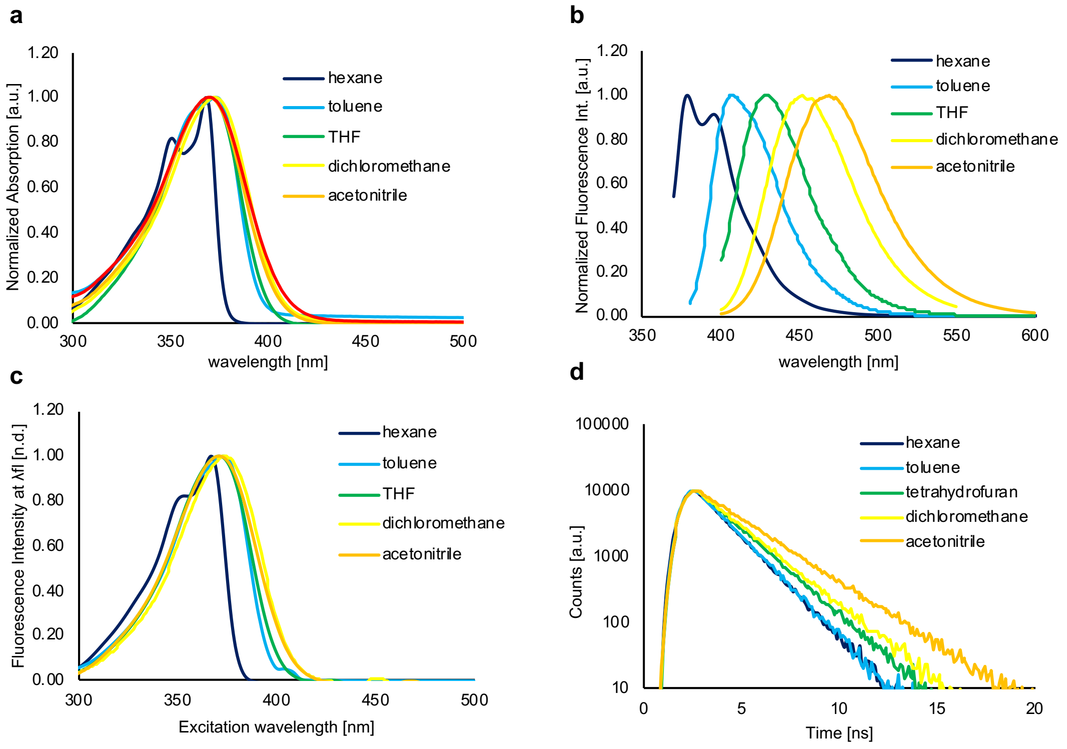

Figure S72. Optical measurement spectra of **FCM** in organic solvents. **a**, absorption spectra. **b**, fluorescence spectra. The excitation wavelength was maximum absorption wavelength in each solvent. **c**, excitation spectra at maximum fluorescence wavelength (λ_{fl}). **d**, fluorescence lifetime decay profile (λ_{ex} = 379 nm).

methyl 6-(piperidin-1-yl)pyrene-1-carboxylate (PCM).

The photophysical properties of the ester derivative **PCM** of the reference compound **PK** was that the λ_{abs}s of PCM were slightly blue shifted compared to that of **PK** (≈ 405 nm), but almost close to 400 nm, showing versatility with respect to common lasers (405 nm). For the fluorene skeleton, the electron-withdrawing property of the ester group was smaller than that of the acetyl group, resulting in a shorter wavelength shift of λ_{abs} by about 30 nm, while the pyrene skeleton showed almost no change. This suggests that the effective conjugation length is longer for the pyrene skeleton than for the fluorene skeleton, regardless of the strength of the electron-withdrawing group.

The luminescence properties of the **PCM** exhibited solvatochromic properties that shifted in long wavelengths according to the polarity of the solvent. A concern with esters with low electron-withdrawing properties was that they did not provide sufficient fluorescence wavelengths, making them unsuitable for ratiometric detection. However, it showed a sufficient wavelength shift that was almost comparable to the λ_{fl} of **PK**. Its λ_{fl} was high regardless of the polarity of the solvent, indicating its potential as a tool for visualizing cell membranes.

Table S6. Optical properties of **PCM**.

solvent	$E_T(30)^b$	ε [M^{-1} cm^{-1}]	λ_{abs} [nm]	λ_{fl} [nm]	Φ_{fl} [-]	τ/χ^{2e} [ns]/[-]	k_r [10^8 s^{-1}]	k_{nr} [10^8 s^{-1}]
n-hexane	31.0	19500	392	456	0.89	3.4/1.06	2.6	0.32
toluene	33.9	19500	391	482	0.96	3.4/1.00	2.8	0.10
THF	37.4	19100	394	503	0.89	4.0/1.06	2.2	0.27
dichloromethane	40.7	18100	398	512	0.92	4.4/1.04	2.1	0.18
acetonitrile	45.6	18200	394	536	0.77	4.4/1.07	1.8	0.52
solid	-	-	-	522^c	0.09^c	-	-	-
		-	-	533^d	0.12^d	-	-	-

a. Difinitions. λ_{abs} = maximum absorption wavelength, λ_{fl} = maximum fluorescence wavelength, λ_{fl} = absolute fluorescence quantum yield, τ = fluorescence lifetime, k_r = radiative deactivation rate constant, k_{nr} = non-radiative deactivation rate constant, THF = tetrahydrofuran b. These data were obtained from ref. S4. c. amorphous solid. d. crystal solid. e. CHI SQUARE.

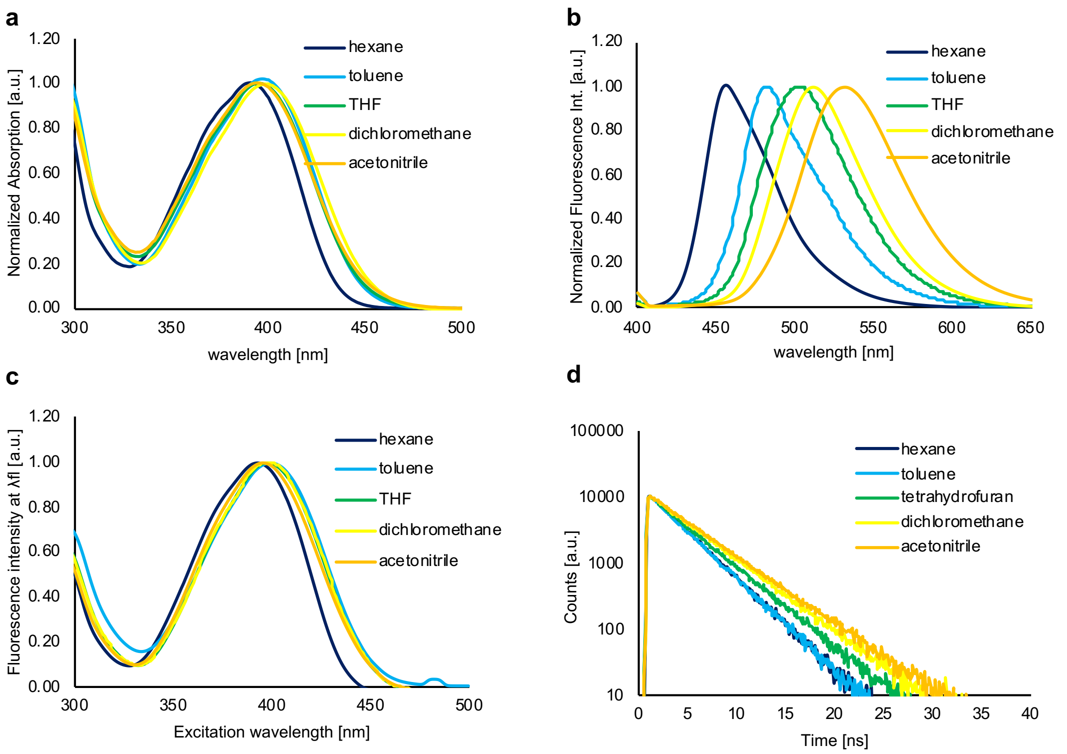

Figure S73. Optical measurement spectra of **PCM** in organic solvents. **a**, absorption spectra. **b**, fluorescence spectra. The excitation wavelength was maximum absorption wavelength in each solvent. **c**, excitation spectra at maximum fluorescence wavelength (λ_{fl}). **d**, fluorescence lifetime decay profile (λ_{ex} = 379 nm).

methyl (*E*)-4-(2-(7-(diethylamino)-9,9-dimethyl-9*H*-fluoren-2-yl)vinyl)-2-fluorobenzoate (FstCM*o*-F).

FstCMo-F exhibits λ_{fl} = 671 nm in highly polar solvent acetonitrile, and it measured a significant Stokes shift with 262 nm (9547 cm^{-1}) in acetonitrile. The fluorescence band extends to around 800 nm, achieving near-infrared emission. Generally, the non-radiative deactivation rate constant (k_{nr}) of organic fluorescent dyes follows the energy gap law, as indicated by the equation S1. It is known that k_{nr} follows the energy gap rule, as shown in the equation.[S5] The limit of induced transition dipole moments in organic single molecules becomes ineffective as ΔE decreases into the near-infrared and infrared regions. As a result, developing dyes that emit in the near-infrared and infrared regions is considered challenging. **FstCMo-F** in acetonitrile

has a higher k_{nr} value than in less polar solvents but records a relatively high Φ_{fl} (0.45) due to the concerted result of similar k_{r} values. k_{nr} can be expressed by the equation S2, where A represents Einstein's A coefficient. A is proportional to ΔE and the transition dipole moment M. The dipole moment of **FstCMo-F** is oriented along the molecular long axis, and furthermore, the $\Delta\mu$ calculated from the Lippert-Mataga plot is 19 D, larger than any dye calculated in **Table S13**. As a result of the large $\Delta\mu$ (M), k_{r} increases, allowing it to bypass the energy gap law and emit strongly. The molecular design with a styryl group using a fluorene skeleton for conjugation extension may be applicable to the development of future near-infrared emitting dyes.

$$k_{nr} \propto \alpha \exp(-\beta \Delta E) \ \text{(eq. S1)} \qquad k_r \propto A = \frac{(\Delta E)^3}{3\pi\varepsilon_0 c^3 \hbar^4}|M|^2 \ \text{(eq. S2)}$$

Table S7. Optical properties of **FstCM*o*-F**.

solvent	$E_{\text{T}}(30)$[b]	ε [M^{-1} cm^{-1}]	λ_{abs} [nm]	λ_{fl} [nm]	Φ_{fl} [-]	τ/χ^{2}[d] [ns]/[-]	k_{r} [10^8 s^{-1}]	k_{nr} [10^8 s^{-1}]
n-hexane	31.0	42300	405	450, 476	0.89	1.6/1.02	5.6	0.69
toluene	33.9	43800	412	500	0.89	1.8/1.07	4.9	0.60
THF	37.4	39300	412	571	0.85	2.8/1.09	3.0	0.54
dichloromethane	40.7	37500	417	600	0.83	2.9/1.10	2.9	0.59
acetonitrile	45.6	38300	409	671	0.45	2.2/1.06	2.0	2.5
solid	-	-<br>-	-<br>-	548[c]	0.18[c]	-<br>-	-<br>-	-<br>-

a. Difinitions. λ_{abs} = maximum absorption wavelength, λ_{fl} = maximum fluorescence wavelength, λ_{fl} = absolute fluorescence quantum yield, τ = fluorescence lifetime, k_{r} = radiative deactivation rate constant, k_{nr} = non-radiative deactivation rate constant, THF = tetrahydrofuran b. These data were obtained from ref. S4. c. amorphous solid. d. CHI SQUARE

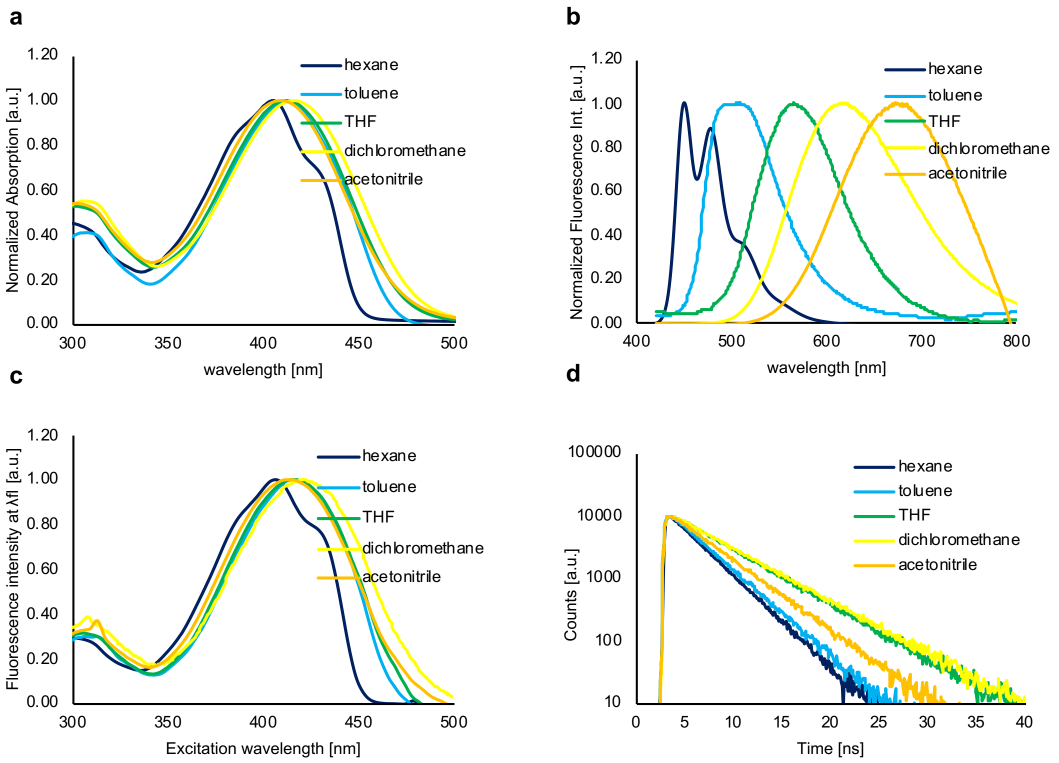

Figure S74. Optical measurement spectra of **FstCM*o*-F** in organic solvents. **a**, absorption spectra. **b**, fluorescence spectra. The excitation wavelength was maximum absorption wavelength in each solvent. **c**, excitation spectra at maximum fluorescence wavelength (λ_{fl}). **d**, fluorescence lifetime decay profile (λ_{ex} = 401 nm)

methyl (*E*)-3-(4-(7-(diethylamino)-9,9-dimethyl-9*H*-fluoren-2-yl)phenyl)acrylate (FπCMst)

The λ_{abs}s of FπCMst is about 10 nm longer than those of FπCM. As shown in **Table S8**, λ_{fl} shows a longer wavelength shift than those of FπCM. However, in a highly polar solvent, acetonitrile, λ_{fl} = 441 nm, charge separation associated with intramolecular charge transfer occurred, and only partial emission (Φ_{fl} = 0.03) was observed.

Table S8. Optical properties of **FπCMst**.

solvent	$E_T(30)$[b]	ε [M^{-1} cm^{-1}]	λ_{abs} [nm]	λ_{fl} [nm]	Φ_{fl} [-]	τ/χ^{2d} [ns]/[-]	k_r [10^8 s^{-1}]	k_{nr} [10^8 s^{-1}]
n-hexane	31.0	52000	376	432, 456	0.79	1.3/1.02	6.1	1.6
toluene	33.9	45100	385	481	0.99	1.8/1.01	5.5	0.10
THF	37.4	47100	383	568	0.99	2.7/1.02	3.7	0.040
dichloromethane	40.7	45700	386	608	0.24	0.88/1.04	2.7	8.6
acetonitrile	45.6	49200	380	441	0.03	1.7/1.09	0.20	5.7
solid	-	-	-	500[c]	0.71[c]	-	-	-

a. Difinitions. λ_{abs} = maximum absorption wavelength, λ_{fl} = maximum fluorescence wavelength, λ_{fl} = absolute fluorescence quantum yield, τ = fluorescence lifetime, k_r = radiative deactivation rate constant, k_{nr} = non-radiative deactivation rate constant, THF = tetrahydrofuran b. These data were obtained from ref. S4. c. amorphous solid. d. CHI SQUARE

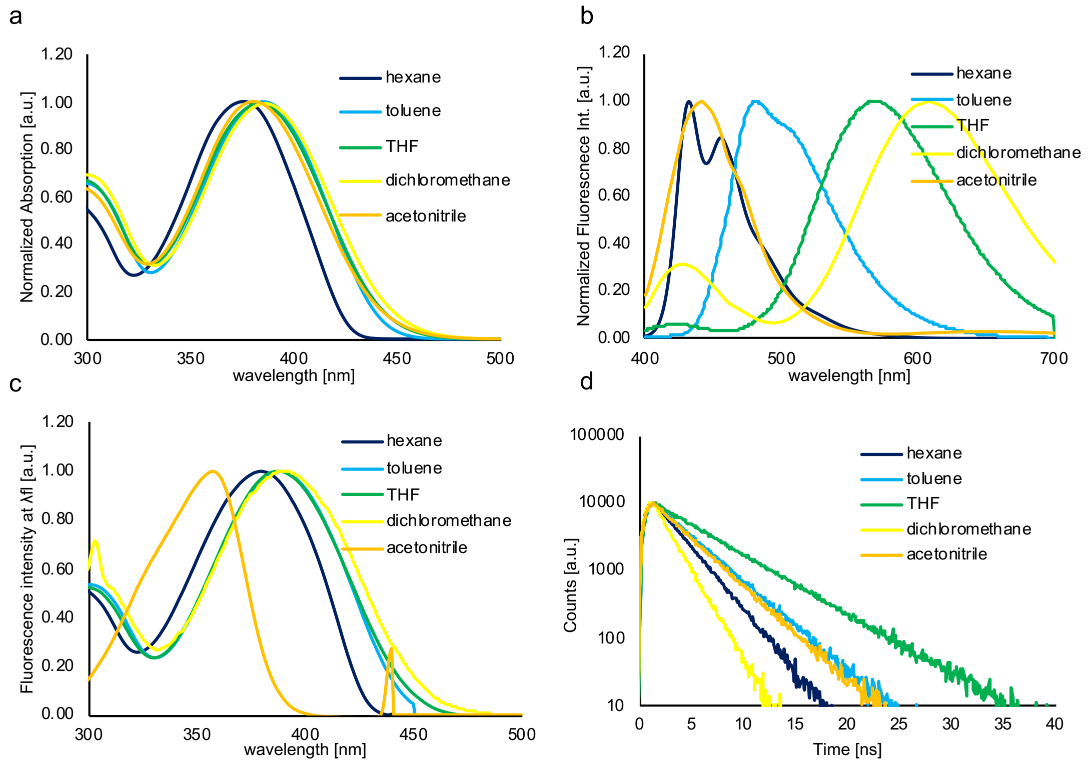

Figure S75. Optical measurement spectra of **FπCMst** in organic solvents. **a**, absorption spectra. **b**, fluorescence spectra. The excitation wavelength was maximum absorption wavelength in each solvent. **c**, excitation spectra at maximum fluorescence wavelength (λ_{fl}). **d**, fluorescence lifetime decay profile (λ_{ex} = 379 nm)

methyl (*E*)-3-(6-(piperidin-1-yl)pyren-1-yl)acrylate (PCMst)

The λ_{abs} of PCMst were longer wavelengths than those of PCM as well as FπCMst. Furthermore, both λ_{fl} and Φ_{fl} showed better values than those of PCM, suggesting that they have sufficient photophysical properties as imaging dyes. However, considering that even PCM takes longer to penetrate cell membranes due to its high hydrophobicity, it can be predicted that PCMst will be more difficult.

Table S9. Optical properties of **PCMst**.

solvent	$E_T(30)^b$	ε [M^{-1} cm^{-1}]	λ_{abs} [nm]	λ_{fl} [nm]	Φ_{fl} [-]	τ/χ^{2d} [ns]/[-]	k_r [10^8 s^{-1}]	k_{nr} [10^8 s^{-1}]
n-hexane	31.0	33200	400	480	0.75	2.7/1.03	2.8	0.93
toluene	33.9	30300	417	525	0.98	3.3/0.99	3.0	0.10
THF	37.4	31100	415	553	0.97	4.0/1.04	2.4	0.070
dichloromethane	40.7	29700	420	577	0.95	4.4/1.10	2.2	0.11
acetonitrile	45.6	30500	414	595	0.95	4.6/1.08	2.1	0.11
solid	-	-	-	522^c	0.72^c	-	-	-

a. Difinitions. λ_{abs} = maximum absorption wavelength, λ_{fl} = maximum fluorescence wavelength, λ_{fl} = absolute fluorescence quantum yield, τ = fluorescence lifetime, k_r = radiative deactivation rate constant, k_{nr} = non-radiative deactivation rate constant, THF = tetrahydrofuran b. These data were obtained from ref. S4. c. amorphous solid. d. CHI SQUARE

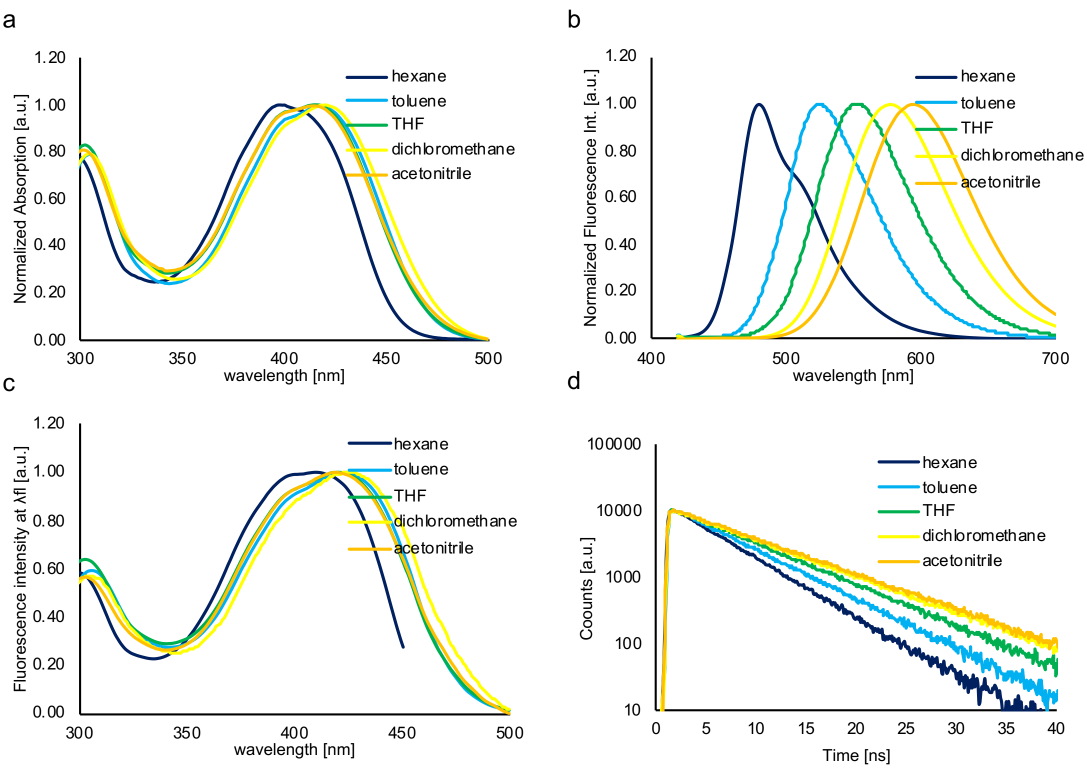

Figure S76. Optical measurement spectra of **PCMst** in organic solvents. **a**, absorption spectra. **b**, fluorescence spectra. The excitation wavelength was maximum absorption wavelength in each solvent. **c**, excitation spectra at maximum fluorescence wavelength (λ_{fl}). **d**, fluorescence lifetime decay profile (λ_{ex} = 401 nm)

methyl (*E*)-4-(2-(7-(diethylamino)-9,9-dimethyl-9*H*-fluoren-2-yl)vinyl)benzoate (FstCM)

Similar to FstCM*o*-F, FstCM has λ_{abs} around 405 nm, λ_{fl} at longer wavelengths than FπCM, and high Φ_{fl} regardless of the polar environment of the solvent, making it suitable for bioimaging.

Table S10. Optical properties of **FstCM**.

solvent	$E_T(30)^b$	ε [M^{-1} cm^{-1}]	λ_{abs} [nm]	λ_{fl} [nm]	Φ_{fl} [-]	τ/χ^{2d} [ns]/[-]	k_r [10^8 s^{-1}]	k_{nr} [10^8 s^{-1}]
n-hexane	31.0	67600	399	440, 469	0.72	1.3/1.05	5.5	2.2
toluene	33.9	57700	407	502	0.94	1.7/1.15	5.5	0.40
THF	37.4	59600	405	552	0.93	2.3/1.04	4.0	0.30
dichloromethane	40.7	58200	408	591	0.93	2.6/1.08	3.6	0.27
acetonitrile	45.6	60400	402	626	0.89	2.4/1.08	3.7	0.46
solid	-	-	-	525^c	0.70^c	-	-	-

a. Difinitions. λ_{abs} = maximum absorption wavelength, λ_{fl} = maximum fluorescence wavelength, λ_{fl} = absolute fluorescence quantum yield, τ = fluorescence lifetime, k_r = radiative deactivation rate constant, k_{nr} = non-radiative deactivation rate constant, THF = tetrahydrofuran b. These data were obtained from ref. S4. c. amorphous solid. d. CHI SQUARE

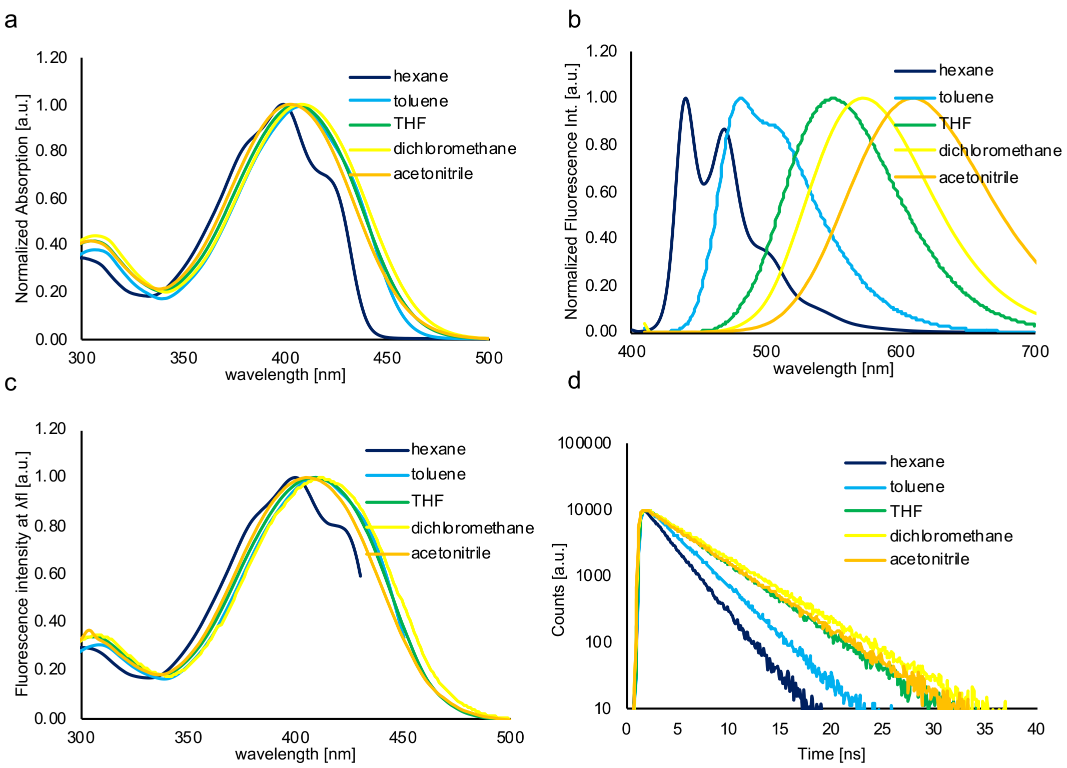

Figure S77. Optical measurement spectra of **FstCM** in organic solvents. **a**, absorption spectra. **b**, fluorescence spectra. The excitation wavelength was maximum absorption wavelength in each solvent. **c**, excitation spectra at maximum fluorescence wavelength (λ_{fl}). **d**, fluorescence lifetime decay profile (λ_{ex} = 401 nm)

S3-2. Photostability

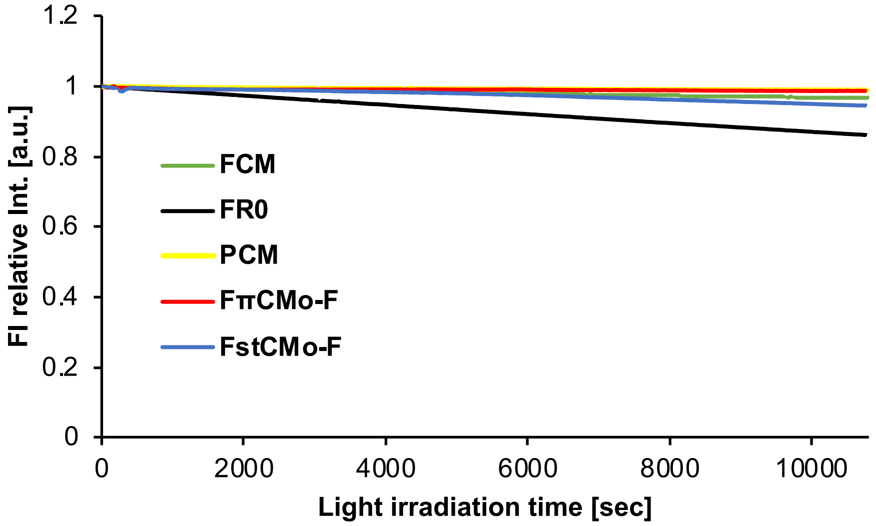

Figure S78. Photodegradation curves of **FCM**, **FR0**, **PCM, FπCM*o*-F** and **FstCMo-F** as a function of time in toluene (1.0×10^{-5} M) under the condition of oxygen.

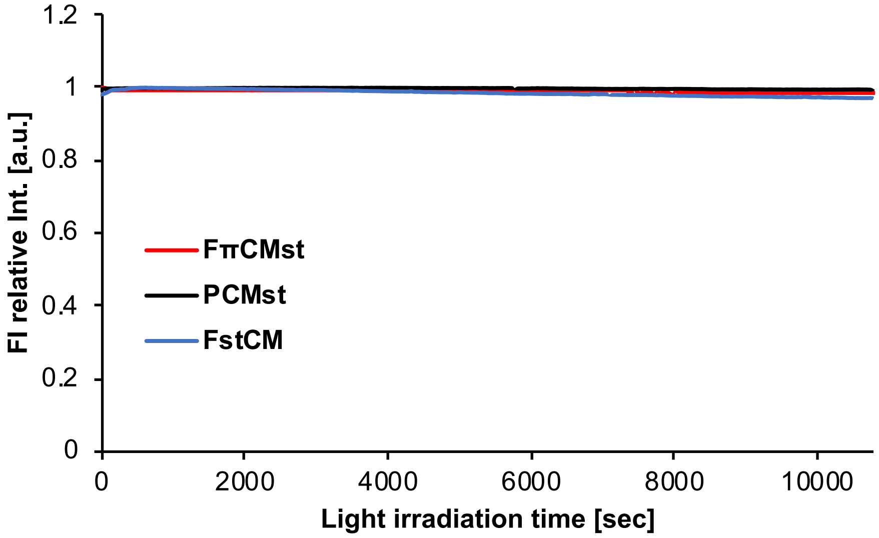

Figure S79. Photodegradation curves of **FπCMst**, **PCMst** and **FstCM** as a function of time in toluene (1.0×10^{-5} M) under the condition of oxygen.

S3-3. Concentration dependence

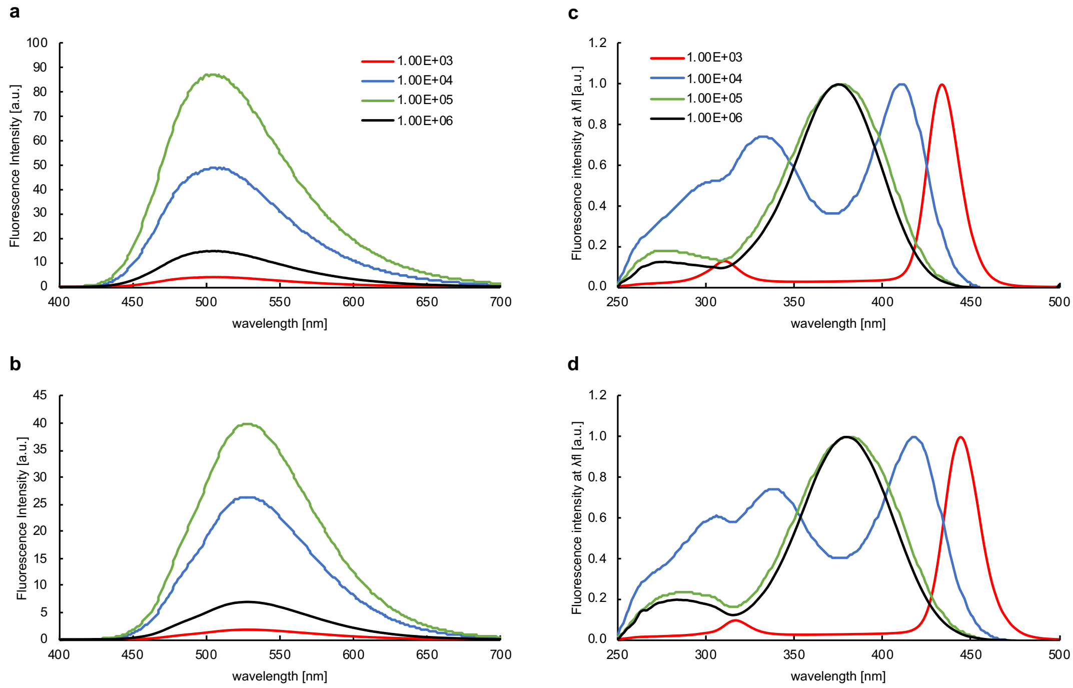

Figure S80. Concentration dependence of the fluorescence spectra **a**, for **FπCM** and **b**, for **FπA** and excitation spectra **c**, for **FπCM** and **d**, for **FπA**. the excitation spectra measured at maximum fluorescence wavelength (λ_{fl}) in each concentration in THF.

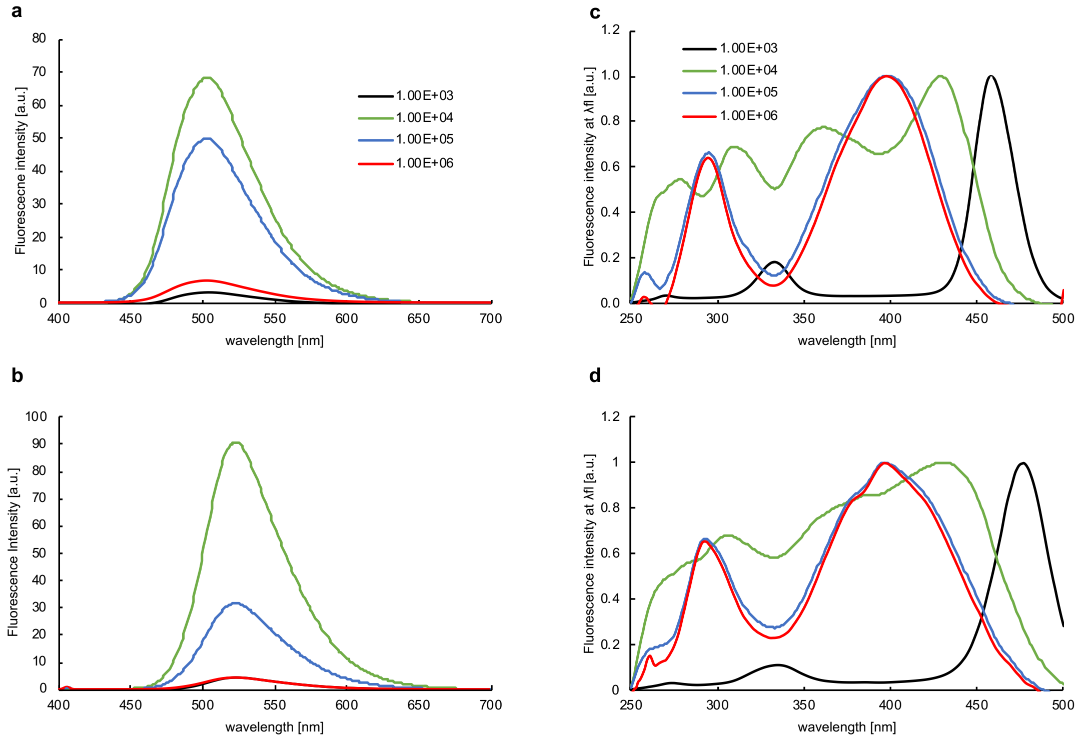

Figure S81. Concentration dependence of the fluorescence spectra **a**, for **PCM** and **b**, for **PK** and excitation spectra **c**, for **PCM** and **d**, for **PK**. the excitation spectra measured at maximum fluorescence wavelength (λ_{fl}) in each concentration in THF.

Table S11. Optical properties depending on the concentration in 10^{-6} to 10^{-3} M.

	**FπCM**				**FπA**			
	10^3 M	10^4 M	10^5 M	10^6 M	10^3 M	10^4 M	10^5 M	10^6 M
$\lambda_{fl,\ max}$ [nm][a]	504	504	504	504	528	528	528	528
Int. $_{fl}$ [-]	3.73/113[c]	49.1/111[c]	87.4	14.4	1.82/67.6[c]	26.4/64.1[c]	39.9	6.97
$\lambda_{ex,\ max}$ [nm]	434	411	380	380	444	418	380	380
Φ_{fl} [-]/λ_{ex} [nm][b]	0.873/373<br>0.870/434	0.876/373<br>0.881/411	0.873/373	0.85/373	0.947/377<br>0.937/444	0.959/377<br>0.951/418	0.975/377	0.992/377
	**PCM**				**PK**			
	10^3 M	10^4 M	10^5 M	10^6 M	10^3 M	10^4 M	10^5 M	10^6 M
$\lambda_{fl,\ max}$ [nm][a]	504	504	504	504	522	522	522	522
Int. $_{fl}$ [-]	3.41/111[c]	68.3/103[c]	49.8	7.03	4.14/120[c]	90.8/98.4[c]	31.6	4.32
$\lambda_{ex,\ max}$ [nm]	458	429	400	400	477	431	400	400
Φ_{fl} [-]/λ_{ex} [nm][b]	0.693/400<br>0.701/458	0.733/400<br>0.727/429	0.866/400	0.834/400	0.651/400<br>0.661/477	0.715/400<br>0.716/431	0.786/400	0.815/400

a. maximum fluorescence wavelength. b. fluorescence quantum yields were measured at maximum excitation wavelength (λ_{ex}). In the concentration of 10^{-3} and 10^{-4}, we detected two λ_{ex}. c. the data were obtained under the same conditions as λ_{fl} [-]/λ_{ex} [nm].

S3-4. Photophysical properties in solid-state and PMMA matrix

The **FπCM** derivatives emitted highly efficient luminescence in the solid and aggregated states. In general, highly planar compounds lose luminescence due to the energy consumed by intermolecular electronic interactions.[S7] Prodan and PK do not emit light in the solid state. (Φ_{fl} = 0.02) However, the solid-state Φ_{fl} of the ester compounds showed luminescence with **FCM**: 0.12, **FπCM**: 0.45, **FπpCM**: 0.24, **FπCMo-F**: 0.54, **FstCMo-F**: 0.18, **PCM**: 0.12. **FπCMst**: 0.71, **PCMst**: 0.72, **FstCM**: 0.70.

Here, we examined the luminescence mechanism of **FπCM**. Firstly, the fluorescence spectra of **FπCM** and **FπA** in the solid state showed an approximately 50 nm redshift compared to their spectra in dilute solution (here, toluene, which is considered to be close to the solid-state environment, was used). This redshift in the fluorescence wavelength is a typical characteristic of *J*-aggregates. Furthermore, comparing the concentration-dependent excitation spectra shown in **Figure S80**, it can be observed that as the concentration increases, the

maximum excitation wavelength shifts to longer wavelengths, and the spectra become sharper. Such results suggest the formation of *J*-aggregates.

Interestingly, **FπCM** and **FπA** exhibit significantly different Φ_{fl} values in the solid state. This is attributed to the fact that in the case of **FπA**, the energy levels of the *J*-aggregates approach the triplet state closely, resulting in a small energy gap and, as a consequence, facilitating intersystem crossing. In contrast, the properties of **FπCM**, which show a low propensity for intersystem crossing even in solution, are retained in the solid state, leading to relatively low non-radiative deactivation due to intersystem crossing and hence, exhibiting luminescence.

Comparing the single crystal structures of **FπCM** and the acetyl form of **FπA** (Φ_{fl} = 0.03), the packing manners are consistent. The intermolecular interaction energies, taking into account the Basis Set Superposition Error (BSSE),[S8] are also almost identical, suggesting that the electron-withdrawing groups are responsible for the fate of the solid-state luminescence. Even **FstCMo-F** and **PCM**, which form dimers in the electronic ground state with small intermolecular distances (**Figures S94** and **S92**), are relatively luminescent.

The λ_{fl} of **FπCM*o*-F** in the solid state, in contrast to that of **FπCM**, changed between the single-crystal state (0.23) and the amorphous state (0.54). λ_{fl} was almost identical, suggesting that the emission components are the same. Comparing the molecular arrangement of **FπCM*o*-F** (**Figure S93**) with that of **FπCM** (**Figure S90**), it was found that **FπCM** takes an arrangement stabilized by intermolecular CH-π interactions, whereas **FπCM*o*-F** takes an almost π-π interaction arrangement with molecules separated from each other. This result was agreed by the interaction energy between the two molecules (-89.1 kJ/mol) calculated by quantum chemical calculations. The decrease in Φ_{fl} in the single crystal state can be attributed to the formation of permanent dipole-canceling aggregates between molecules due to intermolecular interactions. It should be noted here that not all monomer units in the crystal have the same arrangement, and luminescence is not completely lost.

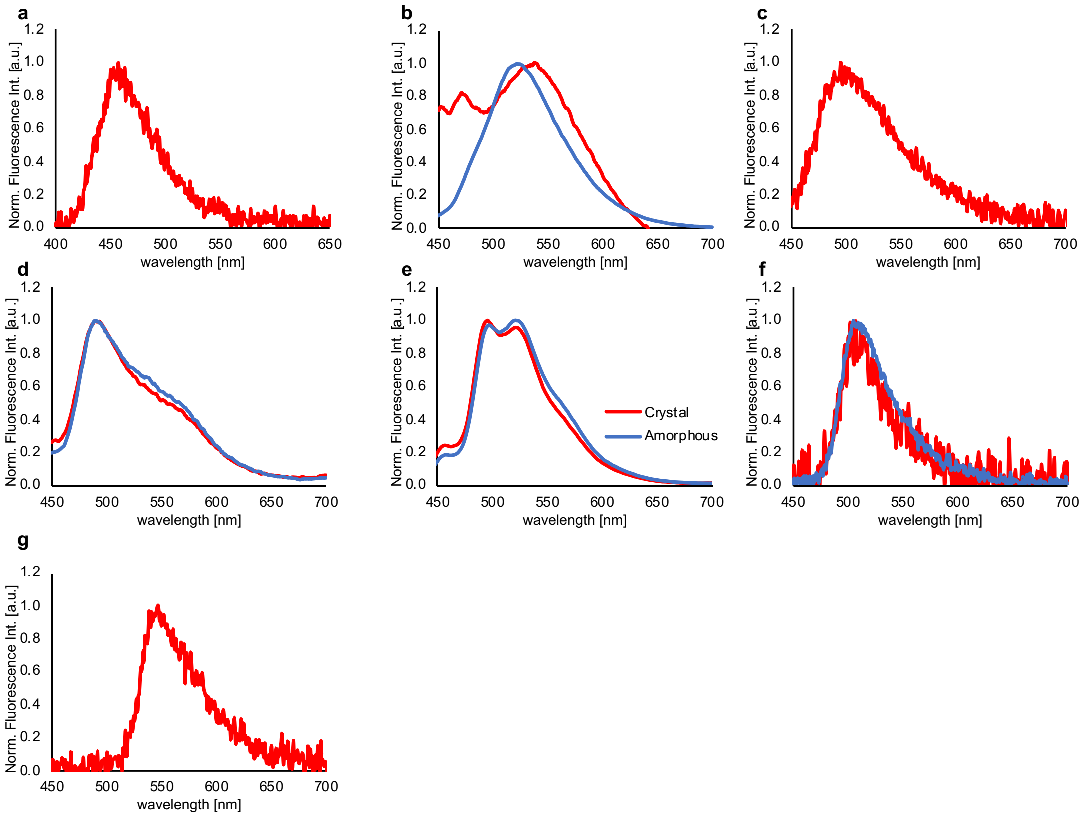

Figure S82. The fluorescence spectra in the solid-state for **a**, **FCM**. **b**, **PCM**. **c**, **FπpCM**. **d**, **FπA**. **e**, **FπCM**. **f**, **FπCM*o*-F** and **g**, **FstCM*o*-F**. The red line shows the solid amorphous state, and the blue line shows the solid crystal state.

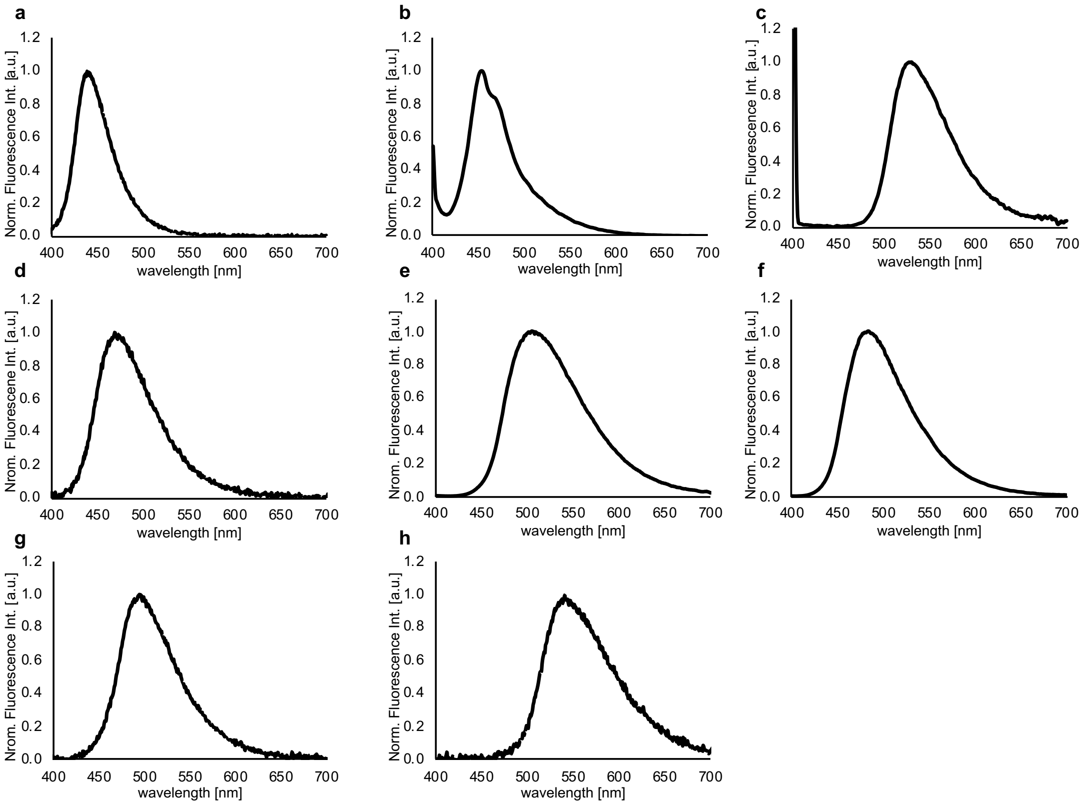

Figure S83. The fluorescence spectra of PMMA film doped with 0.10 ~ 1.0 wt% of **a**, **FCM**. **b**, **PCM**. **c**, **PK**. **d**, **FπpCM**. **e**, **FπA**. **f**, **FπCM**. **g**, **FπCM*o*-F** and **h**, **FstCM*o*-F**.

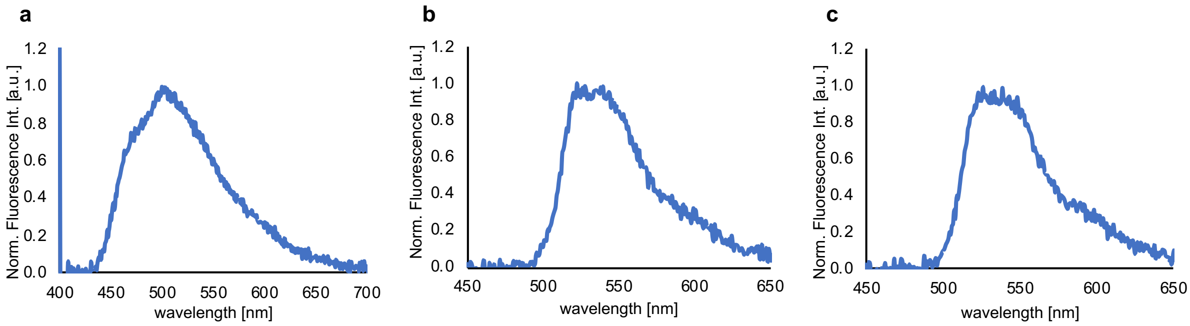

Figure S84. The fluorescence spectra in the solid-state for **a**, **FπCMst**. **b**, **PCMst**. and **c**, **FstCM**. The red line shows the solid amorphous state.

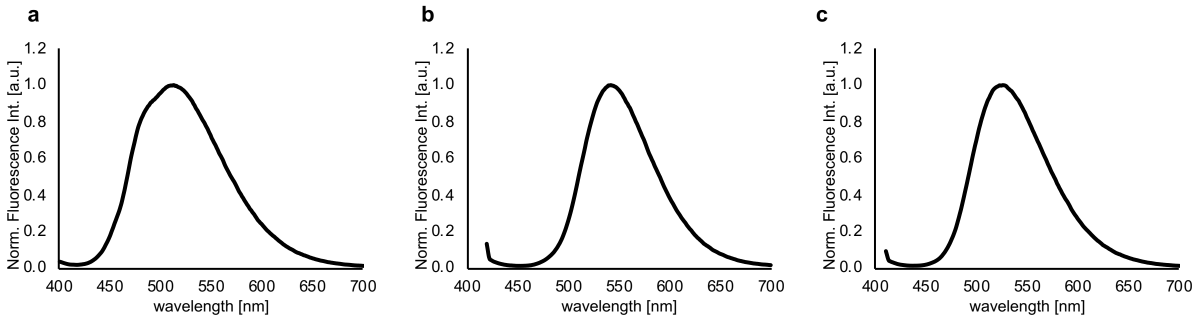

Figure S85. The fluorescence spectra of PMMA film doped with 0.10 ~ 1.0 wt% of **a**, **FπCMst**. **b**, **PCMst**. and **c**, **FstCM**.

Table S12. Optical properties in the solid-state and PMMA matrix film.

Entry	state	λ_{fl} [nm]	Φ_{fl} [-]
**FCM**	PMMA	438	0.94
	amorphous	385	0.12
	crystal	-	-
**PCM**	PMMA	455	0.70
	amorphous	522	0.09
	crystal	533	0.12
**PK**	PMMA	530	0.46
	amorphous	-	0.02
	crystal	-	-
**FπpCM**	PMMA	468	0.97
	amorphous	495	0.23
	crystal	-	-
**FπA**	PMMA	504	0.38
	amorphous	492	0.03
	crystal	492	0.03
**FπCM**	PMMA	483	0.75
	amorphous	496	0.48
	crystal	496	0.43
**FπCM*o*-F**	PMMA	493	0.88
	amorphous	505	0.54
	crystal	507	0.23
**FstCM*o*-F**	PMMA	540	0.98
	amorphous	548	0.18
	crystal	-	-
**FπCMst**	PMMA	512	0.98
	amorphous	500	0.71
	crystal	-	-
**PCMst**	PMMA	542	0.95
	amorphous	522	0.72
	crystal	-	-
**FstCM**	PMMA	525	0.92
	amorphous	525	0.70
	crystal	-	-

S3-5. Lippert-Mataga plot analysis

The solvent polarity effect on the absorption/fluorescence spectra of **FπCM**, **FπA**, **FπpCM**, **FCM**, **PK** and **PCM** was quantitatively estimated by Lippert-Mataga plots (**Figures S60-S62**), in which Stokes Shift were plotted against the solvent polarity parameter Δf defined as follows:

$$\Delta f = \frac{\varepsilon - 1}{2\varepsilon + 1} - \frac{n^2 - 1}{2n^2 + 1} \quad (\text{eq. S3})$$

where ε is relative permittivity, and n is refractive index of the solvents. From the well-known Lippert-Mataga equation S4, [S9,S10] the difference in dipole moments between the ground state (μ_g) and the excited state (μ_{ex}) was calculated for **FπCM**, **FπA**, **FπpCM**, **FCM**, **PK** and **PCM**.

$$\nu_{abs} - \nu_{fl} = \frac{2}{hc}\left(\frac{\varepsilon - 1}{2\varepsilon + 1} - \frac{n^2 - 1}{2n^2 + 1}\right)\frac{\mu_e - \mu_g}{\alpha^3} + \text{const.} \quad (\text{eq. S4})$$

The parameter a is the Onsager's cavity radius, which was estimated to be 5.80 Å for **FπCM** and **FπpCM**, 5.59 Å for **FπA**, 4.86 Å for **FCM**, 4.84 Å for **PCM** and 4.53 Å for **PK** by DFT calculations (ωB97XD/6-311G(d,p)). Since the coefficient of Δf in eq. 1 corresponds to the slope b in **Figures S86**, **S87** and **S88**, the difference in transient dipole moment $\Delta\mu$ (μ_{ex}-μ_g) was calculated to be 17.2 Debye (D), 16.7 D, 16.9 D, 13.8 D, 9.30 D and 9.40 D, respectively.

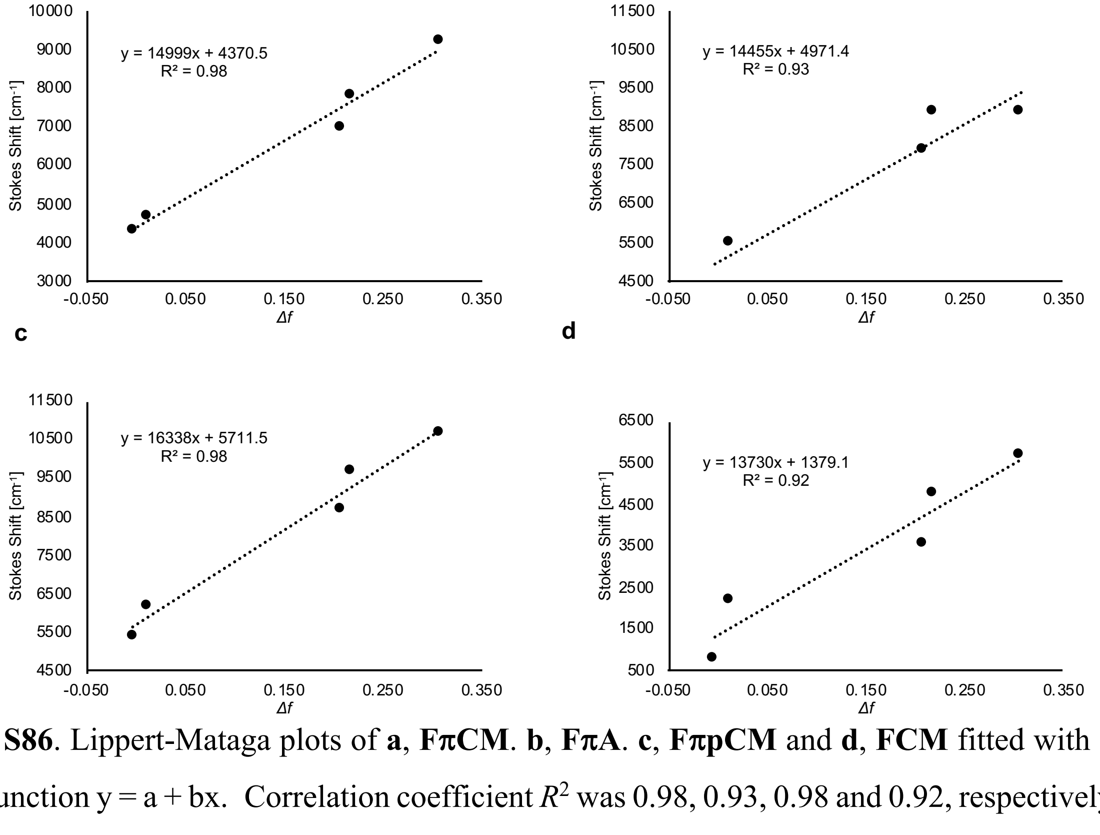

Figure S86. Lippert-Mataga plots of **a**, **FπCM**. **b**, **FπA**. **c**, **FπpCM** and **d**, **FCM** fitted with a linear function y = a + bx. Correlation coefficient R^2 was 0.98, 0.93, 0.98 and 0.92, respectively.

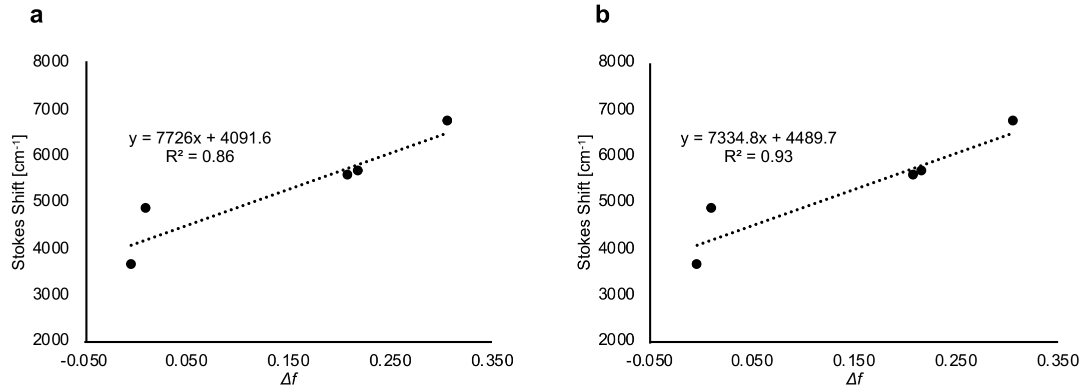

Figure S87. Lippert-Mataga plots of **a**, **PK**, and **b**, **PCM** fitted with a linear function y = a + bx. Correlation coefficient R^2 was 0.86 and 0.92, respectively.

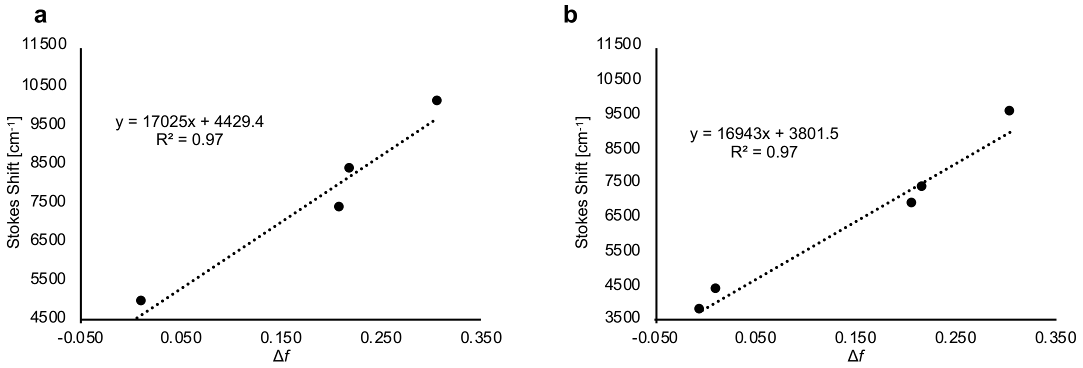

Figure S88. Lippert-Mataga plots of **a**, **FπCM*o*-F**, and **b**, **FstCM*o*-F** fitted with a linear function y = a + bx. Correlation coefficient R^2 was 0.97 and 0.97, respectively.

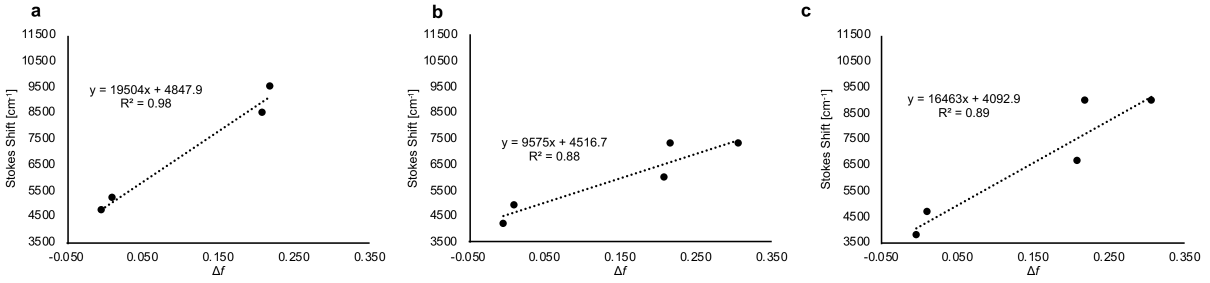

Figure S89. Lippert-Mataga plots of **a**, **FπCMst**, **b**, **PCMst**, and **c**, **FstCM** fitted with a linear function y = a + bx. Correlation coefficient R^2 was 0.98, 0.88 and 0.89, respectively.

Table S13. Summary of the results of Lippert-Mataga plot. [a]

Entry	slope	r^2	μ_g [D] [c]	$\Delta\mu$ [D]	μ_{ex} [D]	a_0 [Å]
**FCM**	13700	0.92	3.90	14.5	18.4	5.15
**PCM**	7700	0.86	3.58	9.30	12.9	4.84
**PK**	7800	0.93	4.53	9.40	13.9	4.53
**FπpCM**	16300	0.98	4.13	16.9	21.0	5.80
**FπA**	14500	0.93	5.62	16.7	22.3	5.59
**FπCM**	15000	0.98	4.13	17.2	21.3	5.59
**FπCM*o*-F**	17000	0.97	4.23	17.2	21.4	5.59
**FstCM*o*-F**	16900	0.97	7.19	19.0	26.2	6.00
**FπCMst**	19500	0.98	6.58	20.5	27.1	6.00
**PCMst**	9600	0.88	4.04	14.5	18.5	6.00
**FstCM**	16500	0.89	6.53	18.7	25.2	6.00
**Prodan**[b]	6600	0.74	5.98	7.00	13.0	4.00

a. Definitions. r^2 = the goodness of fit. μ_g = dipole moment in the ground state. $\Delta\mu$ = transition dipole moment ($\mu_{ex} - \mu_g$). μ_{ex} = dipole moment in the excited state. b. the data of **Prodan** was referred ref. S1. c. μ_g was estimated by DFT calculations at ωB97XD/6-311G** level of theory.

S4. Crystallographic information

Diffraction data were collected on a Rigaku XtaLAB Synergy R, DW HyPix system using CuKα radiation (λ = 1.54184 Å) to a maximum 2θ value of 85.9° at -150 °C. Equivalent reflections were merged and the images were processed with Rigaku CrysAlis[Pro] (1.171). The structure solution was performed using Olex2 (v1.5)[S11] as a graphical user interface (GUI). The structure was solved by SHELXT (2018/2)[S12] and refined by SHELXL (2018/3).[S13] Disordered moieties were refined by DSR program.[S14,S15] Non-hydrogen atoms were refined anisotropically. Hydrogen atoms were refined using a riding model. The data can be obtained free of charge via http://www.ccdc.cam.ac.uk/conts/retrieving.html. **FπCM**: CCDC2303310, **FπCM*o*-F**: CCDC2303308, **FπA**: CCDC2303309, **PCM**: CCDC2303312, **FstCM*o*-F**: CCDC2303311

Table S14. Crystallographic data and structure refinement details for **FπCM**, **FπA**, **PCM**, **FπCM*o*-F** and **FstCM*o*-F**.

	**FπCM**	**FπA**	**PCM**	**FπCM*o*-F**	**FstCM*o*-F**
Empirical formula	$C_{27}H_{29}NO_2$	$C_{27}H_{29}NO$	$C_{23}H_{21}NO_2$	$(C_{27}H_{28}FNO_2)_2$	$C_{35}H_{42}FNO_2$
Formula weight	399.53	383.54	343.43	835.0	527.72

Tempetature/K	123	123	123	123	123
Space group	$P2_1/c$	$P2_1$	$P\bar{1}$	$P\bar{1}$	$R\bar{3}$ (148)
Crystal system	monoclinic	monoclinic	triclinic	triclinic	trigonal
color	colorless	yellow	light yellow	light yellow	yellow needle
a/Å	10.83950(10)	10.68510(10)	7.7620(2)	10.08390(10)	36.3095(6)
b/Å	7.04370(10)	7.05670(10)	11.3678(3)	12.3737(2)	36.3095(6)
c/Å	28.4228(3)	14.2545	11.4120(3)	19.1167(2)	12.0062(2)
a/°	90	90	113.519(2)	75.3690(10)	90
b/°	95.5850(10)	95.0150(10)	101.140(2)	86.4010(10)	90
g/°	90	90	103.695(2)	68.9950(10)	120
Volume/Å^3	2159.78(4)	1070.70(2)	849.00(4)	2153.56(5)	13708.1(5)
Z	4	2	2	2	18
r_{calc}/g cm^{-3}	1.229	1.190	1.343	1.288	1.182
Radiation	CuKα (λ= 1.54184 Å)	CuKα (λ= 1.54184 Å)	CuKα (λ= 1.54184 Å)	CuKα (λ= 1.54184 Å)	CuKα (λ= 1.54184 Å)
m/mm^{-1}	0.598	0.546	0.674	0.692	0.597
F(000)	856.0	412.0	364.0	888.0	5262
Goodness-of-fit on F^2	1.044	1.083	1.074	1.048	1.068
Final R indexes [I≧2s (I)]	R_1 = 0.0355 wR_2 = 0.0934	R_1 = 0.0306 wR_2 = 0.0807	R_1 = 0.0356 wR_2 = 0.1012	R_1 = 0.0403 wR_2 = 0.1115	R_1 = 0.0483 wR_2 = 0.1230
Final R indexes [all data]	R_1 = 0.0384 wR_2 = 0.0957	R_1 = 0.0311 wR_2 = 0.0811	R_1 = 0.0380 wR_2 = 0.1034	R_1 = 0.0435 wR_2 = 0.1142	R_1 = 0.0617 wR_2 = 0.1323
CCDC	2303310	2303309	2303312	2303308	2303311

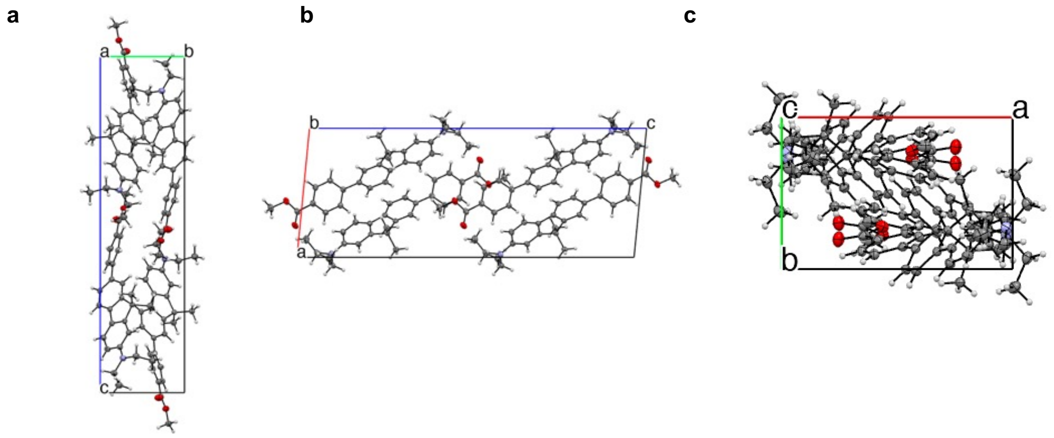

Figure S90. Crystal structure of **FπCM** viewed from different directions. (a) a-axis. (b) b-axis. (c) c-axis. Hydrogen atoms except for those in the disordered groups with low occupancy are omitted for clarity. Thermal ellipsoids are scaled to 50% probability. CCDC2303310

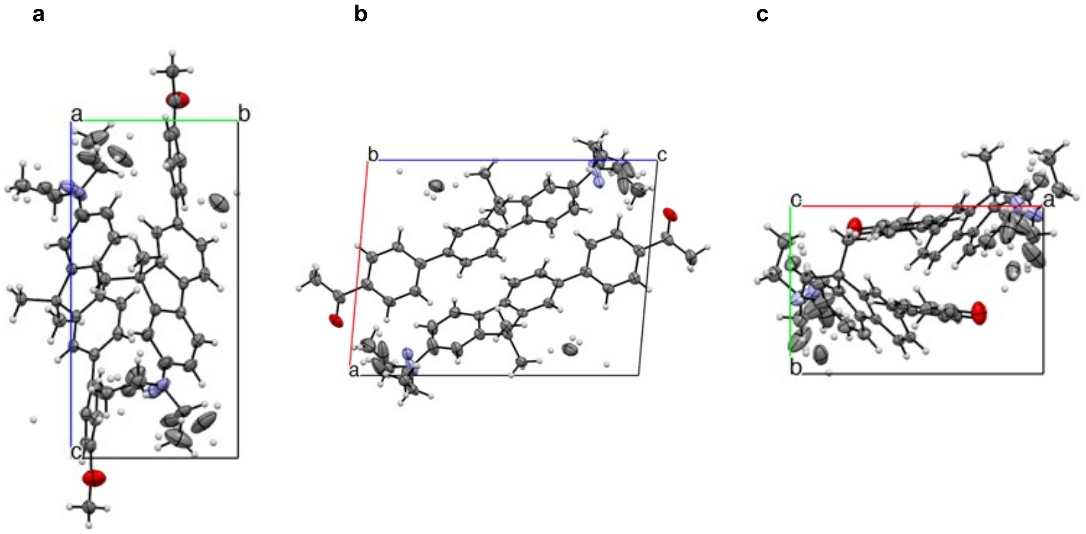

Figure S91. Crystal structure of **FπA** viewed from different directions. (a) a-axis. (b) b-axis. (c) c-axis. Hydrogen atoms except for those in the disordered groups with low occupancy are omitted for clarity. Thermal ellipsoids are scaled to 50% probability. CCDC2303309

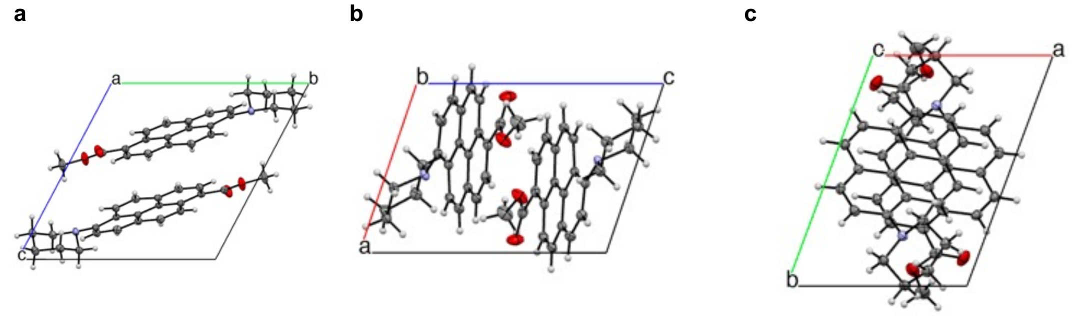

Figure S92. Crystal structure of **PCM** viewed from different directions. (a) a-axis. (b) b-axis. (c) c-axis. Hydrogen atoms except for those in the disordered groups with low occupancy are omitted for clarity. Thermal ellipsoids are scaled to 50% probability. CCDC23033121

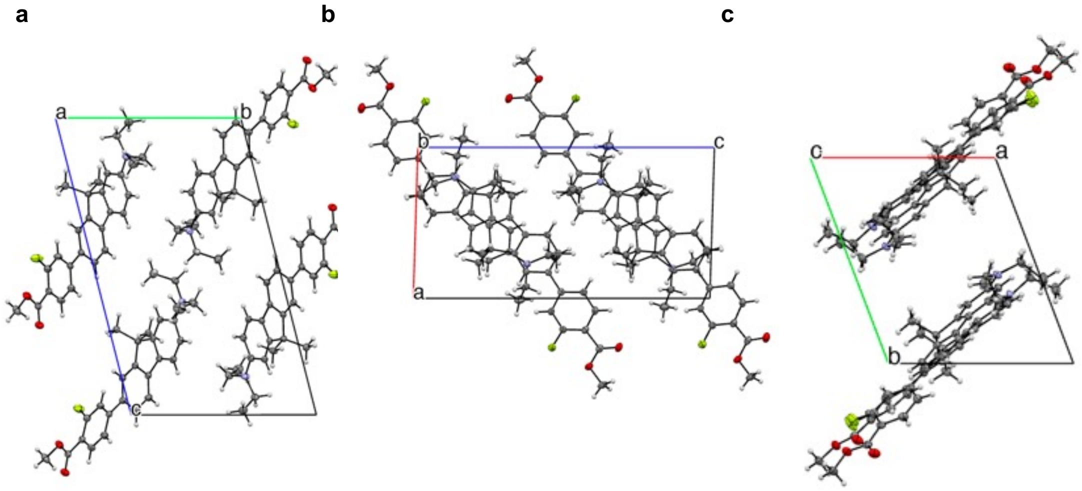

Figure S93. Crystal structure of **FπCMo-F** viewed from different directions. (a) a-axis. (b) b-axis. (c) c-axis. Hydrogen atoms except for those in the disordered groups with low occupancy are omitted for clarity. Thermal ellipsoids are scaled to 50% probability. CCDC2303308

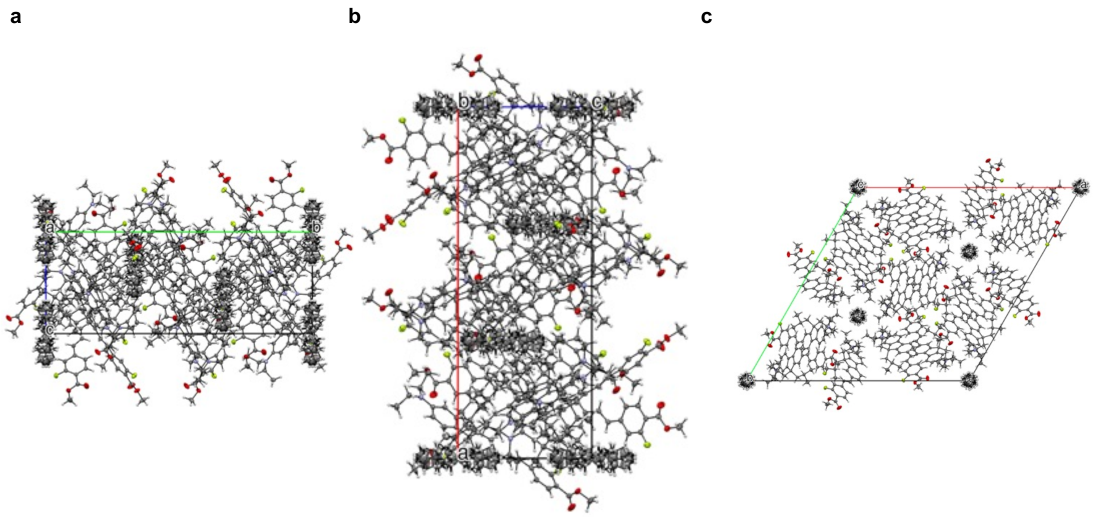

Figure S94. Crystal structure of **FstCMo-F** viewed from different directions. (a) a-axis. (b) b-axis. (c) c-axis. Hydrogen atoms except for those in the disordered groups with low occupancy are omitted for clarity. Thermal ellipsoids are scaled to 50% probability. CCDC2303311

S5. Theoretical calculations

Quantum calculations were carried out with Gaussian 09 and 16 program.[S16,S17] The structures were optimized by DFT method at **ωB97XD/6-31G**** for **FπCM**, **FπA**, **FCM**, **PCM**, **PK** and **Prodan**, **B3LYP/6-311+G** for FπCM**, **FπA**, **FπCMst**, **PCMst** and **FstCM** **ωB97XD/6-311+G** for PCM** and **PK** level of theory. The frequency analysis was carried out for each-minimum structure to give no imaginary wavenumber. The molecular orbitals and energies were calculated by using the optimized structures. The solvent effects were considered by the solute electron density model (SMD) and the polarizable continuum model (PCM) with integral equation formalism (IEF).

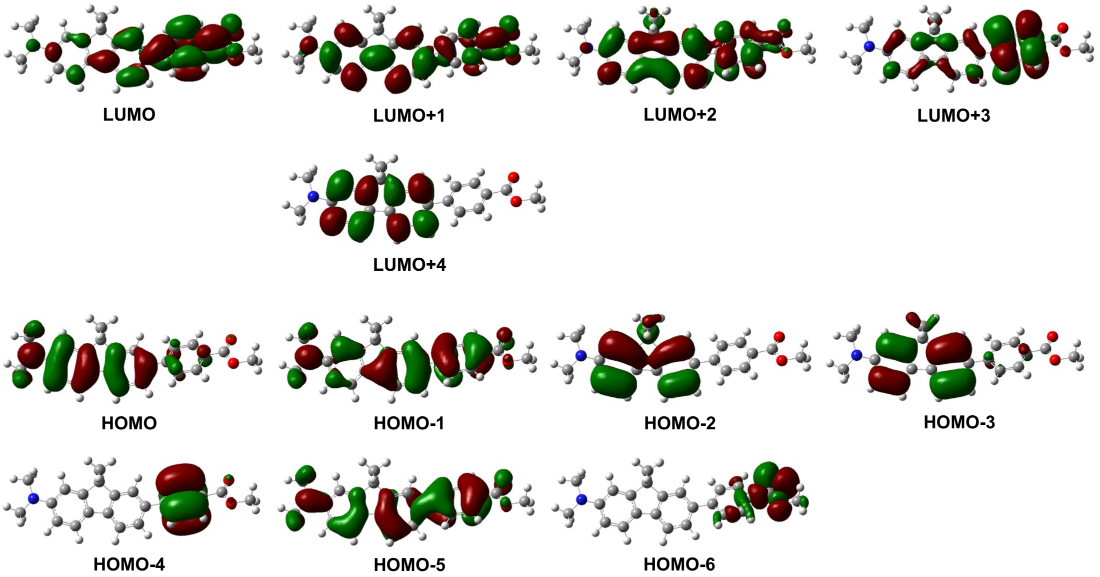

Figure S95. The Molecular orbitals of **FπCM** calculated by DFT (ωB97XD/6-31G(d,p)).

Table S15. Excitation energy, oscillator strength, main transient orbital, attributed transition for **FπCM** calculated using TD-DFT(ωB97XD/6-31G(d,p)).

State	Excitation energy [eV]	Oscillator strength	Main transition orbital		Contribution	transition
T1	2.0275	0	HOMO-5	LUMO+1	0.11690	π-π*
			HOMO-1	LUMO+5	0.10862	π-π*
			HOMO	LUMO	0.57971	π-π*
T2	3.0730	0	HOMO-1	LUMO	0.41563	π-π*
			HOMO	LUMO+5	0.18459	π-π*
S1	3.2380	1.7311	HOMO	LUMO	0.64861	π-π*
T3	3.6672	0	HOMO-1	LUMO+3	0.11842	π-π*
			HOMO	LUMO+3	0.46262	π-π*

T4	3.9461	0	HOMO-9	LUMO	0.16746	π-π*
			HOMO-8	LUMO	0.12563	π-π*
			HOMO-5	LUMO	0.26069	π-π*
			HOMO-4	LUMO+2	0.13688	π-π*
			HOMO-4	LUMO+3	0.10333	π-π*
			HOMO-3	LUMO	0.10894	π-π*
			HOMO-2	LUMO+2	0.12611	π-π*
			HOMO-1	LUMO+2	0.15617	π-π*
			HOMO	LUMO+4	0.19202	π-π*
			HOMO	LUMO+5	0.26375	π-π*
			HOMO	LUMO+6	0.16663	π-π*
T5	4.1385	0	HOMO-3	LUMO	0.19406	π-π*
			HOMO-2	LUMO	0.13482	π-π*
			HOMO-1	LUMO+1	0.19330	π-π*
			HOMO-1	LUMO+2	0.20577	π-π*
			HOMO	LUMO+4	0.32066	π-π*
T6	4.2442	0	HOMO-4	LUMO	0.53810	π-π*
			HOMO-4	LUMO+1	0.20026	π-π*
			HOMO	LUMO+4	0.24924	π-π*
S2	4.3463	0.0087	HOMO-3	LUMO	0.17555	π-π*
			HOMO-1	LUMO	0.20026	π-π*
			HOMO	LUMO+1	0.26382	π-π*
			HOMO	LUMO+2	0.44849	π-π*
S3	4.5967	0.0474	HOMO-1	LUMO	0.466683	π-π*
			HOMO	LUMO+2	0.18072	π-π*
			HOMO	LUMO+5	0.11806	π-π*
S4	4.6533	0.0071	HOMO-4	LUMO	0.13398	π-π*
			HOMO-2	LUMO+1	0.12577	π-π*
			HOMO-1	LUMO+2	0.21500	π-π*
			HOMO	LUMO+4	0.33252	π-π*
S5	4.8595	0.0087	HOMO-4	LUMO	0.50997	π-π*
			HOMO-4	LUMO+1	0.13614	π-π*
			HOMO-2	LUMO	0.11303	π-π*
			HOMO-1	LUMO+2	0.10654	π-π*
			HOMO-1	LUMO+3	0.19468	π-π*
			HOMO-1	LUMO+4	0.11664	π-π*
S6	5.1309	0.0009	HOMO-6	LUMO	0.54951	n-π*
			HOMO-6	LUMO+1	0.35038	n-π*
			HOMO-6	LUMO+5	0.19735	n-π*

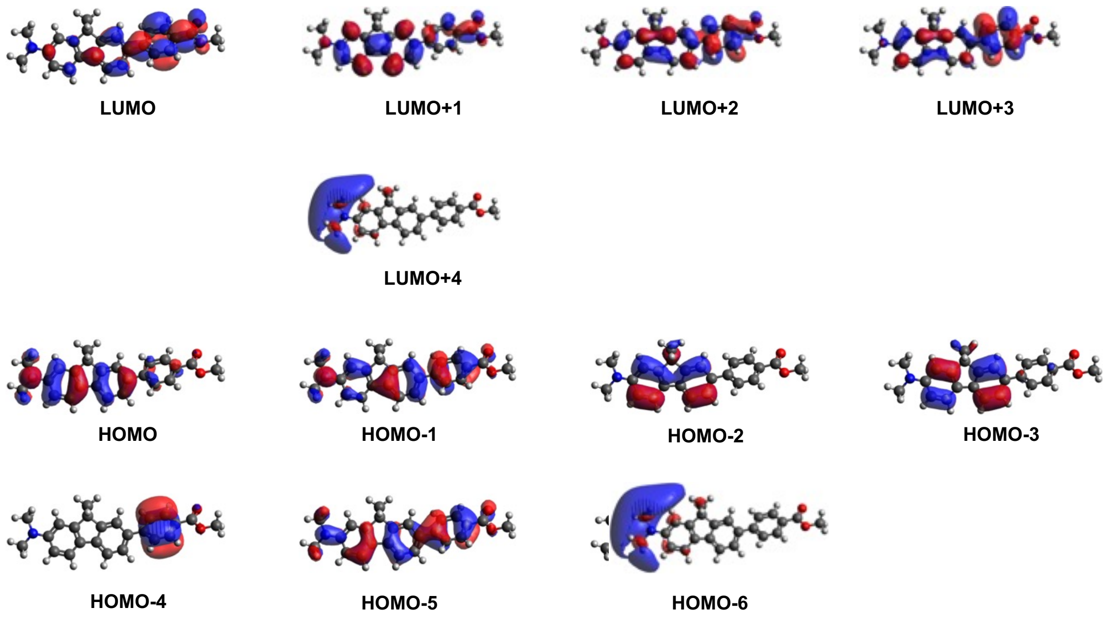

Figure S96. The Molecular orbitals of **FπCM** calculated by DFT (B3LYP/6-311+G(d,p)).

Table S16. Excitation energy, oscillator strength, main transient orbital, attributed transition for **FπCM** calculated using TD-DFT(B3LYP/6-311+G(d,p)).

State	Excitation energy [eV]	Oscillator strength	Main transition orbital		Contribution	transition
T1	2.0951	0	HOMO	LUMO	0.64113	π-π*
			HOMO	LUMO+1	0.15033	π-π*
S1	2.6302	1.0104	HOMO	LUMO	0.70517	π-π*
T2	2.9728	0	HOMO-5	LUMO	0.20391	π-π*
			HOMO-1	LUMO	0.45263	π-π*
			HOMO	LUMO+1	0.41232	π-π*
T3	3.2397	0	HOMO	LUMO+1	0.48201	π-π*
			HOMO	LUMO+2	0.13530	π-π*
			HOMO	LUMO+5	0.10571	π-π*
T4	3.2681	0	HOMO-1	LUMO	0.24003	π-π*
			HOMO	LUMO	0.22435	π-π*
			HOMO	LUMO+2	0.46165	π-π*
			HOMO	LUMO+5	0.10364	π-π*
S2	3.6612	0.1206	HOMO	LUMO+1	0.60930	π-π*
			HOMO	LUMO+2	0.26920	π-π*
T5	3.6640	0	HOMO-1	LUMO+2	0.16706	π-π*
			HOMO	LUMO+5	0.43480	π-π*
T6	3.8502	0	HOMO-6	LUMO	0.26699	π-π*
			HOMO-5	LUMO	0.34802	π-π*
			HOMO-4	LUMO+5	0.11858	π-π*
			HOMO-1	LUMO+2	0.10847	π-π*
			HOMO	LUMO+2	0.14008	π-π*

			HOMO	LUMO+3	0.14329	π-π*
			HOMO	LUMO+5	0.10404	π-π*
			HOMO	LUMO+10	0.19961	π-π*
S3	3.8770	0.5521	HOMO-1	LUMO	0.38645	π-π*
			HOMO	LUMO+2	0.53273	π-π*
S4	4.0194	0.0307	HOMO	LUMO+2	0.30995	π-π*
			HOMO	LUMO+3	0.47410	π-π*
S5	4.0394	0.0374	HOMO-1	LUMO	0.45131	π-π*
			HOMO	LUMO+1	0.25151	π-π*
			HOMO	LUMO+3	0.43108	π-π*
S6	4.2267	0.0052	HOMO-2	LUMO	0.45307	π-π*
			HOMO	LUMO+3	0.11450	π-π*
			HOMO	LUMO+5	0.39539	π-π*

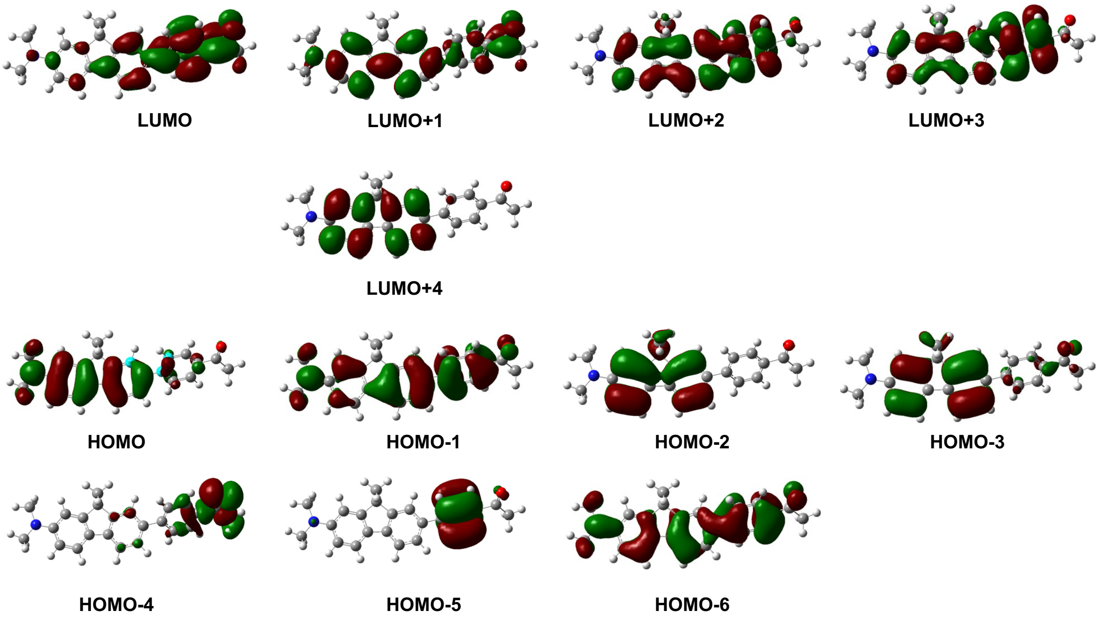

Figure S97. The Molecular orbitals of **FπA** calculated by DFT (ωB97XD/6-31G(d,p)).

Table S17. Excitation energy, oscillator strength, main transient orbital, attributed transition for **FπA** calculated using TD-DFT(ωB97XD/6-31G(d,p)).

State	Excitation energy [eV]	Oscillator strength	Main transition orbital		Contribution	transition
T1	2.5611	0	HOMO-5	LUMO+1	0.12778	π-π*
			HOMO-1	LUMO	0.44268	π-π*
T2	2.7953	0	HOMO-3	LUMO	0.59432	π-π*
			HOMO-3	LUMO+5	0.17360	π-π*
			HOMO-3	LUMO+6	0.11093	π-π*
			HOMO-4	LUMO	0.14947	n-π*
T3	3.0155	0	HOMO-1	LUMO	0.20860	π-π*

			HOMO	LUMO	0.17104	π-π*
			HOMO	LUMO+1	0.45345	π-π*
			HOMO	LUMO+5	0.13825	π-π*
S1	3.3154	0.0029	HOMO-3	LUMO	0.60550	π-π*
			HOMO-3	LUMO+5	0.15819	π-π*
			HOMO-2	LUMO	0.15726	π-π*
T4	3.7838	0	HOMO-7	LUMO+4	0.10084	π-π*
			HOMO-1	LUMO+4	0.11921	π-π*
			HOMO	LUMO+1	0.16529	π-π*
			HOMO	LUMO+2	0.36100	π-π*
			HOMO	LUMO+3	0.36867	π-π*
			HOMO	LUMO+4	0.38653	π-π*
S2	3.8159	1.5745	HOMO	LUMO	0.55284	π-π*
			HOMO	LUMO+1	0.31185	π-π*
T5	3.8719	0	HOMO-4	LUMO+2	0.11466	n-π*
			HOMO-2	LUMO+2	0.18177	π-π*
			HOMO-2	LUMO+3	0.17760	π-π*
			HOMO	LUMO+4	0.14108	π-π*
			HOMO	LUMO+6	0.17341	π-π*
T6	4.0451	0	HOMO-5	LUMO	0.58144	π-π*
S3	4.5172	0.0059	HOMO-1	LUMO	0.14384	π-π*
			HOMO-1	LUMO+4	0.11419	π-π*
			HOMO	LUMO+1	0.28128	π-π*
			HOMO	LUMO+2	0.35716	π-π*
			HOMO	LUMO+3	0.34961	π-π*
			HOMO	LUMO+5	0.11589	π-π*
S4	4.7352	0.0240	HOMO-1	LUMO	0.38189	π-π*
			HOMO	LUMO+1	0.34243	π-π*
			HOMO	LUMO+5	0.11589	π-π*
S5	4.7819	0.0463	HOMO-5	LUMO	0.48291	π-π*
			HOMO-1	LUMO	0.13635	π-π*
			HOMO-1	LUMO+3	0.14976	π-π*
			HOMO	LUMO+1	0.17329	π-π*
S6	4.8945	0.0014	HOMO-4	LUMO	0.26352	n-π*
			HOMO-4	LUMO+1	0.16317	n-π*
			HOMO-2	LUMO	0.22112	π-π*
			HOMO-2	LUMO+1	0.19651	π-π*
			HOMO-1	LUMO+2	0.13808	π-π*
			HOMO-1	LUMO+3	0.13566	π-π*
			HOMO	LUMO+4	0.41130	π-π*

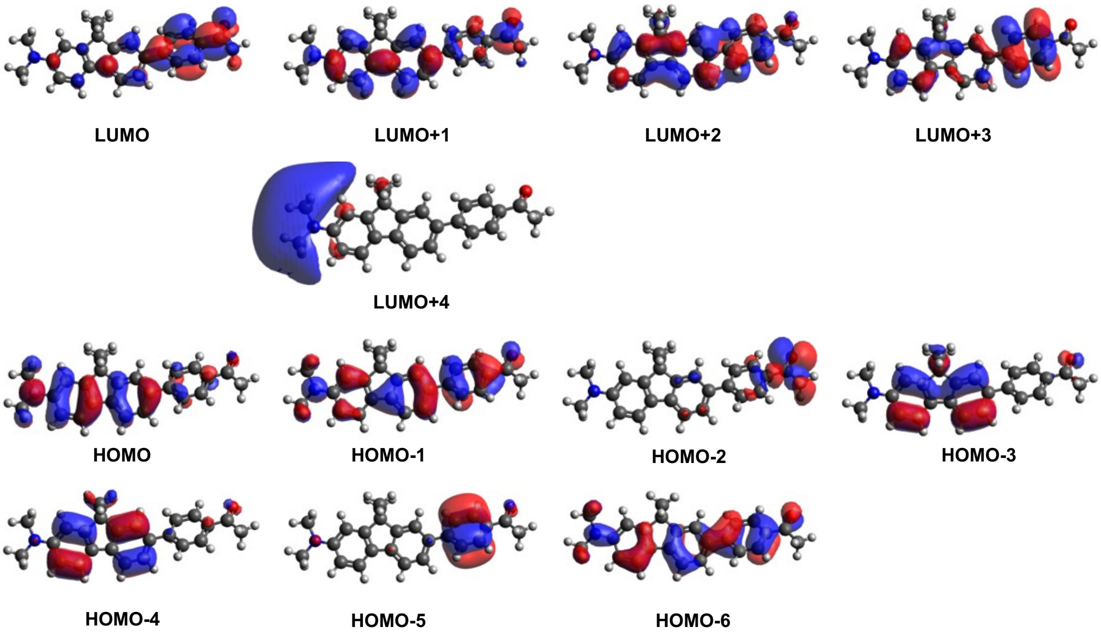

Figure S98. The Molecular orbitals of **FπA** calculated by DFT (B3LYP/6-311+G(d,p)).

Table S18. Excitation energy, oscillator strength, main transient orbital, attributed transition for **FπA** calculated using TD-DFT(B3LYP/6-311+G(d,p)).

State	Excitation energy [eV]	Oscillator strength	Main transition orbital		Contribution	transition
T1	2.1053	0	HOMO-1	LUMO	0.19445	π-π*
			HOMO	LUMO	0.63580	π-π*
S1	2.5395	0.7803	HOMO	LUMO	0.70524	π-π*
T2	2.8885	0	HOMO-6	LUMO	0.22506	π-π*
			HOMO-1	LUMO	0.39427	π-π*
			HOMO	LUMO+1	0.48257	π-π*
T3	3.0608	0	HOMO-4	LUMO	0.35171	π-π*
			HOMO-4	LUMO+1	0.10131	π-π*
			HOMO-3	LUMO	0.54696	π-π*
			HOMO-3	LUMO+1	0.15175	π-π*
T4	3.1130	0	HOMO-6	LUMO	0.14719	π-π*
			HOMO-1	LUMO	0.41223	π-π*
T5	3.2491	0	HOMO	LUMO+1	0.15074	π-π*
			HOMO	LUMO+2	0.50064	π-π*
			HOMO	LUMO+3	0.39923	π-π*
			HOMO	LUMO+5	0.18717	π-π*
S2	3.5128	0.0001	HOMO-4	LUMO	0.36145	π-π*
			HOMO-3	LUMO	0.56013	π-π*
			HOMO-3	LUMO+1	0.12590	π-π*
S3	3.6540	0.3740	HOMO	LUMO+1	0.59426	π-π*
			HOMO	LUMO+2	0.29126	π-π*
			HOMO	LUMO+3	0.16262	π-π*

T6	3.6898	0	HOMO-6	LUMO	0.12092	π-π*
			HOMO-2	LUMO+2	0.10065	n-π*
			HOMO	LUMO+3	0.17005	π-π*
			HOMO	LUMO+5	0.49222	π-π*
S4	3.8057	0.4284	HOMO-1	LUMO	0.26072	π-π*
			HOMO	LUMO+2	0.54417	π-π*
			HOMO	LUMO+3	0.21168	π-π*
S5	3.9686	0.0847	HOMO-1	LUMO	0.63239	π-π*
			HOMO	LUMO+1	0.24041	π-π*
S6	4.0040	0.0058	HOMO	LUMO+3	0.62467	π-π*

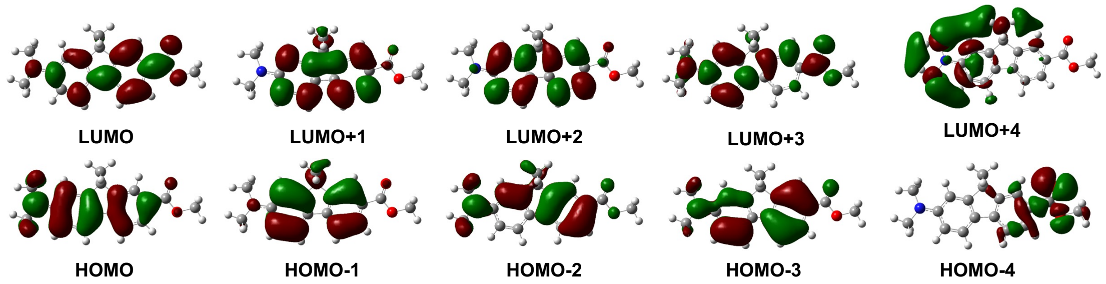

Figure S99. The Molecular orbitals of **FCM** calculated by DFT (ωB97XD/6-31G(d,p)).

Table S19. Excitation energy, oscillator strength, main transient orbital, attributed transition for **FCM** calculated using TD-DFT(ωB97XD/6-31G(d,p)).

State	Excitation energy [eV]	Oscillator strength	Main transition orbital		Contribution	transition
T1	2.2329	0	HOMO	LUMO	0.63931	π-π*
			HOMO	LUMO+3	0.14869	π-π*
S1	3.5111	1.1856	HOMO	LUMO	0.68171	π-π*
T2	3.6834	0	HOMO-6	LUMO	0.18382	π-π*
			HOMO-1	LUMO	0.39923	π-π*
			HOMO	LUMO+1	0.30174	π-π*
			HOMO	LUMO+3	0.32799	π-π*
T3	3.7373	0	HOMO	LUMO+1	0.51788	π-π*
			HOMO	LUMO+2	0.39724	π-π*
T4	4.2363	0	HOMO-3	LUMO	0.45908	π-π*
			HOMO-2	LUMO	0.46475	π-π*
T5	4.3971	0	HOMO-2	LUMO	0.11614	π-π*
			HOMO-1	LUMO	0.14692	π-π*
			HOMO-1	LUMO+1	0.25968	π-π*
			HOMO	LUMO+2	0.40552	π-π*
			HOMO	LUMO+3	0.32281	π-π*
S2	4.4285	0.0198	HOMO-1	LUMO+2	0.10020	π-π*
			HOMO	LUMO+1	0.62009	π-π*
			HOMO	LUMO+2	0.21758	π-π*

T6	4.4813	0	HOMO-3	LUMO	0.17820	π-π*
			HOMO-3	LUMO+1	0.37532	π-π*
			HOMO-3	LUMO+3	0.10202	π-π*
S3	4.7670	0.0003	HOMO-3	LUMO	0.24238	π-π*
			HOMO-2	LUMO	0.42789	π-π*
			HOMO-1	LUMO+1	0.16782	π-π*
			HOMO	LUMO+2	0.39477	π-π*
			HOMO	LUMO+3	0.16039	π-π*
S4	5.1386	0.0003	HOMO-4	LUMO	0.60944	n-π*
			HOMO-4	LUMO+2	0.12271	n-π*
S5	5.3534	0.0477	HOMO-3	LUMO	0.11851	π-π*
			HOMO-1	LUMO	0.52591	π-π*
			HOMO	LUMO+1	0.13603	π-π*
			HOMO	LUMO+3	0.29159	π-π*
S6	5.5728	0.1383	HOMO-3	LUMO	0.52382	π-π*
			HONO	LUMO+1	0.23963	π-π*

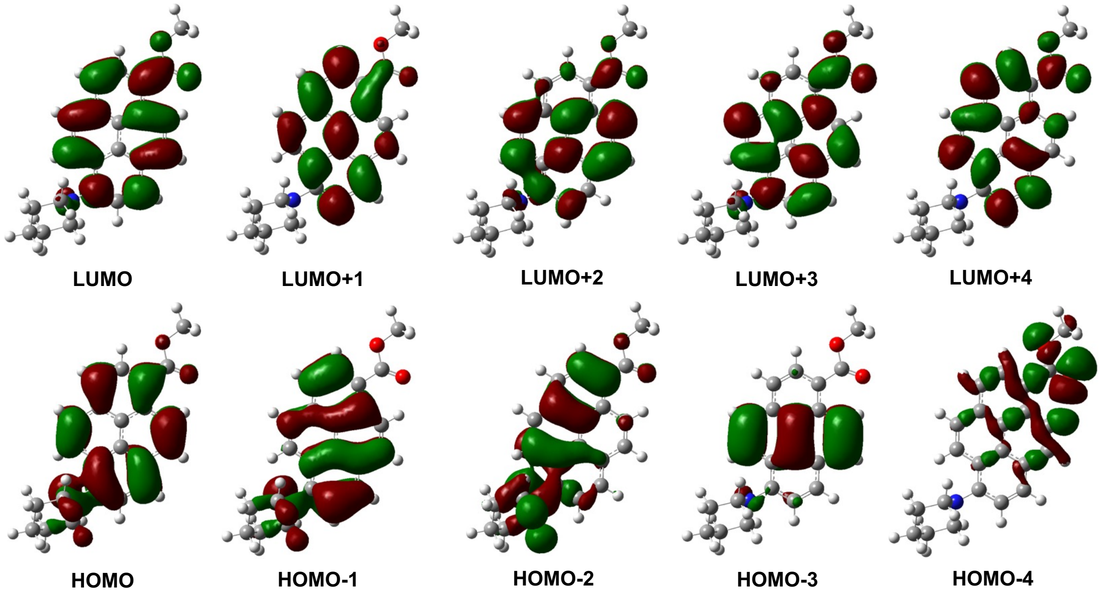

Figure S100. The Molecular orbitals of **PCM** calculated by DFT (ωB97XD/6-31G(d,p)).

Table S20. Excitation energy, oscillator strength, main transient orbital, attributed transition for **PCM** calculated using TD-DFT(ωB97XD/6-31G(d,p)).

State	Excitation energy [eV]	Oscillator strength	Main transition orbital		Contribution	transition
T1	1.3537	0	HOMO-1	LUMO	0.11037	π-π*
			HOMO	LUMO	0.69484	π-π*
S1	2.8616	0.8559	HOMO	LUMO	0.69518	π-π*
T2	3.2489	0	HOMO-5	LUMO	0.19527	π-π*
			HOMO-1	LUMO	0.46591	π-π*
T3	3.3268	0	HOMO	LUMO+1	0.26985	π-π*
			HOMO	LUMO+2	0.41540	π-π*
T4	3.4013	0	HOMO-3	LUMO	0.19264	π-π*
			HOMO-1	LUMO	0.28204	π-π*
			HOMO-1	LUMO+1	0.11882	π-π*
			HOMO	LUMO+1	0.51987	π-π*
T5	3.5155	0	HOMO-2	LUMO	0.49736	π-π*
			HOMO-1	LUMO	0.32153	π-π*
			HOMO	LUMO+3	0.24809	π-π*
S2	3.6746	0.0022	HOMO	LUMO+1	0.52423	π-π*
T6	3.9340	0	HOMO-2	LUMO+1	0.19721	π-π*
			HOMO-1	LUMO+1	0.35031	π-π*
			HOMO	LUMO+3	0.24155	π-π*
S3	4.3661	0.0110	HOMO	LUMO+2	0.57727	π-π*
			HOMO	LUMO+3	0.16187	π-π*
S4	4.3816	0.1714	HOMO-1	LUMO	0.49423	π-π*
			HOMO	LUMO+1	0.34483	π-π*
S5	4.5587	0.2466	HOMO-2	LUMO	0.55515	π-π*
			HOMO-1	LUMO	0.10791	π-π*
			HOMO	LUMO+1	0.34483	π-π*
			HOMO	LUMO+3	0.16282	π-π*
S6	4.6054	0.1735	HOMO-3	LUMO	0.50553	π-π*
			HOMO-1	LUMO	0.21713	π-π*
			HOMO	LUMO+1	0.12010	π-π*
			HOMO	LUMO+2	0.35567	π-π*

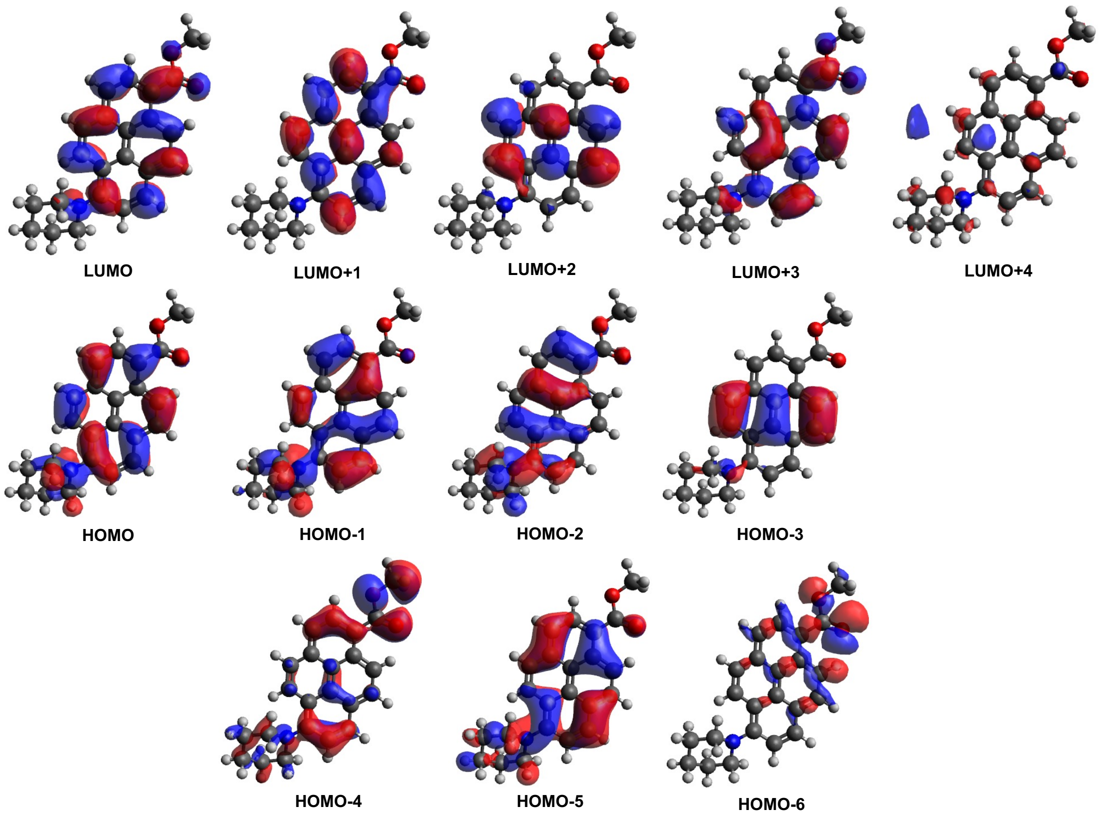

Figure S101. The Molecular orbitals of **PCM** calculated by DFT (ωB97XD/6-311+G(d,p)).

Table S21. Excitation energy, oscillator strength, main transient orbital, attributed transition for **PCM** calculated using TD-DFT(ωB97XD/6-311+G(d,p)).

State	Excitation energy [eV]	Oscillator strength	Main transition orbital		Contribution	transition
T1	1.3212	0	HOMO-1	LUMO	0.10113	π-π*
			HOMO	LUMO	0.69923	π-π*
S1	2.7449	0.8816	HOMO	LUMO	0.69511	π-π*
T2	3.1708	0	HOMO-2	LUMO	0.12469	π-π*
			HOMO	LUMO+1	0.44024	π-π*
			HOMO	LUMO+4	0.10235	π-π*
T3	3.2723	0	HOMO-3	LUMO	0.27618	π-π*
			HOMO-1	LUMO	0.17489	π-π*
			HOMO	LUMO+1	0.35662	π-π*
			HOMO	LUMO+3	0.18467	π-π*
T4	3.3323	0	HOMO-1	LUMO	0.23823	π-π*
			HOMO-1	LUMO+2	0.10332	π-π*
			HOMO	LUMO+1	0.36992	π-π*
			HOMO	LUMO+2	0.32703	π-π*
			HOMO	LUMO+3	0.15191	π-π*
T5	3.4823	0	HOMO-3	LUMO	0.12360	π-π*
			HOMO-2	LUMO	0.48788	π-π*

			HOMO-1	LUMO	0.36587	π-π*
S2	3.5905	0.0069	HOMO	LUMO+1	0.55207	π-π*
T6	3.8738	0	HOMO-3	LUMO	0.16484	π-π*
			HOMO-2	LUMO+1	0.33298	π-π*
			HOMO	LUMO+2	0.20474	π-π*
			HOMO	LUMO+4	0.14244	π-π*
S3	4.1810	0.0252	HOMO-1	LUMO	0.11225	π-π*
			HOMO	LUMO+2	0.64761	π-π*
S4	4.2910	0.2164	HOMO-3	LUMO	0.26440	π-π*
			HOMO-1	LUMO	0.51231	π-π*
			HOMO	LUMO+1	0.28262	π-π*
			HOMO	LUMO+3	0.15014	π-π*
S5	4.4618	0.2468	HOMO-2	LUMO	0.56394	π-π*
			HOMO-1	LUMO	0.12768	π-π*
			HOMO	LUMO+1	0.30687	π-π*
S6	4.5208	0.1244	HOMO-3	LUMO	0.56619	π-π*
			HOMO	LUMO+2	0.21521	π-π*

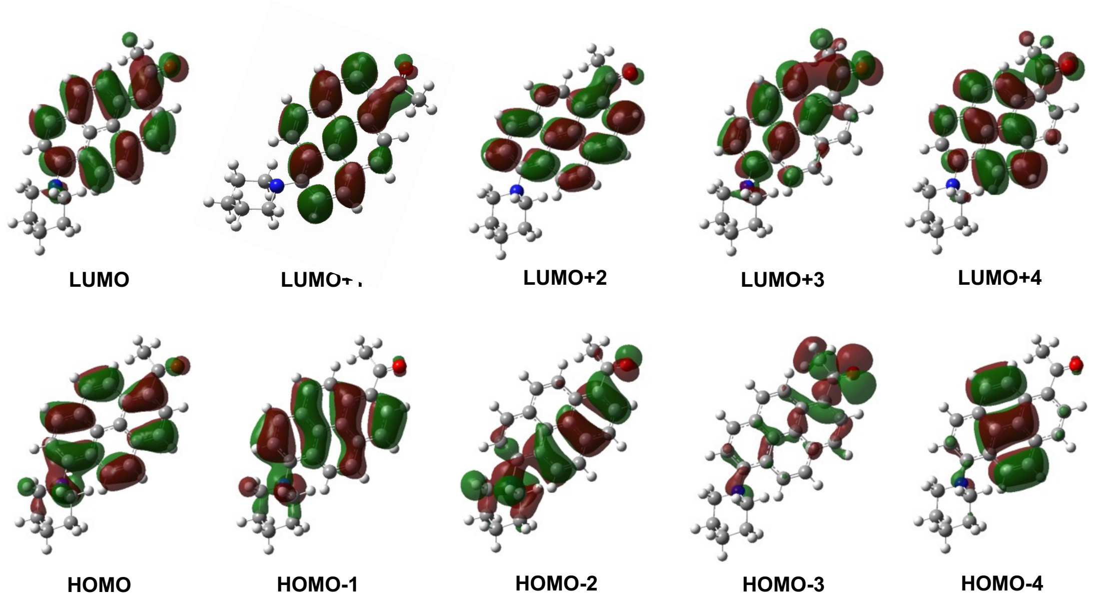

Figure S102. The Molecular orbitals of **PK** calculated by DFT (ωB97XD/6-311+G(d,p)).

Table S22. Excitation energy, oscillator strength, main transient orbital, attributed transition for **PK** calculated using TD-DFT(ωB97XD/6-311+G(d,p)).

State	Excitation energy [eV]	Oscillator strength	Main transition orbital		Contribution	transition
T1	1.29	0	HOMO-4	LUMO+2	0.10843	n-π*
			HOMO-1	LUMO	0.10496	π-π*
			HOMO	LUMO	0.69859	π-π*
			HOMO	LUMO	0.21972	π-π*
S1	2.69	0.87	HOMO	LUMO	0.69514	π-π*
T2	3.10	0	HOMO-5	LUMO	0.18485	π-π*
			HOMO-1	LUMO	0.42685	π-π*
			HOMO	LUMO+1	0.41079	π-π*
T3	3.26	0	HOMO-4	LUMO	0.15206	n-π*
			HOMO-3	LUMO	0.45177	π-π*
			HOMO-3	LUMO+1	0.13975	π-π*
			HOMO	LUMO+2	0.17017	π-π*
T4	3.27	0	HOMO-3	LUMO	0.33199	π-π*
			HOMO-3	LUMO+1	0.11062	π-π*
			HOMO-2	LUMO	0.20481	π-π*
			HOMO	LUMO+1	0.27902	π-π*
			HOMO	LUMO+3	0.22191	π-π*
T5	3.32	0	HOMO-4	LUMO	0.29885	n-π*
			HOMO-1	LUMO+1	0.11619	π-π*
			HOMO	LUMO+1	0.39173	π-π*
			HOMO	LUMO+2	0.31509	π-π*
			HOMO	LUMO+3	0.11129	π-π*
T6	3.43	0	HOMO-4	LUMO	0.19689	n-π*
			HOMO-2	LUMO	0.4431	π-π*
			HOMO-1	LUMO	0.36626	π-π*
			HOMO	LUMO+2	0.20476	π-π*
			HOMO	LUMO+3	0.16823	π-π*
S2	3.57	0.01	HOMO-2	LUMO	0.19698	π-π*
			HOMO-1	LUMO	0.37952	π-π*
			HOMO	LUMO+1	0.53346	π-π*
S3	3.68	0.0007	HOMO-3	LUMO	0.58868	π-π*
			HOMO-3	LUMO+1	0.16367	π-π*
T7	3.82	0	HOMO-4	LUMO	0.13843	n-π*
			HOMO-4	LUMO+2	0.17126	n-π*
			HOMO	LUMO+3	0.344	π-π*
S4	4.18	0.022	HOMO-4	LUMO	0.25302	n-π*
			HOMO	LUMO+2	0.62196	π-π*
S5	4.23	0.170	HOMO-4	LUMO	0.29829	n-π*
			HOMO-1	LUMO	0.48296	π-π*

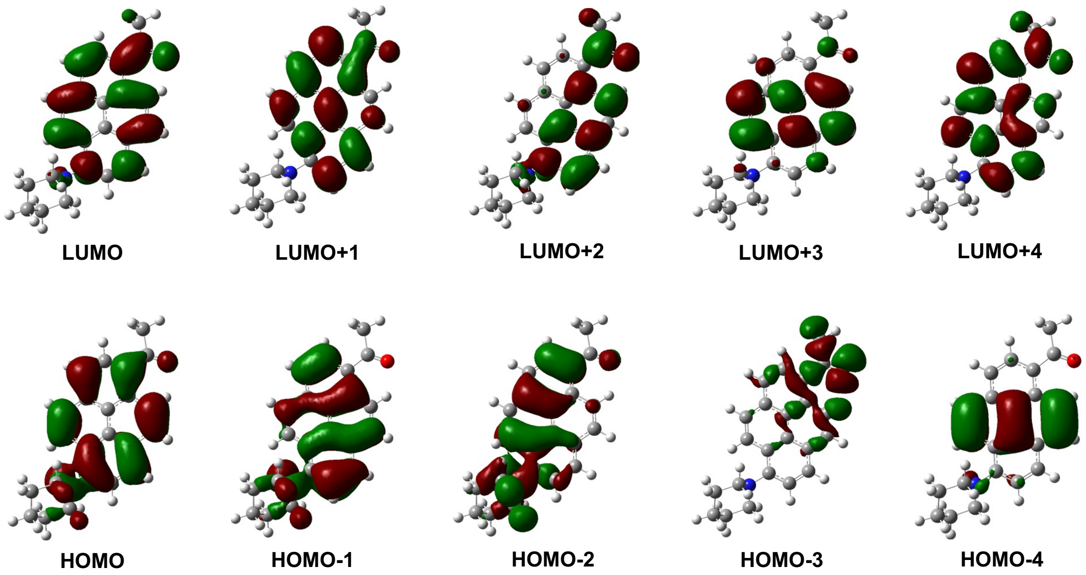

Figure S103. The Molecular orbitals of **PK** calculated by DFT (ωB97XD/6-31G(d,p)).

Table S23. Excitation energy, oscillator strength, main transient orbital, attributed transition for **PK** calculated using TD-DFT(ωB97XD/6-31G(d,p)).

State	Excitation energy [eV]	Oscillator strength	Main transition orbital		Contribution	transition
T1	1.29	0	HOMO-4	LUMO+2	0.10843	n-π*
			HOMO-1	LUMO	0.10496	π-π*
			HOMO	LUMO	0.69859	π-π*
			HOMO	LUMO	0.21972	π-π*
S1	2.69	0.87	HOMO	LUMO	0.69514	π-π*
T2	3.10	0	HOMO-5	LUMO	0.18485	π-π*
			HOMO-1	LUMO	0.42685	π-π*
			HOMO	LUMO+1	0.41079	π-π*
T3	3.26	0	HOMO-4	LUMO	0.15206	n-π*
			HOMO-3	LUMO	0.45177	π-π*
			HOMO-3	LUMO+1	0.13975	π-π*
			HOMO	LUMO+2	0.17017	π-π*
T4	3.27	0	HOMO-3	LUMO	0.33199	π-π*
			HOMO-3	LUMO+1	0.11062	π-π*
			HOMO-2	LUMO	0.20481	π-π*
			HOMO	LUMO+1	0.27902	π-π*
			HOMO	LUMO+3	0.22191	π-π*
T5	3.32	0	HOMO-4	LUMO	0.29885	n-π*
			HOMO-1	LUMO+1	0.11619	π-π*
			HOMO	LUMO+1	0.39173	π-π*
			HOMO	LUMO+2	0.31509	π-π*
			HOMO	LUMO+3	0.11129	π-π*

T6	3.43	0	HOMO-4	LUMO	0.19689	n-π*
			HOMO-2	LUMO	0.4431	π-π*
			HOMO-1	LUMO	0.36626	π-π*
			HOMO	LUMO+2	0.20476	π-π*
			HOMO	LUMO+3	0.16823	π-π*
S2	3.57	0.01	HOMO-2	LUMO	0.19698	π-π*
			HOMO-1	LUMO	0.37952	π-π*
			HOMO	LUMO+1	0.53346	π-π*
S3	3.68	0.0007	HOMO-3	LUMO	0.58868	π-π*
			HOMO-3	LUMO+1	0.16367	π-π*
T7	3.82	0	HOMO-4	LUMO	0.13843	n-π*
			HOMO-4	LUMO+2	0.17126	n-π*
			HOMO	LUMO+3	0.344	π-π*
S4	4.18	0.022	HOMO-4	LUMO	0.25302	n-π*
			HOMO	LUMO+2	0.62196	π-π*
S5	4.23	0.170	HOMO-4	LUMO	0.29829	n-π*
			HOMO-1	LUMO	0.48296	π-π*

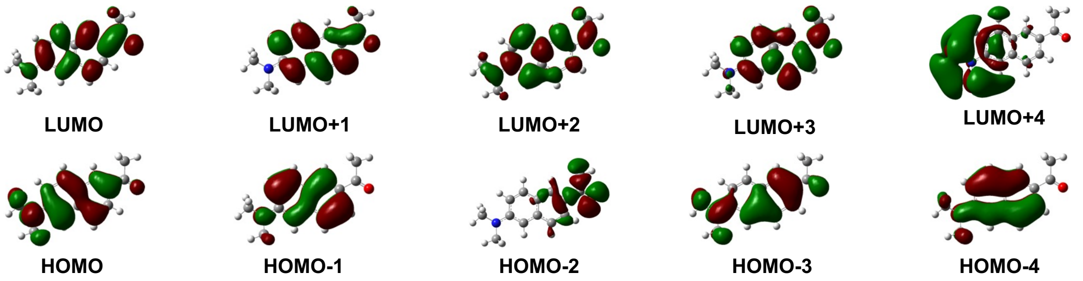

Figure S104. The Molecular orbitals of **Prodan** calculated by DFT (ωB97XD/6-31G(d,p)).

Table S24. Excitation energy, oscillator strength, main transient orbital, attributed transition for **Prodan** calculated using TD-DFT(ωB97XD/6-31G(d,p)).

State	Excitation energy [eV]	Oscillator strength	Main transition orbital		Contribution	transition
T1	2.3688	0	HOMO-1	LUMO	0.14182	π-π*
			HOMO	LUMO	0.58688	π-π*
T2	2.9153	0	HOMO-2	LUMO	0.62218	n-π*
			HOMO-2	LUMO+1	0.19083	n-π*
S1	3.4455	0.0001	HOMO-2	LUMO	0.63476	n-π*
			HOMO-2	LUMO+1	0.18555	n-π*
T3	3.4711	0	HOMO-5	LUMO	0.12081	π-π*
			HOMO-1	LUMO+1	0.31927	π-π*
			HOMO	LUMO	0.11630	π-π*
			HOMO	LUMO+1	0.50470	π-π*
T4	3.5659	0	HOMO-1	LUMO	0.51385	π-π*
			HOMO	LUMO+1	0.27838	π-π*

S2	3.8387	0.6541	HOMO	LUMO	0.67122	π-π^*
T5	3.9769	0	HOMO-3	LUMO	0.29132	π-π^*
			HOMO-1	LUMO	0.33173	π-π^*
			HOMO-1	LUMO+1	0.38413	π-π^*
			HOMO	LUMO	0.19248	π-π^*
			HOMO	LUMO+2	0.17752	π-π^*
T6	4.0914	0	HOMO-5	LUMO	0.16360	π-π^*
			HOMO-4	LUMO	0.23548	π-π^*
			HOMO	LUMO+1	0.35215	π-π^*
			HOMO	LUMO+2	0.29653	π-π^*
S3	4.1213	0.0109	HOMO-1	LUMO	0.46511	π-π^*
			HOMO	LUMO+1	0.49916	π-π^*
S4	4.8426	0.6226	HOMO-3	LUMO	0.12674	π-π^*
			HOMO-1	LUMO	0.49456	π-π^*
S5	5.4610	0.7340	HOMO-1	LUMO+1	0.64358	π-π^*
			HOMO	LUMO+2	0.10170	π-π^*
S6	5.7875	0.0535	HOMO-4	LUMO	0.12109	π-π^*
			HOMO-3	LUMO	0.56544	π-π^*

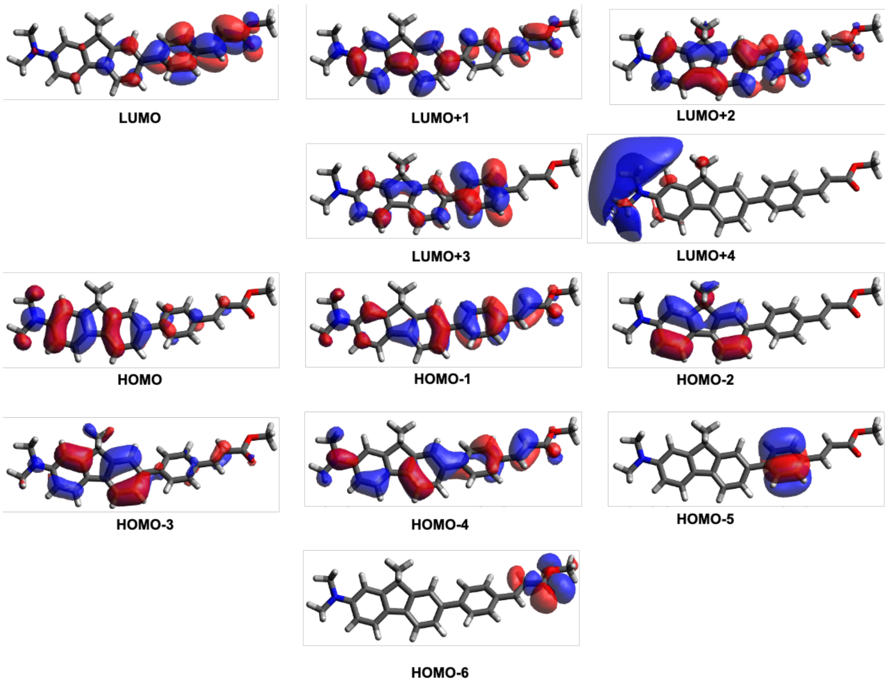

Figure S105. The Molecular orbitals of **FπCMst** calculated by DFT (B3LYP/6-311+G(d,p)).

Table S25. Excitation energy, oscillator strength, main transient orbital, attributed transition for **FπCMst** calculated using TD-DFT(B3LYP/6-311+G(d,p)).

State	Excitation energy [eV]	Oscillator strength	Main transition orbital		Contribution	transition
T1	1.96	0	HOMO	LUMO	0.53934	π-π*
T2	2.43	0	HOMO-4	LUMO	0.18917	π-π*
			HOMO-1	LUMO	0.46199	π-π*
			HOMO	LUMO	0.35031	π-π*
			HOMO	LUMO	0.28211	π-π*
S1	2.45	0.5205	HOMO	LUMO	0.70605	π-π*
T3	2.87	0	HOMO	LUMO+1	0.57618	π-π*
			HOMO	LUMO+7	0.10853	π-π*
T4	3.25	0	HOMO	LUMO+1	0.10928	π-π*
			HOMO	LUMO+2	0.6476	π-π*
T5	3.57	0	HOMO-4	LUMO	0.2966	π-π*
			HOMO-7	LUMO+8	0.10139	π-π*
S2	3.60	1.2578	HOMO-1	LUMO	0.5316	π-π*
T6	3.71	0	HOMO-5	LUMO	0.63395	π-π*
			HOMO-1	LUMO+3	0.16197	π-π*
S3	3.77	0.0215	HOMO-1	LUMO	0.2909	π-π*
			HOMO	LUMO+1	0.26283	π-π*
			HOMO	LUMO+2	0.55256	π-π*
S4	3.87	0.0326	HOMO-3	LUMO	0.10769	π-π*
			HOMO-1	LUMO	0.32019	π-π*
			HOMO	LUMO+1	0.43453	π-π*
S5	3.98	0.0060	HOMO-5	LUMO	0.2096	π-π*
			HOMO-1	LUMO	0.11968	π-π*
			HOMO	LUMO+1	0.13555	π-π*
			HOMO	LUMO+3	0.6383	π-π*
S6	4.06	0.0003	HOMO-6	LUMO	0.68753	n-π*

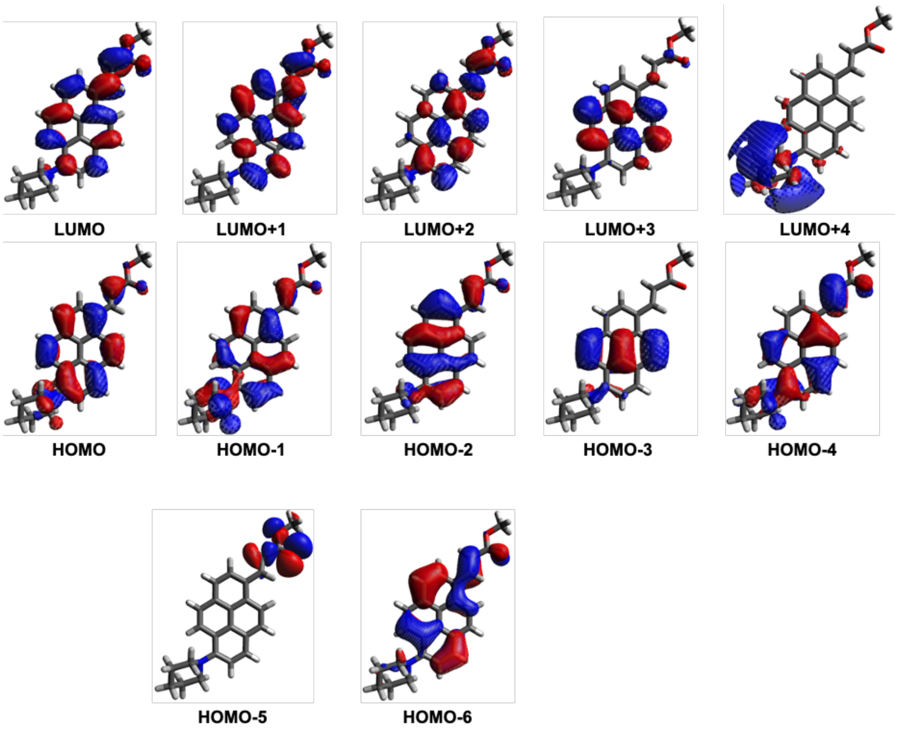

Figure S106. The Molecular orbitals of **PCMst** calculated by DFT (B3LYP/6-311+G(d,p)).

Table S26. Excitation energy, oscillator strength, main transient orbital, attributed transition for **PCMst** calculated using TD-DFT(B3LYP/6-311+G(d,p)).

State	Excitation energy [eV]	Oscillator strength	Main transition orbital		Contribution	transition
T1	1.43	0	HOMO	LUMO	0.67825	π-π*
S1	2.46	0.6198	HOMO	LUMO	0.69011	π-π*
T2	2.52	0	HOMO-5	LUMO	0.17265	π-π*
			HOMO-2	LUMO	0.24605	π-π*
			HOMO	LUMO+1	0.45582	π-π*
T3	2.85	0	HOMO-2	LUMO+1	0.12999	π-π*
			HOMO-1	LUMO	0.42368	π-π*
			HOMO	LUMO+1	0.47368	π-π*
			HOMO	LUMO+2	0.14058	π-π*
T4	3.07	0	HOMO-2	LUMO	0.5983	π-π*
			HOMO-1	LUMO	0.27714	π-π*
			HOMO-1	LUMO+1	0.11655	π-π*
T5	3.18	0	HOMO-2	LUMO	0.17894	π-π*
			HOMO	LUMO+2	0.59744	π-π*
S2	3.22	0.0259	HOMO	LUMO+1	0.55426	π-π*
			HOMO	LUMO+2	0.18367	π-π*
T6	3.29	0	HOMO-3	LUMO	0.35999	π-π*
			HOMO-3	LUMO+1	0.11594	π-π*
			HOMO	LUMO+2	0.19852	π-π*

			HOMO	LUMO+3	0.47749	π-π*
S3	3.52	0.3172	HOMO-1	LUMO	0.63022	π-π*
			HOMO	LUMO	0.12314	π-π*
			HOMO	LUMO+1	0.21413	π-π*
S4	3.71	0.0153	HOMO-5	LUMO	0.12719	π-π*
			HOMO-1	LUMO	0.22232	π-π*
			HOMO	LUMO+2	0.39675	π-π*
S5	3.83	0.0927	HOMO-1	LUMO+1	0.15501	π-π*
			HOMO	LUMO+3	0.49875	π-π*
S6	3.87	0.047	HOMO-2	LUMO	0.27031	π-π*
			HOMO-1	LUMO	0.10818	π-π*
			HOMO	LUMO+2	0.44747	π-π*
			HOMO	LUMO+3	0.41588	π-π*

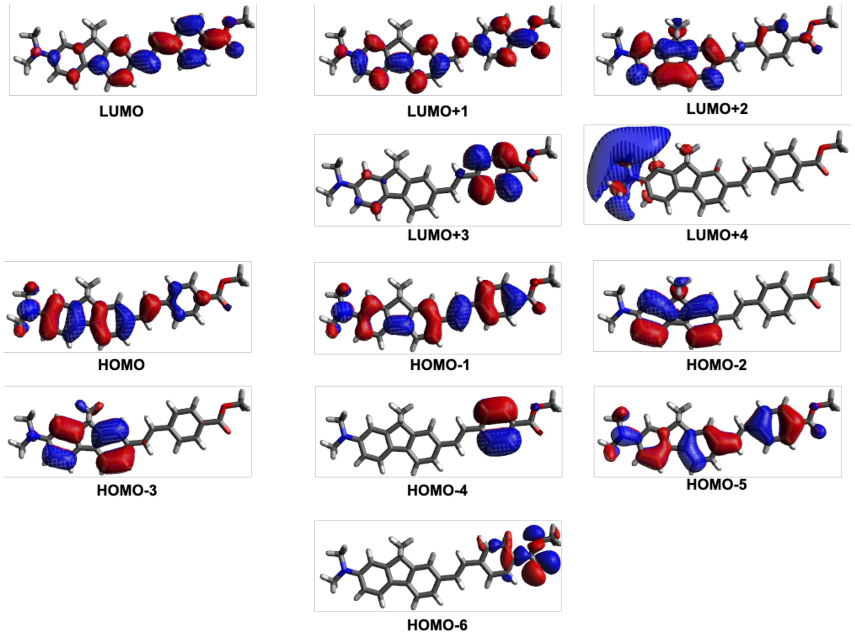

Figure S107. The Molecular orbitals of **FstCM** calculated by DFT (B3LYP/6-311+G(d,p)).

Table S27. Excitation energy, oscillator strength, main transient orbital, attributed transition for **FstCM** calculated using TD-DFT(B3LYP/6-311+G(d,p)).

State	Excitation energy [eV]	Oscillator strength	Main transition orbital		Contribution	transition
T1	1.64	0	HOMO	LUMO	0.53934	π-π*
S2	2.55	1.268	HOMO	LUMO	0.70587	π-π*
T2	2.58	0	HOMO-1	LUMO	0.54733	π-π*
			HOMO	LUMO	0.21957	π-π*
			HOMO	LUMO+7	0.12129	π-π*
T3	3.07	0	HOMO-1	LUMO	0.25601	π-π*

			HOMO	LUMO	0.24438	π-π*
			HOMO	LUMO+1	0.55083	π-π*
T4	3.26	0	HOMO	LUMO+2	0.64913	π-π*
S2	3.47	0.5488	HOMO-1	LUMO	0.6086	π-π*
			HOMO	LUMO+1	0.34885	π-π*
T5	3.5	0	HOMO-7	LUMO	0.19393	π-π*
			HOMO-4	LUMO+3	0.10881	π-π*
			HOMO-1	LUMO+1	0.35423	π-π*
			HOMO	LUMO+5	0.12296	π-π*
T6	3.69	0	HOMO-4	LUMO	0.26071	π-π*
			HOMO-2	LUMO	0.31281	π-π*
			HOMO	LUMO+5	0.36067	π-π*
			HOMO	LUMO+7	0.12709	π-π*
S3	3.71	0.024	HOMO-3	LUMO	0.11994	π-π*
			HOMO-1	LUMO	0.2761	π-π*
			HOMO	LUMO+2	0.44955	π-π*
S4	3.89	0.0426	HOMO-3	LUMO	0.13224	π-π*
			HOMO	LUMO+1	0.42247	π-π*
			HOMO	LUMO+2	0.49196	π-π*
S5	4.03	0.0173	HOMO-6	LUMO	0.27483	π-π*
			HOMO-2	LUMO	0.13564	π-π*
			HOMO	LUMO+3	0.60124	π-π*
S6	4.09	0	HOMO-2	LUMO	0.53859	π-π*
			HOMO	LUMO+2	0.1188	π-π*

Optimized Structures

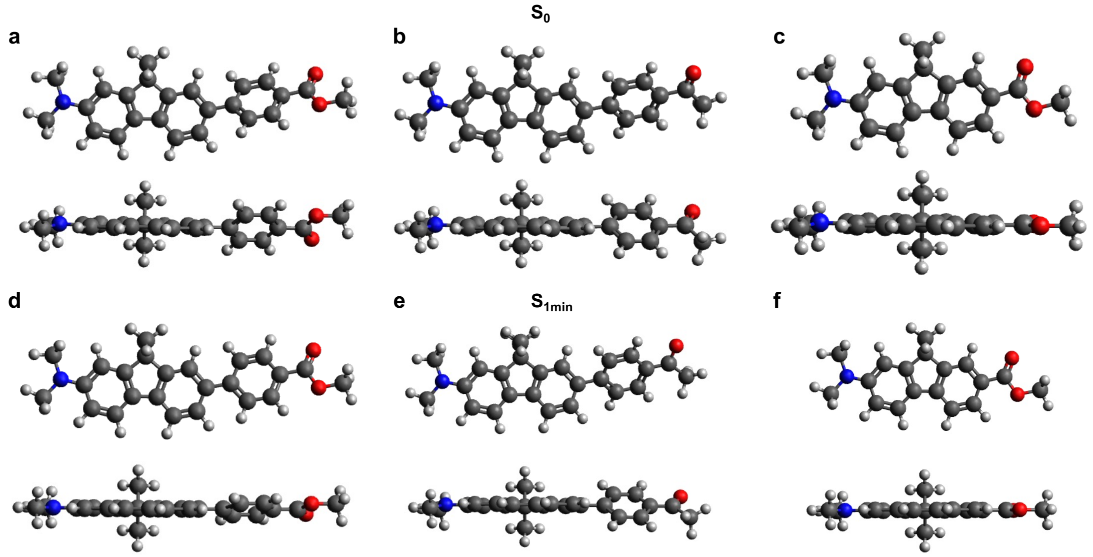

Figure S108. Optimized S_0 and S_{1min} structures. **a**, S_0 of **FπCM**, **b**, S_0 of **FπA, c,** S_0 of **FCM**, **d**, S_{1min} of **FπCM**, **e**, S_{1min} of **FπA, f,** S_{1min} of **FCM** calculated by DFT (ωB97XD/6-31G(d,p).

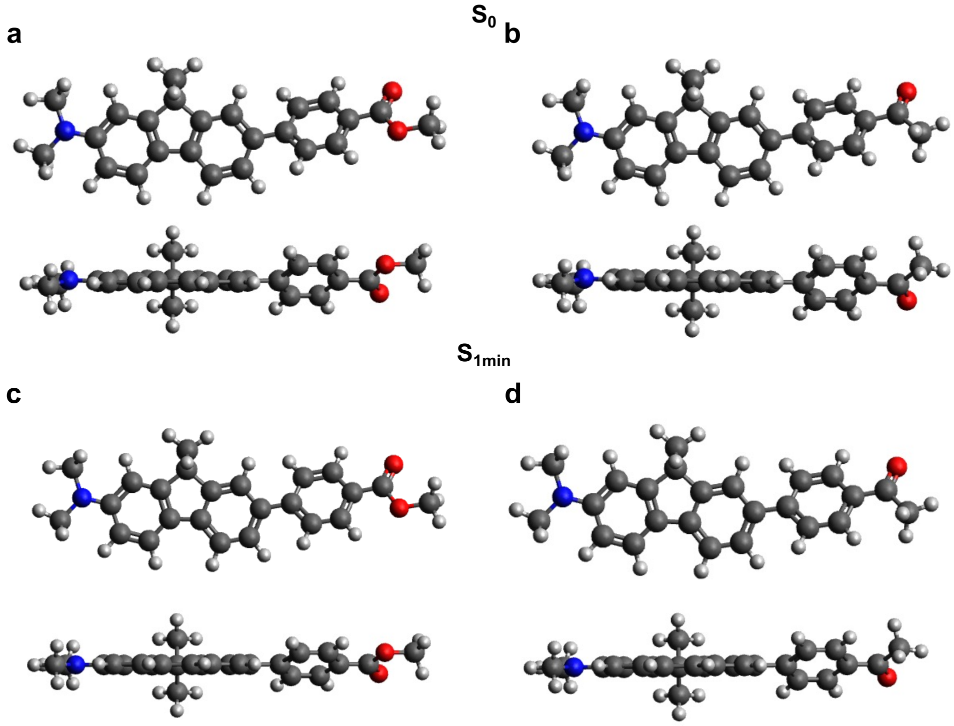

Figure S109. Optimized S_0 and S_{1min} structures. **a**, S_0 of **FπCM**, **b**, S_0 of **FπA**, **c**, S_{1min} of **FπCM**, **d**, S_{1min} of **FπA** calculated by DFT (B3LYP/6-311+G(d,p).

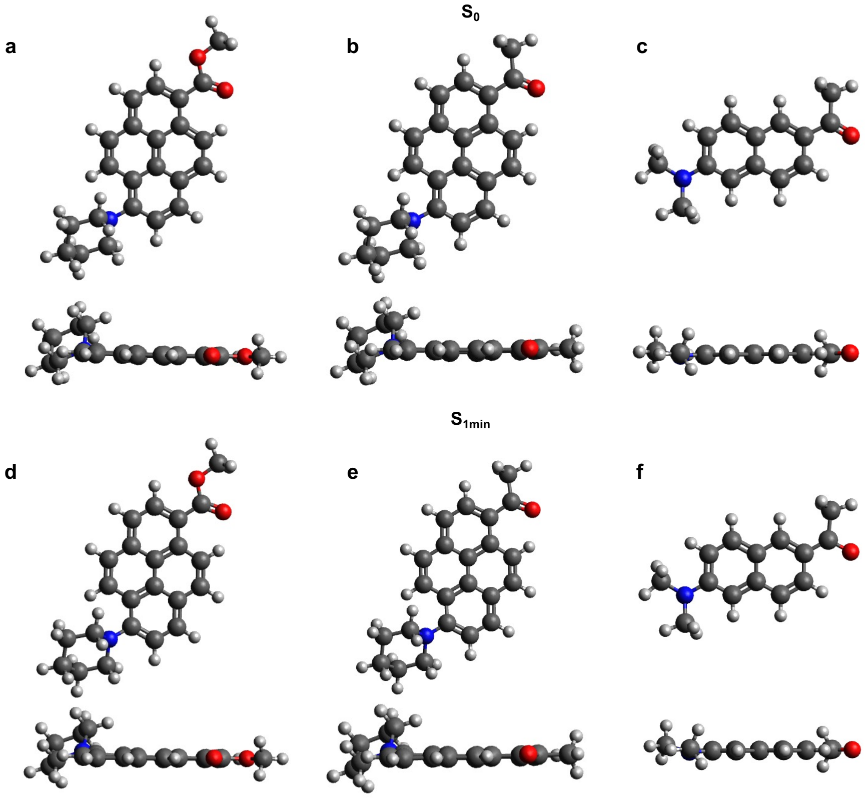

Figure S110. Optimized S_0 and S_{1min} structures. **a**, S_0 of **PCM**, **b**, S_0 of **PK**, **c**, S_0 of **Prodan**, **d**, S_{1min} of **PCM**, **e,** S_{1min} of **PK** and **f**, S_{1min} of **Prodan** calculated by DFT (ωB97XD/6-31G(d,p).

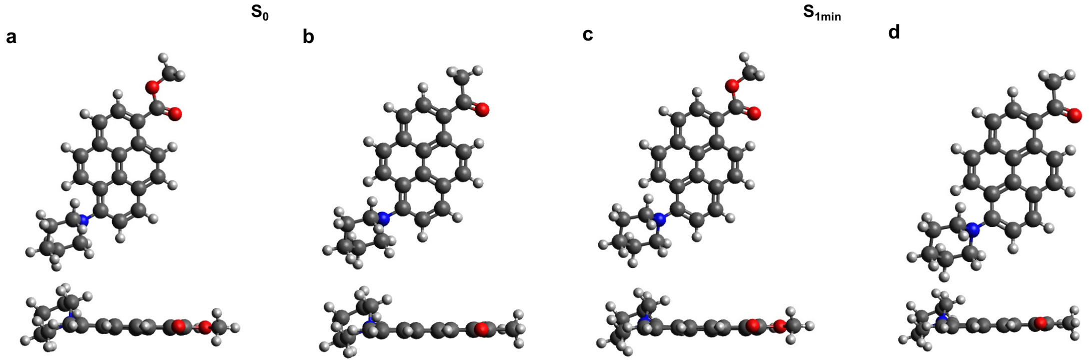

Figure S111. Optimized S_0 and S_{1min} structures. a, S_0 of **PCM**, **b**, S_0 of **PK**, **c**, S_{1min} of **PCM** and **d**, S_{1min} **of PK** calculated by DFT (ωB97XD/6-311+G(d,p).

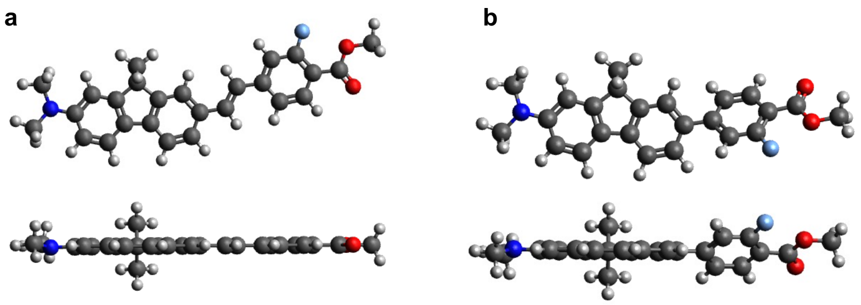

Figure S112. Optimized S_0 structures of **a**, **FπCM*o*-F** and **b**, **FstCM*o*-F** calculated by DFT (ωB97XD/6-311+G(d,p).

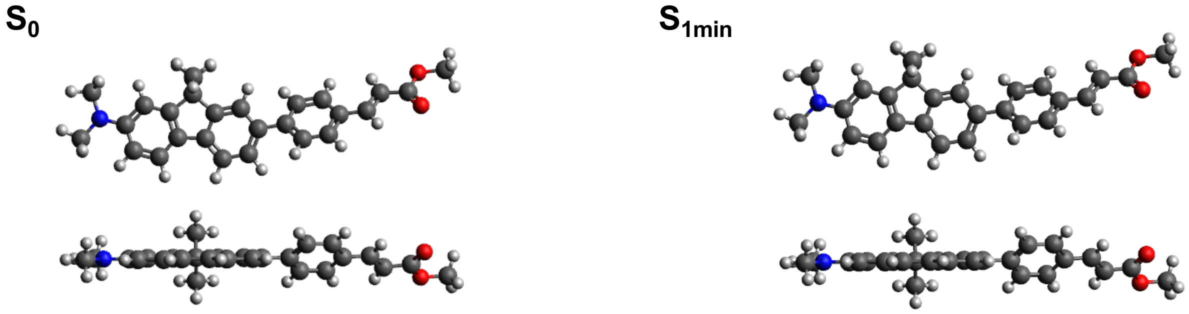

Figure S113. Optimized S_0 and S_{1min} structures of **FπCMst** calculated by DFT (B3LYP/6-311+G(d,p).

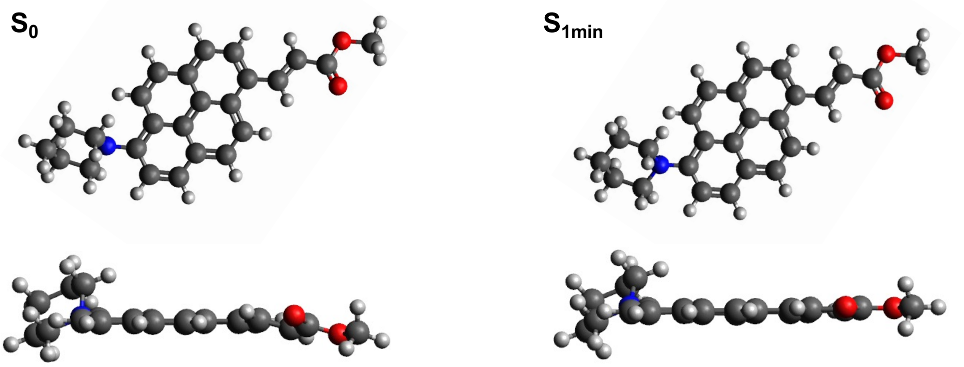

Figure S114. Optimized S_0 and S_{1min} structures of **PCMst** calculated by DFT (B3LYP/6-311+G(d,p).

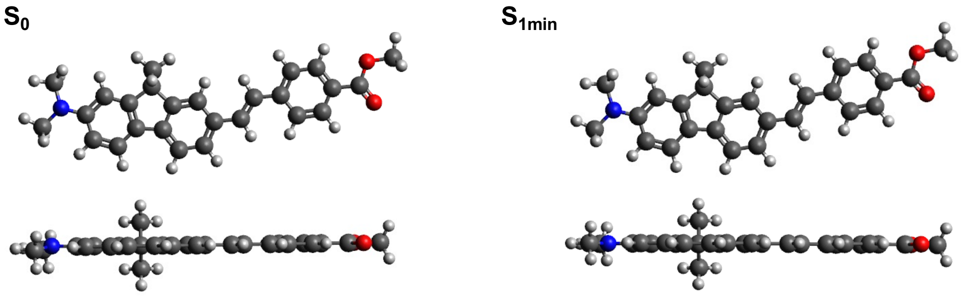

Figure S115. Optimized S_0 and S_{1min} structures of **FstCM** calculated by DFT (B3LYP/6-311+G(d,p).

S5-2. Summary of theoretical calculations for monomer

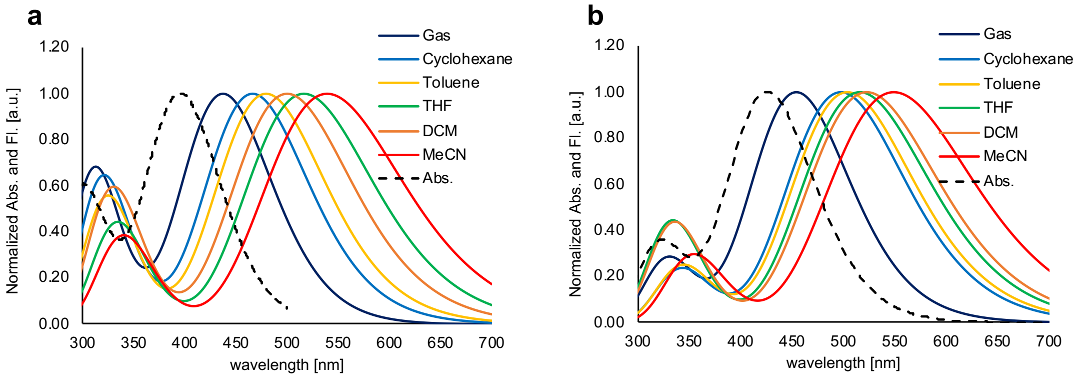

Figure S116. Calculated absorption in gas and fluorescence in gas, cyclohexane, Toluene, THF, dichloromethane (DCM) and acetonitrile (MeCN) spectra of **a**, **FπCM** and **b**, **FπA**. TD-DFT calculation level at B3LYP/6-311+G(d,p) with SMD solvation method.

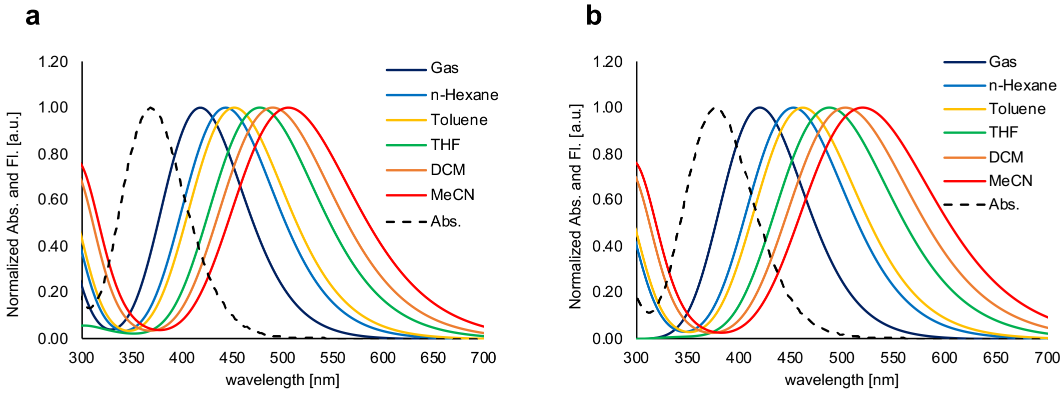

Figure S117. Calculated absorption in gas and fluorescence in gas, cyclohexane, Toluene, THF, dichloromethane (DCM) and acetonitrile (MeCN) spectra of **a**, **PCM** and **b**, **PK**. TD-DFT calculation level at ωB97XD/6-311+G(d,p) with SMD solvation method.

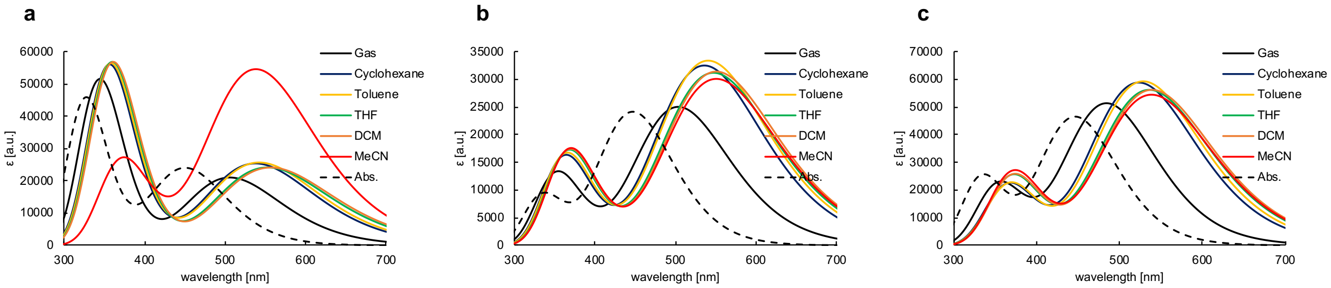

Figure S118. Calculated absorption in gas and fluorescence in gas, cyclohexane, Toluene, THF, dichloromethane (DCM) and acetonitrile (MeCN) spectra of **a**, **FπCMst**, **b**, **PCMst**, and **c**, **FstCM**. TD-DFT calculation level at ωB97XD/6-311+G(d,p) with SMD solvation method.

Table S28. Calculated wavalength and Osicillator strength.

		B3LYP/6-311+G(d,p)										wB97XD/6-311+G(d,p)			
	State	**FπCM**	Osc.	**FπA**	Osc.	**FπCMst**	Osc.	**PCMst**	Osc.	**FstCM**	Osc.	**PCM**	Osc.	**PK**	Osc.
λ_{abs}	Gas	395	0.6707	426	0.5267	450	0.5935	447	0.5950	447	1.1448	368	0.7430	377	0.7343
λ_{fl}	Gas	437	0.8352	454	0.6266	505	0.5198	503	0.6196	485	1.2675	428	0.6369	420	0.6118
	Hexane	466[a]	0.9840[a]	498[a]	1.0203[a]	538	0.6267	535	0.8031	523	1.4491	443[b]	0.8236[b]	453[b]	0.8137[b]
	Toluene	479	1.1041	504	1.0505	542	0.6357	540	0.8218	529	1.4624	452	0.8816	462	0.8735
	THF	516	1.3331	533	1.188	553	0.5954	547	0.7708	536	1.3877	477	1.0625	488	1.0598
	DCM	500	1.1427	523	1.355	557	0.5976	551	0.7773	539	1.3914	490	1.1032	503	1.1008
	MeCN	538	1.4449	549	1.2522	538	1.3451	550	0.7402	539	1.3451	506	1.1803	519	1.1809

a, cyclohexane was used for **FπCM** and **FπA**. b, *n*-hexane was used for **PCM** and **PK**.

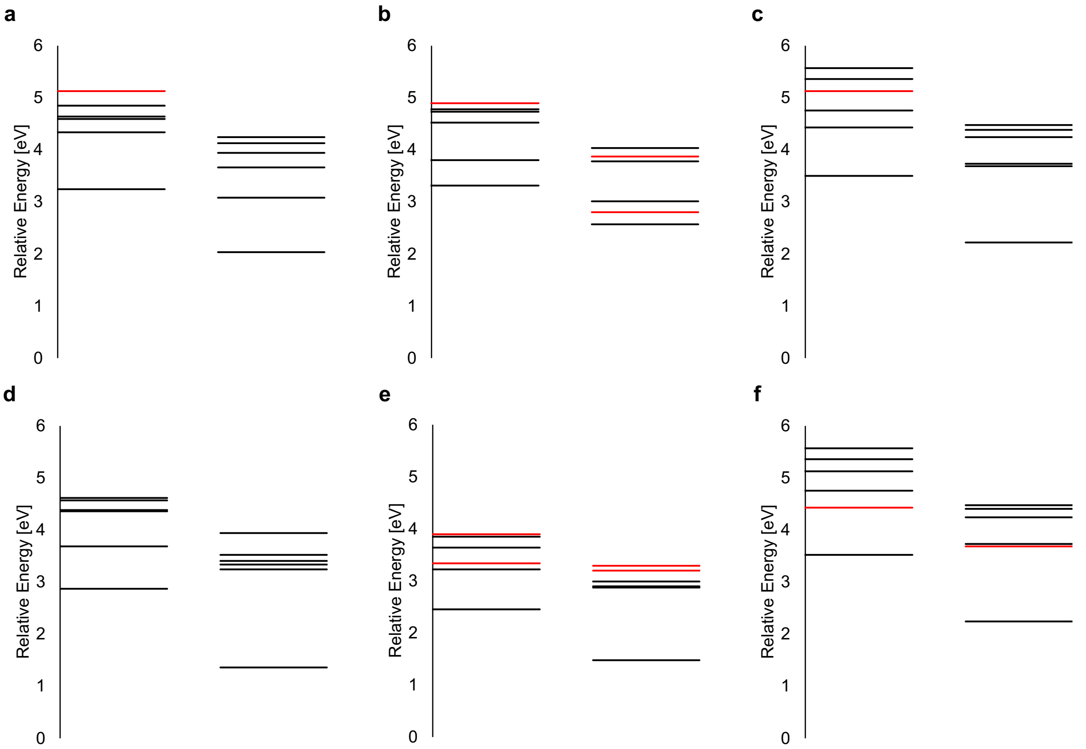

Figure S119. Simulated singlet and triplet energy diagram of **a**, **FπCM**, **b**, **FπA**, **c**, **FCM**, **d**, **PCM**, **e**, **PK** and **f**, **Prodan** by TD-DFT（ωB97XD/6-31G(d,p)）(black line: π-π*, red line: n-π* transition).

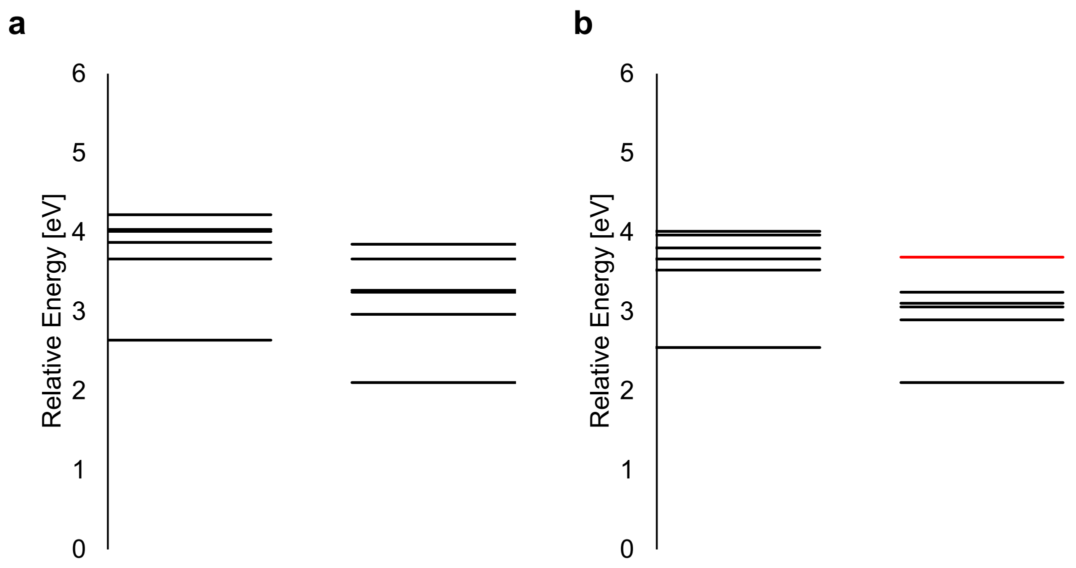

Figure S120. Simulated singlet and triplet energy diagram of **FπCM** and **FπA** by TD-DFT (B3LYP/6-311+G(d,p)) (black line: π-π*, red line: n-π* transition).

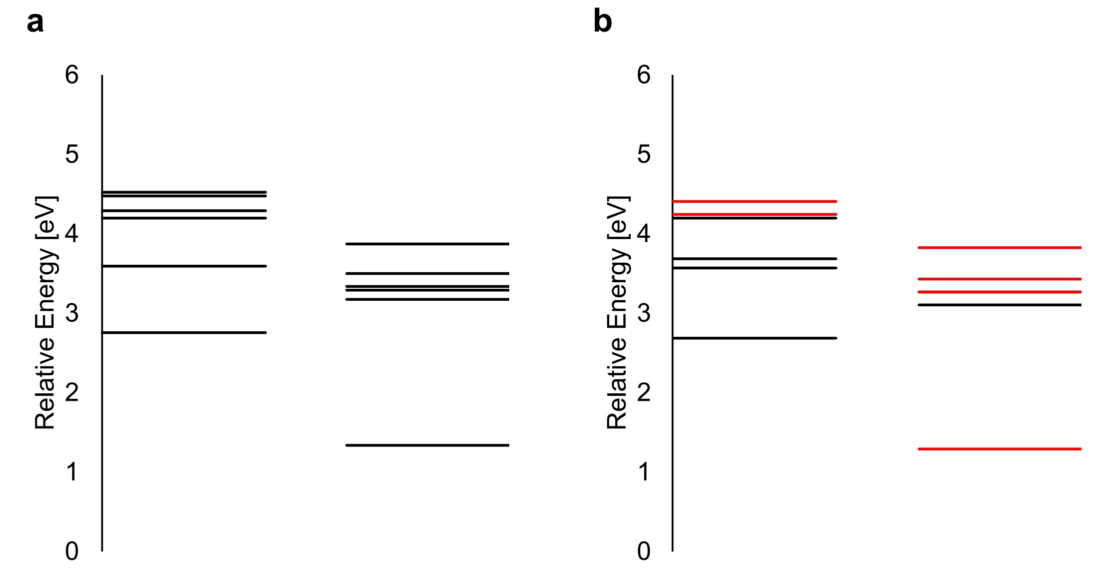

Figure S121. Simulated singlet and triplet energy diagram of **PCM** and **PK** by TD-DFT(ωB97XD/6-311G(d,p)) (black line: π-π*, red line: n-π* transition).

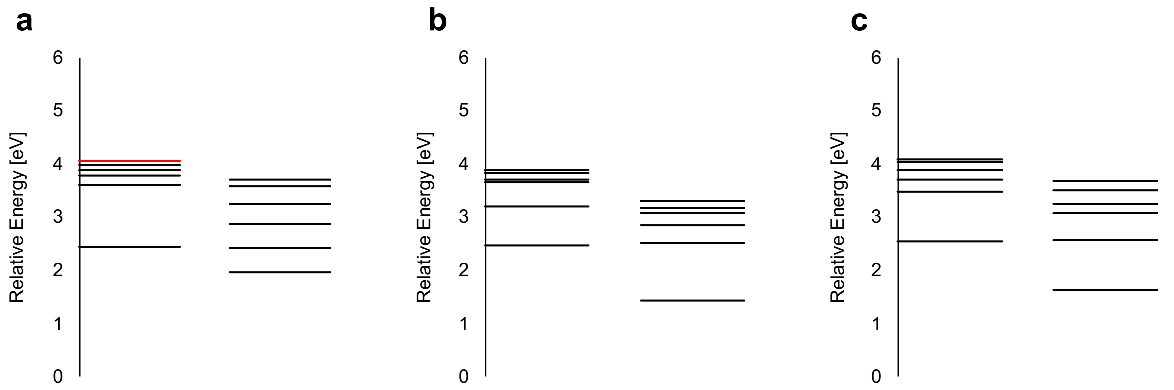

Figure S122. Simulated singlet and triplet energy diagram of **FπCMst**, **PCMst** and **FstCM** by TD-DFT(B3LYP/6-311+G(d,p)) (black line: π-π*, red line: n-π* transition).

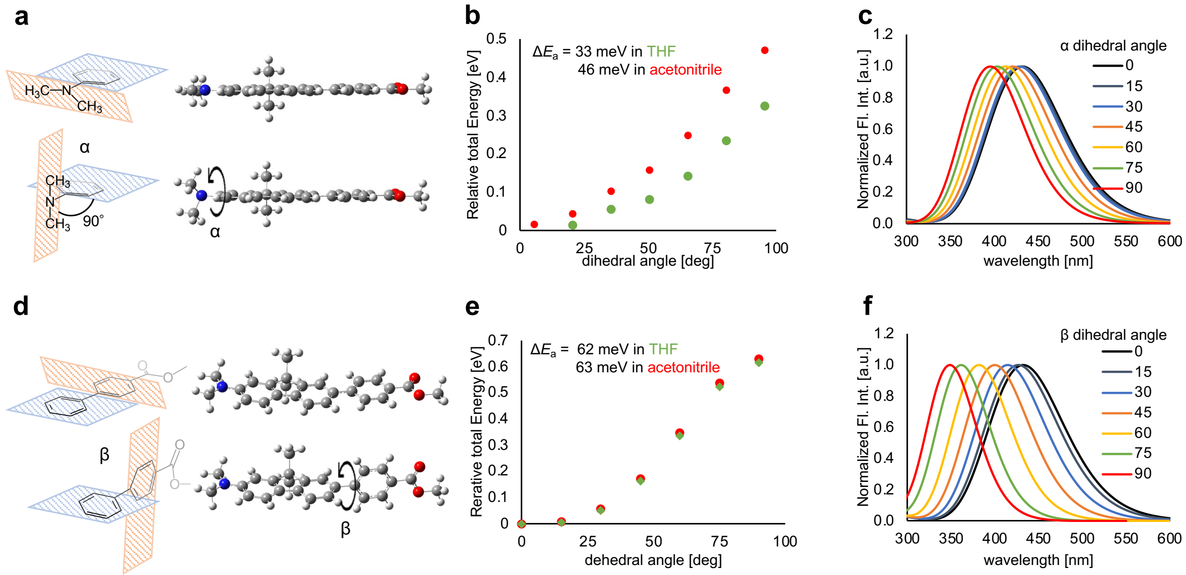

Figure S123. **a**, The optimized structure of relaxed α scan for **FπCM.** **b**, Potential energy surface relaxed α-constrained scans of **FπCM** in THF and acetonitrile with polarizable continuum model solvation. The activation energy ΔE_a to TICT (90 deg in dihedral) were 33 meV in THF and 46 meV in acetonitrile, respectively. **c**, Calculated fluorescence spectra dependent on the dihedral angle of α. **d**, The optimized structure of relaxed β scan for **FπCM.** **e**, Potential energy surface relaxed β-constrained scans of **FπCM** in THF and acetonitrile with polarizable continuum model solvation. The activation energy ΔE_a to 90 deg in dihedral were 62 meV in THF and 63 meV in acetonitrile, respectively. **f**, Calculated fluorescence spectra dependent on the dihedral angle of β.

S5-3. Intermolecular interaction energies and transition probabilities for dimers

The gaussian 09, 16 program were used for ab initio molecular orbital calculations of dimers within the single crystal structures of **FπCM**, **FπA**, **PCM, FπCM*o*-F** and **FstCM*o*-F**. The dimer of each compound was calculated from a combination of intermolecular interactions estimated from the packing pattern obtained from the single crystal X-ray structure analysis. (**Figures S90-S94**) Only one pattern of dimer was observed for **FπCM**, **FπA, FπCM*o*-F** and **PCM** in a unit. Intermolecular interaction calculations were performed at the ωB97XD/6-311G(d,p) level. The basis set superposition error was corrected by the counterpoise method.[S7]

Intermolecular interaction energy calculations of molecular interactions within a crystal were conducted using Crystal Explorer. [S18] The calculation was performed at the HF/3-21G level of theory. In Crystal Explorer, intermolecular interaction calculations are performed for monomers in element notation and each color monomer as a dimer. The the total intermolecular energy (E_{tot}) of dimer is calculated using the following equation S5:

$$E_{\text{tot}} = k_{\text{ele}}E_{\text{ele}} + k_{\text{pol}}E_{\text{pol}} + k_{\text{dis}}E_{\text{dis}} + k_{\text{rep}}E_{\text{rep}} \quad (\text{eq. S5})$$

Here, E_{ele} represents the electrostatic interactions, E_{pol} stands for the induction energy from permanent and induced dipoles, E_{dis} corresponds to the London dispersion forces between induced dipoles, and E_{rep} represents the repulsive term associated with exchange repulsion. The constants k_{ele}, k_{pol}, k_{dis}, and k_{rep} in the equation depend on the specific calculation conditions. In the case of HF/3-21G, the value of k_{ele} is 1.019, $k_{pol,}$ 0.651, $k_{dis,}$0.901 and $k_{rep,}$0.811.

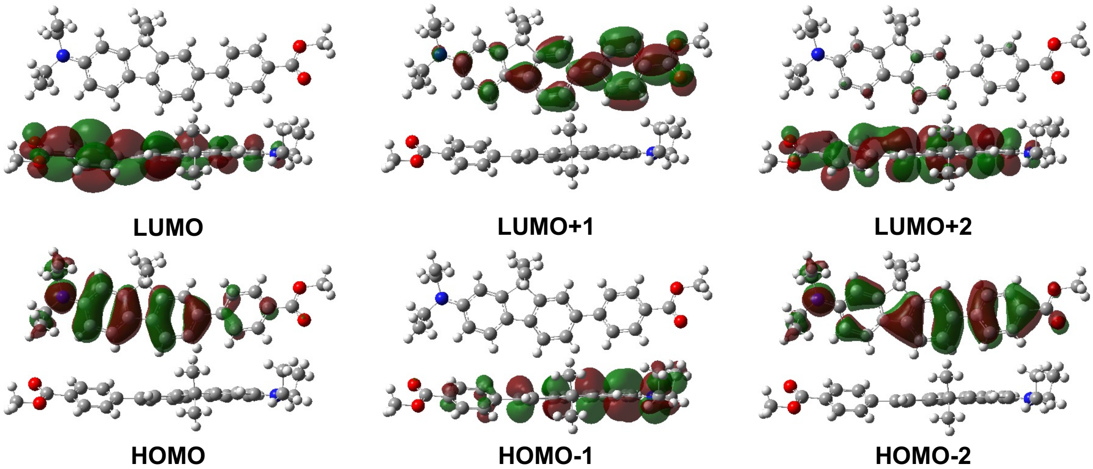

Figure S124. The molecular orbitals of **FπCM** dimer calculated by ωB97XD/6-311G(d,p) using counterpoise method. The intermolecular interaction energy was -72.8 kJ/mol.

Table S29. Energy/wavelength of S_0-S_n, oscillator strength, main transient orbital, Contribution for **FπCM** calculated using TD-DFT(ωB97XD/6-311G(d,p)).

State	energy [eV]/wavelength [nm]	Oscillator strength	Main transition orbital		Contribution
S_0-S_1	3.93/315	1.3605	HOMO	LUMO	0.596
			HOMO	LUMO+2	0.158
S_0-S_2	4.33/286	0.0273	HOMO-3	LUMO	0.109
			HOMO	LUMO	0.102
			HOMO	LUMO+1	0.441
			HOMO	LUMO+2	0.185
			HOMO	LUMO+3	0.403
S_0-S_3	4.77/260	0.0118	HOMO-3	LUMO	0.186
			HOMO-2	LUMO+1	0.103
			HOMO	LUMO+1	0.170
			HOMO	LUMO+2	0.149
			HOMO	LUMO+4	0.452
S_0-S_4	4.99/248	0.0015	HOMO-5	LUMO+2	0.131
			HOMO-4	LUMO	0.489
			HOMO-4	LUMO+1	0.123
			HOMO-1	LUMO+3	0.226
			HOMO	LUMO+2	0.178
S_0-S_5	5.03/246	0.0407	HOMO-1	LUMO	0.442
			HOMO	LUMO+2	0.313
			HOMO	LUMO+3	0.231
			HOMO	LUMO+5	0.136
S_0-S_6	5.27/235	0.0003	HOMO-6	LUMO	0.554
			HOMO-6	LUMO+1	0.226
			HOMO-6	LUMO+5	0.138
			HOMO-6	LUMO+6	0.129

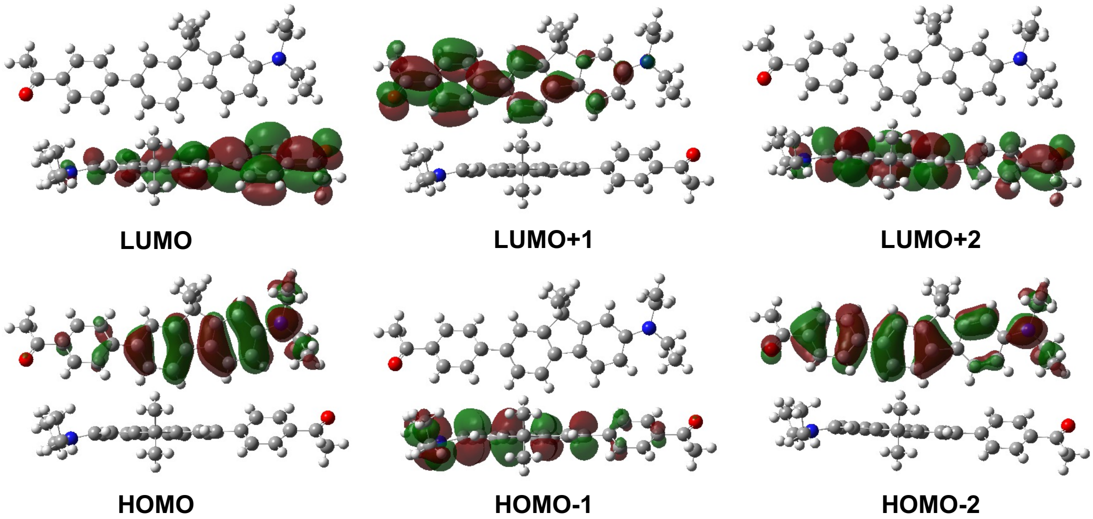

Figure S125. The molecular orbitals of **FπA** dimer calculated by ωB97XD/6-311G(d,p) using counterpoise method. The intermolecular interaction energy was -66.5 kJ/mol.

Table S30. Energy/wavelength of S_0-S_n oscillator strength, main transient orbital, Contribution for **FπA** calculated using TD-DFT (ωB97XD /6-311G(d,p)).

State	energy [eV]/wavelength [nm]	Oscillator strength	Main transition orbital		Contribution
S_0-S_1	3.98/311	1.2915	HOMO	LUMO	0.54975
S_0-S_2	4.04/307	0	HOMO-4	LUMO	0.56524
			HOMO-4	LUMO+1	0.28640
			HOMO-4	LUMO+2	0.13093
			HOMO-4	LUMO+5	0.17893
S_0-S_3	4.37/284	0.0546	HOMO-1	LUMO+4	0.10911
			HOMO	LUMO+2	0.38909
			HOMO	LUMO+4	0.26409
S_0-S_4	4.83/257	0.0097	HOMO-3	LUMO	0.17586
			HOMO-2	LUMO+1	0.17621
			HOMO-1	LUMO+2	0.17214
			HOMO	LUMO+1	0.11036
			HOMO	LUMO+4	0.47258
S_0-S_5	4.96/250	0.0269	HOMO-2	LUMO	0.39932
			HOMO-1	LUMO+4	0.20623
			HOMO	LUMO+5	0.16998
S_0-S_6	4.99/249	0.0042	HOMO-5	LUMO	0.46890
			HOMO-5	LUMO+1	0.12836
			HOMO-1	LUMO	0.14909
			HOMO-1	LUMO+1	0.18634
			HOMO-1	LUMO+3	0.22779

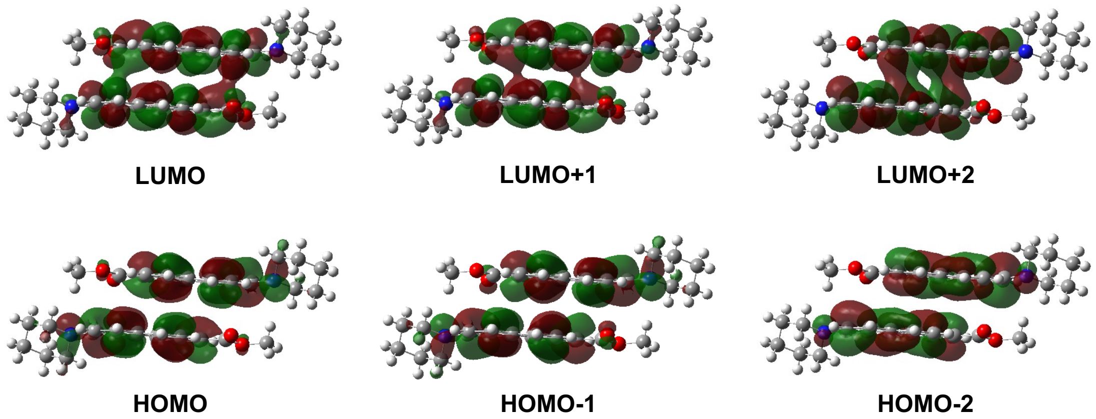

Figure S126. The molecular orbitals of **PCM** dimer calculated by ωB97XD/6-311G(d,p) using counterpoise method. The intermolecular interaction energy was -87.0 kJ/mol.

Table S31. Energy of S_0-S_n/wavelength, oscillator strength, main transient orbital, Contribution for **PCM** calculated using TD-DFT(ωB97XD/6-311G(d,p)).

State	energy [eV]/wavelength [nm]	Oscillator strength	Main transition orbital		Contribution
S_0-S_1	2.77/447	0.0004	HOMO	LUMO	0.706
S_0-S_2	2.85/435	0.0046	HOMO-1	LUMO	0.473
			HOMO-1	LUMO+1	0.319
S_0-S_3	2.89/429	0.0396	HOMO-1	LUMO	0.473
			HOMO-1	LUMO+1	0.319
S_0-S_4	2.98/416	1.4645	HOMO-1	LUMO	0.469
			HOMO	LUMO+1	0.525
S_0-S_5	3.68/337	0.0108	HOMO-1	LUMO+2	0.552
			HOMO	LUMO+2	0.230
S_0-S_6	3.69/336	0.0496	HOMO-1	LUMO+2	0.393
			HOMO	LUMO+3	0.574

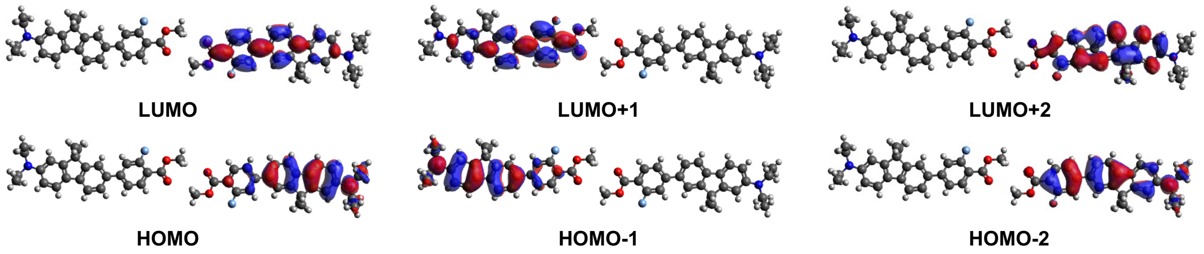

Figure S127. The molecular orbitals of **FπCM*o*-F** dimer 1 calculated by ωB97XD/6-311G(d,p) using counterpoise method. The intermolecular interaction energy was -17.5 kJ/mol.

Table S32. Energy of S_0-S_n/wavelength, oscillator strength, main transient orbital, Contribution for **FπCM*o*-F** dimer 1 calculated using TD-DFT(ωB97XD/6-311G(d,p)).

State	energy [eV]/wavelength [nm]	Oscillator strength	Main transition orbital		Contribution
S_0-S_1	3.83/324	1.3900	HOMO	LUMO	0.61501
			HOMO	LUMO+2	0.18976
S_0-S_2	4.35/285	0.0137	HOMO-3	LUMO	0.12900
			HOMO	LUMO+1	0.50367
			HOMO	LUMO+4	0.11620
S_0-S_3	4.71/263	0.0086	HOMO-4	LUMO	0.10937
			HOMO-1	LUMO+1	0.22995
			HOMO-1	LUMO+2	0.11319
			HOMO	LUMO+4	0.31903
S_0-S_4	4.89/253	0.0138	HOMO-4	LUMO	0.45892
			HOMO-1	LUMO+2	0.11802
			HOMO-1	LUMO+3	0.12490
			HOMO	LUMO+1	0.10805
S_0-S_5	5.05/246	0.0381	HOMO-4	LUMO	0.27082
			HOMO-1	LUMO	0.38114
			HOMO	LUMO+2	0.29448
S_0-S_6	5.37/231	0.0005	HOMO-6	LUMO	0.55204
			HOMO-6	LUMO+1	0.16271
			HOMO-6	LUMO+3	0.13015
			HOMO-6	LUMO	0.12123

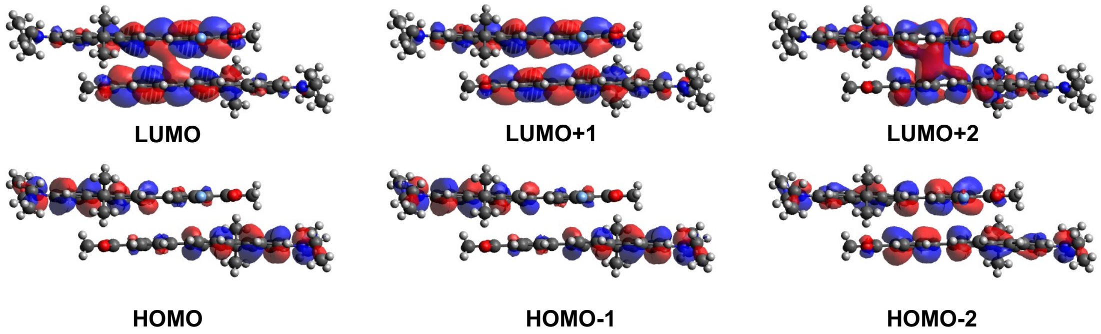

Figure S128. The molecular orbitals of **FπCM*o*-F** dimer 2 calculated by ωB97XD/6-311G(d,p) using counterpoise method. The intermolecular interaction energy was -89.1 kJ/mol.

Table S33. Energy of S_0-S_n/wavelength, oscillator strength, main transient orbital, Contribution for **FπCM*o*-F** dimer 2 calculated using TD-DFT(ωB97XD/6-311G(d,p)).

State	energy [eV]/wavelength [nm]	Oscillator strength	Main transition orbital		Contribution
S_0-S_1	3.78/328	1.4233	HOMO	LUMO	0.61789
			HOMO	LUMO+1	0.12387
			HOMO	LUMO+2	0.19188
S_0-S_2	4.31/287	0.0174	HOMO	LUMO+1	0.50642
S_0-S_3	4.67/265	0.0087	HOMO-3	LUMO	0.23638
			HOMO-2	LUMO	0.28577
			HOMO-2	LUMO+2	0.10324
			HOMO-1	LUMO	0.11654
			HOMO-1	LUMO+1	0.24230
			HOMO	LUMO+2	0.22700
			HOMO	LUMO+4	0.28128
S_0-S_4	4.90/253	0.0097	HOMO-4	LUMO	0.45466
			HOMO-1	LUMO	0.27123
			HOMO-1	LUMO+3	0.10120
			HOMO-1	LUMO+4	0.12721
			HOMO	LUMO+1	0.10020
			HOMO	LUMO+2	0.17944
			HOMO	LUMO+3	0.11795
S_0-S_5	5.03/246	0.0332	HOMO-1	LUMO	0.38799
			HOMO	LUMO+2	0.27868
			HOMO	LUMO+3	0.21075
			HOMO	LUMO+4	0.12155
			HOMO	LUMO+5	0.14689
S_0-S_6	5.38/230	0.0003	HOMO-6	LUMO	0.55206
			HOMO-6	LUMO+5	0.20634

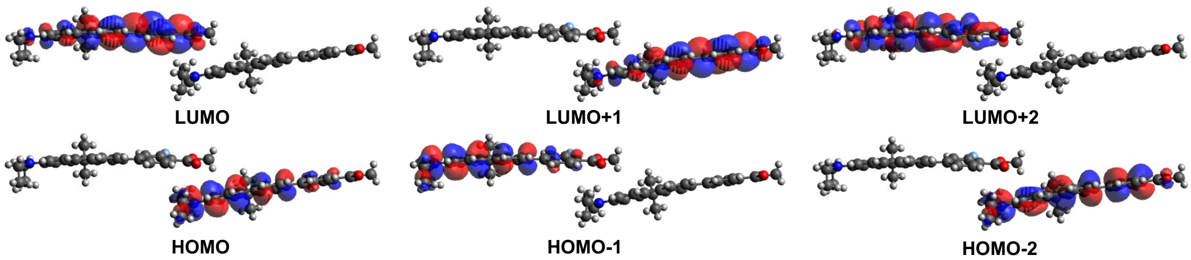

Figure S129. The molecular orbitals of **FπCM*o*-F** dimer 3 calculated by ωB97XD/6-311G(d,p) using counterpoise method. The intermolecular interaction energy was -30.7 kJ/mol.

Table S34. Energy of S_0-S_n/wavelength, oscillator strength, main transient orbital, Contribution for **FπCM*o*-F** dimer 3 calculated using TD-DFT(ωB97XD/6-311G(d,p)).

State	energy [eV]/wavelength [nm]	Oscillator strength	Main transition orbital		Contribution
S_0-S_1	3.78/328	1.4232	HOMO	LUMO	0.61784
			HOMO	LUMO+2	0.19203
S_0-S_2	4.31/287	0.0174	HOMO-3	LUMO	0.12686
			HOMO	LUMO+1	0.50640
S_0-S_3	4.67/265	0.0087	HOMO-3	LUMO	0.23644
			HOMO-2	LUMO	0.28561
			HOMO-2	LUMO+2	0.10324
			HOMO-1	LUMO	0.11663
			HOMO	LUMO+1	0.18326
			HOMO	LUMO+2	0.22698
			HOMO	LUMO+3	0.24135
S_0-S_4	4.90/253	0.0097	HOMO-4	LUMO	0.45487
			HOMO	LUMO+2	0.11064
			HOMO	LUMO+3	0.10125
			HOMO	LUMO+4	0.12722
			HOMO	LUMO+1	0.10011
			HOMO	LUMO+3	0.11786
S_0-S_5	5.03/246	0.0332	HOMO-4	LUMO	0.27872
			HOMO-1	LUMO	0.38814
			HOMO	LUMO+2	0.27878
S_0-S_6	5.38/230	0.0003	HOMO-6	LUMO	0.55211
			HOMO-6	LUMO+1	0.13775
			HOMO-6	LUMO+3	0.14372

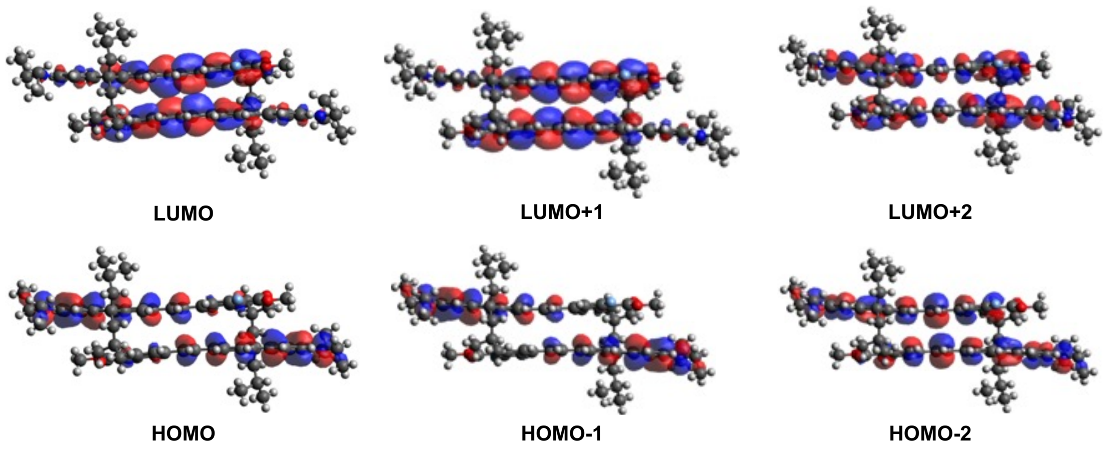

Figure S130. The molecular orbitals of **FstCM*o*-F** dimer 1 calculated by ωB97XD/6-311G(d,p) using counterpoise method. The intermolecular interaction energy was -60.9 kJ/mol.

Table S35. Energy of S_0-S_n/wavelength, oscillator strength, main transient orbital, Contribution for **FstCM*o*-F** dimer 1 calculated using TD-DFT(ωB97XD/6-311G(d,p)).

State	energy [eV]/wavelength [nm]	Oscillator strength	Main transition orbital		Contribution
S_0-S_1	3.52/352	1.7335	HOMO	LUMO	0.60283
S_0-S_2	4.27/290	0.0145	HOMO-1	LUMO	0.11317
			HOMO	LUMO+2	0.54534
S_0-S_3	4.61/269	0.0735	HOMO-4	LUMO	0.16742
			HOMO-1	LUMO	0.49395
			HOMO	LUMO+5	0.15383
S_0-S_4	4.66/266	0.0051	HOMO-3	LUMO+1	0.10391
			HOMO-2	LUMO+1	0.16889
			HOMO-1	LUMO+1	0.11426
			HOMO	LUMO+4	0.46064
S_0-S_5	4.81/258	0.0225	HOMO-5	LUMO	0.22633
			HOMO-5	LUMO+3	0.15125
			HOMO-4	LUMO	0.47290
			HOMO-4	LUMO+1	0.18557
			HOMO	LUMO+3	0.19808
S_0-S_6	5.23/237	0.1072	HOMO-1	LUMO	0.28928
			HOMO	LUMO	0.29147
			HOMO	LUMO+1	0.40186
			HOMO	LUMO+2	0.16478

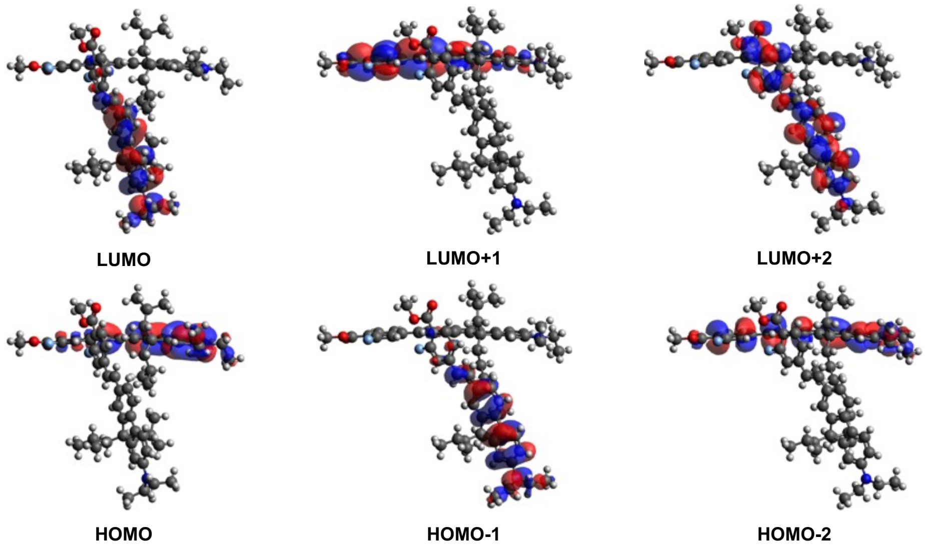

Figure S131. The molecular orbitals of **FstCM*o*-F** dimer 2 calculated by ωB97XD/6-311G(d,p) using counterpoise method. The intermolecular interaction energy was -57.4 kcal/mol.

Table S36. Energy of S_0-S_n/wavelength, oscillator strength, main transient orbital, Contribution for **FstCM*o*-F** dimer 2 calculated using TD-DFT(ωB97XD/6-311G(d,p)).

State	energy [eV]/wavelength [nm]	Oscillator strength	Main transition orbital		Contribution
S_0-S_1	3.52/352	1.7335	HOMO	LUMO	0.60283
S_0-S_2	4.27/290	0.0145	HOMO-1	LUMO	0.11307
			HOMO	LUMO+2	0.54537
S_0-S_3	4.61/269	0.0736	HOMO-4	LUMO	0.16734
			HOMO-1	LUMO	0.49391
			HOMO	LUMO+5	0.15386
S_0-S_4	4.66/266	0.0051	HOMO-3	LUMO+1	0.10393
			HOMO-2	LUMO+1	0.16888
			HOMO-1	LUMO+1	0.11418
			HOMO	LUMO+4	0.46057
S_0-S_5	4.81/258	0.0225	HOMO-5	LUMO	0.22646
			HOMO-5	LUMO+3	0.15122
			HOMO-4	LUMO	0.47287
			HOMO-4	LUMO+1	0.18553
			HOMO	LUMO+3	0.19806
S_0-S_6	5.23/237	0.1072	HOMO-1	LUO	0.28930
			HOMO	LUMO	0.29150
			HOMO	LUMO+1	0.40181
			HOMO	LUMO+2	0.16482

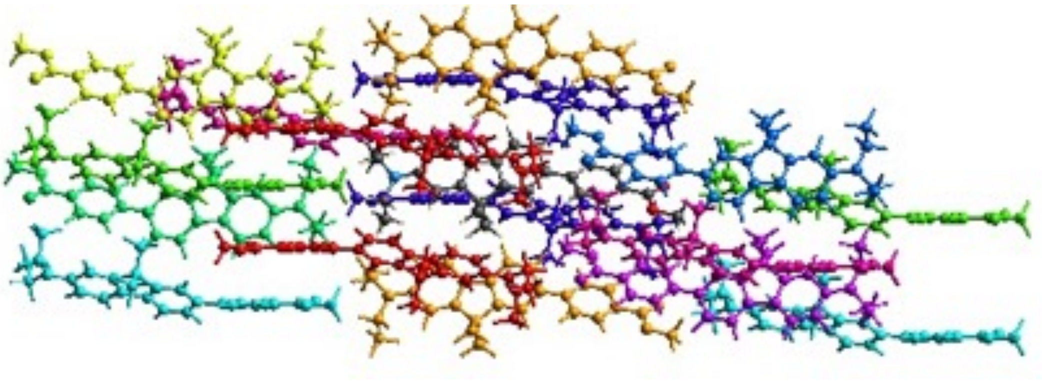

Figure S132. Intermolecular interaction energy calculation for **FπCM** crystal structure.

Table S37. Each Intermolecular interaction energy for **FπCM** crystal.

Color	N	*R*	E_{ele} [kJ/mol]	E_{pol} [kJ/mol]	E_{dis} [kJ/mol]	E_{rep} [kJ/mol]	E_{tot} [kJ/mol]
Red	2	8.36	-4.90	-3.00	-51.1	22.1	-35.1
Orange	2	7.04	-2.50	-1.30	-25.3	8.80	-19.0
Yellow	1	18.9	1.30	-0.40	-8.50	2.50	-4.50
Green	2	18.7	-10.8	0	-13.3	10.5	-14.5
Light Green	1	18.9	0.60	-6.10	-12.6	3.80	-11.6
Aqua	2	20.0	0.10	-0.10	-0.90	0	-0.80
Light Blue	1	20.0	0.10	-1.60	-0.90	0	-1.80
Deep Blue	2	5.55	-24.8	-2.70	-74.0	36.9	-63.8
Purple	1	13.4	-1.40	-4.90	-38.5	20.3	-22.9
Magenta	2	14.2	-1.10	-8.10	-14.9	6.50	-14.5

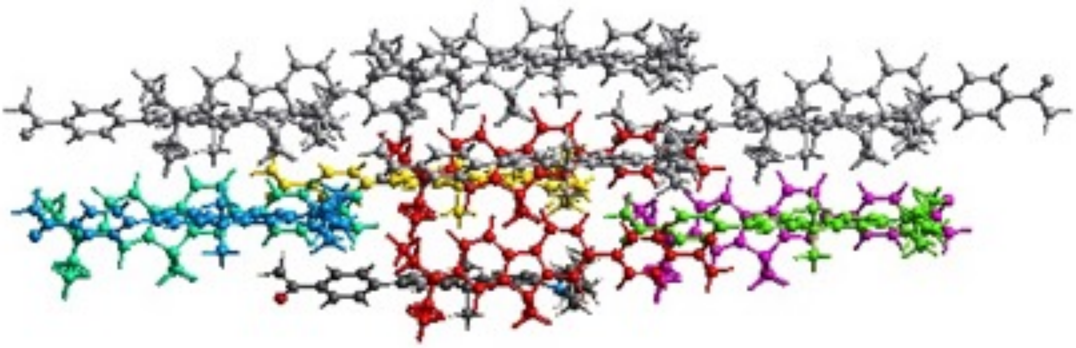

Figure S133. Intermolecular interaction energy calculation for **FπA** crystal structure.

Table S38. Each Intermolecular interaction energy for **FπA** crystal.

Color	N	*R*	E_{ele} [kJ/mol]	E_{pol} [kJ/mol]	E_{dis} [kJ/mol]	E_{rep} [kJ/mol]	E_{tot} [kJ/mol]
Red	2	6.79	-7.20	-8.70	-83.0	41.1	-54.5
Orange	1	7.06	-1.40	-5.50	-42.3	27.6	-20.7
Green	1	18.6	4.60	-10.4	-18.7	49.7	21.4
Light Green	1	15.6	-17.7	-4.10	-46.1	29.6	-38.3
Light Blue	1	20.0	-0.80	-2.50	-26.2	17.0	-12.2
Deep Blue	1	14.3	-17.7	-10.5	-85.8	35.2	-73.6
Pink	1	16.5	-6.20	-2.10	-30.2	27.9	-12.3

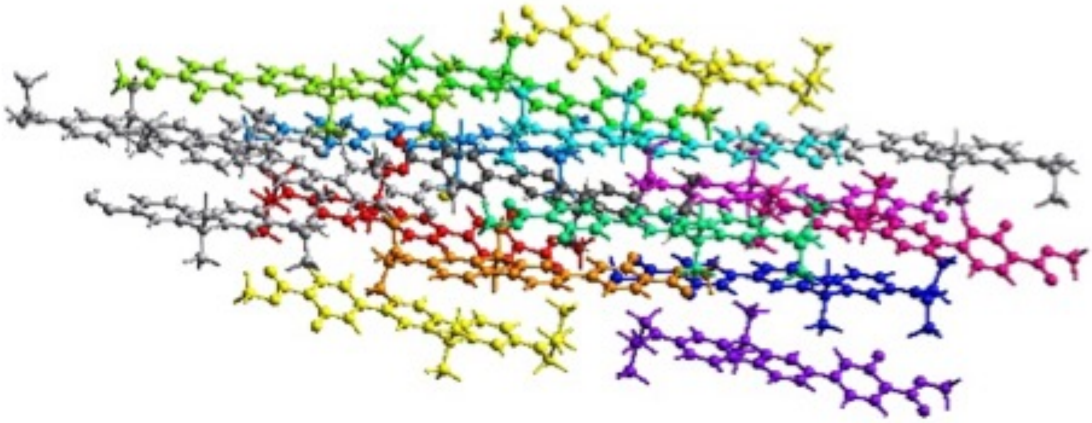

Figure S134. Intermolecular interaction energy calculation for **FπCMo-F** crystal structure.

Table S39. Each Intermolecular interaction energy for **FπCMo-F** crystal.

Color	N	*R*	E_{ele} [kJ/mol]	E_{pol} [kJ/mol]	E_{dis} [kJ/mol]	E_{rep} [kJ/mol]	E_{tot} [kJ/mol]
Red	1	7.24	-13.6	-6.1	-93.2	46.1	-64.4
Orange	1	6.64	-0.40	0	-0.26	0	-0.64
Yellow	2	10.1	0.30	-0.20	-7.50	1.10	-5.70
Green	1	14.7	-1.80	-0.90	-5.90	0.50	-7.30
Light Green	1	6.88	-15.0	-4.00	-41.7	15.0	-43.4
Aqua	1	9.58	-0.40	0	-0.30	0	-0.70
Light Blue	1	7.12	0.30	-0.20	-7.50	1.10	-5.70
Deep Blue	1	9.55	-0.20	-0.20	-3.70	0	-3.60
Purple	1	14.2	-0.20	-0.20	-3.70	0	-3.60
Magenta	1	15.5	-0.10	0	-0.20	0	-0.20
Pink	1	15.5	2.10	-0.80	-14.6	3.40	-8.80

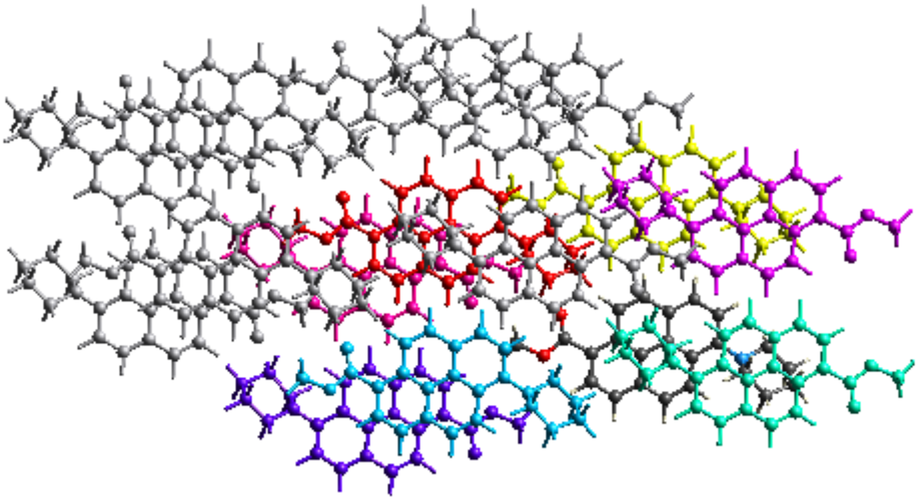

Figure S135. Intermolecular interaction energy calculation for **PCM** crystal structure.

Table S40. Each Intermolecular interaction energy for **PCM** crystal.

Color	N	*R*	E_{ele} [kJ/mol]	E_{pol} [kJ/mol]	E_{dis} [kJ/mol]	E_{rep} [kJ/mol]	E_{tot} [kJ/mol]
Red	1	12.2	-2.6	0	-24.2	13.7	-13.4
Yellow	1	7.76	-7.90	0	-30.1	15.98	-22.2
Green	1	5.34	-10.0	-9.50	-109	55.6	-69.0
Aqua	1	6.33	-17.1	-4.7	-77.1	34.0	-62.5
Magenta	1	7.95	-7.20	-1.90	-28.0	12.4	-23.8
Pink	1	15.1	-12.9	-3.3	-10.0	4.4	-20.8

S6. Biological optical imaging

Supplementary Video 1. Confocal fluorescence moving image of living cells stained with **FπCM** under continuous light irradiation. Morphology of chromosomes were simultaneously observed with mCherry-conjugated H2B. Cells were incubated with 1 μM dyes for 10 min. 405 nm laser, 5%; 561 nm laser, 1%. Bars, 50 μm.

Supplementary Video 2. Confocal fluorescence moving image of living cells stained with **PK** under continuous light irradiation. Morphology of chromosomes were simultaneously observed with mCherry-conjugated H2B. Cells were incubated with 1 μM dyes for 10 min. 405 nm laser, 5%; 561 nm laser, 1%. Bars, 50 μm.

Supplementary Video 3. Ratiometric movie images of HeLa cells expressing Histone 2B-mCherry stained with **FπCM-SO_3** displayed in the same way as **a**. 405 nm laser, 1%. 561 nm laser, 1%. Bars, 10 mm.

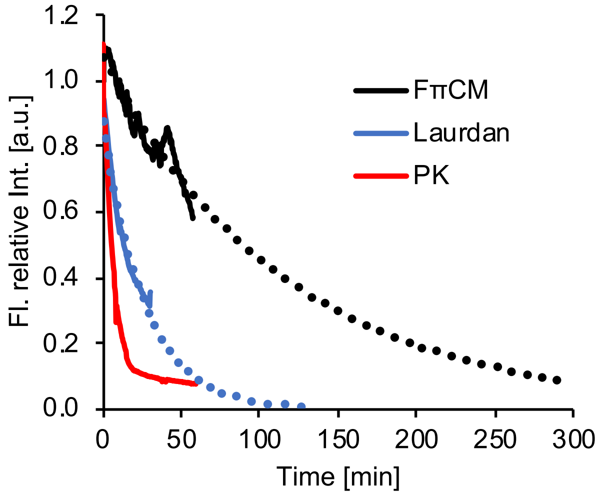

Figure S136. Photodegradation curves (solid line) of **FπCM**, **PK**, and **Laurdan** as a function of time in cell membranes under continuous light irradiation and the exponential approximation curve (dashed line) from each decay curve. it was predicted that PK and Laurdan would be completely quenched in about 30 min, whereas FπCM would endure for about 5 h.

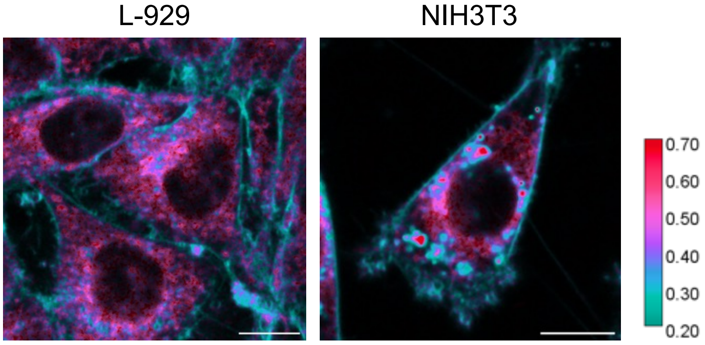

Figure S137. Confocal fluorescence images of L-929 and NIH/3T3 cells stained with 1 μM FπCM for 10 min. Images were obtained with excitation at 405 nm and with detection of 'blue' channel and 'red' channel at 400-500 nm and 500-700 nm, respectively. Images are displayed as Red/Blue Ratio (color) × Intensity (brightness).

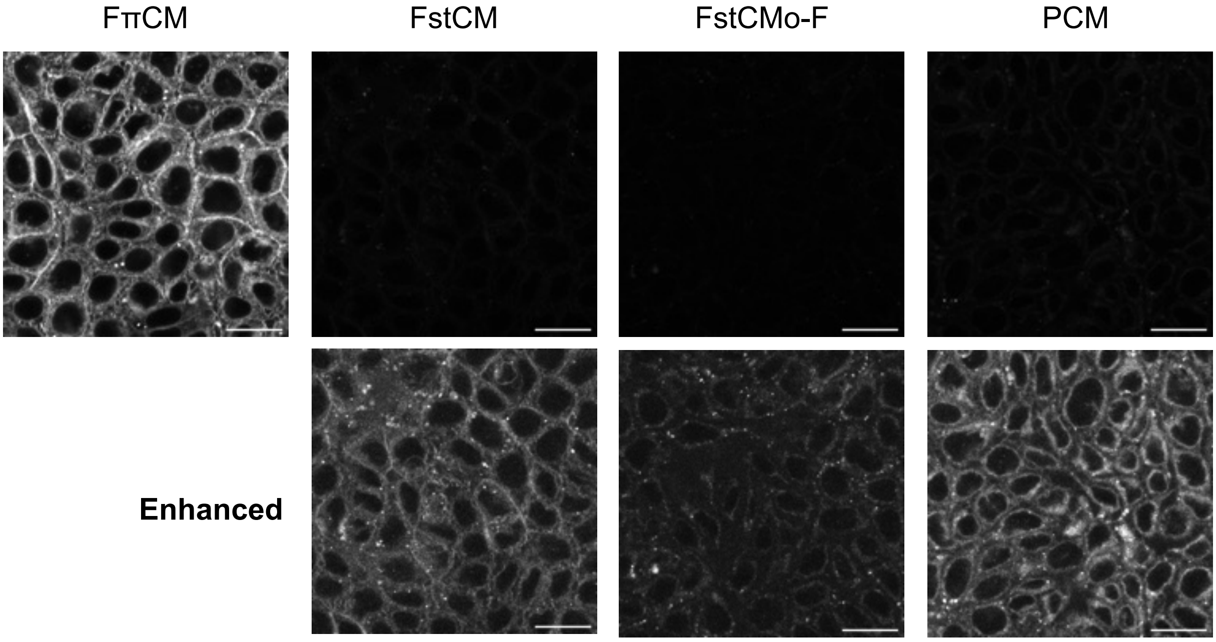

Figure S138. Confocal fluorescence images of EpH4 stained with 1 μM FπCM, FstCM, FstCMo-F or PCM for 10 min. All images were obtained under the same conditions with laser intensity (405 nm, 10%) and detecter gains (800 V). All available signals (400-700 nm) were detected. Comparison of 10x enhanced images for FstCM, FstCMo-F and PCM are also shown in lower panels. Bars, 20 μm.

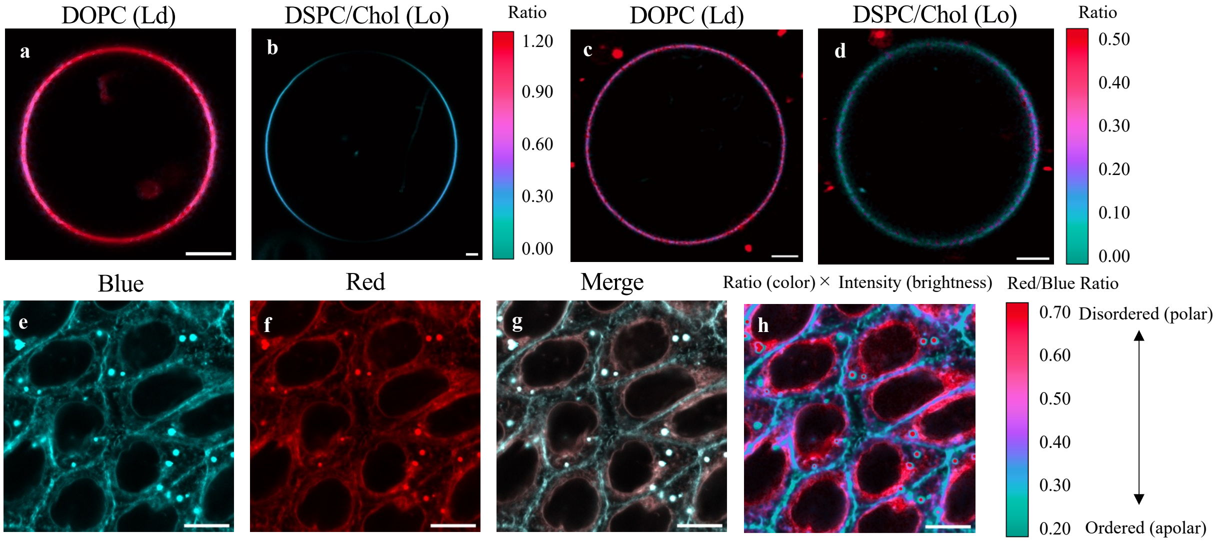

Figure S139. Confocal fluorescence images. **a.** Ld phase in GUVs stained with **FπCM**. **b**. Lo phase in GUVs stained with **FπCM**. **c**. Ld phase in GUVs stained with **PCM**. d. Lo phase in GUVs stained with **PCM**. a, b, c, d detected the differences in order of GUVs. **e**. blue image of EpH4 stained with **FπCM**. f. red image of EpH4 stained with **FπCM**. g. merge image of EpH4 stained with **FπCM**. h. ratio × intensity image of EpH4 stained with **FπCM**. **e**, **f**, **g**, **h** detected membrane order heterogeneity in living cells. One-photon laser excitation at 405 nm was employed. GUVs were composed of DOPC (Ld) and DSPC/Chol (Lo). Images obtained by collecting the fluorescence intensity recorded for both channels: 400 ~ 450 nm and 450 ~ 700 nm for **PCM**, 400 ~ 500 nm and 500 ~ 700 nm for **FπCM**. Probe concentration was 100 μM for **PCM** and 1 μM for **FπCM**. The scale bar is 5 μm for GUVs and 10 μm for EpH4.

S7. Cartesian coordinates information

B3LYP/6-311+G(d,p) for FπCM and FπA, ωB97XD/6-311+G(d) for PCM and PK

In Gas phase

Table S41. Franck-Condon state in XYZ format for **FπCM calculated by ωB97XD/6-31G(d,p)**.

	Coordinates (Å)		
Atom No.	X	Y	Z
6	-4.663271	0.74619	0.177848
6	-2.949442	-0.950012	-0.097791
6	-3.928092	-1.927887	-0.227977
6	-5.268993	-1.576271	-0.154006

6	-5.668406	-0.238074	0.060313
1	-4.922368	1.788338	0.325501
1	-3.658788	-2.967346	-0.392934
1	-6.013721	-2.353989	-0.266466
6	-1.486498	-1.014691	-0.136388
6	-0.614265	-2.084871	-0.303052
6	-0.982297	0.283147	0.036639
6	0.756534	-1.846366	-0.294419
1	-0.990335	-3.093499	-0.446286
6	0.380268	0.515788	0.043123
6	1.271968	-0.554876	-0.123271
1	1.442837	-2.673669	-0.44894
1	0.767685	1.519327	0.201365
6	-2.113324	1.288017	0.208747
6	-3.332509	0.381032	0.102484
6	-2.099879	2.338539	-0.912932
1	-2.956377	3.013971	-0.817906
1	-1.18689	2.941017	-0.865519
1	-2.146648	1.859788	-1.894597
6	-2.046548	1.971831	1.583633
1	-2.055515	1.230679	2.387092
1	-1.132592	2.567784	1.673975
1	-2.902531	2.640349	1.722159
6	2.736011	-0.320193	-0.11885
6	3.605464	-1.213574	0.519778
6	3.283653	0.803339	-0.751828
6	4.975758	-0.996283	0.526237
1	3.197297	-2.075879	1.037684
6	4.6517	1.027405	-0.744874
1	2.628694	1.493695	-1.274225
6	5.507855	0.128925	-0.106394
1	5.637558	-1.691246	1.029874
1	5.079419	1.892708	-1.239546
7	-7.008672	0.097504	0.157995
6	-8.012403	-0.889096	-0.176034
1	-9.002825	-0.452145	-0.043775
1	-7.929884	-1.246199	-1.213616
1	-7.948166	-1.757648	0.488794
6	-7.388148	1.49307	0.167014

1	-7.07044	2.026829	-0.741528
1	-8.473175	1.569715	0.245425
1	-6.961375	2.012112	1.032633
6	6.967181	0.417484	-0.130508
8	7.467695	1.381526	-0.665951
8	7.687606	-0.518336	0.512332
6	9.096041	-0.29611	0.523562
1	9.523358	-1.132334	1.075328
1	9.489895	-0.268358	-0.494991
1	9.333044	0.649171	1.017169

Table S42. Minimum excited singlet state in XYZ format for **FπCM calculated by ωB97XD/6-31G(d,p)**.

	Coordinates (Å)		
Atom No.	X	Y	Z
6	-4.659741	0.7588	0.063824
6	-2.922207	-0.952692	-0.065543
6	-3.91235	-1.962387	-0.118358
6	-5.242904	-1.614809	-0.081427
6	-5.659124	-0.254563	0.010208
1	-4.937761	1.804058	0.133923
1	-3.62909	-3.007648	-0.187546
1	-5.985782	-2.401448	-0.123074
6	-1.510881	-1.011012	-0.083613
6	-0.606147	-2.104671	-0.156126
6	-0.98757	0.321338	-0.007273
6	0.73872	-1.861652	-0.142455
1	-0.978097	-3.121923	-0.232644
6	0.354333	0.558927	-0.003041
6	1.289031	-0.530175	-0.066727
1	1.416346	-2.702248	-0.233263
1	0.718568	1.576282	0.091827
6	-2.125076	1.332487	0.07295
6	-3.336909	0.411913	0.026278
6	-2.105961	2.289822	-1.129734
1	-2.960623	2.973805	-1.094953
1	-1.189808	2.888647	-1.123612

1	-2.144971	1.734147	-2.070106
6	-2.079112	2.124955	1.389484
1	-2.096535	1.451308	2.249983
1	-1.164171	2.723331	1.441478
1	-2.935025	2.804285	1.463106
6	2.705637	-0.305458	-0.058957
6	3.63866	-1.356115	0.198266
6	3.262783	0.987308	-0.304694
6	4.995711	-1.133207	0.214222
1	3.279082	-2.351803	0.432242
6	4.62005	1.201394	-0.296846
1	2.610621	1.818496	-0.54809
6	5.518792	0.150283	-0.035101
1	5.675839	-1.949523	0.429494
1	5.027232	2.185933	-0.501356
7	-6.989681	0.069923	0.046121
6	-8.000154	-0.966762	-0.012722
1	-8.987958	-0.509177	0.026342
1	-7.931472	-1.545058	-0.942648
1	-7.915334	-1.661817	0.832082
6	-7.39806	1.456452	0.143285
1	-7.04523	2.042587	-0.714724
1	-8.485825	1.511214	0.163899
1	-7.017676	1.925621	1.059366
6	6.956922	0.442974	-0.035086
8	7.450188	1.536754	-0.240212
8	7.712175	-0.651879	0.222737
6	9.114377	-0.42182	0.235883
1	9.572815	-1.386537	0.453339
1	9.457702	-0.047377	-0.732052
1	9.383093	0.308404	1.003753

Table S43. Franck-Condon state in XYZ format for **FπCM calculated by B3LYP/6-311+G(d,p)**.

	Coordinates (Å)		
Atom No.	X	Y	Z
6	-4.682696	0.74134	0.174981

6	-2.952894	-0.946841	-0.094108
6	-3.930699	-1.93216	-0.221903
6	-5.275543	-1.589558	-0.149414
6	-5.686981	-0.250962	0.062305
1	-4.954556	1.778264	0.319693
1	-3.658398	-2.969849	-0.384058
1	-6.012805	-2.372106	-0.258925
6	-1.492798	-1.006629	-0.13307
6	-0.613385	-2.077881	-0.29496
6	-0.985706	0.296438	0.034525
6	0.758362	-1.839835	-0.284813
1	-0.984521	-3.087324	-0.435852
6	0.379246	0.526945	0.038102
6	1.279292	-0.544994	-0.120578
1	1.439878	-2.669389	-0.434983
1	0.767301	1.528542	0.191118
6	-2.124638	1.302933	0.205743
6	-3.34644	0.387603	0.101824
6	-2.115032	2.35957	-0.919904
1	-2.971891	3.032789	-0.824976
1	-1.205646	2.96551	-0.873361
1	-2.160558	1.885349	-1.902809
6	-2.057975	1.995862	1.584083
1	-2.064278	1.261231	2.392328
1	-1.146768	2.594252	1.672465
1	-2.913427	2.663413	1.722012
6	2.742463	-0.31145	-0.115707
6	3.621228	-1.224348	0.491458
6	3.296705	0.831752	-0.717289
6	4.993174	-1.008832	0.497485
1	3.220267	-2.100806	0.986891
6	4.66637	1.053035	-0.711715
1	2.646513	1.539949	-1.217422
6	5.530745	0.134999	-0.10457
1	5.652699	-1.719523	0.97802
1	5.088012	1.931241	-1.185193
7	-7.033253	0.076225	0.169031
6	-8.040558	-0.913088	-0.179648
1	-9.028705	-0.491238	0.000017

1	-7.984582	-1.224938	-1.233182
1	-7.947326	-1.80535	0.445348
6	-7.431527	1.474356	0.150863
1	-7.162265	1.981766	-0.787589
1	-8.511874	1.537301	0.275264
1	-6.974616	2.023958	0.978577
6	6.989678	0.419244	-0.128521
8	7.494103	1.395512	-0.63778
8	7.716024	-0.541812	0.488557
6	9.138697	-0.336416	0.506972
1	9.548887	-1.19436	1.035588
1	9.53122	-0.288046	-0.509797
1	9.384657	0.589709	1.028549

Table S44. Minimum excited singlet state in XYZ format for **FπCM calculated by B3LYP/6-311+G(d,p)**.

	Coordinates (Å)		
Atom No.	X	Y	Z
6	-4.681908	0.756782	0.102442
6	-2.949854	-0.942489	-0.097535
6	-3.927758	-1.948337	-0.184439
6	-5.267278	-1.611734	-0.130038
6	-5.682732	-0.256497	0.014421
1	-4.967447	1.79413	0.211586
1	-3.641801	-2.98783	-0.292468
1	-6.005268	-2.398184	-0.196995
6	-1.504494	-1.002507	-0.121741
6	-0.619186	-2.085089	-0.238979
6	-0.99591	0.310227	0.003014
6	0.742808	-1.846301	-0.237185
1	-0.989864	-3.099517	-0.344537
6	0.36685	0.54324	0.02069
6	1.283438	-0.532457	-0.103423
1	1.422687	-2.678574	-0.36368
1	0.749188	1.547648	0.155046
6	-2.131021	1.329506	0.119797
6	-3.353591	0.414846	0.045606
6	-2.109313	2.331026	-1.055689

1	-2.951554	3.026712	-0.993129
1	-1.187349	2.916979	-1.034813
1	-2.158847	1.81304	-2.015924
6	-2.07714	2.086089	1.464981
1	-2.103041	1.39282	2.308488
1	-1.155265	2.668356	1.534343
1	-2.91933	2.778274	1.558914
6	2.731009	-0.302937	-0.092814
6	3.640657	-1.308507	0.329128
6	3.28922	0.938651	-0.501117
6	5.003506	-1.092179	0.354547
1	3.26284	-2.262652	0.680091
6	4.649825	1.158108	-0.494469
1	2.639794	1.72627	-0.867852
6	5.552218	0.150324	-0.061427
1	5.668421	-1.87218	0.701395
1	5.058854	2.103491	-0.828963
7	-7.015938	0.06654	0.066579
6	-8.038671	-0.968891	-0.036021
1	-9.020678	-0.505632	0.016566
1	-7.963187	-1.507749	-0.985816
1	-7.955296	-1.691837	0.781921
6	-7.439076	1.453673	0.224985
1	-7.094307	2.072095	-0.610098
1	-8.525036	1.493554	0.253677
1	-7.054812	1.88151	1.156388
6	6.97536	0.434715	-0.065882
8	7.499719	1.491391	-0.405772
8	7.73561	-0.6346	0.365797
6	9.144736	-0.412842	0.38322
1	9.586107	-1.342423	0.742445
1	9.518716	-0.178739	-0.616504
1	9.405248	0.411819	1.051232

Table S45. Franck-Condon state in XYZ format for **FπA calculated by ωB97XD/6-31G(d,p)**.

	Coordinates (Å)		
Atom No.	X	Y	Z

6	4.279983	0.765568	0.0751
6	2.569887	-0.939907	-0.163401
6	3.550751	-1.915844	-0.291365
6	4.890932	-1.557872	-0.239272
6	5.287349	-0.213153	-0.064526
1	4.536405	1.807844	0.226107
1	3.283754	-2.959951	-0.42796
1	5.637279	-2.336083	-0.335616
6	1.10679	-1.012154	-0.172555
6	0.237196	-2.087657	-0.316906
6	0.599448	0.283961	0.003961
6	-1.134305	-1.856159	-0.282391
1	0.615813	-3.094788	-0.463496
6	-0.763911	0.509645	0.035996
6	-1.652949	-0.56656	-0.107101
1	-1.819197	-2.687528	-0.420168
1	-1.153333	1.51195	0.196493
6	1.72842	1.295362	0.148638
6	2.949853	0.394274	0.020745
6	1.686249	1.984106	1.522129
1	2.541332	2.657496	1.64068
1	0.771459	2.57592	1.628798
1	1.715238	1.245988	2.327866
6	1.686293	2.341503	-0.976567
1	1.715857	1.859122	-1.957024
1	0.771264	2.939234	-0.912823
1	2.54094	3.021611	-0.90152
6	-3.117637	-0.339026	-0.074511
6	-3.9699	-1.232924	0.58396
6	-3.684904	0.780005	-0.699962
6	-5.341289	-1.01883	0.615132
1	-3.548593	-2.091715	1.09708
6	-5.052504	0.996713	-0.665974
1	-3.043439	1.47156	-1.237533
6	-5.899627	0.099955	-0.00839
1	-5.972427	-1.728478	1.140528
1	-5.496007	1.857705	-1.154361
7	6.627585	0.136424	-0.037372
6	7.629677	-0.904533	0.034187

1	8.619664	-0.448245	0.064629
1	7.517947	-1.541972	0.924048
1	7.594703	-1.548185	-0.851916
6	6.996944	1.479955	0.350719
1	6.648216	1.742034	1.361062
1	8.083139	1.574308	0.330333
1	6.59214	2.218505	-0.350458
6	-7.369449	0.383461	-0.004559
6	-8.29324	-0.585292	0.705013
1	-8.213484	-1.586934	0.270835
1	-8.033138	-0.663801	1.765397
1	-9.31747	-0.226868	0.607401
8	-7.812687	1.371089	-0.559422

Table S46. Minimum excited singlet state in XYZ format for **FπA calculated by ωB97XD/6-31G(d,p)**.

	Coordinates (Å)		
Atom No.	X	Y	Z
6	4.286985	0.765281	0.064431
6	2.576224	-0.941842	-0.16005
6	3.557435	-1.917955	-0.281219
6	4.89812	-1.559173	-0.232935
6	5.294487	-0.213766	-0.069271
1	4.542925	1.80875	0.208172
1	3.290597	-2.963209	-0.409289
1	5.644228	-2.338335	-0.323819
6	1.112723	-1.01389	-0.167093
6	0.241109	-2.088898	-0.301623
6	0.605527	0.283093	0.000077
6	-1.129961	-1.855388	-0.269914
1	0.618173	-3.09783	-0.4411
6	-0.757615	0.509945	0.034964
6	-1.651601	-0.564439	-0.101622
1	-1.81427	-2.68764	-0.405306
1	-1.144055	1.513656	0.194141
6	1.735063	1.295421	0.136431
6	2.956682	0.393673	0.013819

6	1.694652	1.994213	1.5048
1	2.549562	2.668969	1.617804
1	0.779416	2.585934	1.608382
1	1.724692	1.26166	2.315576
6	1.69198	2.333472	-0.9961
1	1.721019	1.843703	-1.972927
1	0.776211	2.93053	-0.936206
1	2.546205	3.014916	-0.926827
6	-3.113121	-0.336888	-0.069894
6	-3.978255	-1.246585	0.56108
6	-3.685986	0.7969	-0.663222
6	-5.34309	-1.045828	0.601299
1	-3.560556	-2.11646	1.059436
6	-5.049441	1.019724	-0.638713
1	-3.04611	1.506345	-1.179901
6	-5.921553	0.09592	-0.007757
1	-5.964802	-1.761836	1.127898
1	-5.45993	1.899166	-1.122816
7	6.636485	0.137135	-0.047967
6	7.635335	-0.904631	0.051685
1	8.626494	-0.450233	0.076552
1	7.517869	-1.52345	0.954196
1	7.602813	-1.566516	-0.820788
6	7.003483	1.479264	0.34714
1	6.659371	1.735323	1.360915
1	8.089354	1.577435	0.320516
1	6.592573	2.220033	-0.347799
6	-7.322848	0.325979	0.0055
6	-8.361359	-0.588361	0.617078
1	-8.051578	-1.626879	0.486214
1	-8.487005	-0.37879	1.685592
1	-9.325398	-0.446825	0.123839
8	-7.841631	1.417524	-0.473755

Table S47. Franck-Condon state in XYZ format for **FπA calculated by B3LYP/6-311+G(d,p)**.

	Coordinates (Å)		
Atom No.	X	Y	Z
6	-4.295693	0.75036	0.159797

6	-2.571539	-0.945696	-0.095674
6	-3.552791	-1.926493	-0.231572
6	-4.89659	-1.577734	-0.169874
6	-5.303578	-0.237301	0.038802
1	-4.563952	1.788496	0.30253
1	-3.283954	-2.965393	-0.391745
1	-5.636527	-2.35687	-0.285502
6	-1.111466	-1.012179	-0.122856
6	-0.235699	-2.087428	-0.277867
6	-0.59977	0.288517	0.049108
6	1.136999	-1.855683	-0.256591
1	-0.610313	-3.095132	-0.421968
6	0.766152	0.512755	0.063757
6	1.662522	-0.563276	-0.087878
1	1.815892	-2.68833	-0.40143
1	1.157551	1.512533	0.220116
6	-1.735417	1.300199	0.211337
6	-2.960527	0.390506	0.097358
6	-1.71187	2.35698	-0.91397
1	-2.56637	3.034109	-0.825821
1	-0.800119	2.958731	-0.859954
1	-1.751632	1.883147	-1.897312
6	-1.67671	1.992572	1.590319
1	-1.692912	1.257834	2.398329
1	-0.76351	2.586757	1.686185
1	-2.530175	2.664018	1.721477
6	3.126662	-0.336452	-0.07114
6	3.996296	-1.253482	0.542907
6	3.690986	0.8043	-0.667982
6	5.369123	-1.044267	0.56006
1	3.587329	-2.128177	1.034898
6	5.061562	1.019292	-0.651299
1	3.048111	1.515562	-1.173198
6	5.916783	0.097191	-0.03739
1	6.021477	-1.758063	1.045754
1	5.49104	1.895637	-1.121169
7	-6.649153	0.096044	0.134722
6	-7.658138	-0.888573	-0.222293
1	-8.645757	-0.462223	-0.050524

1	-7.595086	-1.200491	-1.275407
1	-7.574052	-1.781364	0.403245
6	-7.040847	1.495991	0.113637
1	-6.761688	2.002326	-0.822502
1	-8.121863	1.563873	0.229322
1	-6.588119	2.043344	0.945131
6	7.377151	0.37474	-0.049497
8	7.814867	1.417566	-0.601303
6	8.354353	-0.613919	0.613248
1	8.118547	-0.705629	1.652904
1	8.267895	-1.570736	0.142159
1	9.355582	-0.252316	0.50516

Table S48. Minimum excited singlet state in XYZ format for **FπA calculated by B3LYP/6-311+G(d,p)**.

	Coordinates (Å)		
Atom No.	X	Y	Z
6	-4.295032	0.773361	0.066917
6	-2.57024	-0.939124	-0.082066
6	-3.550672	-1.949534	-0.149278
6	-4.885313	-1.609743	-0.107889
6	-5.299605	-0.244341	0.002596
1	-4.581997	1.812327	0.151028
1	-3.264335	-2.991521	-0.232818
1	-5.627654	-2.393012	-0.16129
6	-1.133189	-1.003969	-0.098007
6	-0.248459	-2.09494	-0.184124
6	-0.613989	0.31222	-0.001706
6	1.113446	-1.86385	-0.172994
1	-0.625303	-3.108685	-0.269394
6	0.746479	0.537867	0.015219
6	1.662823	-0.548527	-0.070674
1	1.782027	-2.709817	-0.267613
1	1.121282	1.548473	0.118957
6	-1.74573	1.33714	0.086861
6	-2.970512	0.424142	0.026065
6	-1.71416	2.316185	-1.105901
1	-2.560389	3.007622	-1.059017
1	-0.794369	2.906908	-1.086601

1	-1.757336	1.782364	-2.058714
6	-1.69515	2.121959	1.414904
1	-1.722724	1.448413	2.275296
1	-0.776008	2.711239	1.471641
1	-2.542286	2.809861	1.487912
6	3.106818	-0.328852	-0.053627
6	4.024896	-1.369214	0.26685
6	3.679093	0.942045	-0.352819
6	5.38925	-1.159628	0.294396
1	3.653612	-2.353211	0.529085
6	5.040075	1.152538	-0.335957
1	3.035418	1.768552	-0.632102
6	5.957489	0.111538	-0.007042
1	6.032985	-1.989064	0.563563
1	5.441147	2.12796	-0.584726
7	-6.61963	0.078759	0.046459
6	-7.650146	-0.957444	-0.011768
1	-8.627072	-0.489647	0.075356
1	-7.605798	-1.501005	-0.960356
1	-7.531826	-1.671539	0.807761
6	-7.044082	1.474864	0.140325
1	-6.685883	2.050274	-0.718344
1	-8.129673	1.513885	0.155032
1	-6.665285	1.937723	1.0562
6	7.385833	0.375461	0.010957
8	7.854335	1.50947	-0.24393
6	8.339009	-0.760448	0.35081
1	8.154876	-1.159701	1.354889
1	8.243035	-1.597617	-0.349943
1	9.362695	-0.386084	0.308134

Table S49. Franck-Condon state in XYZ format for **FπCM*o*-F calculated by B3LYP/6-311+G(d,p)**.

	Coordinates (Å)		
Atom No.	X	Y	Z
6	-4.919943	0.731406	0.2056
6	-3.169734	-0.918204	-0.154433

6	-4.134964	-1.909568	-0.325768
6	-5.483446	-1.589282	-0.229927
6	-5.911519	-0.267995	0.049264
1	-5.204999	1.756163	0.402189
1	-3.849665	-2.934261	-0.539772
1	-6.210994	-2.375287	-0.373942
6	-1.7098	-0.956636	-0.202891
6	-0.81758	-2.00746	-0.420708
6	-1.218693	0.343606	0.025746
6	0.550737	-1.752618	-0.404435
1	-1.176316	-3.013564	-0.60888
6	0.142778	0.591667	0.033951
6	1.054998	-0.460335	-0.179856
1	1.241516	-2.5654	-0.59777
1	0.51825	1.589692	0.233677
6	-2.369618	1.325307	0.251264
6	-3.579685	0.399744	0.108547
6	-2.379908	2.43558	-0.821536
1	-3.245407	3.091248	-0.689938
1	-1.478841	3.05134	-0.749287
1	-2.423956	2.009513	-1.82631
6	-2.304484	1.950877	1.661551
1	-2.296184	1.17777	2.433036
1	-1.401269	2.557173	1.774591
1	-3.168248	2.598787	1.836064
6	2.513703	-0.206764	-0.169733
6	3.403436	-1.146896	0.365303
6	3.054506	0.981288	-0.694001
6	4.767664	-0.905943	0.368922
1	3.047467	-2.066173	0.812761
6	4.419836	1.206339	-0.678727
1	2.399267	1.718064	-1.141714
6	5.322581	0.270759	-0.147457
1	4.834845	2.117132	-1.091956
7	-7.260526	0.034764	0.177698
6	-8.257391	-0.951626	-0.207809
1	-9.24964	-0.551047	-0.004135
1	-8.204099	-1.21575	-1.274268
1	-8.148651	-1.869333	0.376665

6	-7.678696	1.425831	0.240256
1	-7.422605	1.988476	-0.669815
1	-8.758977	1.466123	0.373731
1	-7.22434	1.934972	1.094897
6	6.76922	0.627284	-0.192289
8	7.180323	1.677495	-0.636367
8	7.57723	-0.325532	0.303125
6	8.984463	-0.027141	0.280867
1	9.468477	-0.89901	0.715147
1	9.324885	0.133197	-0.743058
1	9.195132	0.865372	0.871668
9	5.545205	-1.862176	0.915364

Table S50. Franck-Condon state in XYZ format for **FstCM*o*-F calculated by B3LYP/6-311+G(d,p)**.

	Coordinates (Å)		
Atom No.	X	Y	Z
9	-6.436658	-2.237507	-0.003059
8	-8.709086	-0.928833	0.012359
7	8.177177	-0.56768	-0.086207
8	-8.668421	1.318933	0.02459
6	4.483498	-0.386706	-0.034801
6	2.832164	1.257833	-0.017947
6	4.268588	1.002381	-0.025907
6	2.15457	0.018516	-0.021641
6	5.761315	-0.917753	-0.040051
1	5.895152	-1.991101	-0.039702
6	6.887317	-0.05799	-0.046478
6	3.152442	-1.141221	-0.031596
6	-3.740381	0.186543	0.000797
6	2.102521	2.446887	-0.008665
1	2.602857	3.409097	-0.006002
6	0.019399	1.160526	-0.007195
6	-6.594555	0.151158	0.010818

6	6.6554	1.339864	-0.022916
1	7.489482	2.026888	-0.0091
6	5.368081	1.86082	-0.016756
1	5.2341	2.937337	0.000048
6	-5.841684	-1.027829	0.001549
6	0.775517	-0.033484	-0.016422
1	0.269361	-0.992697	-0.019387
6	0.712816	2.386369	-0.003438
1	0.140772	3.308633	0.003549
6	-1.439491	1.187361	-0.001657
1	-1.864566	2.187616	0.005715
6	-2.280808	0.132848	-0.00479
1	-1.866992	-0.871375	-0.012228
6	3.006468	-2.017725	1.23104
1	3.76814	-2.802572	1.24623
1	2.025681	-2.501126	1.254908
6	-5.866729	1.356009	0.01493
1	-6.436579	2.276761	0.022065
6	-4.486488	1.383772	0.010146
1	-3.982588	2.341718	0.013633
6	-4.456842	-1.020089	-0.003379
1	-3.940115	-1.972609	-0.010574
6	-8.07933	0.259204	0.01667
6	8.390992	-1.993249	0.103418
1	7.867021	-2.574722	-0.660555
1	9.454069	-2.20906	0.005518
6	2.9941	-2.006703	-1.300327
1	2.012708	-2.489095	-1.31937
1	3.754919	-2.791986	-1.329266
6	9.308908	0.320658	0.127215
1	9.300074	0.787666	1.122878
1	10.231305	-0.249617	0.025663
6	-10.146476	-0.875175	0.017652
1	-10.506643	-0.362314	0.910597
1	-10.512817	-0.352336	-0.866953
1	-10.471479	-1.913185	0.012958
1	3.094501	-1.400412	-2.203263
1	3.114558	-1.419107	2.138211
1	9.331395	1.116341	-0.622624

1	8.057555	-2.345169	1.090827

Table S51. Franck-Condon state in XYZ format for **FCM calculated by ωB97XD/6-31G(d,p)**.

	Coordinates (Å)		
Atom No.	X	Y	Z
6	-2.203054	0.810401	-0.000984
6	-0.457746	-0.863721	-0.018945
6	-1.402902	-1.885968	-0.012474
6	-2.753291	-1.554932	-0.000103
6	-3.1567	-0.215583	0.005742
1	-2.544705	1.841036	0.003749
1	-1.096265	-2.927433	-0.017206
1	-3.506566	-2.334031	0.005109
6	1.005249	-0.903111	-0.031528
6	1.906594	-1.961461	-0.033498
6	1.488352	0.410466	-0.034102
6	3.270106	-1.70529	-0.041514
1	1.558253	-2.990348	-0.024533
6	2.843499	0.681071	-0.041719
6	3.770715	-0.383265	-0.054302
1	3.953234	-2.545052	-0.03689
1	3.180082	1.711253	-0.036677
6	0.341874	1.412761	-0.021765
6	-0.862362	0.481828	-0.013196
6	0.356565	2.292837	-1.281947
1	-0.51083	2.960232	-1.294646
1	1.260646	2.909773	-1.308968
1	0.332264	1.678278	-2.185612
6	0.378524	2.28687	1.242145
1	0.36898	1.668036	2.14319
1	1.283198	2.903394	1.256829
1	-0.488108	2.95467	1.272648
7	5.132386	-0.139358	-0.083967
6	5.612867	1.213214	0.094424
1	5.303705	1.649373	1.056004
1	6.702532	1.216183	0.054425
1	5.254363	1.86933	-0.707048
6	6.056383	-1.23708	0.100041

1	7.07772	-0.857027	0.061508
1	5.918038	-1.750611	1.062966
1	5.95388	-1.979536	-0.699574
6	-4.588892	0.177707	0.019173
8	-4.988162	1.322276	0.024169
8	-5.418324	-0.881955	0.024492
6	-6.806626	-0.563283	0.036809
1	-7.329852	-1.518709	0.039884
1	-7.062574	0.015402	0.927444
1	-7.077722	0.017963	-0.847655

Table S52. Minimum excited singlet state in XYZ format for **FCM calculated by ωB97XD/6-31G(d,p)**.

	Coordinates (Å)		
Atom No.	X	Y	Z
6	2.187182	0.838945	0.0015
6	0.434109	-0.860179	0.018793
6	1.401385	-1.913657	0.01225
6	2.737291	-1.585316	0.000491
6	3.153417	-0.215245	-0.00513
1	2.533499	1.867425	-0.003118
1	1.096248	-2.95548	0.016678
1	3.488159	-2.366246	-0.004584
6	-0.974902	-0.898345	0.030255
6	-1.894833	-1.990684	0.032478
6	-1.478236	0.444892	0.032954
6	-3.246394	-1.731967	0.040686
1	-1.546525	-3.018564	0.02371
6	-2.822068	0.696089	0.040841
6	-3.756366	-0.396991	0.050831
1	-3.927407	-2.573707	0.037118
1	-3.165487	1.72402	0.037027
6	-0.340437	1.458646	0.021214

6	0.857855	0.52024	0.01314
6	-0.35599	2.339189	1.280991
1	0.512621	3.005054	1.293429
1	-1.260116	2.95689	1.309254
1	-0.330946	1.724491	2.184534
6	-0.376761	2.333882	-1.241893
1	-0.365708	1.715359	-2.143133
1	-1.281433	2.951254	-1.258288
1	0.491156	3.000126	-1.271084
7	-5.119718	-0.158853	0.081578
6	-5.616733	1.190153	-0.09477
1	-5.320239	1.624807	-1.059751
1	-6.705871	1.181506	-0.046432
1	-5.258892	1.850018	0.70263
6	-6.044323	-1.258702	-0.094138
1	-7.066142	-0.88106	-0.049847
1	-5.908591	-1.770259	-1.057526
1	-5.933392	-1.99856	0.705842
6	4.572606	0.167199	-0.017999
8	4.977514	1.31506	-0.02349
8	5.400048	-0.900291	-0.024109
6	6.787995	-0.586005	-0.036891
1	7.308899	-1.542939	-0.040591
1	7.046511	-0.007488	-0.927205
1	7.062412	-0.005705	0.847482

Table S53. Franck-Condon state in XYZ format for PCM calculated by ωB97XD/6-31G(d,p).

	Coordinates (Å)		
Atom No.	X	Y	Z
8	-5.27211	1.285201	0.193531
8	-5.893274	-0.853343	0.047503
7	3.754156	0.080087	-0.10213
6	1.391564	-1.483406	-0.344427
1	2.36703	-1.933848	-0.490443
6	-2.473321	1.828012	0.099618
1	-3.440064	2.303032	0.172713
6	-0.041099	1.971296	0.015744

6	-2.425808	0.38899	-0.005313
6	2.496336	0.730362	-0.095078
6	0.267822	-2.235888	-0.370694
1	0.327155	-3.310298	-0.518394
6	-1.341056	2.568716	0.102507
1	-1.405039	3.650972	0.176829
6	-1.032087	-1.641275	-0.231794
6	-1.14239	-0.231246	-0.104715
6	1.13144	2.728379	0.02127
1	1.062488	3.810706	0.086051
6	1.326568	-0.059831	-0.163717
6	0.051986	0.560147	-0.080424
6	-4.958888	0.118006	0.08509
6	4.106241	-0.568587	1.165794
1	3.224009	-1.078713	1.558764
1	4.401505	0.191632	1.913346
6	2.377015	2.119997	-0.02305
1	3.269052	2.73497	0.019278
6	-3.577214	-0.436932	-0.019829
6	4.876842	0.828148	-0.652892
1	5.22345	1.618031	0.04136
1	4.548105	1.319128	-1.5731
6	-2.19379	-2.418765	-0.244989
1	-2.112784	-3.497169	-0.342581
6	6.041721	-0.117607	-0.939113
1	6.882634	0.453931	-1.345599
1	5.727884	-0.835882	-1.705258
6	-3.435006	-1.82549	-0.137592
1	-4.324801	-2.441243	-0.147433
6	5.249866	-1.557088	0.960534
1	4.903731	-2.364526	0.303643
1	5.518733	-2.007774	1.921551
6	6.458634	-0.863445	0.329693
1	7.248563	-1.588328	0.109289
1	6.876143	-0.145259	1.048497
6	-7.240409	-0.401469	0.143219
1	-7.482994	0.270348	-0.683328
1	-7.405187	0.128027	1.084413
1	-7.855437	-1.299356	0.097999

Table S54. Minimum excited singlet state in XYZ format for **PCM calculated by ωB97XD/6-31G(d,p)**.

	Coordinates (Å)		
Atom No.	X	Y	Z
8	-5.262196	1.272706	0.189906
8	-5.890432	-0.874503	0.038693
7	3.766189	0.212068	0.034585
6	1.430515	-1.434503	-0.31858
1	2.396892	-1.891489	-0.493075
6	-2.469073	1.801667	0.078316
1	-3.442402	2.268751	0.139085
6	-0.04839	1.997878	0.011132
6	-2.410584	0.38632	-0.012262
6	2.515404	0.801551	-0.02876
6	0.281935	-2.208262	-0.361891
1	0.362462	-3.282443	-0.503344
6	-1.325667	2.576276	0.077838
1	-1.412189	3.65786	0.129325
6	-0.993235	-1.639715	-0.232276
6	-1.122361	-0.220792	-0.096749
6	1.136325	2.793568	-0.010705
1	1.042641	3.874147	0.030629
6	1.343513	-0.039402	-0.111435
6	0.058563	0.575699	-0.054152
6	-4.941428	0.098376	0.077674
6	4.065013	-0.708653	1.137676
1	3.142002	-1.193569	1.455561
1	4.430857	-0.111966	1.991019
6	2.374882	2.206725	-0.035126
1	3.26159	2.829321	0.009523
6	-3.585107	-0.449066	-0.028514
6	4.934701	0.936522	-0.444039
1	5.337483	1.592237	0.348362
1	4.631371	1.568541	-1.281228
6	-2.180968	-2.4261	-0.253368
1	-2.091596	-3.503807	-0.355949
6	6.018482	-0.045214	-0.894413

1	6.898399	0.522214	-1.214146
1	5.648087	-0.596308	-1.766339
6	-3.418756	-1.845378	-0.150005
1	-4.303605	-2.468811	-0.165338
6	5.123715	-1.729327	0.736387
1	4.71576	-2.381603	-0.04532
1	5.35648	-2.360929	1.599347
6	6.380133	-1.025977	0.221202
1	7.112577	-1.756321	-0.13542
1	6.851927	-0.47799	1.047904
6	-7.228352	-0.409819	0.135513
1	-7.469582	0.266173	-0.689135
1	-7.391838	0.122225	1.076447
1	-7.855173	-1.300581	0.090429

Table S55. Franck-Condon state in XYZ format for **PCM calculated by ωB97XD/6-311+G(d,p)**.

	Coordinates (Å)		
Atom No.	X	Y	Z
8	-5.264804	1.280707	0.200699
8	-5.885907	-0.850492	0.045699
7	3.750595	0.081795	-0.101901
6	1.389192	-1.480402	-0.346901
1	2.362992	-1.931303	-0.492001
6	-2.468903	1.828103	0.100299
1	-3.433302	2.304705	0.174399
6	-0.040203	1.9701	0.014799
6	-2.422705	0.390003	-0.004801
6	2.493996	0.730396	-0.097101
6	0.267591	-2.2309	-0.374201
1	0.32659	-3.3038	-0.523901
6	-1.338802	2.567102	0.102199
1	-1.4026	3.648102	0.176099
6	-1.031208	-1.636899	-0.233901
6	-1.140906	-0.229198	-0.105001
6	1.130698	2.725898	0.018599

1	1.062	3.806998	0.082099
6	1.325294	-0.058202	-0.163801
6	0.052695	0.561	-0.080901
6	-4.954605	0.120107	0.087799
6	4.097594	-0.573206	1.163099
1	3.215293	-1.082905	1.552499
1	4.390695	0.185094	1.912299
6	2.374297	2.118197	-0.026401
1	3.265098	2.732795	0.013399
6	-3.572506	-0.434395	-0.019301
6	4.874896	0.828293	-0.648801
1	5.218897	1.616193	0.047599
1	4.550296	1.319294	-1.568801
6	-2.191309	-2.413197	-0.247501
1	-2.11091	-3.490197	-0.346701
6	6.038694	-0.117009	-0.932901
1	6.880895	0.45479	-1.333501
1	5.727993	-0.831408	-1.702601
6	-3.430208	-1.820595	-0.138601
1	-4.319809	-2.434094	-0.148501
6	5.240292	-1.560707	0.958899
1	4.895891	-2.366807	0.301199
1	5.506292	-2.012208	1.919099
6	6.450193	-0.867209	0.333399
1	7.238592	-1.59191	0.112699
1	6.866894	-0.15301	1.054999
6	-7.237206	-0.40899	0.143099
1	-7.483105	0.263311	-0.680801
1	-7.404905	0.11321	1.086699
1	-7.843507	-1.310889	0.093399

Table S56. Minimum excited singlet state in XYZ format for **PCM calculated by ωB97XD/6-311+G(d,p)**.

	Coordinates (Å)		
Atom No.	X	Y	Z
8	-5.255653	1.268361	0.193658
8	-5.883321	-0.871553	0.038565
7	3.762334	0.21408	0.037859

6	1.428531	-1.431284	-0.318523
1	2.393187	-1.889952	-0.487998
6	-2.463989	1.801857	0.079069
1	-3.43446	2.271411	0.140262
6	-0.046967	1.996351	0.01048
6	-2.40724	0.387757	-0.011331
6	2.513859	0.801414	-0.027311
6	0.280921	-2.203508	-0.364257
1	0.361091	-3.276109	-0.508036
6	-1.322149	2.574369	0.077488
1	-1.4084	3.654765	0.127413
6	-0.992197	-1.635819	-0.23411
6	-1.120683	-0.218661	-0.096027
6	1.136794	2.791453	-0.015892
1	1.043379	3.8709	0.021571
6	1.342571	-0.038588	-0.108653
6	0.059349	0.576226	-0.052811
6	-4.937139	0.100468	0.07932
6	4.059232	-0.710313	1.137289
1	3.137278	-1.191641	1.458229
1	4.427941	-0.11402	1.988608
6	2.372741	2.205322	-0.039587
1	3.258407	2.827441	-0.000446
6	-3.580427	-0.446226	-0.028651
6	4.932095	0.936114	-0.438803
1	5.335105	1.5875	0.355827
1	4.632308	1.570144	-1.273762
6	-2.179102	-2.420811	-0.257031
1	-2.090948	-3.497131	-0.361548
6	6.012175	-0.046542	-0.891251
1	6.893029	0.518887	-1.208146
1	5.641029	-0.592689	-1.764718
6	-3.414432	-1.840322	-0.152671
1	-4.299194	-2.461394	-0.168992
6	5.113848	-1.732218	0.734167
1	4.703728	-2.382519	-0.046493
1	5.345846	-2.364242	1.595603
6	6.370508	-1.031697	0.21969
1	7.09953	-1.762401	-0.139486

1	6.844446	-0.488607	1.047057
6	-7.225458	-0.416862	0.136374
1	-7.469871	0.257946	-0.686948
1	-7.391486	0.109648	1.07879
1	-7.843518	-1.311843	0.088591

Table S57. Franck-Condon state in XYZ format for **PK calculated by ωB97XD/6-31G(d,p)**.

	Coordinates (Å)		
Atom No.	X	Y	Z
8	-5.577837	1.249746	0.218543
7	3.424256	0.092231	-0.107807
6	1.071323	-1.48526	-0.34094
1	2.04902	-1.92946	-0.490904
6	-2.812227	1.801796	0.115555
1	-3.781146	2.270132	0.191779
6	-0.381474	1.960257	0.022073
6	-2.759535	0.363058	0.012167
6	2.162703	0.734756	-0.096921
6	-0.047577	-2.244807	-0.362199
1	0.017602	-3.318892	-0.509717
6	-1.683845	2.54936	0.113129
1	-1.754471	3.631303	0.186382
6	-1.350155	-1.657671	-0.218243
6	-1.47157	-0.248078	-0.091656
6	0.786843	2.724695	0.022234
1	0.711269	3.806603	0.086206
6	0.997449	-0.062176	-0.160489
6	-0.280999	0.549871	-0.07291
6	-5.313316	0.064404	0.113901
6	3.785241	-0.55378	1.159074
1	2.907513	-1.068488	1.556074
1	4.079423	0.208525	1.904849
6	2.035533	2.124114	-0.02619
1	2.924073	2.744342	0.012086
6	-3.91465	-0.466017	0.004054
6	4.540526	0.846337	-0.663642
1	4.88546	1.638461	0.02879
1	4.205273	1.334876	-1.582767

6	-2.505915	-2.442126	-0.226076
1	-2.419515	-3.520135	-0.323165
6	5.70939	-0.093138	-0.954202
1	6.54539	0.482858	-1.364422
1	5.396398	-0.813485	-1.718736
6	-3.749208	-1.853333	-0.113721
1	-4.618795	-2.498938	-0.121968
6	4.933434	-1.536016	0.949464
1	4.589065	-2.345772	0.294495
1	5.208818	-1.984617	1.909583
6	6.135681	-0.836024	0.313196
1	6.928623	-1.556672	0.089846
1	6.552267	-0.115237	1.029894
6	-6.469023	-0.926563	0.094833
1	-6.490839	-1.499364	-0.837075
1	-6.402425	-1.63664	0.924517
1	-7.391877	-0.354942	0.185603

Table S58. Minimum excited singlet state in XYZ format for **PK calculated by ωB97XD/6-31G(d,p)**.

	Coordinates (Å)		
Atom No.	X	Y	Z
8	-5.564146	1.2392	0.236547
7	3.43487	0.224225	0.027505
6	1.1112	-1.438194	-0.318037
1	2.080523	-1.887553	-0.495426
6	-2.809604	1.771747	0.089201
1	-3.788321	2.226628	0.152556
6	-0.390886	1.985471	0.011944
6	-2.74345	0.355885	0.002832
6	2.180248	0.805641	-0.033179
6	-0.030506	-2.218435	-0.357376
1	0.056161	-3.291912	-0.501049
6	-1.671516	2.554989	0.082719
1	-1.765812	3.63602	0.132287
6	-1.309646	-1.657827	-0.222311
6	-1.451047	-0.240681	-0.087325
6	0.787789	2.787725	-0.015128
1	0.687207	3.86779	0.024758

6	1.013903	-0.041621	-0.111899
6	-0.274397	0.563803	-0.051464
6	-5.293095	0.043476	0.112996
6	3.742027	-0.693206	1.131282
1	2.823462	-1.1849	1.451509
1	4.104845	-0.093178	1.983524
6	2.030599	2.209637	-0.041826
1	2.912818	2.838635	-0.000608
6	-3.922532	-0.48146	-0.004455
6	4.59787	0.957988	-0.451138
1	4.995865	1.616624	0.341192
1	4.289856	1.587716	-1.288352
6	-2.490921	-2.450767	-0.239268
1	-2.395952	-3.528007	-0.342635
6	5.688686	-0.015528	-0.90218
1	6.564079	0.558491	-1.222465
1	5.321871	-0.569328	-1.773914
6	-3.731693	-1.876242	-0.129748
1	-4.595745	-2.529978	-0.143869
6	4.80777	-1.706253	0.729353
1	4.404055	-2.361688	-0.051932
1	5.045919	-2.33589	1.592275
6	6.05854	-0.993689	0.21304
1	6.795982	-1.718619	-0.144279
1	6.527035	-0.442358	1.039368
6	-6.45514	-0.944167	0.081424
1	-6.483931	-1.511195	-0.854861
1	-6.401272	-1.663339	0.905438
1	-7.374761	-0.36687	0.174832

Table S59. Franck-Condon state in XYZ format for **PK calculated by ωB97XD/6-311+G(d,p)**.

	Coordinates (Å)		
Atom No.	X	Y	Z
8	-5.571745	1.244013	0.224468
7	3.420739	0.094442	-0.107851
6	1.069686	-1.482388	-0.343245
1	2.045912	-1.926678	-0.492221
6	-2.808154	1.800456	0.116706

1	-3.774536	2.270478	0.194116
6	-0.380973	1.95841	0.021321
6	-2.756296	0.362836	0.01286
6	2.160212	0.734946	-0.09909
6	-0.046901	-2.240365	-0.365368
1	0.01819	-3.312928	-0.514616
6	-1.682053	2.546626	0.113323
1	-1.753136	3.627278	0.186553
6	-1.348463	-1.654233	-0.220014
6	-1.469811	-0.246998	-0.09173
6	0.785469	2.722009	0.019564
1	0.709733	3.802672	0.082292
6	0.996327	-0.060747	-0.160561
6	-0.280358	0.550174	-0.073302
6	-5.309287	0.065162	0.116156
6	3.776928	-0.557553	1.156279
1	2.899361	-1.072234	1.54983
1	4.068537	0.203107	1.903527
6	2.0323	2.122485	-0.029676
1	2.919447	2.742598	0.005707
6	-3.909638	-0.465029	0.004716
6	4.538386	0.847298	-0.660072
1	4.880424	1.637632	0.034385
1	4.207052	1.335571	-1.579133
6	-2.502389	-2.437869	-0.228107
1	-2.416044	-3.514435	-0.326469
6	5.706559	-0.09131	-0.948309
1	6.543597	0.485109	-1.352881
1	5.39692	-0.808117	-1.716135
6	-3.743629	-1.850069	-0.114463
1	-4.612117	-2.49484	-0.122773
6	4.924663	-1.538317	0.948049
1	4.582427	-2.347033	0.292376
1	5.197435	-1.987354	1.907456
6	6.127767	-0.838097	0.316882
1	6.919527	-1.558208	0.093439
1	6.543286	-0.120842	1.036213
6	-6.461859	-0.927686	0.093308
1	-6.480098	-1.49722	-0.83941

1	-6.393378	-1.638078	0.921182
1	-7.384668	-0.358118	0.184411

Table S60. Minimum excited singlet state in XYZ format for **PK calculated by ωB97XD/6-311+G(d,p)**.

	Coordinates (Å)		
Atom No.	X	Y	Z
8	-5.558597	1.232848	0.246099
7	3.431173	0.227076	0.031238
6	1.109753	-1.435041	-0.317054
1	2.077444	-1.886028	-0.489267
6	-2.80481	1.771025	0.088806
1	-3.78034	2.228947	0.15238
6	-0.389798	1.98364	0.01061
6	-2.740075	0.356524	0.003665
6	2.178702	0.805816	-0.031683
6	-0.030746	-2.213916	-0.358742
1	0.055808	-3.285838	-0.504345
6	-1.668428	2.552507	0.080964
1	-1.76288	3.632321	0.128294
6	-1.308035	-1.654463	-0.223527
6	-1.449179	-0.239123	-0.086446
6	0.787717	2.785492	-0.020931
1	0.687044	3.864393	0.014905
6	1.013166	-0.040788	-0.108663
6	-0.273579	0.564018	-0.05013
6	-5.289015	0.043913	0.11583
6	3.73663	-0.694306	1.131015
1	2.819136	-1.182521	1.454249
1	4.102388	-0.094923	1.981374
6	2.028077	2.20844	-0.046601
1	2.909125	2.837111	-0.010838
6	-3.917774	-0.480081	-0.004678
6	4.595233	0.958527	-0.445855
1	4.993676	1.612817	0.348596
1	4.290313	1.590286	-1.280678
6	-2.48813	-2.446555	-0.24249
1	-2.393758	-3.522373	-0.347521

6	5.682272	-0.01586	-0.89935
1	6.558523	0.556209	-1.216976
1	5.314471	-0.564652	-1.772583
6	-3.726747	-1.872668	-0.132396
1	-4.589584	-2.525629	-0.147574
6	4.798328	-1.708447	0.726851
1	4.392345	-2.361866	-0.05339
1	5.036023	-2.338604	1.588094
6	6.049147	-0.998486	0.211045
1	6.783191	-1.723672	-0.149026
1	6.519924	-0.45205	1.037988
6	-6.447793	-0.945539	0.077792
1	-6.470353	-1.509676	-0.859056
1	-6.3939	-1.664094	0.90087
1	-7.367529	-0.370253	0.168858

Table S61. Franck-Condon state in XYZ format for **Prodan calculated by ωB97XD/6-31G(d,p)**.

	Coordinates (Å)		
Atom No.	X	Y	Z
6	2.480978	-0.131785	-0.057409
6	1.570596	0.918585	-0.036298
1	1.908679	1.947658	-0.026617
6	0.17793	0.689319	-0.024636
6	-0.320781	-0.641651	-0.023996
6	0.619257	-1.702675	-0.033944
1	0.255983	-2.726886	-0.027847
6	1.964582	-1.466491	-0.047702
1	2.643045	-2.310256	-0.051536
6	-0.761194	1.760053	-0.008019
1	-0.389523	2.780963	-0.007535
6	-2.104814	1.516948	0.005938
1	-2.825165	2.327469	0.017815
6	-2.606142	0.186842	0.005208
6	-1.716612	-0.864849	-0.009129
1	-2.06959	-1.892595	-0.009227
6	4.341831	1.43636	0.031953
1	3.963838	2.068564	-0.779466

1	5.429918	1.429451	-0.03674
1	4.05869	1.902511	0.987369
6	4.759153	-1.018903	0.117373
1	4.610101	-1.514008	1.087469
1	5.782307	-0.643761	0.084793
1	4.665351	-1.773654	-0.67142
6	-4.086068	-0.007176	0.020651
6	-4.637266	-1.419807	0.019557
1	-5.72555	-1.36841	0.031072
1	-4.289142	-1.974165	0.896745
1	-4.307293	-1.96587	-0.869743
7	3.84443	0.084051	-0.093482
8	-4.83892	0.949501	0.033996

Table S62. Minimum excited singlet state in XYZ format for **Prodan calculated by ωB97XD/6-31G(d,p)**.

	Coordinates (Å)		
Atom No.	X	Y	Z
6	2.48805	-0.134974	-0.086064
6	1.575345	0.911692	-0.055866
1	1.910809	1.942027	-0.044493
6	0.183733	0.681635	-0.037547
6	-0.323092	-0.646863	-0.036181
6	0.622712	-1.70459	-0.05236
1	0.263249	-2.730161	-0.044206
6	1.96915	-1.465446	-0.07373
1	2.645837	-2.310977	-0.08177
6	-0.755578	1.755313	-0.012779
1	-0.381601	2.775713	-0.012279
6	-2.097123	1.527504	0.008612
1	-2.790842	2.361768	0.026202
6	-2.619942	0.187861	0.008157
6	-1.714508	-0.87706	-0.013655
1	-2.066801	-1.904083	-0.013482
6	4.345402	1.431889	0.047995
1	3.95297	2.095212	-0.730145
1	5.432934	1.438754	-0.036234
1	4.069736	1.854868	1.02692

6	4.760155	-1.006457	0.178527
1	4.592588	-1.424496	1.182667
1	5.787708	-0.643909	0.128935
1	4.671508	-1.819456	-0.549601
6	-4.022431	-0.026196	0.030066
6	-4.669309	-1.396744	0.031372
1	-5.756147	-1.310308	0.051711
1	-4.343598	-1.959238	0.912621
1	-4.375857	-1.945869	-0.869421
7	3.859632	0.082745	-0.139194
8	-4.885663	0.946945	0.050956

Table S63. Franck-Condon state in XYZ format for **FπCMst calculated by B3LYP/6-311+G(d,p)**.

	Coordinates (Å)		
Atom No.	X	Y	Z
6	-5.425584	0.935398	0.104051
6	3.842808	0.891102	-0.170528
6	4.898177	1.781457	-0.365061
6	6.209425	1.323695	-0.327159
6	6.507674	-0.04171	-0.092591
1	5.613127	-1.985241	0.286328
1	4.711845	2.834666	-0.547594
1	7.010105	2.033378	-0.481156
6	2.393772	1.080706	-0.156787
6	1.606119	2.219923	-0.328494
6	1.780031	-0.164445	0.081147
6	0.220349	2.106515	-0.258978
1	2.058734	3.186192	-0.52355
6	0.401528	-0.271119	0.144184
6	-0.406906	0.870654	-0.024136
1	-0.390117	2.988084	-0.418192
1	-0.065996	-1.22788	0.352384
6	2.831369	-1.262443	0.25072
6	4.124349	-0.465798	0.063407
6	2.681914	-2.355259	-0.829462
1	3.479115	-3.09881	-0.739678
1	1.725197	-2.874849	-0.723905
1	2.728589	-1.923996	-1.831912
6	2.76018	-1.894028	1.657931
1	2.86468	-1.132852	2.434246

1	1.803812	-2.403861	1.805107
1	3.55803	-2.630343	1.791239
6	-1.882409	0.770464	0.043528
6	-2.657572	1.783137	0.629182
6	-2.561622	-0.347084	-0.477919
6	-4.041256	1.683387	0.688414
1	-2.168507	2.645912	1.066053
6	-3.941832	-0.447612	-0.417729
1	-1.99536	-1.134754	-0.961354
6	-4.718534	0.568085	0.168354
1	-4.614274	2.477508	1.155843
1	-4.423326	-1.321214	-0.841488
7	7.816367	-0.48833	-0.056629
6	8.913622	0.441167	-0.260447
1	9.856421	-0.09971	-0.196521
1	8.866755	0.919905	-1.246641
1	8.927222	1.231936	0.500173
6	8.102933	-1.890726	0.186405
1	7.666339	-2.537799	-0.585057
1	9.181084	-2.04224	0.177685
1	7.724725	-2.223152	1.161546
6	-6.173912	0.519425	0.260132
6	-6.995062	-0.448388	-0.182517
1	-6.647209	1.370495	0.744392
1	-6.641223	-1.342344	-0.68064
6	-8.45252	-0.330639	-0.004168
8	-9.086213	-1.414501	-0.522057
8	-9.043007	0.586498	0.524365
6	-10.518208	-1.415764	-0.408232
1	-10.84627	-2.345801	-0.867879
1	-10.819394	-1.378592	0.639943
1	-10.944912	-0.558131	-0.930911

Table S64. Minimum excited singlet state in XYZ format for **FπCMst calculated by B3LYP/6-311+G(d,p)**.

	Coordinates (Å)		
Atom No.	X	Y	Z
6	5.406169	-0.95891	0.116744
6	3.837372	0.874325	-0.18566
6	4.898104	1.772447	-0.392277
6	6.199477	1.317519	-0.347882

6	6.492667	-0.056091	-0.092909
1	5.59917	-2.004737	0.311055
1	4.703988	2.820557	-0.585225
1	7.004252	2.020179	-0.507405
6	2.403825	1.072411	-0.17312
6	1.629651	2.224332	-0.364247
6	1.781821	-0.166782	0.086991
6	0.249399	2.119124	-0.306884
1	2.094549	3.183015	-0.568105
6	0.401256	-0.255176	0.169626
6	-0.396623	0.887372	-0.034984
1	-0.362467	2.994628	-0.484248
1	-0.084509	-1.1951	0.404099
6	2.816397	-1.278182	0.261436
6	4.115527	-0.497517	0.068059
6	2.653605	-2.37106	-0.8179
1	3.428093	-3.13699	-0.717756
1	1.682972	-2.861003	-0.714435
1	2.713674	-1.947311	-1.822708
6	2.74217	-1.904593	1.671166
1	2.866879	-1.147157	2.448005
1	1.772837	-2.385256	1.819885
1	3.516767	-2.665879	1.801299
6	-1.874838	0.803721	0.036736
6	-2.632764	1.801912	0.683696
6	-2.575109	-0.275488	-0.528196
6	-4.009931	1.711379	0.770495
1	-2.129644	2.643134	1.150521
6	-3.955882	-0.363383	-0.461766
1	-2.028029	-1.049422	-1.058823
6	-4.735363	0.629341	0.200359
1	-4.561832	2.487341	1.290982
1	-4.450389	-1.205445	-0.929735
7	7.786046	-0.496257	-0.051453
6	8.899009	0.423816	-0.279737
1	9.833181	-0.128015	-0.220036
1	8.833341	0.882968	-1.270057
1	8.917813	1.21551	0.474861
6	8.088087	-1.899786	0.223079
1	7.660129	-2.552593	-0.543268
1	9.166021	-2.036937	0.226511
1	7.699472	-2.202118	1.199595

6	-6.163783	0.580333	0.308511
6	-6.99632	-0.411255	-0.176024
1	-6.641875	1.405948	0.826577
1	-6.61326	-1.274451	-0.705422
6	-8.419769	-0.353183	-0.002736
8	-9.045124	-1.45945	-0.57107
8	-9.078903	0.51751	0.557753
6	-10.462927	-1.483928	-0.447624
1	-10.789114	-2.40168	-0.938045
1	-10.770429	-1.489389	0.601669
1	-10.919047	-0.616678	-0.932813

Table S65. Franck-Condon state in XYZ format for **PCMst calculated by B3LYP/6-311+G(d,p)**.

	Coordinates (Å)		
Atom No.	X	Y	Z
7	4.543072	0.131411	-0.106729
6	2.183281	-1.501813	-0.304699
1	3.158345	-1.948	-0.448791
6	-1.739717	1.73918	0.093532
1	-2.705468	2.223197	0.14023
6	0.686506	1.938604	-0.007116
6	-1.665245	0.305132	0.036744
6	3.263102	0.740622	-0.103697
6	1.065104	-2.277346	-0.316211
1	1.146344	-3.350972	-0.450625
6	-0.621273	2.512127	0.069953
1	-0.709335	3.593063	0.107182
6	-0.239048	-1.70733	-0.177335
6	-0.367019	-0.29245	-0.066273
6	1.848365	2.718708	-0.035701
1	1.761008	3.799781	-0.002166
6	2.10299	-0.080229	-0.145635
6	0.810345	0.520606	-0.065641
6	4.921794	-0.543568	1.15056
1	4.058132	-1.085131	1.53577
1	5.193375	0.211738	1.910305
6	3.106484	2.132527	-0.074909
1	3.981364	2.769598	-0.059101
6	-2.818636	-0.529941	0.049589
6	5.653289	0.912483	-0.658177

1	5.973402	1.709721	0.037947
1	5.312328	1.395911	-1.576066
6	-1.399451	-2.500055	-0.167975
1	-1.304262	-3.578165	-0.24073
6	6.850643	0.003677	-0.952423
1	7.673385	0.611204	-1.34301
1	6.569512	-0.706525	-1.737636
6	-2.647777	-1.923035	-0.052047
1	-3.520393	-2.563705	-0.014004
6	6.100545	-1.492959	0.930446
1	5.782915	-2.30942	0.271871
1	6.384157	-1.941206	1.888102
6	7.290812	-0.756752	0.303914
1	8.095491	-1.458932	0.065938
1	7.699977	-0.045377	1.032922
6	-4.156449	0.037097	0.186379
6	-5.323935	-0.559216	-0.117636
1	-4.23917	1.044437	0.579536
1	-5.38991	-1.550839	-0.547189
6	-6.597868	0.145192	0.106644
8	-6.734737	1.261569	0.559717
8	-7.646417	-0.630382	-0.271149
6	-8.948821	-0.048184	-0.101776
1	-9.652484	-0.803585	-0.445384
1	-9.127017	0.193637	0.947255
1	-9.042109	0.861738	-0.696868

Table S66. Minimum excited singlet state in XYZ format for **PCMst calculated by B3LYP/6-311+G(d,p)**.

	Coordinates (Å)		
Atom No.	X	Y	Z
7	4.555009	0.240973	0.058594
6	2.21927	-1.469164	-0.261216
1	3.187196	-1.927831	-0.399285
6	-1.715745	1.756935	0.037425
1	-2.682103	2.239466	0.077759
6	0.70229	1.976602	0.007272
6	-1.641603	0.331125	-0.042161
6	3.276276	0.79883	-0.001846
6	1.081986	-2.242008	-0.320051
1	1.170326	-3.31565	-0.454922

6	-0.593665	2.54717	0.052035
1	-0.690865	3.626293	0.096955
6	-0.209747	-1.680729	-0.221256
6	-0.350543	-0.259149	-0.101875
6	1.869764	2.780418	0.002207
1	1.768487	3.859116	0.038794
6	2.122132	-0.052126	-0.081369
6	0.828967	0.550308	-0.047359
6	4.894237	-0.677854	1.158048
1	3.991096	-1.18686	1.487299
1	5.247798	-0.067605	2.006492
6	3.118565	2.203066	-0.008823
1	3.995178	2.836072	0.038728
6	-2.827535	-0.490513	-0.069429
6	5.703213	0.985524	-0.45786
1	6.103537	1.659453	0.318766
1	5.369558	1.599894	-1.293978
6	-1.388162	-2.464727	-0.262043
1	-1.294198	-3.542095	-0.35747
6	6.811065	0.025964	-0.914084
1	7.668673	0.618848	-1.246413
1	6.452391	-0.538675	-1.781126
6	-2.637434	-1.894867	-0.184726
1	-3.505975	-2.539656	-0.215588
6	5.986226	-1.669634	0.753961
1	5.590238	-2.352663	-0.005617
1	6.250649	-2.275964	1.625311
6	7.216493	-0.938187	0.205506
1	7.956915	-1.655193	-0.16001
1	7.696896	-0.375501	1.015995
6	-4.147359	0.073244	0.015838
6	-5.338371	-0.603447	-0.016358
1	-4.245173	1.146507	0.117312
1	-5.408141	-1.679028	-0.112891
6	-6.594707	0.117753	0.079382
8	-6.741886	1.325391	0.191927
8	-7.664742	-0.740915	0.028371
6	-8.953666	-0.127027	0.115707
1	-9.672679	-0.943274	0.061304
1	-9.064349	0.414775	1.057695
1	-9.109178	0.571918	-0.7092

Table S67. Franck-Condon state in XYZ format for **FstCM calculated by B3LYP/6-311+G(d,p)**.

	Coordinates (Å)		
Atom No.	X	Y	Z
6	5.521459	-0.917021	0.041905
6	4.008169	0.987046	0.026983
6	5.098511	1.856756	0.017737
6	6.391559	1.349294	0.024299
6	6.638278	-0.045645	0.048616
1	5.666423	-1.988944	0.041781
1	4.953277	2.931818	0.000403
1	7.218188	2.04529	0.010252
6	2.568532	1.227063	0.018542
6	1.825675	2.407657	0.008762
6	1.904534	-0.019163	0.022324
6	0.436473	2.331767	0.003134
1	2.315197	3.375479	0.005998
6	0.525829	-0.086394	0.016758
6	-0.24386	1.098687	0.007064
1	-0.145553	3.247778	-0.004307
1	0.0303	-1.051155	0.019733
6	2.914901	-1.168141	0.032659
6	4.237939	-0.399536	0.036235
6	2.778869	-2.046236	-1.22997
1	3.548852	-2.823008	-1.244918
1	1.803145	-2.539809	-1.254304
1	2.880955	-1.446384	-2.137025
6	2.765552	-2.035394	1.301226
1	2.85932	-1.428022	2.204153
1	1.789181	-2.527932	1.320087
1	3.53463	-2.812657	1.330378
7	7.934265	-0.542166	0.090009
6	9.055381	0.357509	-0.131145
1	9.984416	-0.201512	-0.026753
1	9.04023	0.818123	-1.129889
1	9.069694	1.158187	0.613427
6	8.162362	-1.964341	-0.107604
1	7.835127	-2.314163	-1.098011
1	9.227251	-2.170422	-0.007635
1	7.64203	-2.555193	0.651528
6	-1.703882	1.109241	0.001163
6	-2.53293	0.04542	0.00515

1	-2.139714	2.104962	-0.007191
1	-2.104961	-0.953284	0.013847
6	-3.994094	0.073871	-0.000842
6	-4.695236	-1.146764	0.005426
6	-4.754902	1.260676	-0.012608
6	-6.082569	-1.189792	0.000301
1	-4.134177	-2.075448	0.014522
6	-6.138378	1.222802	-0.017742
1	-4.258903	2.223634	-0.017786
6	-6.821914	-0.001886	-0.011372
1	-6.59829	-2.141049	0.005319
1	-6.716209	2.13892	-0.026768
6	-8.306628	0.020452	-0.017356
8	-8.982091	1.026128	-0.027408
8	-8.844932	-1.222099	-0.01034
6	-10.280978	-1.281798	-0.015481
1	-10.680808	-0.800174	-0.909072
1	-10.527494	-2.341528	-0.00879
1	-10.687692	-0.787358	0.867945

Table S68. Minimum excited singlet state in XYZ format for **FstCM calculated by B3LYP/6-311+G(d,p)**.

	Coordinates (Å)		
Atom No.	X	Y	Z
6	5.475538	-0.952077	0.000482
6	4.003373	0.987165	-0.000491
6	5.114929	1.845315	-0.001158
6	6.393247	1.316791	-0.001007
6	6.611064	-0.089987	-0.000181
1	5.60842	-2.025438	0.001118
1	4.98263	2.921031	-0.001788
1	7.235882	1.99337	-0.001522
6	2.581844	1.253225	-0.000428
6	1.861053	2.46017	-0.000936
6	1.888018	0.022786	0.000385
6	0.480553	2.417195	-0.000697
1	2.374116	3.416128	-0.001524
6	0.507971	-0.018178	0.000681
6	-0.245206	1.189285	0.000059
1	-0.08266	3.343925	-0.001131
1	-0.007668	-0.970706	0.001553
6	2.868082	-1.152985	0.000919

6	4.209091	-0.418694	0.000317
6	2.705135	-2.021602	-1.265155
1	3.443012	-2.829218	-1.282429
1	1.71086	-2.474583	-1.289086
1	2.826391	-1.42363	-2.171015
6	2.705468	-2.02025	1.267944
1	2.826947	-1.421319	2.173141
1	1.711208	-2.473226	1.292616
1	3.443353	-2.827843	1.285892
7	7.885375	-0.604335	-0.000003
6	9.044155	0.280326	-0.000927
1	9.950683	-0.32017	-0.000427
1	9.057275	0.919672	-0.890023
1	9.057404	0.921292	0.886992
6	8.103068	-2.045651	0.000575
1	7.66772	-2.515574	-0.887794
1	9.172103	-2.244538	0.000829
1	7.667463	-2.514872	0.889178
6	-1.691341	1.229846	0.00024
6	-2.52139	0.133641	-0.000573
1	-2.128622	2.222331	0.000841
1	-2.063723	-0.852899	-0.001776
6	-3.955859	0.116546	-0.000252
6	-4.644793	-1.126278	-0.001947
6	-4.755496	1.297526	0.001726
6	-6.022273	-1.198693	-0.00177
1	-4.063541	-2.044034	-0.003458
6	-6.130869	1.227857	0.001922
1	-4.27898	2.270921	0.003179
6	-6.802477	-0.016401	0.000164
1	-6.517937	-2.160511	-0.003107
1	-6.728865	2.131364	0.003456
6	-8.263967	-0.0222	0.00045
8	-8.982285	0.965637	0.002083
8	-8.787848	-1.28984	-0.001397
6	-10.215922	-1.365285	-0.001255
1	-10.632171	-0.88141	-0.887518
1	-10.453985	-2.428276	-0.002876
1	-10.631849	-0.88419	0.88667

S8. References

S1. O. A. Kucherak, P. Didier, Y. Mély, A. S. Klymchenko, *J. Phys. Chem. Lett*. **2010**, *1*, 616.

S2. S. Cao, X. Tian, M. Cao, J. Wang, G. Niu, B. Z. Tang, *Chem. Mater*. **2023**, *35*, 2472.

S3. Y. Niko, S. Kawauchi, G. Konishi, *Chem. Eur. J*. **2013**, *19*, 9760.

S4. C. Reichardt, *Chem. Rev.* **1994**, *94*, 2319.

S5. J. Shaya, F. F. Vivi, B. Y. Michel, A. Burger, *Chem. Eur. J*. **2016**, *22*, 10627.

S6. W. Siebrand, *J. Chem. Phys*. **1967**, *47*, 2411.

S7. D. Tsuzuki, T. Uchimaru, *Phys. Chem. Chem. Phys*. **2020**, *22*, 22508.

S8. B. Paizs, S. Suhai, *J. Comput. Chem*. **1998**, *19*, 575.

S9. E. L. Lippert, Organic Molecular Photophysics; Wiley-Interscience: New York, 1975.

S10. N. Mataga, T. Kubota, Molecular Interactions and Electronic Spectra; M. Dekker, New York, 1970.

S11. O. V. Dolomanov, L. J. Bourthis, R. J. Glidea, J. A. K. Howard, H. Puschmann, OLEX2, A complete Structure Solution, Refinement and Analysis Program. *J. Appl. Cryst.* **2009**, *42*, 339.

S12. G. M. Sheldrick, *SHELXT*-Integrated space-group and crystal-structure determination. *Acta Cryst.* 2015*, A71*, 3.

S13. G. M. Sheldrick, Crystal structure refinement with *SHELXL. Acta Cryst.* **2015**, *C71*, 3.

S14. D. Kratzert, J. J. Holstein, I. Krossing, DSR: enhanced modeling and refinement disordered structures with *SHELXL*. *J. Appl. Cryst.* **2018**, *48*, 933.

S15. D. Kratzert,I. Krossing*,* Recent improvements in *DSR*. *J. Appl. Cryst.* **2018**, *51*, 928.

S16. Gaussian 09, Revision B.01, M. J. Frisch, G. W. Trucks, H. B. Schlegel, G. E. Scuseria, M. A. Robb, J. R. Cheeseman, G. Scalmani, V. Barone, B. Mennucci, G. A. Petersson, H. Nakatsuji, M. Caricato, X. Li, H. P. Hratchian, A. F. Izmaylov, J. Bloino, G. Zheng, J. L. Sonnenberg, M. Hada, M. Ehara, K. Toyota, R. Fukuda, J. Hasegawa, M. Ishida, T. Nakajima, Y. Honda, O. Kitao, H. Nakai, T. Vreven, J. A. Montgomery, Jr., J. E. Peralta, F. Ogliaro, M. Bearpark, J. J. Heyd, E. Brothers, K. N. Kudin, V. N. Staroverov, R. Kobayashi, J. Normand, K. Raghavachari, A. Rendell, J. C. Burant, S. S. Iyengar, J. Tomasi, M. Cossi, N. Rega, J. M. Millam, M. Klene, J. E. Knox, J. B. Cross, V. Bakken, C. Adamo, J. Jaramillo, R. Gomperts, R. E. Stratmann, O. Yazyev, A. J. Austin, R. Cammi, C. Pomelli, J. W. Ochterski, R. L. Martin, K. Morokuma, V. G. Zakrzewski, G. A. Voth, P. Salvador, J. J. Dannenberg, S. Dapprich, A. D. Daniels, Ö. Farkas, J. B. Foresman, J. V. Ortiz, J. Cioslowski, D. J. Fox, Gaussian, Inc., Wallingford CT, 2009.

S17. Gaussian 16, Revision C.01, M. J. Frisch, G. W. Trucks, H. B. Schlegel, G. E. Scuseria, M. A. Robb, J. R. Cheeseman, G. Scalmani, V. Barone, G. A. Petersson, H. Nakatsuji, X.

Li, M. Caricato, A. V. Marenich, J. Bloino, B. G. Janesko, R. Gomperts, B. Mennucci, H. P. Hratchian, J. V. Ortiz, A. F. Izmaylov, J. L. Sonnenberg, D. Williams-Young, F. Ding, F. Lipparini, F. Egidi, J. Goings, B. Peng, A. Petrone, T. Henderson, D. Ranasinghe, V. G. Zakrzewski, J. Gao, N. Rega, G. Zheng, W. Liang, M. Hada, M. Ehara, K. Toyota, R. Fukuda, J. Hasegawa, M. Ishida, T. Nakajima, Y. Honda, O. Kitao, H. Nakai, T. Vreven, K. Throssell, J. A. Montgomery, Jr., J. E. Peralta, F. Ogliaro, M. J. Bearpark, J. J. Heyd, E. N. Brothers, K. N. Kudin, V. N. Staroverov, T. A. Keith, R. Kobayashi, J. Normand, K. Raghavachari, A. P. Rendell, J. C. Burant, S. S. Iyengar, J. Tomasi, M. Cossi, J. M. Millam, M. Klene, C. Adamo, R. Cammi, J. W. Ochterski, R. L. Martin, K. Morokuma, O. Farkas, J. B. Foresman, and D. J. Fox, Gaussian, Inc., Wallingford CT, 2019.

S18. P. R. Spackman, M. J. Turner, S. K. Wolff, D. J. Grimwood, D. Jayatilaka, M. A. Spackman, *CrystalExplorer*: a program for Hirshfeld surface analysis, visualization and quantitative analysis of molecular crystals. *J. Appl. Cryst.* **2021**, *54*, 1006.
